# Bringing Selectivity in H/D Exchange Reactions Catalyzed by Metal Nanoparticles through Modulation of the Metal and the Ligand Shell

**DOI:** 10.1021/acs.inorgchem.2c04442

**Published:** 2023-03-09

**Authors:** Francisco Martinez-Espinar, Antoni Salom-Català, Emma Bresó-Femenia, Carmen Claver, Francesca Baletto, Josep M. Ricart, Bruno Chaudret, Jorge J. Carbó, Cyril Godard, Sergio Castillon

**Affiliations:** † Departament de Química Física i Inorgànica, Universitat Rovira i Virgili, C/ Marcel·lí Domingo s/n, 43007 Tarragona, Spain; ‡ Laboratoire de Physique et Chimie des Nano Objets, LPCNO, UMR5215 INSA-UPS-CNRS, Université de Toulouse, Institut National des Sciences Appliquées, 135 Avenue de Rangueil, 31077 Toulouse, France; § Departament de Química Analítica i Orgànica, Universitat Rovira i Virgili, C/ Marcel·lí Domingo s/n, 43007 Tarragona, Spain; ∥ Department of Physics, King’s College London, London, Strand Building, Strand WC2R 2LS, United Kingdom

## Abstract

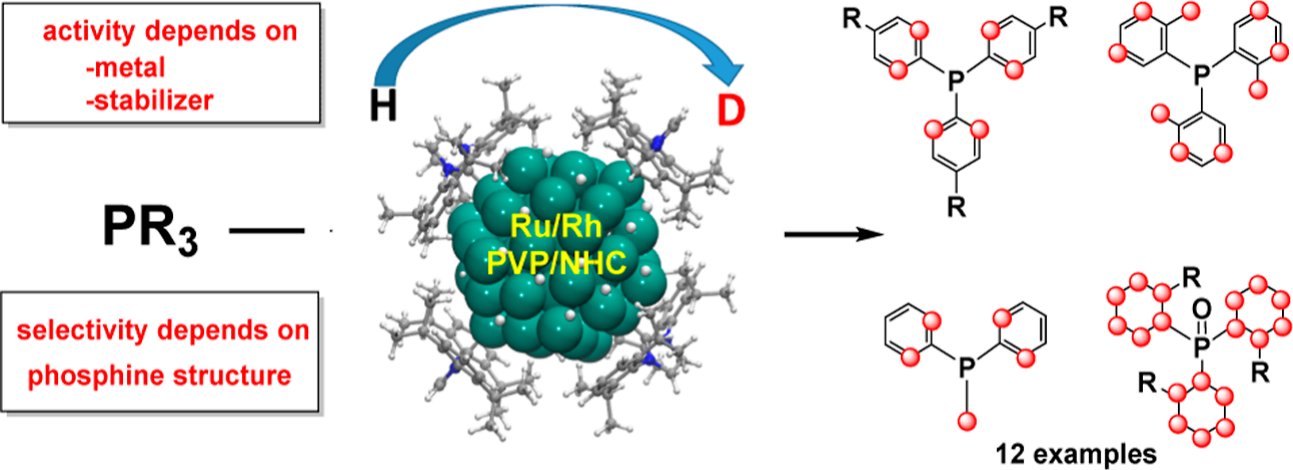

Ru and Rh nanoparticles catalyze the selective H/D exchange in phosphines using D_2_ as the deuterium source. The position of the deuterium incorporation is determined by the structure of the P-based substrates, while activity depends on the nature of the metal, the properties of the stabilizing agents, and the type of the substituent on phosphorus. The appropriate catalyst can thus be selected either for the exclusive H/D exchange in aromatic rings or also for alkyl substituents. The selectivity observed in each case provides relevant information on the coordination mode of the ligand. Density functional theory calculations provide insights into the H/D exchange mechanism and reveal a strong influence of the phosphine structure on the selectivity. The isotope exchange proceeds via C–H bond activation at nanoparticle edges. Phosphines with strong coordination through the phosphorus atom such as PPh_3_ or PPh_2_Me show preferred deuteration at ortho positions of aromatic rings and at the methyl substituents. This selectivity is observed because the corresponding C–H moieties can interact with the nanoparticle surface while the phosphine is P-coordinated, and the C–H activation results in stable metallacyclic intermediates. For weakly coordinating phosphines such as P(*o*-tolyl)_3_, the interaction with the nanoparticle can occur directly through phosphine substituents, and then, other deuteration patterns are observed.

## Abstract 2

Ru and Rh nanoparticles catalyze the selective H/D exchange in phosphines using D_2_ as the deuterium source. The position of the deuterium incorporation is determined by the structure of the P-based substrates, while activity depends on the nature of the metal, the properties of the stabilizing agents, and the type of the substituent on phosphorus. The selectivity observed in each case provides relevant information on the coordination mode of the ligand.

## Introduction

Over the last years, hydrogen isotope exchange (HIE) reactions have attracted much interest due to the increasing importance of isotope-containing molecules in various areas including materials and life sciences, in addition to their established utilization in mechanistic studies in chemistry and biology.^1,2^ Moreover, C–H activation has become a powerful technique for the synthesis or functionalization of complex organic compounds via the subsequent formation of a large variety of C–C, C–N, C–O, and C–B bonds.^3^

In this context, several methodologies based on H/D exchange using homogeneous and heterogeneous metal catalysts have been reported.^4^ Currently, the use of well-defined heterogeneous catalysts, and particularly metal nanoparticles (M-NPs), is of high interest for academic and industrial chemists.^5,6^ Regarding the H/D exchange using M-NPs, the labeling of N-containing compounds has been mainly studied.^7^ Sullivan and co-workers reported the application of Pd NPs stabilized by 4-dimethylaminopyridine (DMAP) for the selective H/D exchange of pyridine-based compounds using D_2_O as the deuterium source.^8^

This catalytic system promoted selective H/D exchange of protons in the α position of the endocyclic N atom of DMAP. The use of Pd NPs stabilized by polyvinylpyrrolidone (PVP) was also investigated for the HIE of N-based molecules such as pyridines, *N*-methylimidazole, and quinoline, indicating interactions between the NP surface and the N atom in all cases.^9,10^

Mobile and reactive hydride species at the surface of Ru NPs stabilized by hexadecylamine undergo H/D exchange when exposed to a D_2_ atmosphere,^11^ and H/D exchange in pyridine^12^ derivatives and phosphine oxides^13^ used as stabilizers was also observed by ^2^D magic angle spinning (MAS) NMR after exposure to D_2_.

**Scheme 1:**
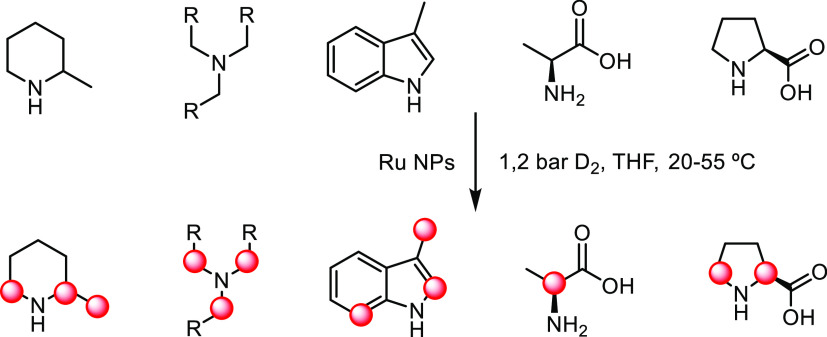
H/D Exchange of Amino Derivatives Using Ru NPs; refs (14) and (15)

Later, some of us reported the application of Ru NPs stabilized by PVP for the regioselective and stereospecific H/D exchange of N-containing substrates under mild reaction conditions using D_2_ as the deuterium source (Scheme 1).^14^ The D labeling of pyridines, quinolines, indoles, and alkyl amines, with high isotopic enrichment in positions close to the N atom suggested the direct coordination of the N atom to the surface of the Ru NPs. Following the same methodology, the enantiospecific deuterium incorporation at the stereogenic center of amino acids was also achieved.^15^ More recently, water-soluble Ru NPs stabilized by sulfonated NHC ligands were also reported in the H/D exchange of l-lysine.^16^ These catalytic systems allowed an efficient and selective late-stage H/D exchange in complex molecules.

Catalytic deuterium incorporation in phosphorus-based compounds has not been reported, and only stoichiometric labeling in molecular complexes has been described.^17^ In this context, we ambitioned to develop the catalytic labeling of phosphorus ligands using NPs, aiming at gaining information on their coordination mode at metallic surfaces.^18^ We previously reported that Ru NPs stabilized by PVP (**Ru@PVP**) efficiently catalyze the H/D exchange in the ortho position of aryl phosphines such as PPh_3_ and derivatives. In contrast, the alkyl group of alkylphosphines remained unaltered and O=PPh_3_ underwent reduction of the ring.^19^ These results showed that phosphines coordinate to the Ru NP surface through the P atom, although not exclusively, and that reactivities of aryl and alkyl substituents are different. With these results in hand, the next step was to understand how the activity and selectivity of H/D exchange varies as a function of the metal type, coordination sphere of NPs (stabilizer), and phosphine type. We report here a comparative study on the use of Ru versus Rh NPs stabilized by N-heterocyclic carbene (IPr)^20^ and PVP^19^ for the H/D exchange of a range of structurally distinct P ligands using D_2_ as the deuterium source. We show that activity depends on both the metal and the stabilizer. Density functional theory (DFT) calculations provide atomistic information on ligand coordination and on the C–H activation mechanism at the NP surface, which explain the observed selectivity.

## Results and Discussion

### H/D Exchange in Phosphines Catalyzed by Ru and Rh NPs

**Figure 1:**
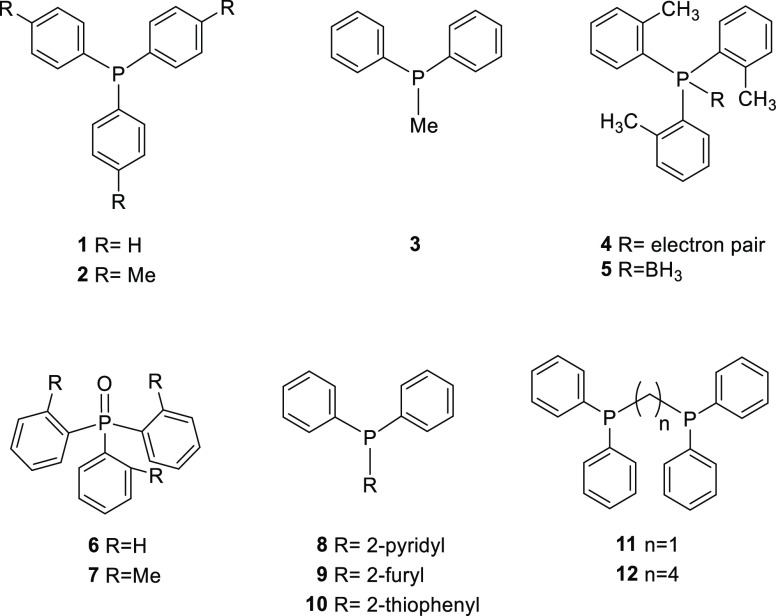
Selected phosphines for H/D exchange using NPs as catalysts and D_2_ as the deuterium source.

For this study, a set of structurally distinct phosphines was selected such as aryl phosphines (**1** and **2**) with different cone angles (**4** and **5**), mixed aryl/alkyl phosphine (**3**), phosphine oxides (**6** and **7**), phosphines containing heteroatoms (**8–10**), and diphosphines (**11** and **12**) (Figure 1). No deuteration of the solvents used was observed.

The nanocatalysts used in this work, **Ru@NHC**,^20^**Rh@NHC**,^21^**Ru@PVP**,^19^ and **Rh@PVP**,^22^ were prepared following procedures previously reported by our groups. All the NPs exhibited small sizes (<2 nm), spherical shapes, and narrow size distributions. The Ru and Rh NPs presented hcp and fcc packing, respectively, with the metals in the zero oxidation state. In the case of Rh@NHC, the presence of a protonated ligand was detected and attributed to a second coordination sphere stabilizer.^21^ Based on previous reports, deuteration of these ligands is to be expected under a deuterium atmosphere. It was not investigated in this work.^23^

Initially, H/D exchange in PPh_3_ (**1**) was tested using **Ru@NHC** NPs as the catalyst. For the sake of comparison, catalytic experiments were performed under the reaction conditions used in our previous report:^19^ tetrahydrofuran (THF) as the solvent, a D_2_ pressure of 2 bar, and a temperature of 55 °C for 48 h.

**Figure 2:**
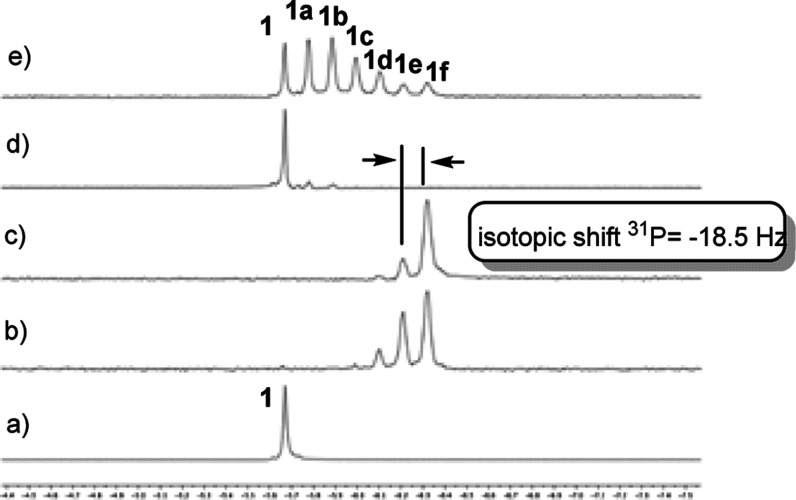
^31^P{^1^H} NMR spectra of (a) phosphine **1** and of the reaction mixtures after 48 h of reaction under 2 bar of D_2_ (D/H ratio = *ca*. 5) at 55 °C using (b) Ru@PVP, (c) Ru@NHC, (d) Rh@PVP, and (e) Rh@NHC.

The reaction was monitored by ^31^P, ^13^C, and ^2^H NMR spectroscopy as well as mass spectrometry.^19^ Up to six different products were formed during this reaction by successive introduction of deuterium atoms in the ortho position of the phenyl rings of the substrate (products **1a–f**, Table 1). Figure 2 displays the spectrum of **1** and the spectra of the reaction mixtures after 48 h of the reaction using the different catalysts (see also Figures S1–S3 in the Supporting Information). Successive deuterated products show distinct signals in ^31^P NMR due to an isotopic shift, which facilitates quantification of the deuterium content by integration of the corresponding signals.

**Table 1:** Selective H/D Exchange of the Aryl Phosphines **1** and **2** Using Ru and Rh NPs^a^^,^^b^^,^^c^

Entry	PR_3_	NPs	P	a	b	c	d	e	f
1^19^	**1**	Ru@PVP^d^	**1**	0	0	2	12	35	51
2^19^	**1**	Ru@PVP^d^^,^^f^	**1**	0	0	0	0	7	93
3	**1**	Rh@PVP^e^	**1**	1	3	1	0	0	0
4	**1**	Ru@NHC^e^	**1**	0	0	0	0	17	83
5	**1**	Rh@NHC^e^	**1**	20	23	17	12	7	7
6^19^	**2**	Ru@PVP^d^	**2**	0	0	0	1	12	87
7	**2**	Ru@NHC^e^	**2**	0	0	0	0	11	89
8	**2**	Rh@NHC^e^	**2**	22	21	10	6	3	7

a Percentage of Products After 48 h.

b Conditions: NPs, solvent = THF. D_2_ pressure = 2 bar (D/H ratio = ca. 5). *T* = 55 °C. *t* = 48 h.

c NMR yield of deuterated products.

d 3 mol % cat.

e 5 mol % cat.

f 80 °C.

Deuteration in ortho positions of the aromatic ring took place as previously observed with **Ru@PVP** (Table 1, entries 1 and 4). However, a higher loading of **Ru@NHC** was required to achieve a similar conversion to that achieved with **Ru@PVP** at 80 °C (Table 1, entries 2 and 4). The **Rh@PVP** catalyst showed low solubility under the working conditions, resulting in very low reactivity and was therefore discarded (Table 1, entry 3; see also Figure 2). H/D exchange with **Rh@NHC** was slower than with **Ru@NHC** since mono-, di-, and trideuterated products (**1a**–**c**) were the main products after 48 h of reaction (Table 1, entries 4 and 5). Long reaction times were required in all cases since up to six consecutive reactions must take place to achieve the final product. Moreover, the H/D exchange of the last protons must be statistically less favorable.

H/D exchange in P(*p*-tolyl)_3_ (**2**) in the presence of **Ru@NHC** afforded full conversion and 89% selectivity toward the ortho-deuterated product **2f**, similarly to the results obtained with **Ru@PVP** (Table 1, entries 6 and 7). However, higher catalyst loadings of **Ru@NHC** were required. When the reaction was performed with **Rh@NHC** as the catalyst, 69% conversion into a mixture of deuterated compounds was obtained, where the fully ortho-deuterated compound **2f** was present in only a 7% yield (Table 1, entry 8). In all cases, no exchange in the methyl groups was detected.

**Scheme 2:**
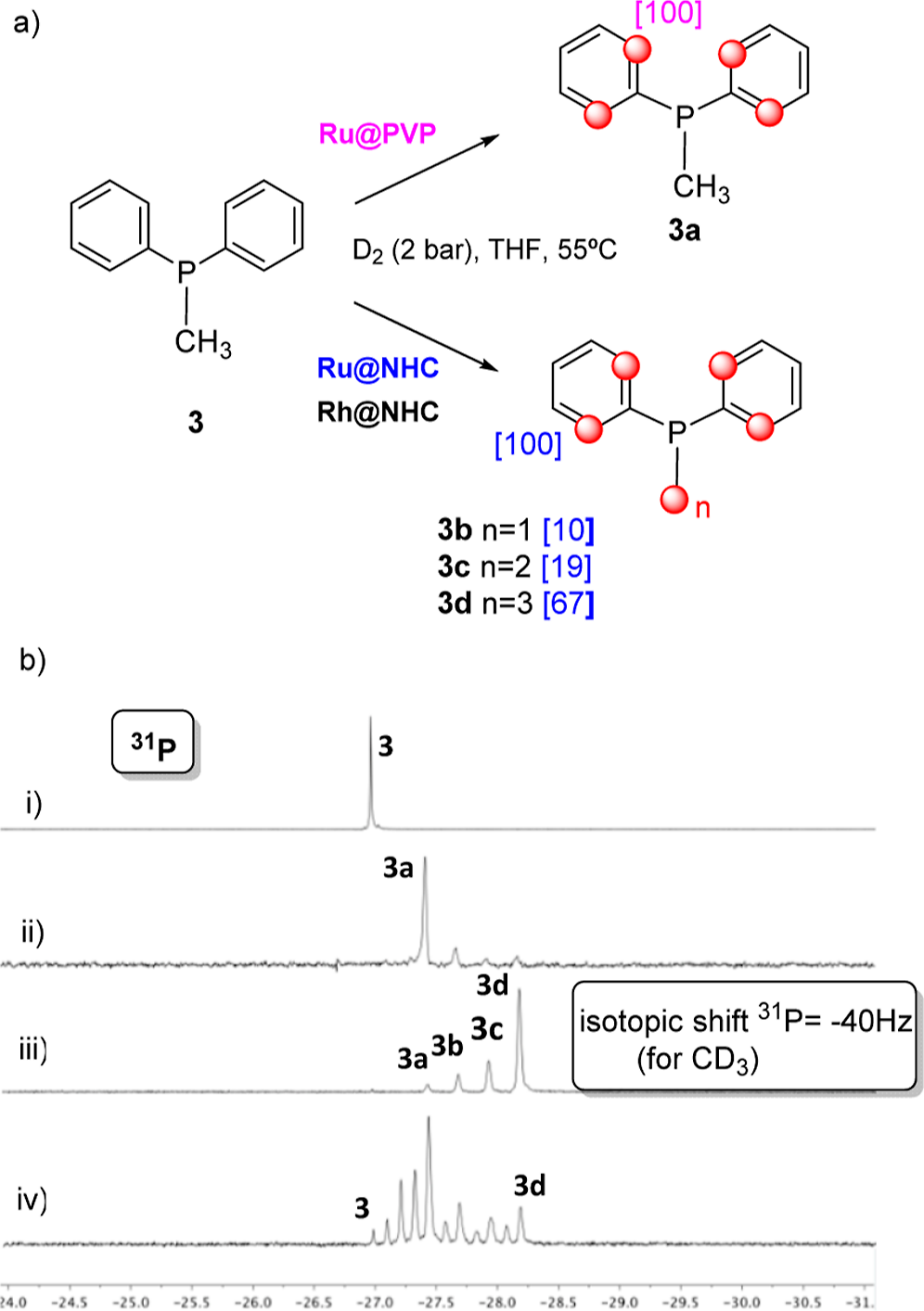
(a) H/D Exchange Reaction of **3** Using Ru@PVP, Ru@NHC, and Rh@NHC Nanocatalysts; (b) ^31^P{^1^H} NMR Spectrum of **3** (i), and of the Reaction Mixture Resulting From the H/D Exchange at 55 °C for 8 days Using (ii) Ru@PVP, (iii) Ru@NHCm and (iv) Rh@NHC

**Scheme 3:**
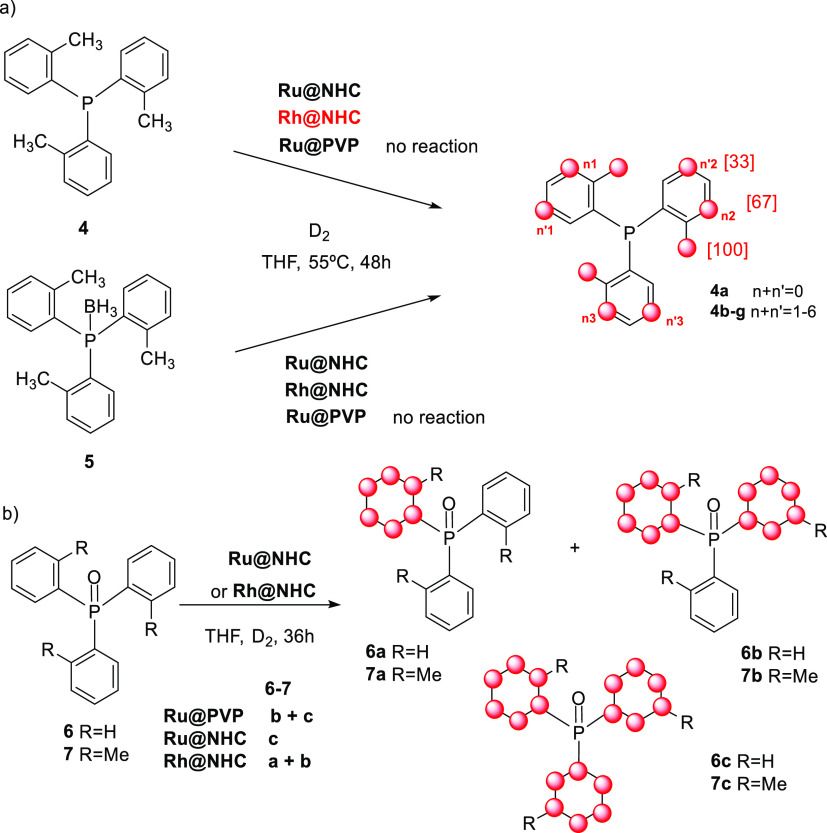
H/D Exchange of Substrates **4–7** in the Presence of Ru and Rh Nanocatalysts

When the reaction was carried out using PPh_2_Me (**3**) using **Ru@NHC** as the catalyst, compounds **3b–d** (Scheme 2a) were formed (ratio 10:19:67). ^2^H NMR indicated that these products were fully deuterated at ortho positions of phenyl rings, while the methyl group was partially deuterated [see ^31^P spectra, Scheme 2b(iii)]. In contrast, the reaction using **Ru@PVP** as the catalyst afforded almost exclusively compound **3a** [Scheme 3b(ii)], and only traces of deuteration at the methyl group were detected.

When H/D exchange was catalyzed by **Rh@NHC**, the reaction was slower than with its Ru analogue, and a complex mixture of partially deuterated products at the aromatic ring and methyl group was observed after 8 days at 55 °C [Scheme 2b(iv)]. Signals at lower chemical shifts correspond to compounds partially deuterated in both the aromatic ring and CH_3_. The different isotopic effect, approximately half for aromatic D than for CD_3_, produces this complex set of signals.

These results indicated that both NHC-stabilized catalysts **Rh@NHC** and **Ru@NHC** promote the H/D exchange of the ortho positions and the P–CH_3_ group of phosphine **3**, while **Ru@PVP** only provided H/D exchange for aromatic protons. These results illustrate well the influence of the stabilizer on the activity/selectivity of the reaction.

P(*o*-tolyl)_3_ (**4**) was then tested as the substrate. This compound presents a larger cone angle (194°)^24^ than PPh_3_ (145°), which was expected to affect its coordination to the NP surface and, consequently, to the deuteration patterns. When **4** was treated under the standard reaction conditions with **Ru@NHC** as the catalyst, the methyl groups and, surprisingly, the meta positions of the aryl rings were deuterated. A higher degree of H/D exchange was observed in position 3 than in position 5 (Scheme 3a) (see Figures S42–S47 in the Supporting Information). Interestingly, using the **Ru@PVP** catalyst, only traces of deuterated products were detected by ^31^P and ^2^H NMR.

Both **Rh@NHC** and **Ru@NHC** afforded similar results, but in this case, the reaction was faster using the Rh catalyst, and the CH_3_ was almost completely deuterated under the reaction conditions. The reaction was monitored by ^31^P NMR recording spectra from 7 h to 12 days. The ^31^P{^1^H} NMR spectra of the reaction mixture catalyzed by **Ru@NHC** showed a complex set of signals, which can be explained by the simultaneous H/D exchange in 15 different protons, 9 from methyl groups and 6 from the meta position of the aromatic rings (Figure S44, Supporting Information). Interestingly, and taking as reference the signal of phosphine **4**, a difference in the sign of the isotopic shift was evidenced depending on the deuterated position.

Indeed, when the H/D exchange took place at the CH_3_ groups, an isotopic shift of +2.5 Hz was detected, giving rise to the observation of nine different products at higher chemical shifts comparing to that with **4**. The observation of new signals at lower chemical shifts with an isotopic shift of −14.5 Hz was attributed to the H/D exchange at meta positions of the arene rings.

The ^31^P{^1^H} NMR spectra of the reaction mixture catalyzed by **Ru@NHC** showed almost complete deuteration of CH_3_ after 2 days, which simplified the ^31^P NMR spectra that essentially contained four sets of signals (Figure S52, Supporting Information). The intensity of signals at higher fields increased notably at long reaction times, which was attributed to the progressive deuteration of the meta positions of the aromatic rings after the complete deuteration of the methyl groups. The broadness of these ^31^P signals may indicate a slightly different isotopic shift depending on the meta position (3 or 5) where the deuteration takes place.

After reaction completion, the selectivity obtained in the H/D exchange in **4** with **Ru@NHC** and with **Rh@NHC** was similar. However, **Ru@NHC** deuterates the aromatic rings faster than the methyl group, while **Rh@NHC** deuterates the methyl group faster than the aromatic rings. Indeed, after 48 h, the ratio *D*_aromatic_/*D*_aliphatic_ = 47:53 was observed by ^2^H NMR for **Ru@NHC**, while for **Rh@NHC**, the ratio *D*_aromatic_/*D*_aliphatic_ = 17:83. The faster H/D exchange of aromatic protons with Ru NPs than with Rh NPs agrees with the results obtained with substrates **1** and **2**.

The ^2^H NMR spectrum registered after 12 days of reaction performed with **Rh@NHC** showed approximately 66% of H/D exchange for H-3 protons and 33% for H-5 (see Figure S43, Supporting Information), which confirmed the presence of **4d** (*n*1 + *n*2 + *n*3 = 2, *n*′1 + *n*′2 + *n*′3 = 1) as the main product (Scheme 3). These results agreed with the main signal detected by mass spectrometry at 316.3 (**4**, M^+^ + 12) (see Figure S47, Supporting Information).

To obtain further insights, the H/D exchange reactions of H_3_B–P(*o*-tolyl)_3_ (**5**) and O=P(*o*-tolyl)_3_ (**7**) were also studied. However, deprotection of **5** was observed during the reaction, and consequently, the distribution of deuterated products was the same as that obtained from phosphine **4** (Scheme 3a). When phosphine oxide **7** was treated under the standard reaction conditions in the presence of **Ru@NHC**, product **7c** resulting from the reduction of the aromatic rings was obtained (Scheme 3b). The use of **Rh@NHC** also produced the aromatic ring reduction, but it was less active, and a mixture of the partially reduced products **7a,b** was obtained. In this case, deuteration of ortho positions of the remaining aromatic rings was also observed. The behavior is similar to that observed for the reaction of **6** with **Ru@PVP**.^19^ In the case of *ortho*-tolylphosphine oxide (**7**), a partial deuteration of the methyl groups was also observed (Figures S58–S68, Supporting Information).

**Figure 3:**
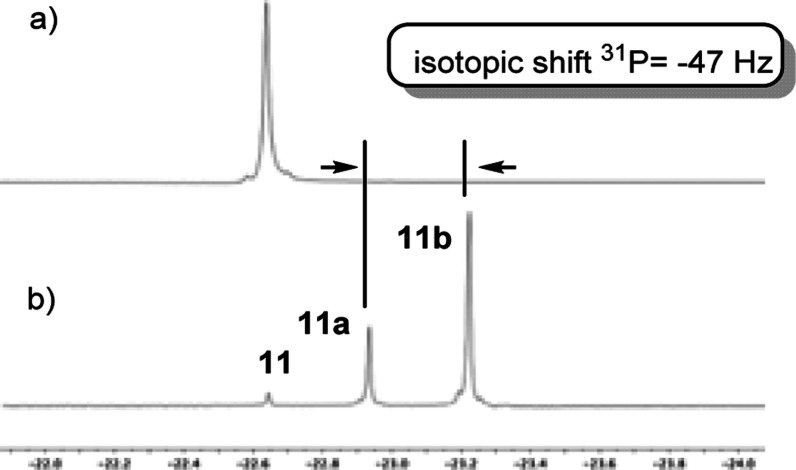
^31^P{^1^H} NMR spectra of (a) **11** and (b) deuteration reaction mixture of **11** after 48 h at 55 °C using Rh@NHC as the catalyst.

**Scheme 4:**
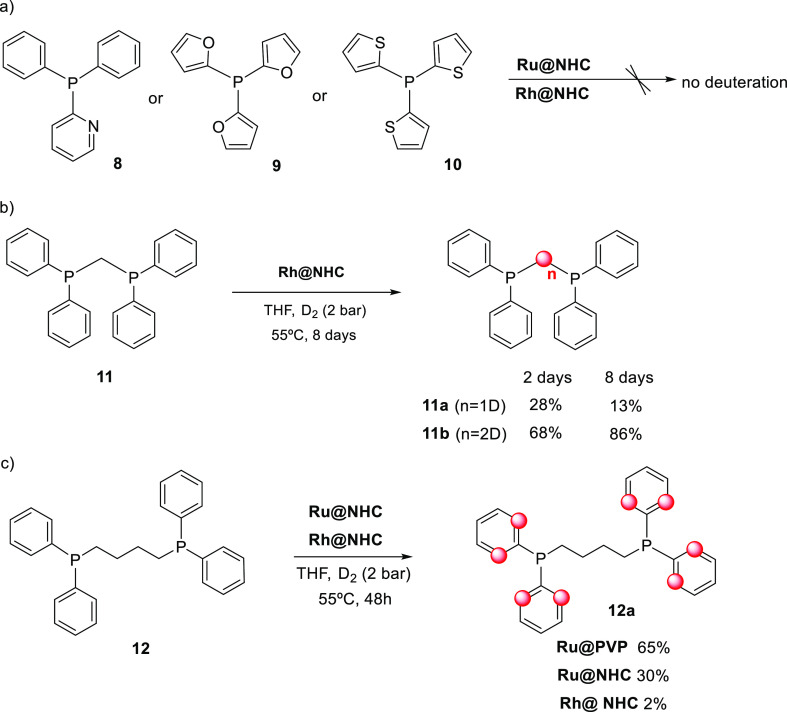
H/D Exchange of Chelating Monophosphines **8–10** and Diphosphines **11** and **12**

The monophosphines containing heteroaromatic rings diphenylpyridyl phosphine (**8**), tris-(2-furyl)phosphine (**9**), and tris-(2-thiophenyl)phosphine (**10**) were also tested as substrates. However, no deuteration was observed (Scheme 4). The same result was observed when diphenylphosphinomethane (dppm) (**11**) was treated under D_2_ pressure (2 bar) at 55 °C using **Ru@NHC** as catalysts even after 8 days of reaction (Scheme 4). However, when the **Rh@NHC** catalyst was used, selective H/D exchange at the methylene carbon was observed and the mono- and di-deuterium labeled products **11a** and **11b** (Scheme 4) were detected by ^31^P NMR (See Figure 3). In this case, the isotopic shift was −47 Hz, a value similar to that previously described in the labeling of compound **3** (ca. 40 Hz). Significant labeling was observed after 24 h, which achieved 86% of the dideuterated compound in 8 days (Scheme 4b).

To investigate the potential role of uncoordinated NHC in the reaction, **11** was treated in the absence of a metal catalyst but in the presence of NHC (IPr) under the same reaction conditions. However, after 48 h, no reaction was observed. The H/D exchange reaction in **11** was also attempted in the presence of 5 mol % of **Rh**/**C** under similar reaction conditions to those previously tested, but only traces of deuteration of THF used as reaction media and small amounts of D_2_O coming from the **Rh**/**C** were detected by ^2^H NMR.

The influence of the bridge length between the P atoms was investigated and the diphenylphosphinobutane (dppb) ligand (**12**) (Scheme 4c) was tested as the substrate. In this case, no deuteration of the alkyl chain was observed, and product **12a** resulting from the exclusive deuteration at the ortho positions of the aromatic rings was obtained when **Rh@NHC** and **Ru@NHC** were used as catalysts. Selectivity with **Ru@PVP** was also similar^19^ but much more efficient. Thus, while labeling using **Ru@PVP** yielded 65% of the fully deuterated compound **12a**, 30% yield was obtained using **Rh@NHC**. These results suggest a similar behavior to that observed for the monophosphines **1** and **2**.

Overall, these results show that Ru and Rh NPs stabilized with NHC (IPr)^16b^ and PVP catalyze the selective H/D exchange of a variety of aryl and alkyl-aryl monophosphines and diphosphines under low D_2_ pressure. Activity depends on the metal and the stabilizer. H/D exchange in aromatic rings was faster using **Ru@NHC** as catalysts than with **Rh@NHC**. However, the opposite trend was observed in the H/D exchange of alkyl chains, particularly when they were linked to an aromatic ring, as in the case of phosphine **4**. Concerning the stabilizer effect, H/D exchange of aromatic rings is faster with **Ru@PVP** than with **Ru@NHC** (case of phosphines **1**, **2**, **3**, and **12**), but H/D exchange in methyl groups of **4** only takes place in the presence of **Ru@NHC** and **Rh@NHC** and not in the presence of **Ru@PVP**. The higher reactivity of **Ru@PVP** when compared to that of **Ru@NHC** can be justified by the higher surface availability in the polymer-stabilized catalyst.

For explaining the lack of conversion in phosphines **8-10**, more complex factors must be taken into consideration. H/D exchange catalysis depends on several parameters including not only the ability to break C–H bonds but also the residence time of the substrate on the catalyst. According to the Sabatier principle, the substrate must stay long enough in contact with the metal to allow catalysis to proceed, but a too strong coordination of the substrate would hamper any catalysis. The chelating monophosphines **8–10** presumably coordinate strongly to the metal NPs through the phosphorus and the heteroatom simultaneously, hence preventing catalytic H/D exchange. For the related phosphine dppm (**11**), a strong coordination was also expected, but deuteration of the methylene bridge was observed with **Rh@NHC**. The chelate coordination of **11** could place the methylene bridge close to the metal surface, while preventing the interaction of the aromatic rings.

### Computational Study on the Origin of Selectivity

To shed light on the mechanism of deuterium exchange and on the origin of the observed selectivity in the H/D exchange, DFT calculations were performed.^25^ In general, Ru and Rh NPs show similar selectivity for all phosphines, although for dppm, the isotope exchange is not observed for Ru NPs due to their low reactivity toward the aliphatic C–H bonds. Here, we have selected Rh NPs, using a 55-atom icosahedron (ICO) structure with a diameter of 1.29 nm. The size and the shape of the cluster model are consistent with the experimental observations in which all NPs exhibited small sizes (<2 nm) and spherical shapes.^18b,19,21^

**Figure 4:**
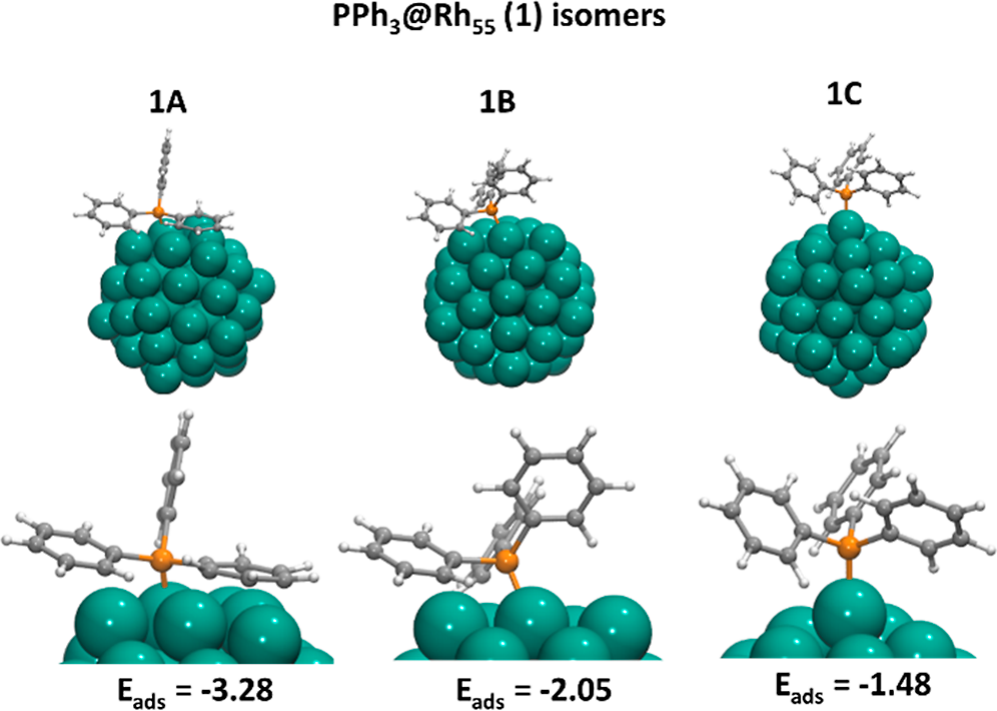
Geometries and adsorption energies (eV) of different coordination modes of PPh_3_ (**1**) onto the icosahedral Rh_55_ cluster.

Moreover, the icosahedral structure is 1.8 eV lower in energy than other symmetric, spherical-type cuboctahedron structure and constitutes a simple model with only one type of face, (111), and two possible adsorption sites: vertex and edge (see Figure 4). Initially, we based our study on naked models to reduce complexity and provide a fundamental understanding of the interaction of phosphines with different structural motifs of the NP.

Then, we compared key reaction energy profiles with more realistic models including hydrides and NHC stabilizers on the metal surface. Phosphines **1**, **2**, **3**, and **12** show a similar behavior in selectivity and activation of ortho positions, which is very different from that of phosphine **4** that undergoes activation at two different meta-aromatic C–H sites and at aliphatic C–H bonds. In this context, we selected phosphines **1** and **4** as representative ligands.

### Origin of Selectivity toward Aromatic *ortho* C–H Bonds in PPh_3_

Figure 4 shows three representative coordination modes and the corresponding adsorption energies of PPh_3_ phosphine (**1**) onto the Rh_55_ icosahedral NP, highlighting the specific interactions with the C–H *ortho* bonds for which deuteration is observed. In all cases, phosphine adsorbs in a top mode through the coordination of the phosphorus lone pair to the rhodium atom as reported for other computational studies for different metal surfaces.^26−28^

In the most stable structure (**1A** in Figure 4), phosphine coordinates through the phosphorus lone pair to the edge Rh atom, bending toward one of the NP faces, thus allowing the interaction of two phenyl substituents.

One of the phenyl groups interacts with a neighboring (111) facet through the six carbon atoms of the π system. The η^6^-phenyl interaction is characterized by short Rh–C distances (2.18 Å in av.) and some pyramidalization of the carbon atoms that breaks the planarity of the aromatic ring, lifting the hydrogen atoms out of the ring plane. The other phenyl group interacts with a vertex Rh atom, displaying an η^2^-phenyl interaction with the ipso and ortho carbon atoms at distances of 2.34 and 2.35 Å, respectively (structure **1A** in Figure 4).

**Figure 5:**
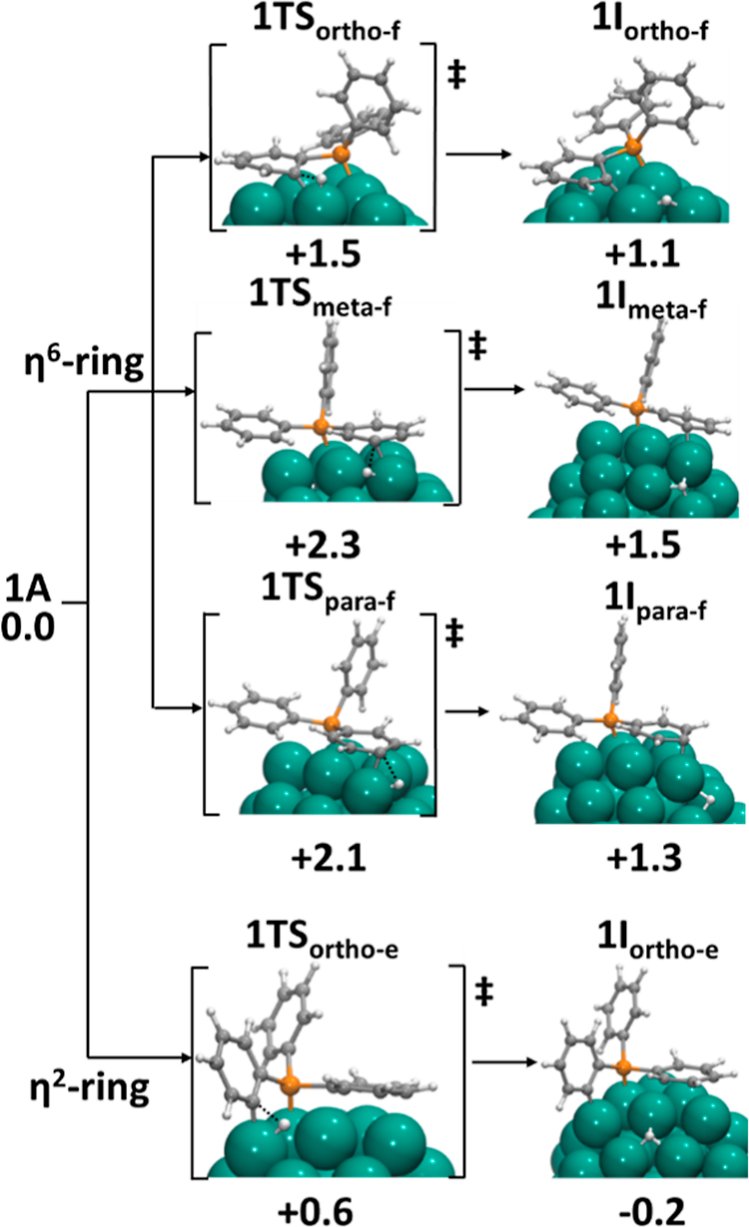
Possible C–H activation paths of PPh_3_ (**1**) adsorbed on Rh_55_: *ortho*-(**1TS**_**ortho-f**_), *meta*-(**1TS**_**meta-f**_), and *para*-face (**1TS**_**para-f**_) and *ortho*-edge (**1TS**_**ortho-e**_). Energy barriers and relative energies in eV.

These two types of phenyl interactions with the Rh NP in an adsorbed phosphine indicate that there are two possible paths for C–H activation, the face activation and the edge activation. Figure 5 shows the computed transition states and the resulting intermediates for all the possible pathways for C–H activation of PPh_3_ phosphine on Rh_55_ from structure **1A**. For the phenyl group interacting in the η^6^-mode, the computed energy barriers are relatively high for all the possible activations: 1.5, 2.3, and 2.1 eV for *ortho*, *meta*, and *para* C–H bond types, respectively.

In these transition states, as the bond breaks, the carbon atom has to lift out of the ring plane to optimize its interaction with the Rh atom (see Figure 5). This results in a large geometric distortion of the phenyl ring that loses resonance, and consequently, the energy penalty results in a prohibitively high energy barriers and high-energy laying intermediates.

For instance, the C_ortho_–C_ipso_–C_ortho_–C_meta_ torsion angle in **1TS**_**ortho-f**_ and **1I**_**ortho-f**_ was around 10°, while in **1A**, it was 4°, or in the case of meta-activation, the C_meta_–C_ortho_–C_ipso_–P dihedral was decreased from 172° in **1A** to 165° or 162° in **1TS**_**meta-f**_ and **1I**_**meta-f**_, respectively. The distortion in para-activation is less pronounced, as reflected in the change in the P–C_ipso_–C_ortho_–C_para_ angle from 167° in **1A** to 165° in **1TS**_**para-f**_ and **1I**_**para-f**_. It is likely that this description is a good approximation of the behavior of aromatic compounds over larger (111) surfaces in extended models,^29,30^ for which C–H activations were not observed to the best of our knowledge.^31^ On the other hand, the *ortho* C–H bond interacting with the edge of the NP can be activated with a moderate energy barrier (+0.6 eV), while the phenyl ring is not significantly distorted along the process (see structures 1**TS**_**ortho-e**_ and **1I**_**ortho-e**_ in Figure 5). In fact, the resulting intermediate could be described as an ortho-metalated phosphine dirhodium fragment, which is a well-known complex type in coordination chemistry. Moreover, the resulting intermediate has a similar energy to that of the reactants, and so, the H* atom can then easily exchange its position with deuterides (D*) and can return to the adsorbed PPh_3_ phosphine. The other *ortho* C–H bonds become accessible for activation through rotations of the phenyl rings and bending of the phosphorus atom. It is also worth noting that similar energy schemes were reported in computational studies of H/D exchange on N-containing substrates.^13,14,32^ Looking at the results obtained for phosphine oxides **6** and **7**, one can envisage a different mechanism for H/D exchange, in which D* species present at the surface reduce the phenyl ring, the phenyl rotates, and then, the H of the reduced carbon moves to the Rh surface. Nevertheless, the computed barrier for the reduction of the ortho position by one hydrogen at the surface (1.1 eV) is significantly larger than for the corresponding C–H activation (see Figure S111), and therefore, this associative mechanism can be discarded for phosphine **1**. Overall, we can conclude that for PPh_3_ phosphines, the H/D exchange occurs through C–H activation at NP edges where activation of *ortho* C–H bonds does not involve a significant distortion of the phenyl ring. In contrast, the interaction of *meta* and *para* C–H bonds with the Rh surface involve phenyl arrangements that are not suitable for smooth activations at the aromatic ring.

### Origin of Selectivity toward Aliphatic and Aromatic *meta* C–H Bonds in P(*o*-tolyl)_3_

**Figure 6:**
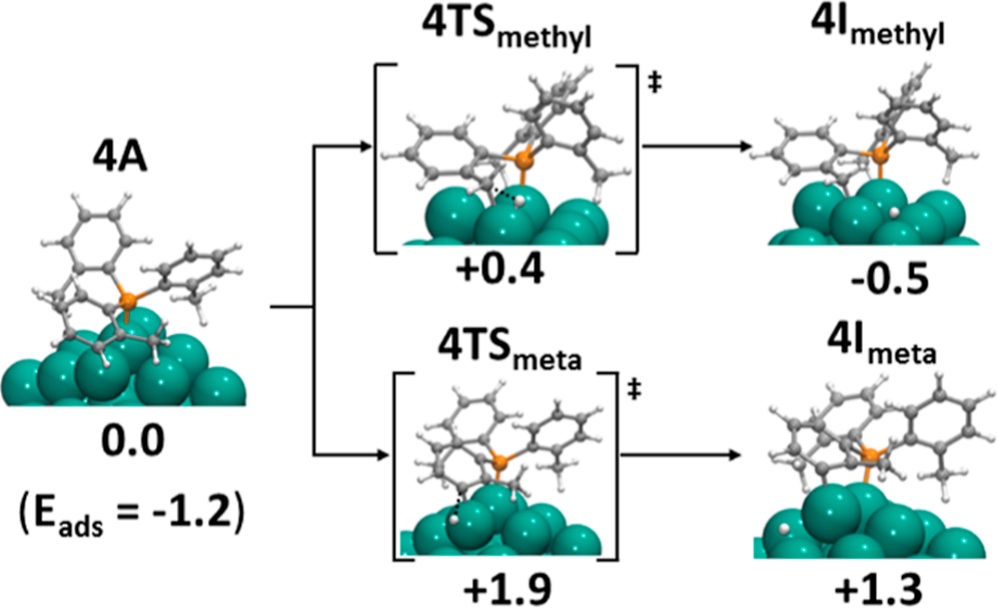
Possible C–H activation paths of P(*o*-tolyl)_3_ (**4**) on Rh_55_: methyl C–H activation (**4TS**_**methyl**_) and aromatic C–H meta-activation on the Rh edge (**4TS**_**meta**_). Energy barriers and relative energies in eV.

Next, we turned our attention to P(*o*-tolyl)_3_ phosphine (**4**), in which H/D exchange is observed at aliphatic C–H bonds and at a different position of the aromatic ring (meta) compared with that in phosphine **1**. Using as reference the structures obtained for the adsorption of **1**, we tried to build analogous interactions between Rh_55_ and phosphine **4** (Figure 6). However, the adsorption energy for **4** (+1.2 eV) is significantly lower than for **1** because the presence of a methyl group prevents η^6^-interactions of the aromatic rings. As shown in Figure 6, **4** adsorbs onto the Rh surface through coordination of the phosphorus lone pair on an edge rhodium atom and through one of the tolyl substituents where the methyl group and the aromatic *meta* carbon are directly interacting with the (111) facet and the vertex Rh atom (structure **4A**). Figure 6 also illustrates the possible pathways for C–H bond activation from P-adsorbed phosphine.

The computed energy barriers are 0.4 and 1.9 eV for aliphatic and aromatic *meta* C–H activations, respectively. This agrees with the relative rates of aliphatic and aromatic carbons observed experimentally, but the barrier of the aromatic C–H activation is too high to be feasible at current experimental conditions. Alternatively, the *meta* C–H bond could be activated through a sequential mechanism. After the initial activation of the methyl group, the resulting intermediate could undergo a second C–H activation at the meta position, but the computed energy barrier for the second step is even higher (2.3 eV).

**Figure 7:**
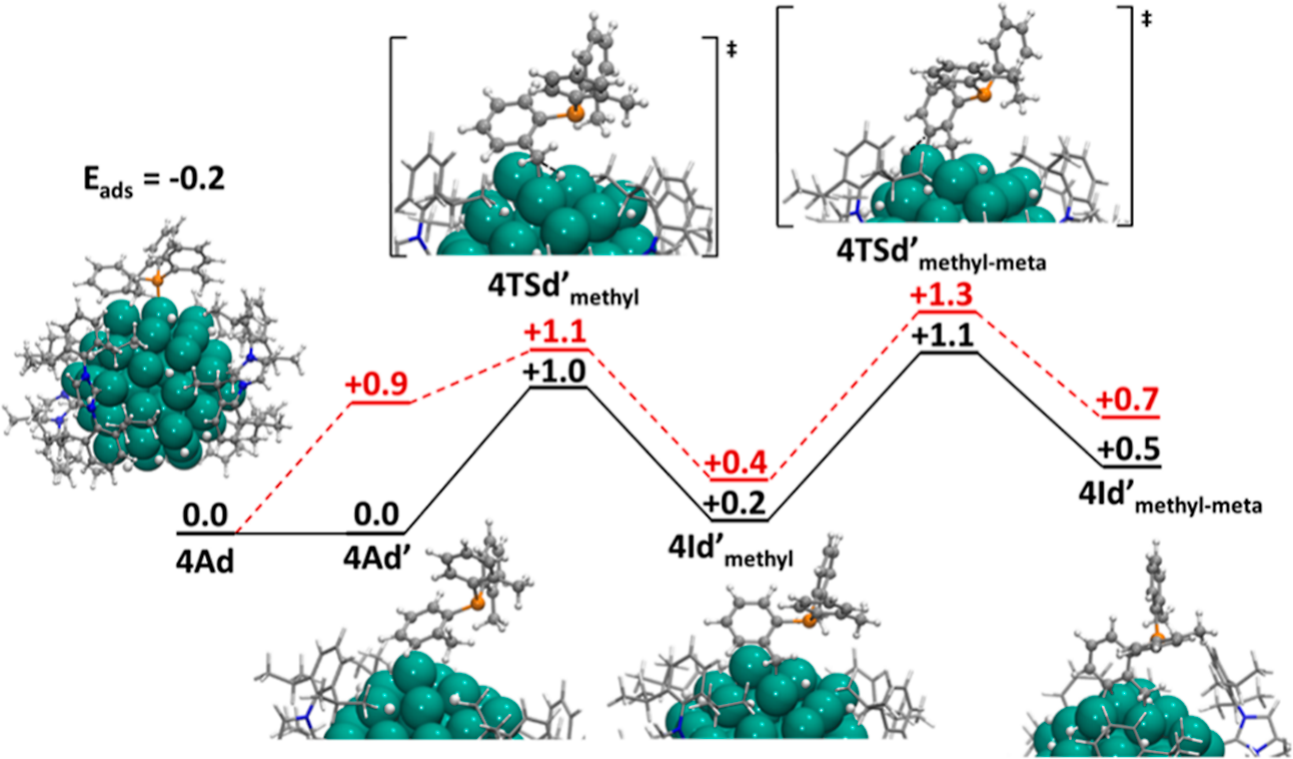
Potential energy profiles (eV) for the selective H/D exchange of P(*o*-tolyl)_3_ phosphine (**4**) on the decorated Rh_55_ NP (solid black lines) through the dissociative mechanism. For comparison, values on the naked NP are reported (dashed red lines).

To explain the selectivity observed for phosphine **4**, we propose a dissociative mechanism and consider the effect of the ligands decorating the rhodium NP. Figure 7 compares the reaction energy profiles for the two C–H activations on naked NPs (red dashed lines) and on a decorated NP with 0.6 H atoms and 0.1 NHC ligands per Rh surface atom (black solid lines). This dissociative mechanism can be divided into three main steps: (1) the phosphine first detaches the phosphorus atom from the Rh surface, resulting in a weakly phosphine-bonded intermediate in which the phosphine interacts only through the methyl group and the *meta* and *para* carbon atoms of the aromatic ring (species **4Ad′**); (2) from this adduct, the activation of the C–H bonds of methyl groups occurs, yielding alkyl-rhodium intermediates with the *meta* carbon interacting with one rhodium in the vertex; and (3) the *meta* C–H bond is activated, resulting in a five-membered dirhodium metallacycle intermediate.

Initially, we evaluated the effect on phosphine **4** adsorption of the hydrides and NHC stabilizers on the Rh surface (**4Ad** in Figure 7). In the selected model, the adsorption energy for **4** is only −0.2 eV, significantly lower than that computed on the naked NP (−1.2 eV). This can be attributed to two factors: (1) the electronic stabilization of the Rh_55_ cluster by the ligands (the d band center shifts from −1.95 to −2.17 eV) reducing the Rh–P interaction and (2) the large cone angle of phosphine **4** causing steric repulsions between phosphine substituents and other species at the Rh surface. Consequently, the first step of the proposed mechanism involving phosphorus decoordination (from **4Ad** to **4Ad′**, see Figure 7) is isoenergetic, indicating that the interaction of phosphine **4** with the decorated NP is weak and can occur either through the phosphorus lone pair or through the tolyl substituents. From adduct **4Ad′**, the activation of the methyl C–H bond has a moderate energy barrier (1.0 eV) and forms the intermediate **4Id′**_**methyl**_ (Figure 7). Then, the *meta* C–H bond interacting with a Rh atom in the vertex can be activated, overcoming an energy barrier of 0.9 eV. The resulting C–H diactivated intermediate **4Id′**_**methyl-*meta***_**′** lies 0.5 eV above the P-coordinated phosphine adduct **4Ad** and can incorporate deuterium from the metal surface. Compared to that for naked NPs, the energy profile for decorated NPs is shifted down in energy (0.2 eV approximately), leading to an overall energy barrier for the double C–H activation of 1.1 eV, which is accessible at working conditions. Moreover, the reduction of the energy barrier when introducing the effect of NHC ligands could explain why phosphine **4** is deuterated with **Rh@NHC** and **Ru@NHC** NPs, but the reaction is not observed for **Ru@PVP** systems where the NP surface is more available.

Finally, we also investigated the observed activation of the C–H bonds in the 5-*meta* position of the tolyl substituent using the simpler naked Rh_55_ cluster model (see Figure S112). Starting with phosphine **4** adsorbed through the 5-meta and para positions of the tolyl substituent on a vertex Rh atom, the computed energy barrier for the corresponding C–H activation is 0.9 eV. This barrier is larger than that for methyl C–H activation computed from **4A** (0.4 eV, Figure 6), in agreement with the experimental observations, in which the methyl and the 3-meta position are deuterated faster than the 5-meta position. Although the access to accurate energy barriers is difficult because of the structural complexity of the organometallic NP, we succeeded in reproducing relative rates of C–H activations and in explaining the selectivities observed for several phosphines.

## Conclusions

We have shown that the Ru and Rh NPs stabilized by bulky NHC ligands can perform deuterium exchange reactions. In general, the activity of the reaction depends on the nature of the metal and on the stabilizer, whereas the selectivity is mainly related to the phosphine structure.

Concerning the activity, the following general trends among the studied systems were observed: (a) Ru NPs are more active than Rh NPs for the H/D exchange of aromatic protons. (b) Rh NPs are more active than Ru NPs for the H/D exchange of alkyl chains, particularly when they were linked to an aromatic ring, as in the case of phosphine **4**. It is however not so clear when the alkyl groups are directly bonded to phosphorus, as is the case of phosphine **3**. c) The higher reactivity of **Ru@PVP** when compared to that of **Ru@NHC** can be justified by the higher surface availability in **Ru@PVP**.

**Figure 8:**
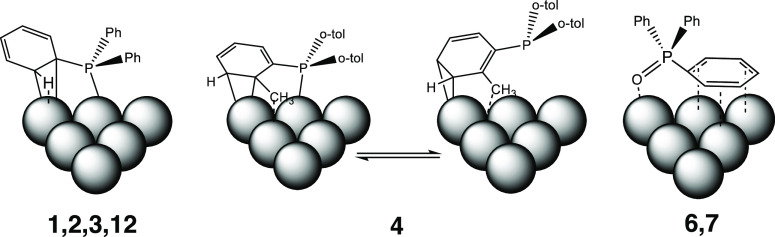
Proposed coordination modes of phosphines **1**–**4**, **6**, **7**, and **12** to the NP surface.

The combination of experimental observations and atomistic simulations has unraveled some crucial factors affecting the selectivity on H/D exchange. DFT comparison of representative phosphines **1** (PPh_3_) and **4** (P(*o*-tolyl)_3_) shows that selectivity depends on the interaction modes with the NP surface (see Figure 8). Phosphine **1** adsorbs to the Rh NP through P lone pair electrons, while the phenyl substituents can simultaneously interact with the Rh surface. The η^2^ interaction mode through *ortho* and *ipso* C atoms with the NP edge leads to a moderate energy barrier for *ortho* C–H activation and to an unstressed five membered dimetallacycle. This indicates that isotope exchange via C–H activation is favored on the edge atoms of the NPs.

P(*o*-tolyl)_3_ phosphine **4** adsorbs also through P, but the adsorption energy is lower than that for **1** because the presence of methyl substituents prevents the π-interaction of aromatic rings with the NP. When NHC ligands are present on the NP surface, the P-coordination becomes isoenergetic with adducts interacting weakly through the phosphine substituents. From these adducts, a multistep C–H activation mechanism is proposed: (1) the C–H methyl bonds are activated at one NP edge and (2) the *meta* C–H bond of the aromatic ring is subsequently activated, yielding an accessible five-membered dirhodium metallacycle intermediate. This would explain why the activation is observed for **Rh@NHC** and **Ru@NHC** NPs but not for **Ru@PVP** in which the surface is not decorated with ligands.

Results with phosphines **2**, **3**, and **12** (Figure 8), similarly to those with **1**, are compatible with a strong coordination to the NPs through the P atom, which results in the preferred deuteration at ortho positions of aromatic rings and eventually at the methyl group in **3**.

Small natural bite angle bidentate ligands such as pyridyl, furyl, and thiophenyl diphenylphosphines (**8–10**) are not deuterated under the tested conditions, probably due to their strong coordination to the surface that prevents the interaction of C–H bonds with the NP surface and/or the formation of metallacycles. This trend is also observed for the dppm (**11**) substrate using Ru nanocatalysts; however, in the presence of both Rh and NHC, the dppm ligand is exclusively deuterated in the methylene bridge between the two P atoms. Chelate coordination of **11** could place the methylene bridge close to the metal surface, while preventing the interaction of the aromatic rings. The case of phosphine oxides **6** and **7** is different since the aromatic ring is reduced. These results can be explained by the coordination of **6** and **7** to the NP surface via π-interactions of the phenyl rings with the surface, thus favoring their reduction.

Overall, the positions available for H/D exchange in a phosphine depends on its coordination mode to the NPs, enabling the establishment of structure–coordination–selectivity relationships. Moreover, aromatic sites can be selectively deuterated, or both aromatic and alkyl sites can be labeled by selecting the appropriate catalyst. These results also show the usefulness of the presence of a coordination sphere on decorated NPs, which can, as in molecular chemistry, orient a reaction and even allow otherwise impossible reactions.

## Supporting Information Available

Synthetic procedures; ^1^H, ^13^C, and ^2^D NMR spectra and MS spectra; details of quantum-chemical calculations of computed deuteration processes, including those calculated for NHC-decorated NPs; and DFT-optimized Cartesian coordinates (PDF)
