# Supplementary material for: Bringing Selectivity in H/D Exchange Reactions Catalyzed by Metal Nanoparticles through Modulation of the Metal and the Ligand Shell
Source: Inorg Chem. 2023 Mar 9;62(11):4570–80. doi: 10.1021/acs.inorgchem.2c04442 (PMC10031563; doi:10.1021/acs.inorgchem.2c04442)
Supplement: Supplementary file 1 — ic2c04442_si_001.pdf [file ic2c04442_si_001.pdf]

SUPPORTING INFORMATION

Bringing selectivity in H/D exchange reactions catalyzed by metal nanoparticles through modulation of the metal and the ligand shell

Francisco Martinez-Espinar, [a,b] Antoni Salom-Català,[a] Emma Bresó-Femenia,[b,c] Carmen Claver,[a] Francesca Baletto,[d] Josep M. Ricart,[a] Bruno Chaudret,[b] Jorge J. Carbó,[a]* Cyril Godard, [a]* and Sergio Castillón[c]*

[a]Departament de Química Física i Inorgànica, Universitat Rovira i Virgili, C/ Marcel.lí Domingo s/n, 43007, Tarragona, Spain.

E-mail: cyril.godard@urv.cat; j.carbo@urv.cat

[b]Laboratoire de Physique et Chimie des Nano Objets, LPCNO, UMR5215 INSA-UPS-CNRS, Université de Toulouse; Institut National des Sciences Appliquées, 135 avenue de Rangueil, 31077 Toulouse

[c]Departament de Química Analítica i Orgànica; Universitat Rovira i Virgili; C/ Marcel.lí Domingo s/n, 43007 Tarragona, Spain. E-mail: sergio.castillon@urv.cat

[d]Department of Physics, King's College London, London, Strand Building, Strand, WC2R 2LS, United Kingdom.

Table of contents:

1. General methods and characterization techniques S3

2. General procedure for H/D exchange reactions S4

3. Synthesis of substrates S4

4. NMR and MS spectra S5

4.1. H/D exchange of PPh_3 (**1**) S5

4.2. H/D exchange of P(*p*-tolyl)$_3$ (**2**) S13

4.3. H/D exchange of PPh_2Me (**3**) S19

4.4. H/D exchange of P(*o*-tolyl)$_3$ (**4**) S28

4.5. H/D exchange of H_3B-P(*o*-tolyl)$_3$ (**5**) S35

4.6. H/D exchange of O=PPh_3 (**6**) S36

4.7. H/D exchange of O=P(*o*-tolyl)$_3$ (**7**) S42

4.8. H/D exchange of dppm (**11**) S43

4.9. H/D exchange of dppb (**12**) S46

5. Quantification of isomers ratio in deuteration experiments by ^{31}P NMR S52

5.1. Deuteration of PPh_3 (**1**) S52

5.2. Deuteration of P(p-tolyl)$_3$ (**2**) S56

5.3. Deuteration of $PMePh_2$ (**3**) S58

5.4. Deuteration of P(o-tolyl)$_3$ (**4**) S60

5.5. Deuteration of diphenylphosphinomethane (**11**) S62

5.6. Deuteration of diphenylphosphinobutane (**12**) S63

6. Computational information

6.1 Computational details S65

6.2 Alternative associative mechanism for the H/D exchange on the ortho position of **1** (Figure S111) S65

6.3 Proposed associative mechanism for the H/D exchange of 5-meta and *para* aromatic positions of phosphine **4** (Figure S112) S66

7. DFT-optimized cartesian coordinates S67

8. References S172

1. General methods and characterization techniques

General methods. All operations were carried out using standard Schlenk tubes, Fischer-Porter bottle techniques or in a glove-box under argon atmosphere. The chemicals were purchased from Sigma-Aldrich and used without further purification. THF and pentane were dried over sodium/benzophenone, distilled and then thoroughly degassed before use by three freeze- pump cycles. Rh nanoparticles were synthesized following a reported methodology starting from the appropriate metal precursor and stored in a glove box under argon atmosphere. The synthesis of the nanoparticles and the catalysis was carried out in a Fischer-Porter glassware under argon following the procedure previously reported by our group.[1]

Solution Nuclear Magnetic Resonance (NMR). ^{1}H and ^{13}C, spectra were recorded on a Varian® Mercury VX 400 (400 MHz and 100.6 MHz respectively). Chemical shift values for ^{1}H and ^{13}C were referred to internal $SiMe_4$ (0.0ppm). Chemical shifts are reported in parts per million (ppm) and coupling constants are reported in Hertz (Hz). Mass spectra was recorded on a Finnigan MAT 900S (EB-Trap Geometry) Syringes pump Model 22.

Gas Chromatography – Mass Spectrometry (GC-MS). The structure of the different deuteration reaction products was determined by GC-FID on an Agilent Technologies 7890A spectrometer, with an achiral HP-5 column (30m x 0.25mm x 0.25μm). The method used consists in an initial isotherm period at 80°C for 2 min followed by a 15°C/min ramp to 280°C and a hold time of 20 min, with a flow of 1.3ml/min.

2. General procedure for H/D exchange reactions

A 100 ml Fischer-porter glassware was charged in a dry-box (nanoparticles were always manipulated in a dry box) with the desired **Ru@PVP** (3mol%), **Rh@PVP** (25mol%), **Ru@NHC** or **Rh@NHC** (25mol%) and a magnetic stirrer. The Fischer-Porter was left under vacuum for 5 minutes and then it was pressurized under 3 bar of D_2 gas during 2 hours. Next a solution of the substrate (0.15 mmol) in degassed THF (2 ml) was added under argon. The reaction was stirred under 2 bar of D_2 under the required temperatures and time. Then the solution was cooled down to room temperature, the solvent was removed, deuterated products were extracted using pentane and evaporated to dryness. In the case of compounds with low boiling points, direct NMR analysis was performed without isolation of the reaction products.

3. Synthesis of substrates

Synthesis of O=P(*o*-tolyl)$_3$ (**11**)[2]:

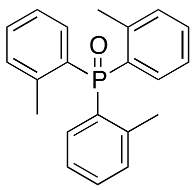

P(*o*-tolyl)$_3$ (200mg, 0.657 mmol) was dissolved in 9 ml of Toluene in an Schlenk flask and the solution was cooled to 0°C. Then, H_2O_2 (0.65ml, 6.57 mmol) were added dropwise with stirring. The reaction was stirred overnight at room temperature. Then, the toluene phase was transferred into a round bottom flask with a metal cannula, the aqueous phase was washed with toluene (3x2 ml) and all organic phases were combined. The resulting solution was stirred for 4h in the presence of molecular sieves. After retrieving the molecular sieves, the solvent was removed in vacuum to obtain the product **11** as a white crystalline solid. (Yield: 179 mg, 85%). **^{1}H-NMR** ($CDCl_3$, 400MHz, δ in ppm): δ = 7.42 (t, 3H, -$CH_{arom,meta3}$-, J= 7.46 Hz), 7.31 (dd, 3H, -$CH_{arom,meta5}$-, J= 7.01 Hz, J= 4.17 Hz), 7.16 (t, 3H, -$CH_{arom,ortho}$-, J= 6.59 Hz), 7.09 (dd, 3H, -$CH_{arom,para}$-, J= 11.5 Hz, J= 8.72 Hz), 2.49 (s, 9H, -CH_3). **^{31}P{^{1}H} NMR** ($CDCl_3$, 162MHz, δ in ppm): δ = 37.16.

Synthesis of H_3B-P(*o*-tolyl)$_3$ (**12**)[3]:

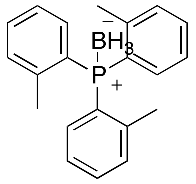

To a solution of P(*o*-tolyl)$_3$ (200mg, 0.657 mmol) in dried THF (2ml) a solution of BH_3·THF (1.65 ml, 1.6425 mmol) was added at -78°C with stirring. The reaction was stirred overnight at room temperature. All volatiles were removed in vacuum and the resulting residue was dissolved in CH_2Cl_2 with subsequent filtration over silica. The product **12** was obtained as a white solid after removing the solvent. (Yield: 173 mg, 83%). **^{1}H-NMR** ($CDCl_3$, 400MHz, δ in ppm): δ = 7.42 (t, 3H, -$CH_{arom,meta3}$-, J= 6.84 Hz), 7.33 (dd, 3H, -$CH_{arom,meta5}$-, J= 7.71 Hz, J= 4.13 Hz), 7.14 (t, 3H, -$CH_{arom,ortho}$-, J= 6.74 Hz), 6.98 (dd, 3H,

-CH$_{arom,para}$-, J= 11.3 Hz, J= 8.58 Hz), 2.41 (s, 9H, -CH$_3$). **^{31}P{^{1}H} NMR** (CDCl$_3$, 162MHz, δ in ppm): δ = 22.69. **^{11}B{^{1}H}-NMR** (C_6D_6, 128MHz, δ in ppm): δ = -31.56 (s).

4. NMR and MS spectra

<u>4.1. H/D exchange of PPh$_3$ (1):</u>

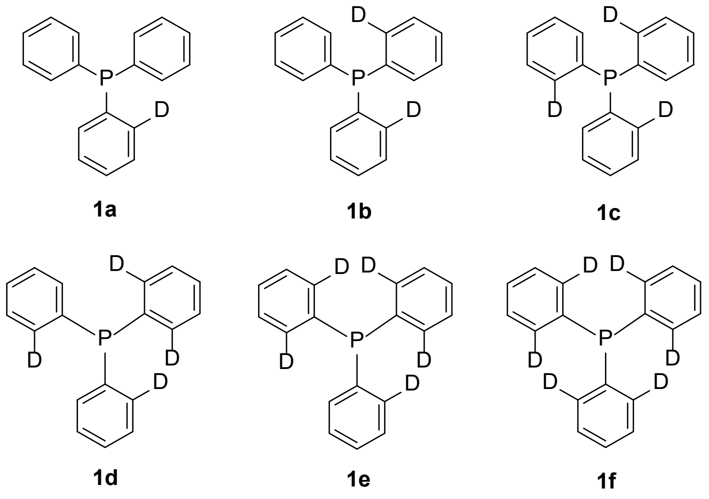

Fig. S1. Products **1a-f** detected during the deuteration of PPh$_3$ catalysed by Ru@PVP.

<u>4.1.1. Using **Ru@PVP**:</u>

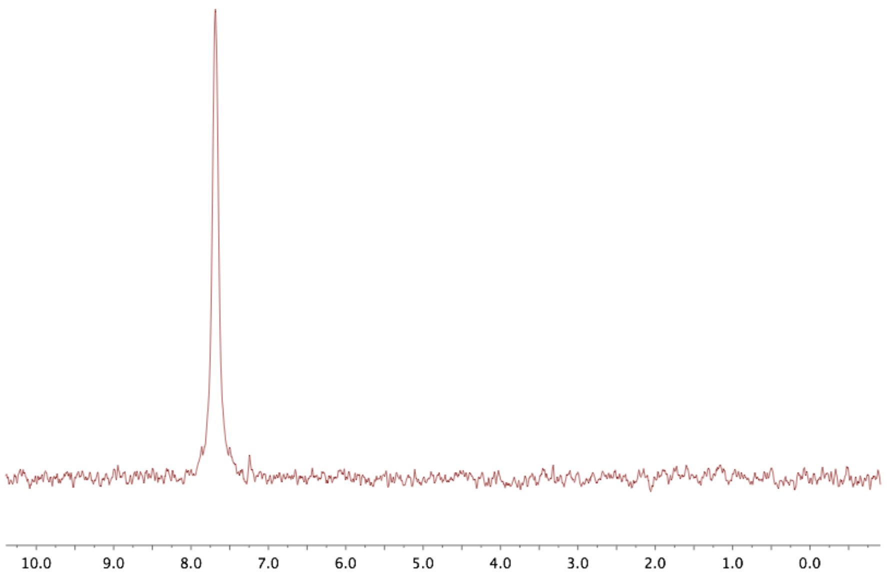

Figure S2. ^{2}D NMR (CHCl$_3$, 61.49MHz) spectrum of **1f** resulting from the deuteration of PPh$_3$ (**1**) using **Ru@PVP** after 48h at 55°C

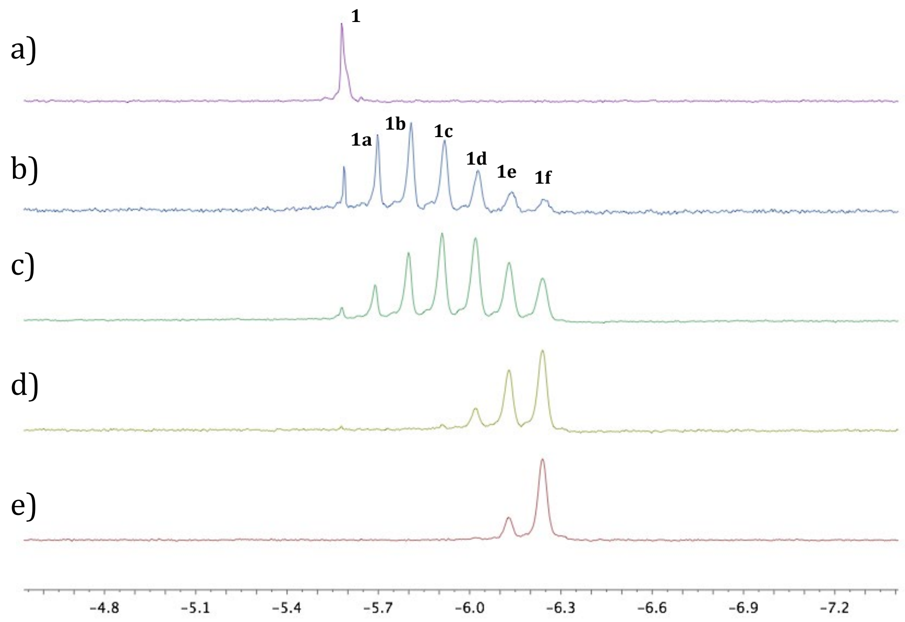

Figure S3. $^{31}P\{^{1}H\}$ NMR ($CDCl_3$, 162MHz) spectra of PPh_3 (**1**) deuteration evolution using **Ru@PVP**: (a) t=0, (b) 16h, (c) 36h, and (d) 48h at 55°C and (e) 48h at 80°C

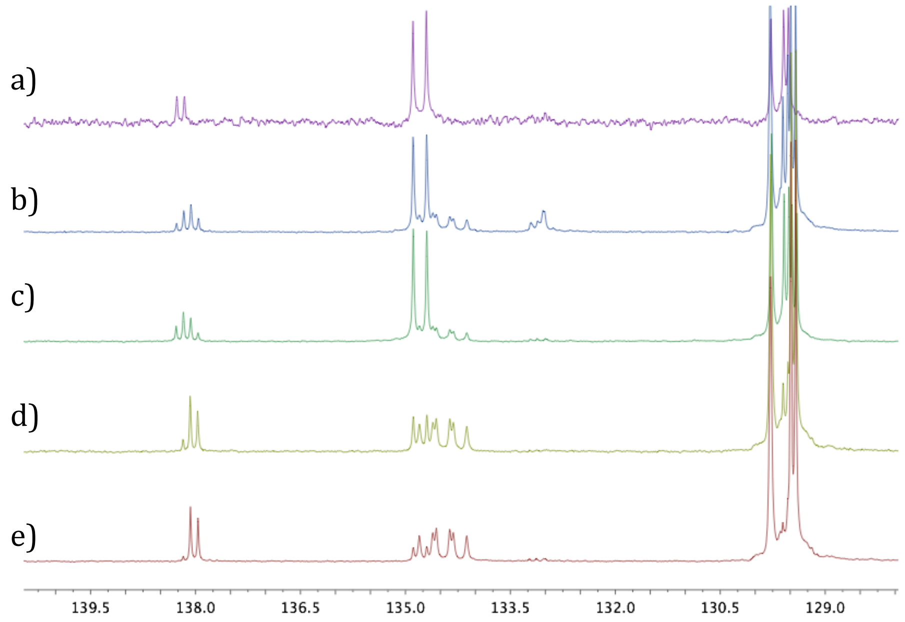

Figure S4. $^{13}C\{^{1}H\}$ NMR ($CDCl_3$, 100.6MHz) spectra of PPh_3 (**1**) deuteration evolution using **Ru@PVP**: (a) t=0, (b) 16h, (c) 36h, and (d) 48h at 55°C and (e) 48h at 80°C

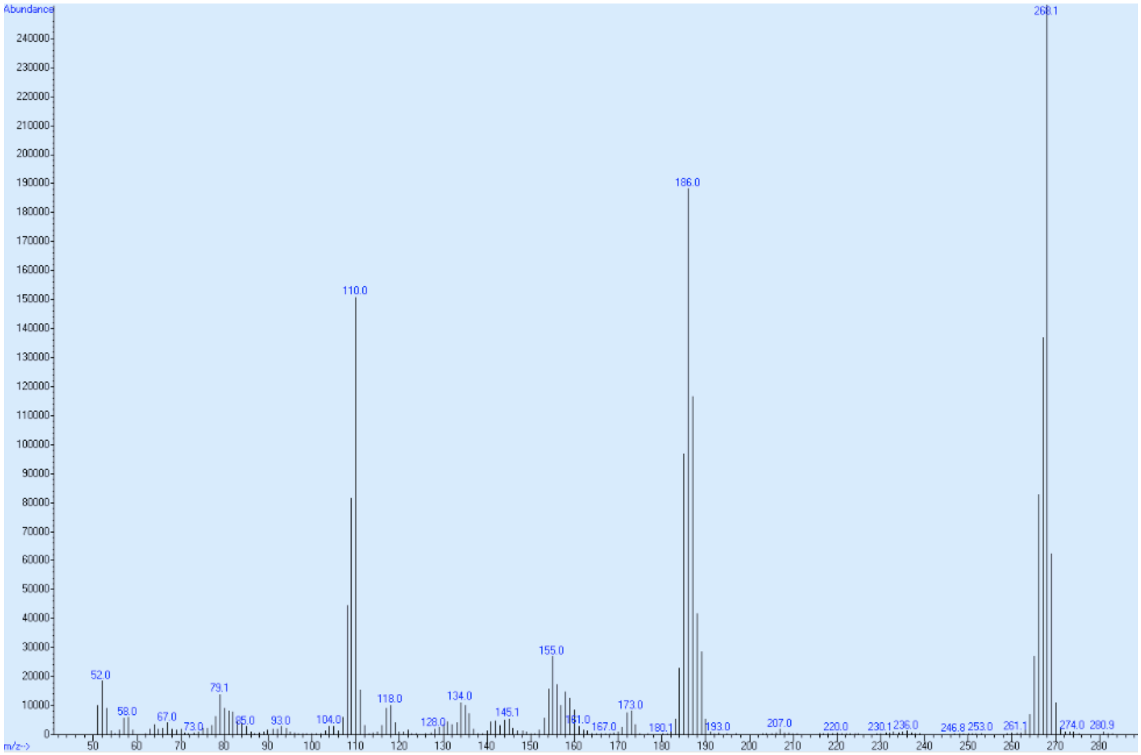

Figure S5. Mass spectrum of the deuteration of PPh_3 (**1**) using **Ru@PVP** after 48h at 80°C.

4.1.2. Using **Rh@PVP**:

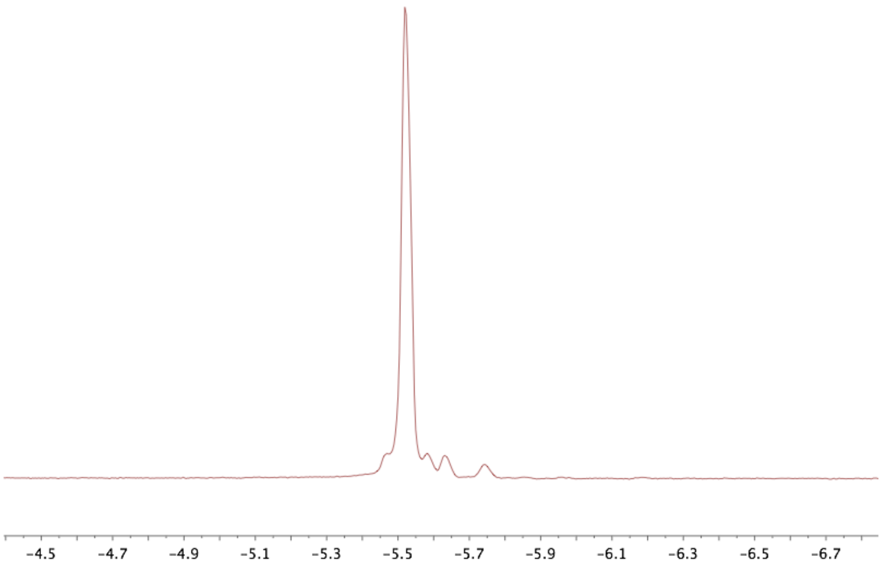

Figure S6. $^{31}P\{^1H\}$ NMR ($CDCl_3$, 162MHz) spectrum the deuteration of PPh_3 (**1**) using **Rh@PVP** after 48h at 55°C. The signal of highest intensity corresponds to non-deuterated PPh_3.

4.1.3. Using **Ru@NHC**:

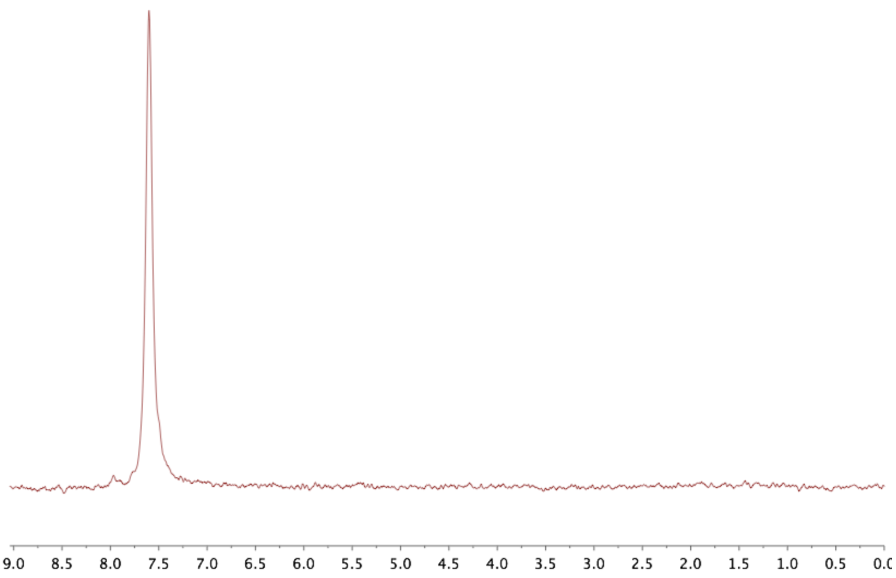

Figure S7. ^{2}D NMR ($CHCl_3$, 61.49MHz) spectrum of the deuteration of PPh_3 (**1**) using **Ru@NHC** after 48h at 55°C.

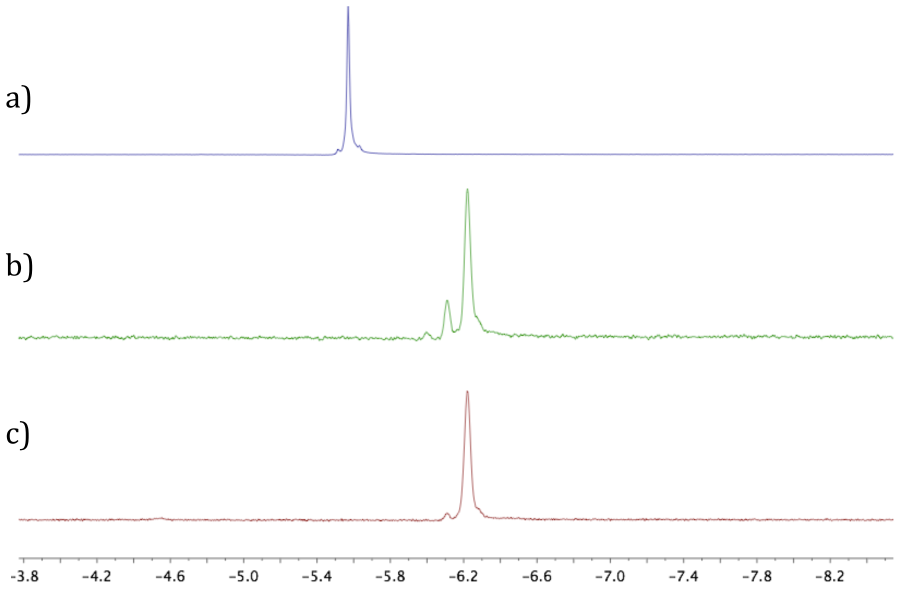

Figure S8. $^{31}P\{^{1}H\}$ NMR ($CDCl_3$, 162MHz) spectra of PPh_3 (**1**) deuteration evolution using **Ru@NHC**: (a) t=0, (b) 48h and (c) 8 days at 55°C.

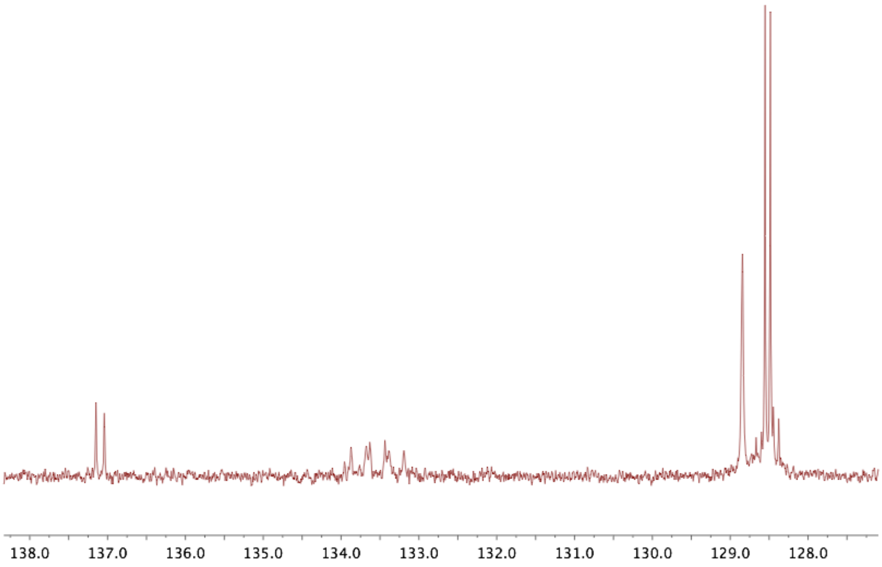

Figure S9. $^{13}C\{^{1}H\}$ NMR ($CDCl_3$, 100.6MHz) spectrum of the deuteration of PPh_3 (**1**) using **Ru@NHC** after 48h at 55°C.

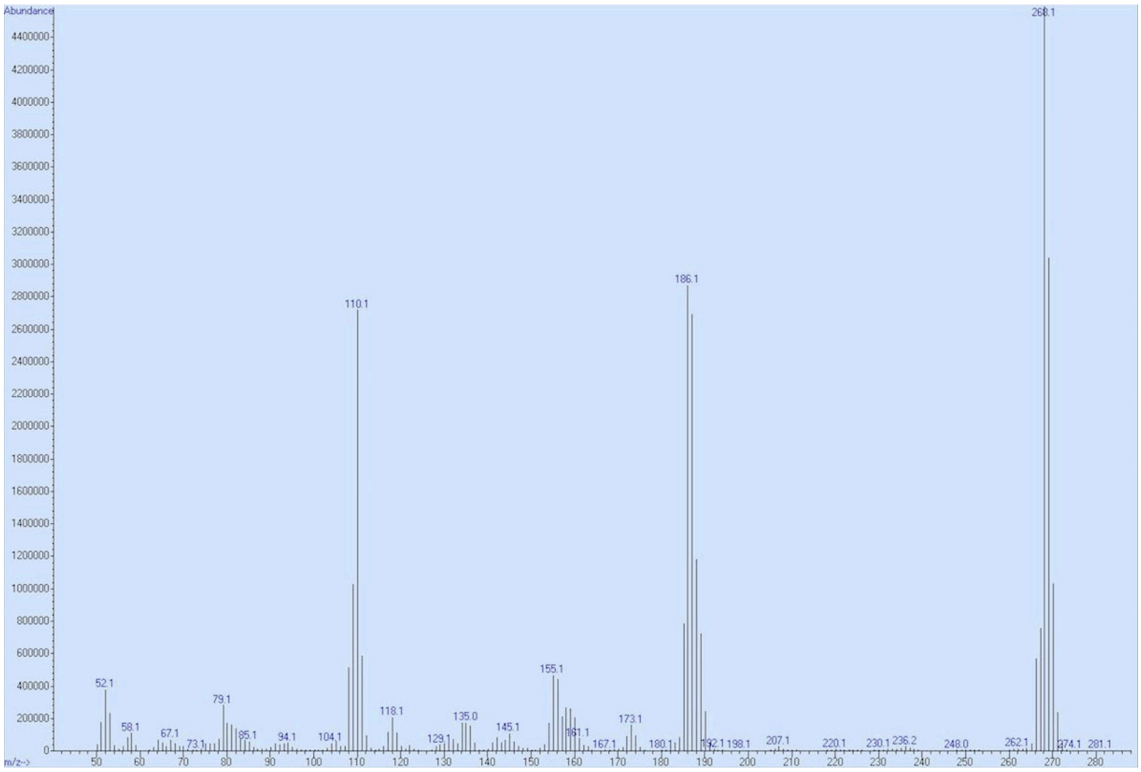

Figure S10. Mass spectrum of the deuteration of PPh_3 (**1**) using **Ru@NHC** after 8 days at 55°C.

4.1.4. Using **Rh@NHC**:

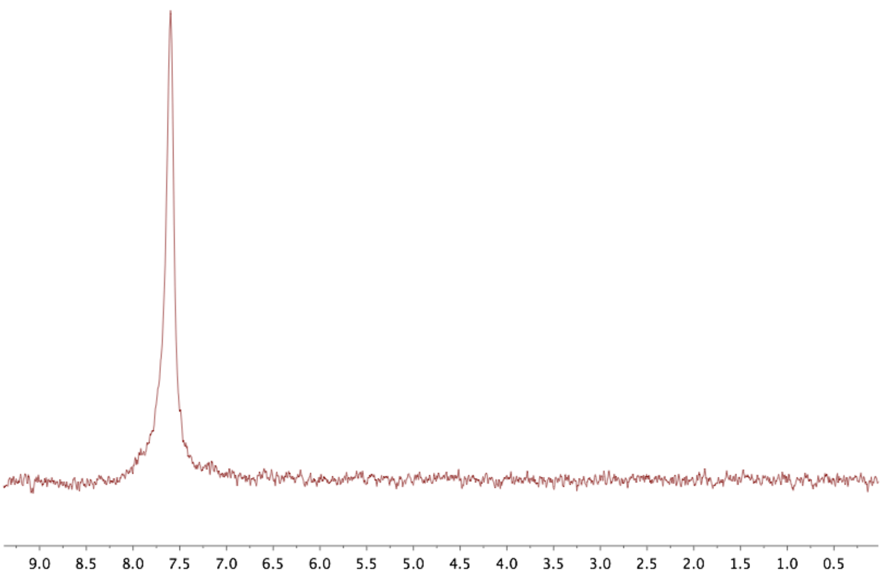

Figure S11. ^{2}D NMR ($CHCl_3$, 61.49MHz) spectrum of the deuteration of PPh_3 (**1**) using **Rh@NHC** after 48h at 55°C.

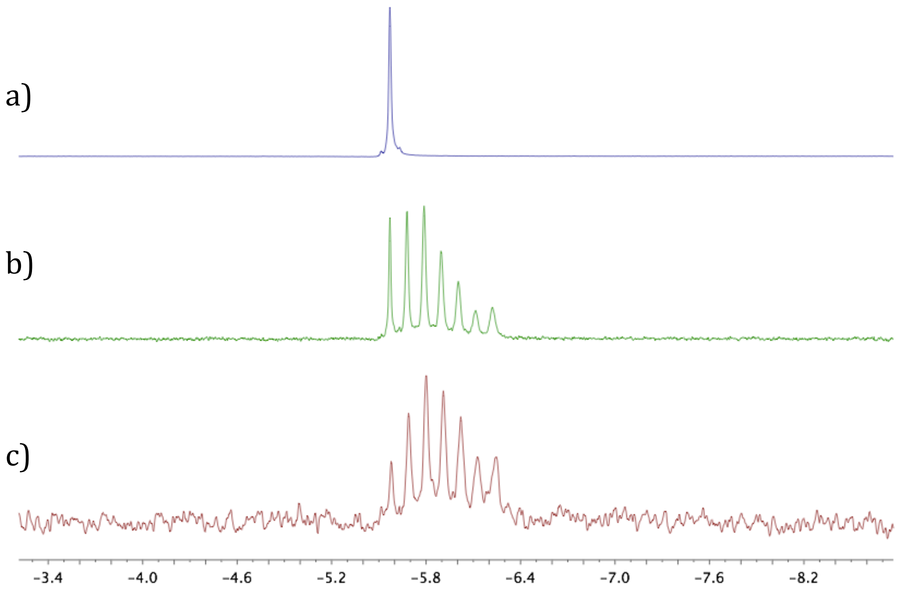

Figure S12. $^{31}P\{^{1}H\}$ NMR ($CDCl_3$, 162MHz) spectra of PPh_3 (**1**) deuteration evolution using **Rh@NHC**: (a) t=0, (b) 48h and c) 8 days at 55°C.

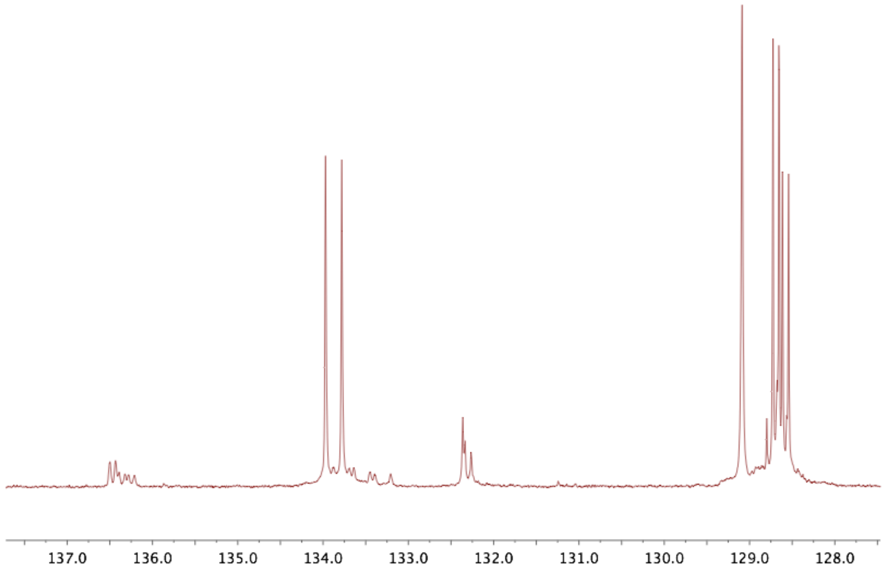

Figure S13. $^{13}C\{^{1}H\}$ NMR ($CDCl_3$, 100.6MHz) spectrum of the deuteration of PPh_3 (**1**) using **Rh@NHC** after 48h at 55°C.

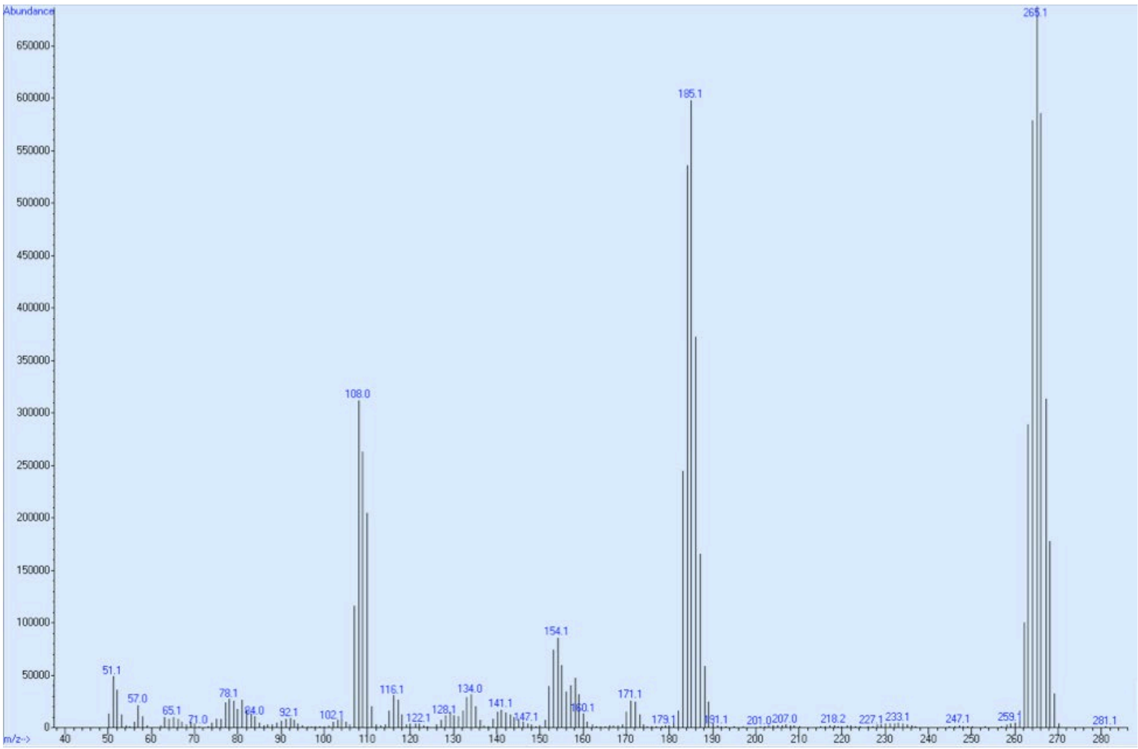

Figure S14. Mass spectrum of the reaction mixture for the deuteration of PPh_3 (**1**) using **Rh@NHC** after 8 days at 55°C.

4.2. H/D exchange of P(*p*-tolyl)$_3$ (2):

4.2.1. Using **Ru@PVP**:

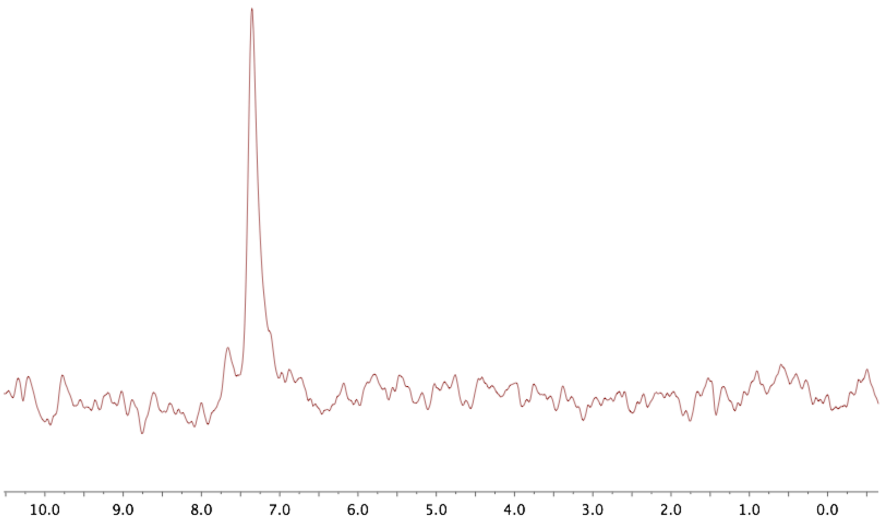

Figure S15. ^{2}D NMR ($CHCl_3$, 61.49MHz) spectrum of the deuteration of P(*p*-tolyl)$_3$ (**2**) using **Ru@PVP** after 48h at 55°C.

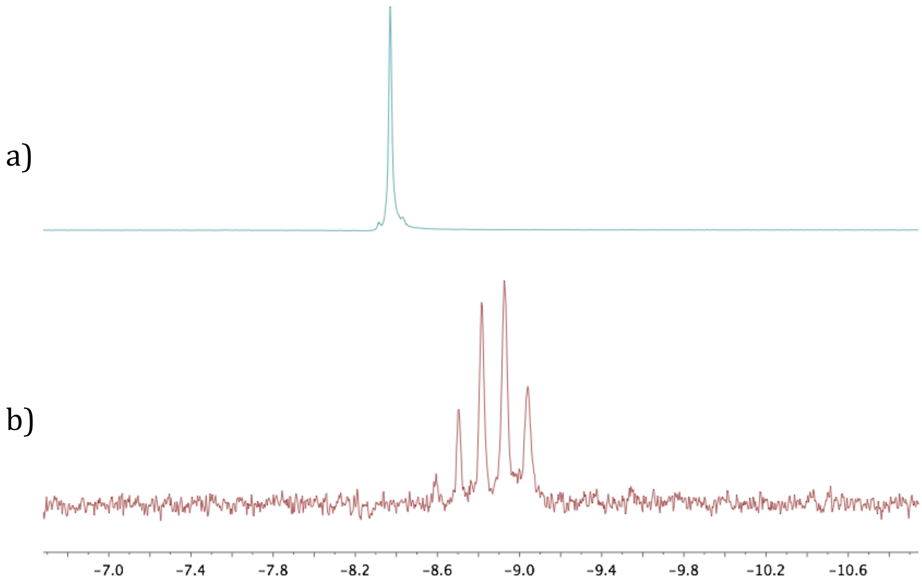

Figure S16. $^{31}P\{^1H\}$ NMR ($CDCl_3$, 162MHz) spectra of P(*p*-tolyl)$_3$ (**2**) deuteration evolution using **Ru@PVP**: (a) t=0 and (b) 48h at 55°C.

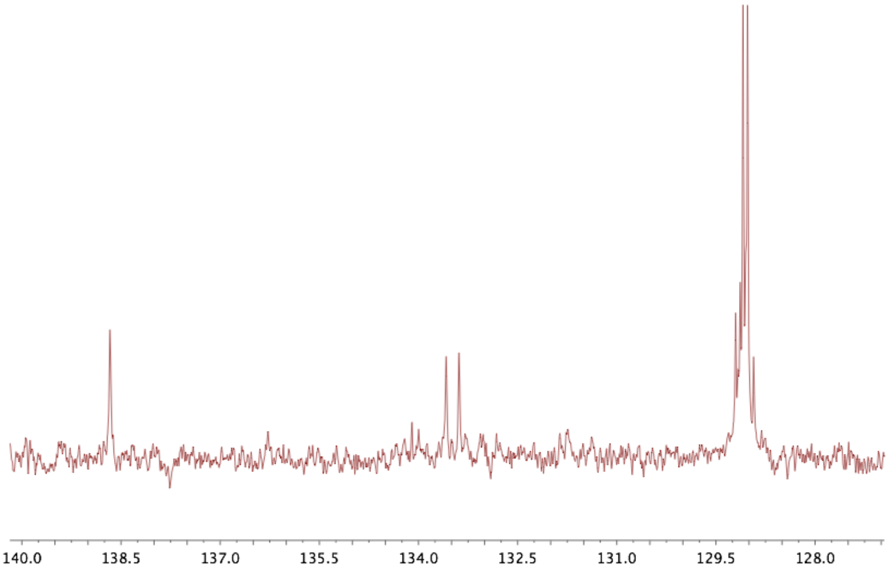

Figure S17. $^{13}C\{^{1}H\}$ NMR ($CDCl_3$, 100.6MHz) spectrum of deuteration of P(*p*-tolyl)$_3$ (**2**) using **Ru@PVP** after 48h at 55°C.

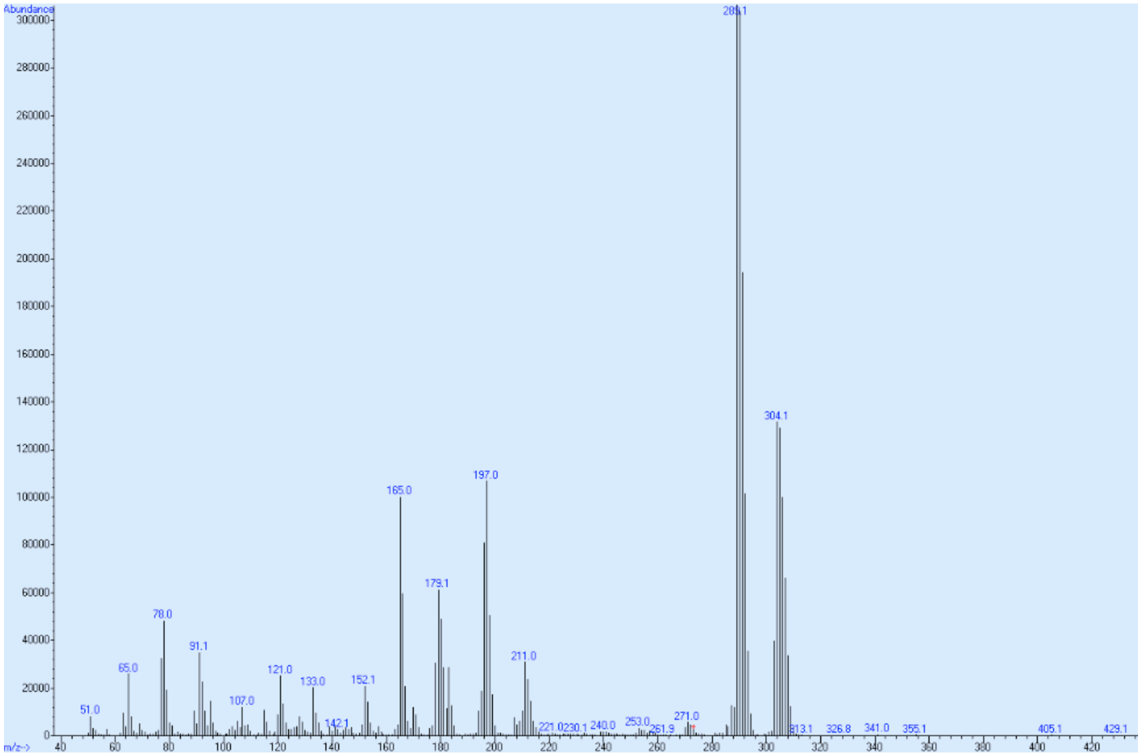

Figure S18. Mass spectrum of the deuteration of P(*p*-tolyl)$_3$ (**2**) using **Ru@PVP** after 48 days at 55°C.

4.2.2.Using **Ru@NHC**:

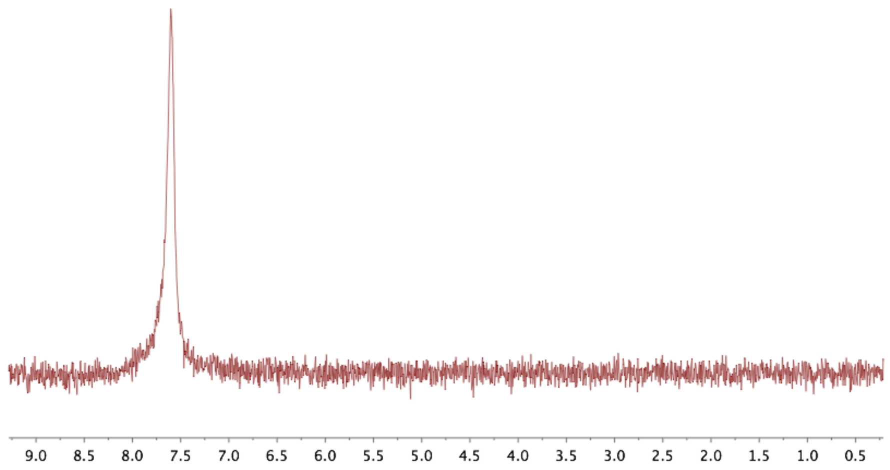

Figure S19. ^{2}D NMR ($CHCl_3$, 61.49MHz) spectrum of the deuteration of P(*p*-tolyl)$_3$ (**2**) using **Ru@NHC** after 48h at 55°C.

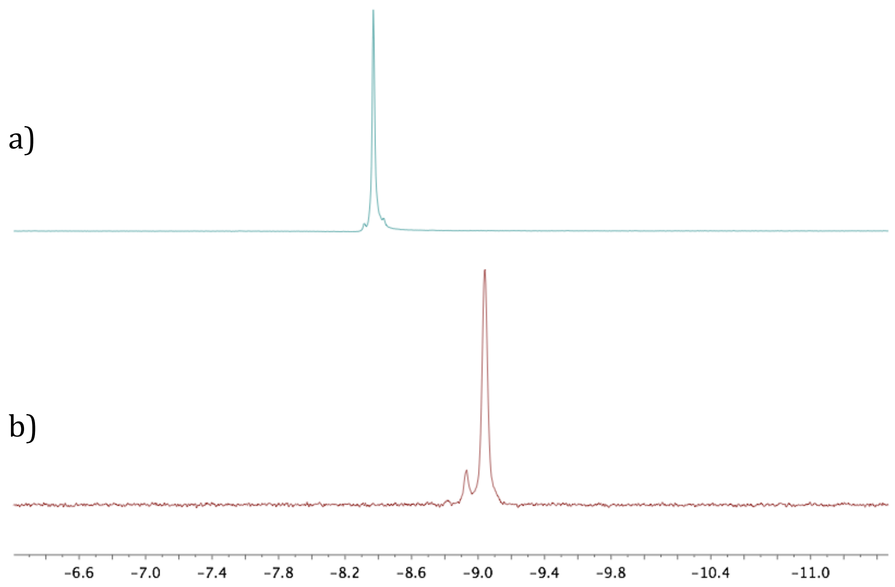

Figure S20. $^{31}P\{^{1}H\}$ NMR ($CDCl_3$, 162MHz) spectra of P(*p*-tolyl)$_3$ (**2**) deuteration evolution using **Ru@NHC**: (a) t=0 and (b) 48h at 55°C.

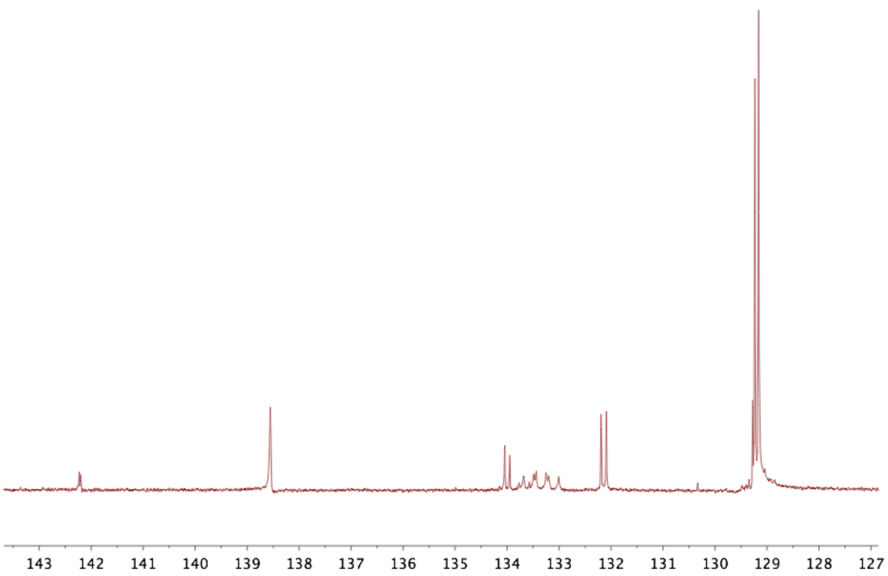

Figure S21. $^{13}C\{^{1}H\}$ NMR ($CDCl_3$, 100.6MHz) spectrum of the deuteration of P(*p*-tolyl)$_3$ (**2**) using **Ru@NHC** after 48h at 55°C.

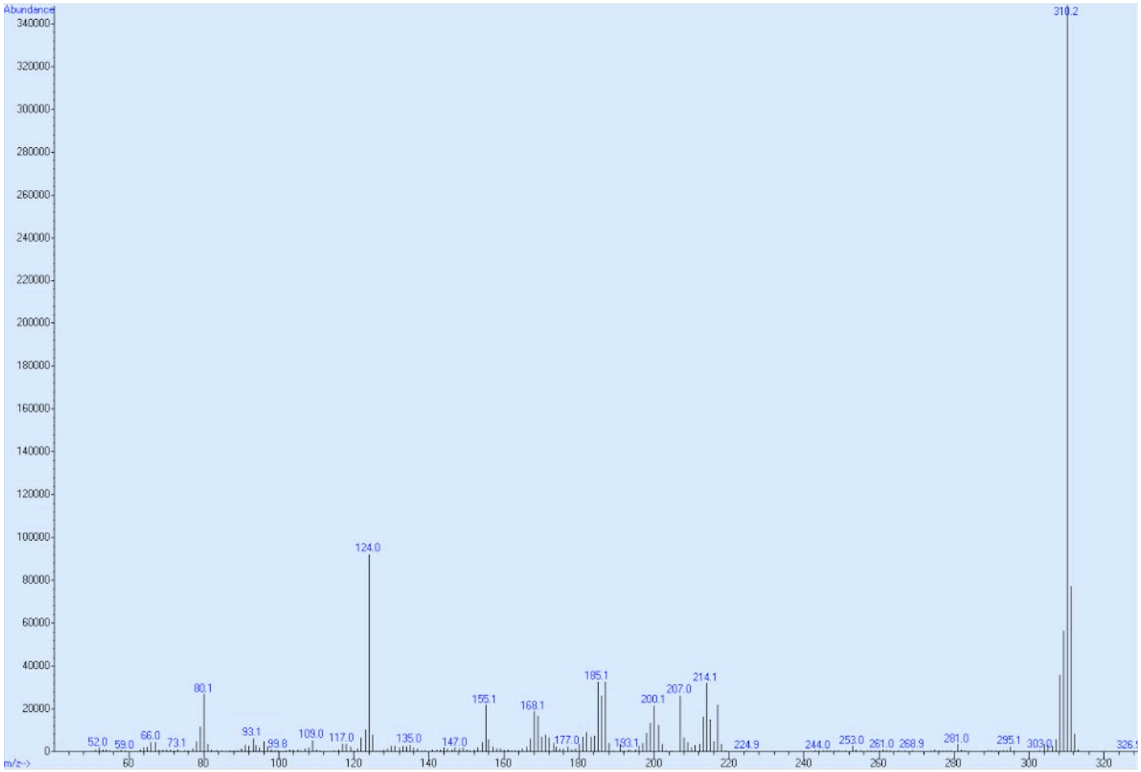

Figure S22. Mass spectrum of the deuteration of P(*p*-tolyl)$_3$ (**2**) using **Ru@NHC** after 8 days at 55°C.

4.2.3. Using **Rh@NHC**:

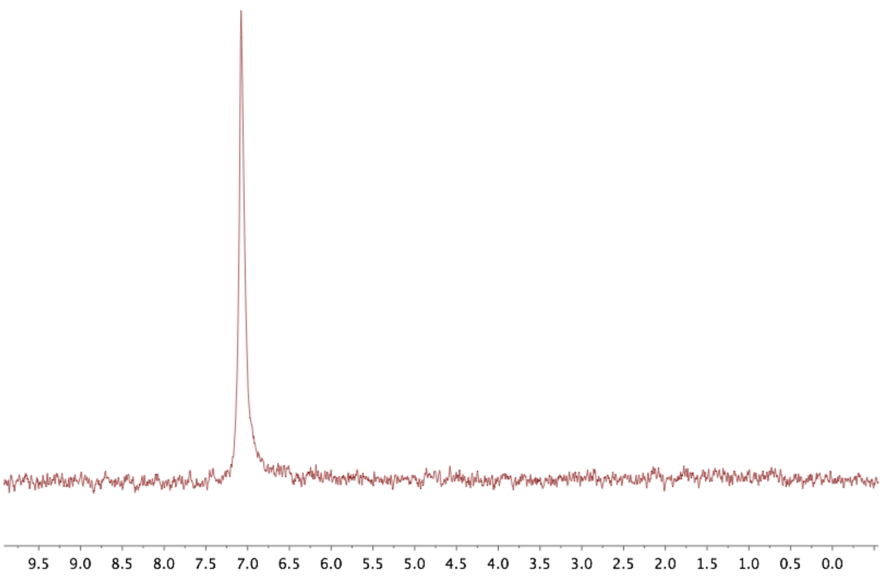

Figure S23. ^{2}D NMR ($CHCl_3$, 61.49MHz) spectrum of the deuteration of P(*p*-tolyl)$_3$ (**2**) using **Rh@NHC** after 48h at 55°C.

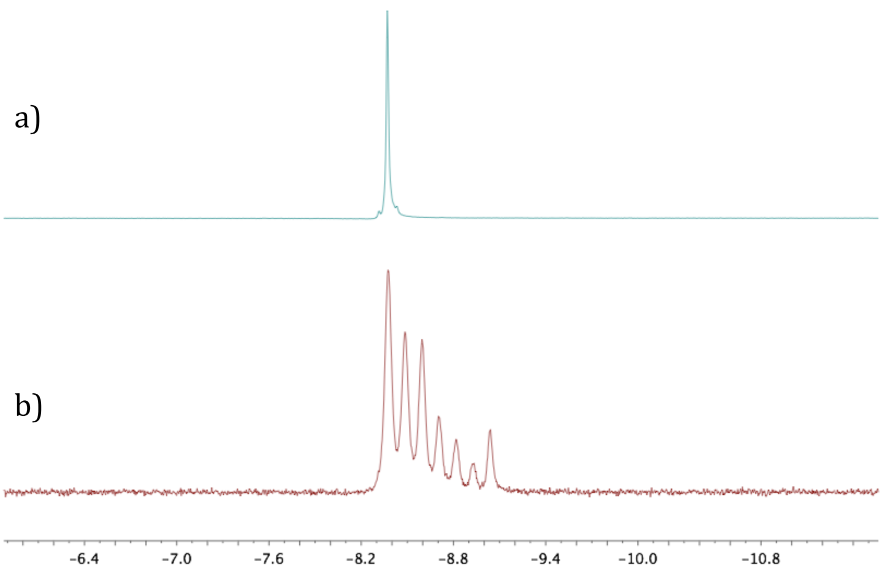

Figure S24. $^{31}P\{^{1}H\}$ NMR ($CDCl_3$, 162MHz) spectra of P(*p*-tolyl)$_3$ (**2**) deuteration evolution using **Rh@NHC**: (a) t=0 and (b) 48h at 55°C.

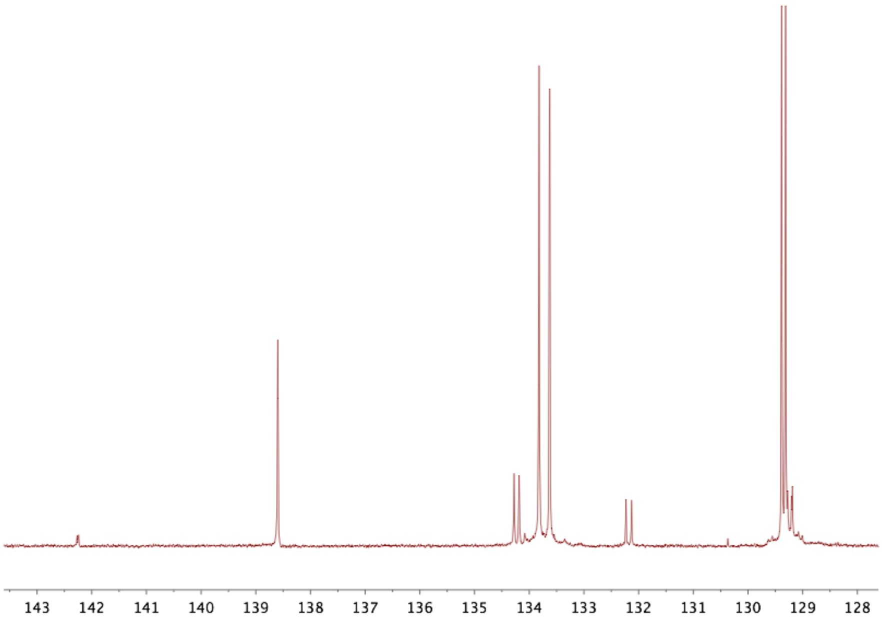

Figure S25. $^{13}C\{^1H\}$ NMR ($CDCl_3$, 100.6MHz) spectrum of the deuteration of P(*p*-tolyl)$_3$ (**2**) using **Rh@NHC** after 48h at 55°C.

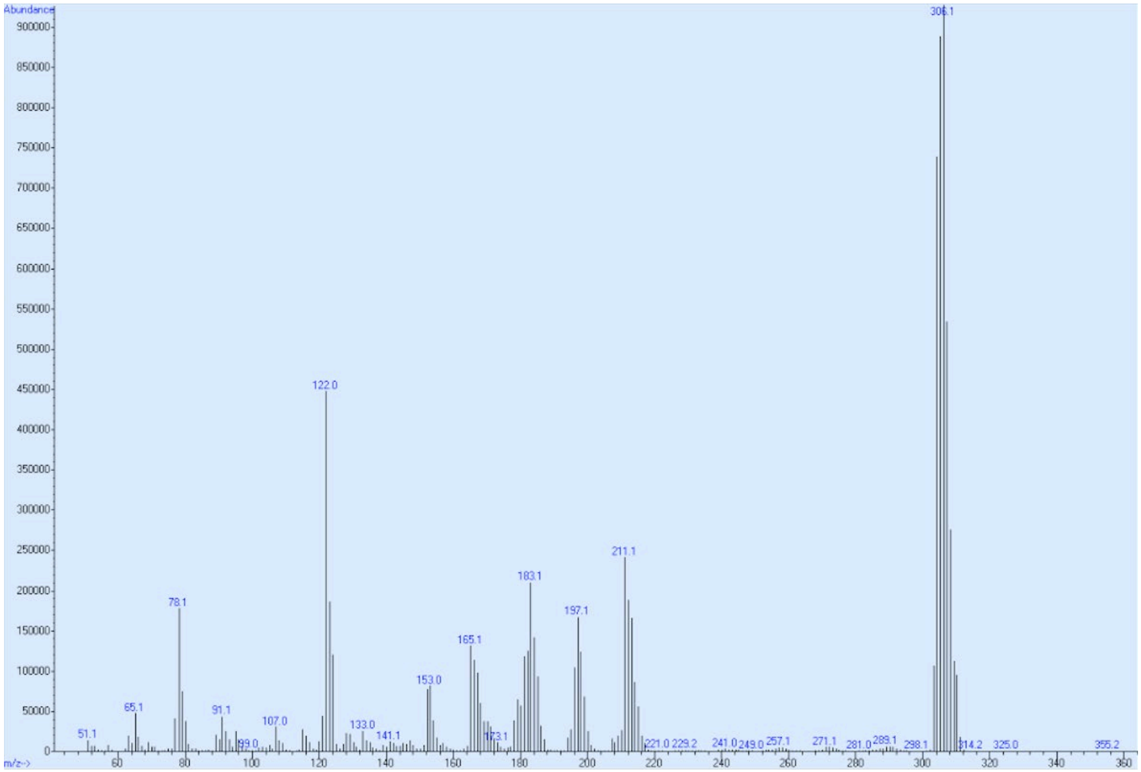

Figure S26. Mass spectrum of the deuteration of P(*p*-tolyl)$_3$ (**2**) using **Rh@NHC** after 8 days at 55°C.

4.3.H/D exchange of PPh_2Me (3):

4.3.1.Using **Ru@PVP**:

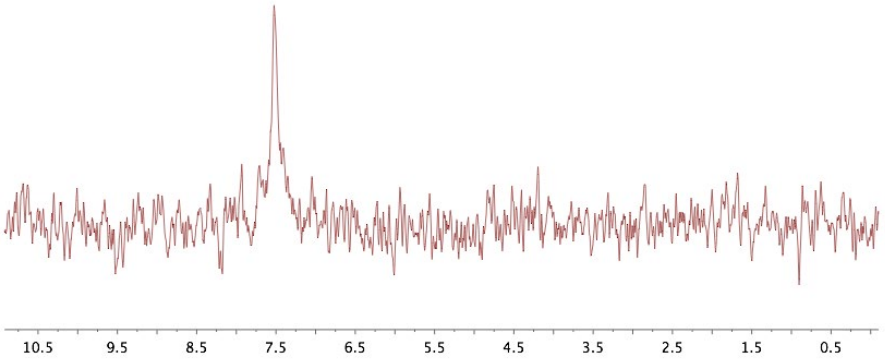

Figure S27. ^2D NMR ($CHCl_3$, 61.49MHz) spectrum of the deuteration of PPh_2Me (**3**) using **Ru@PVP** after 88h at 80°C.

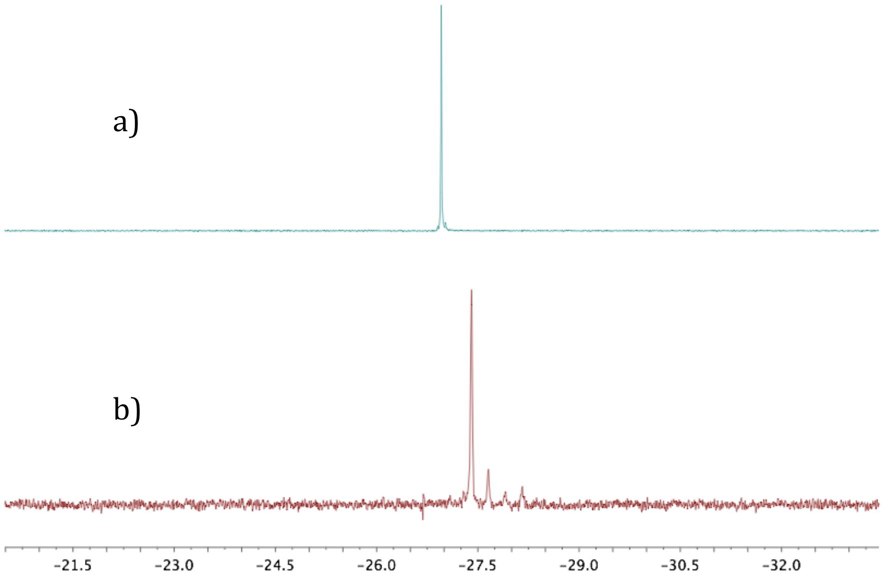

Figure S28. $^{31}P\{^1H\}$ NMR ($CDCl_3$, 162MHz) spectra of PPh_2Me (**3**) deuteration evolution using **Ru@PVP**: (a) t=0 and (b) 88h at 80°C.

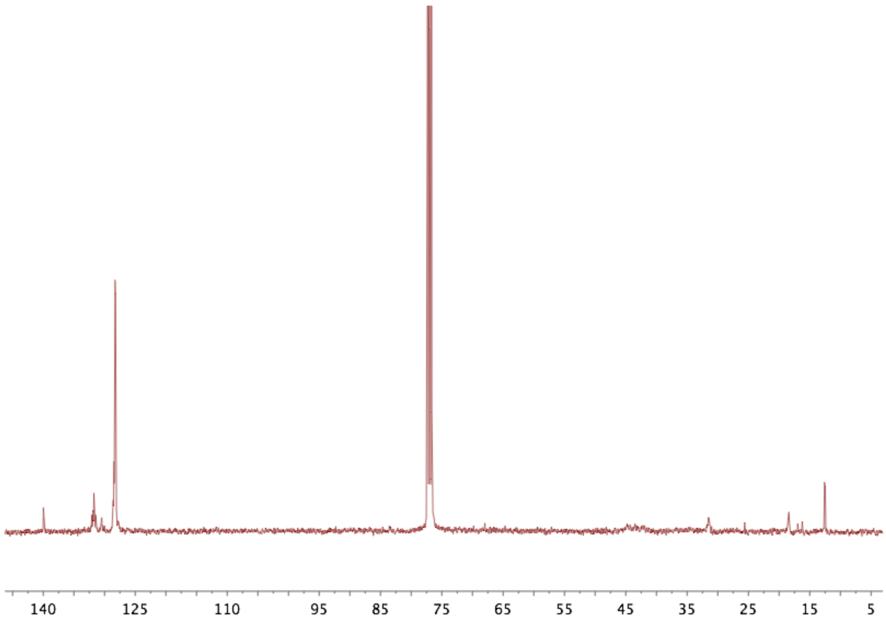

Figure S29. $^{13}C\{^{1}H\}$ NMR ($CDCl_3$, 100.6MHz) spectrum of the deuteration of PPh_2Me (**3**) using **Ru@PVP** after 88h at 80°C.

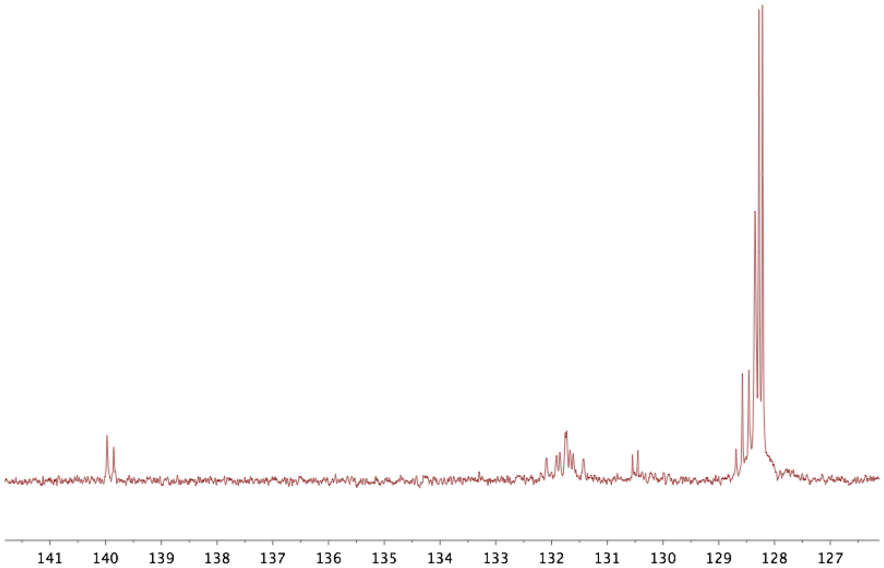

Figure S30. $^{13}C\{^{1}H\}$ NMR ($CDCl_3$, 100.6MHz) spectrum of the deuteration of PPh_2Me (**3**) using **Ru@PVP** after 88h at 80°C.

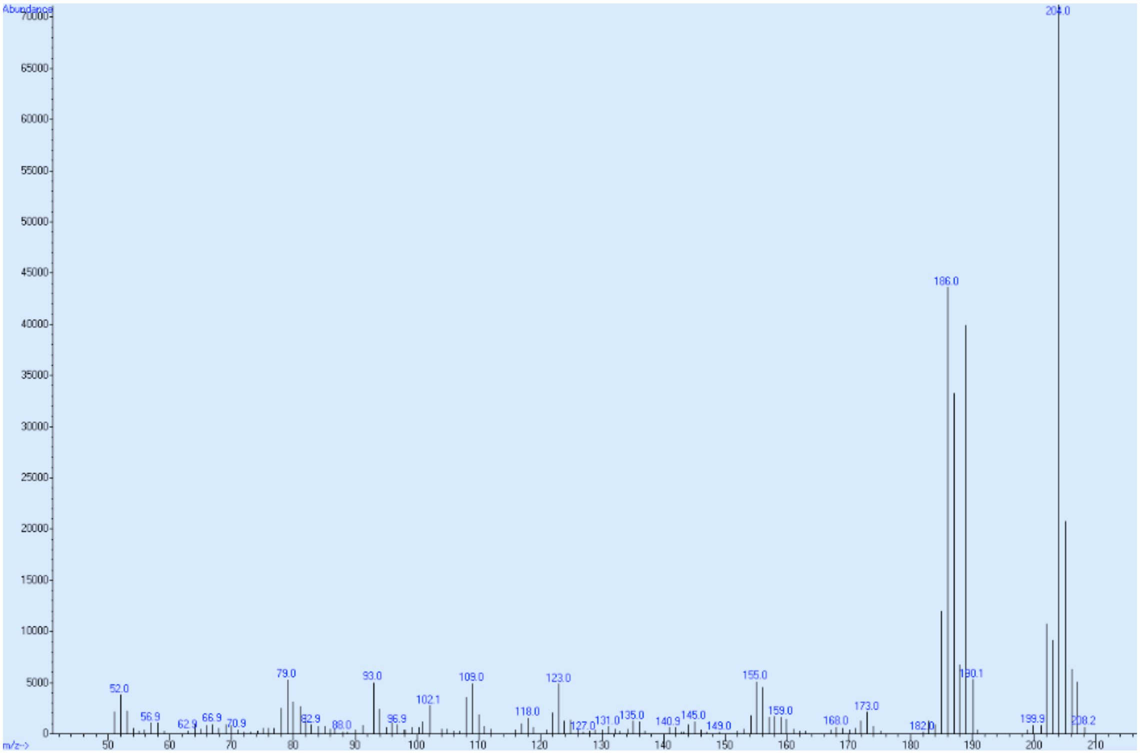

Figure S31. Mass spectrum of the deuteration of PPh_2Me (**3**) using **Ru@PVP** after 88h at 80°C

4.3.2. Using **Ru@NHC**:

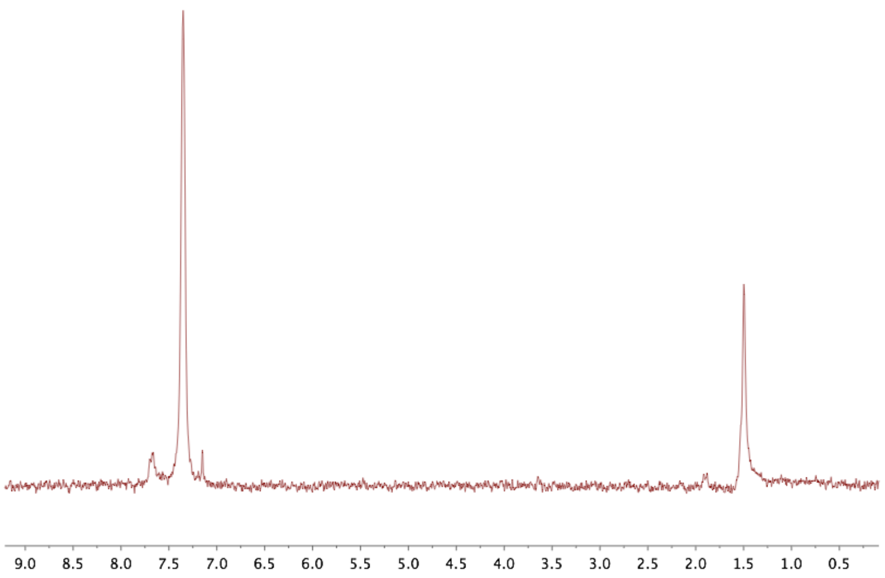

Figure S32. ^{2}D NMR ($CHCl_3$, 61.49MHz) spectrum of the deuteration of PPh_2Me (**3**) using **Ru@NHC** after 8 days at 55°C.

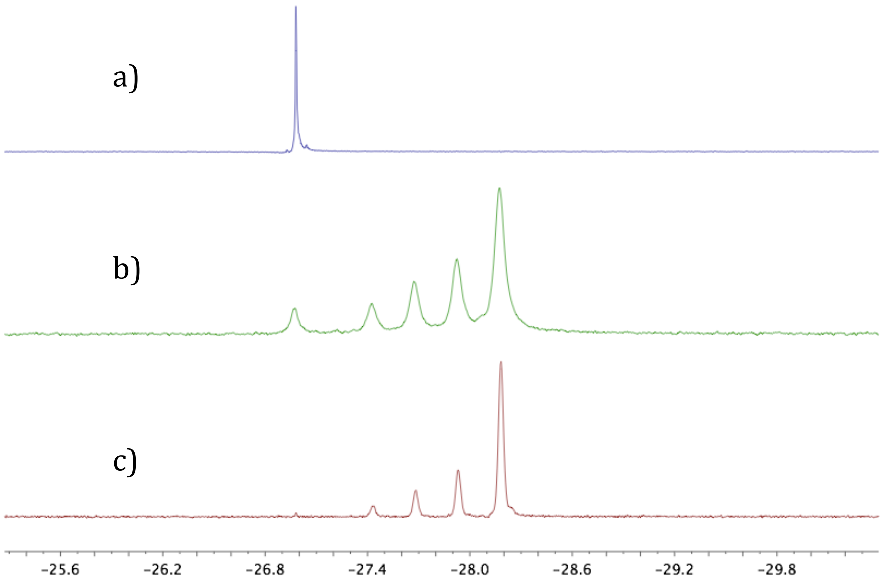

Figure S33. $^{31}P\{^{1}H\}$ NMR ($CDCl_3$, 162MHz) spectra of PPh_2Me (**3**) deuteration evolution using **Ru@NHC**: (a) t=0, (b) 48h and (c) 8 days at 55°C.

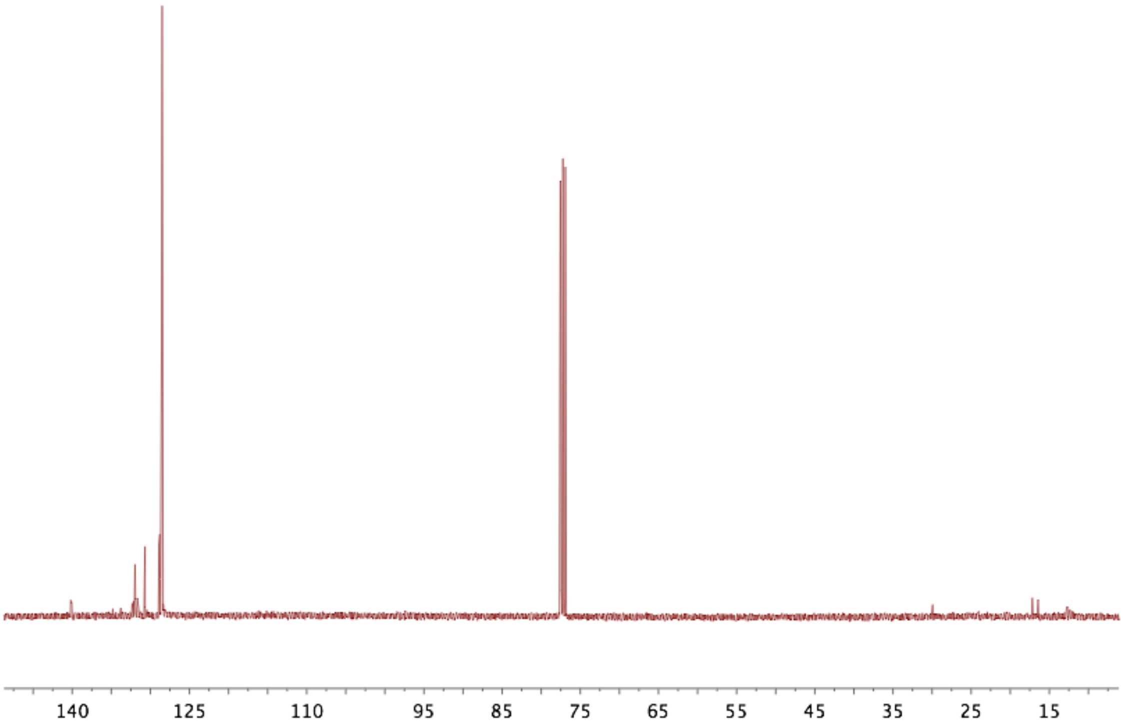

Figure S34. $^{13}C\{^{1}H\}$ NMR ($CDCl_3$, 100.6MHz) spectrum of the deuteration of PPh_2Me (**3**) using **Ru@NHC** after 8 days at 55°C.

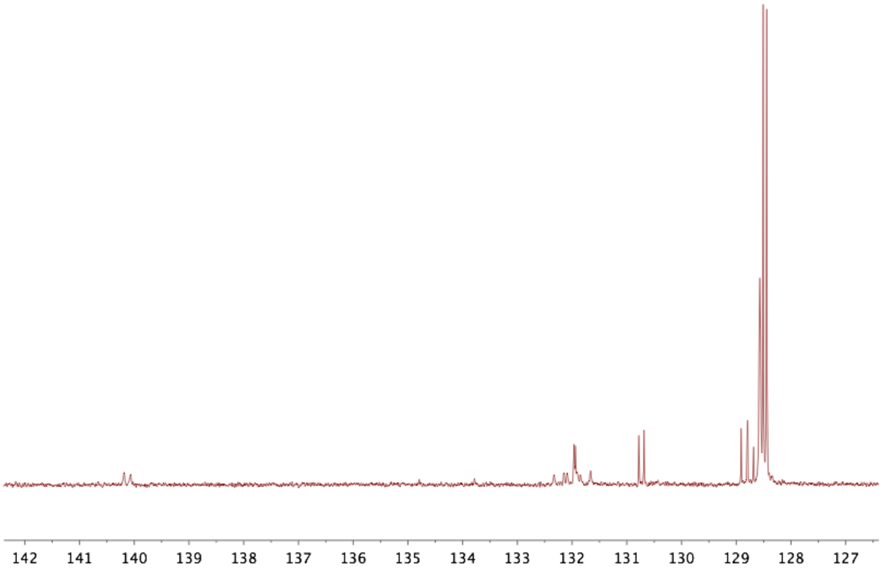

Figure S35. $^{13}C\{^{1}H\}$ NMR ($CDCl_3$, 100.6MHz) spectrum of the deuteration of PPh_2Me (**3**) using **Ru@NHC** after 8 days at 55°C.

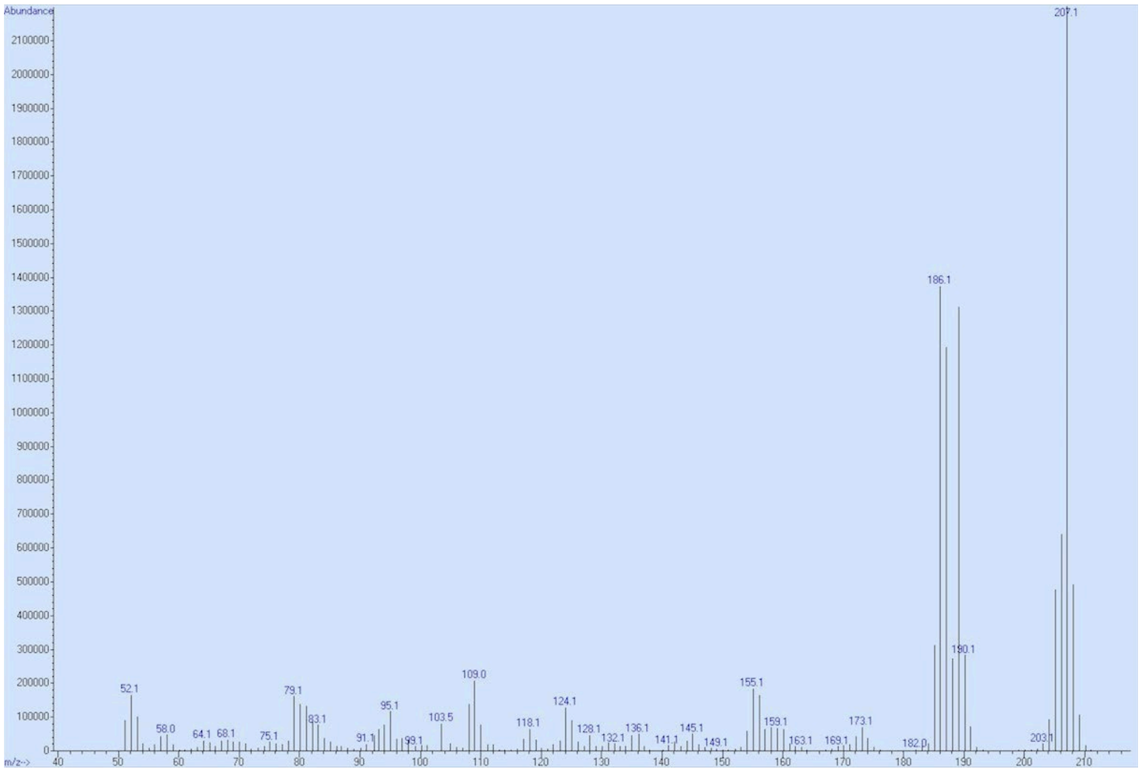

Figure S36. Mass spectrum of the deuteration of PPh_2Me (**3**) using **Ru@NHC** after 8 days at 55°C.

4.3.3.Using **Rh@NHC**:

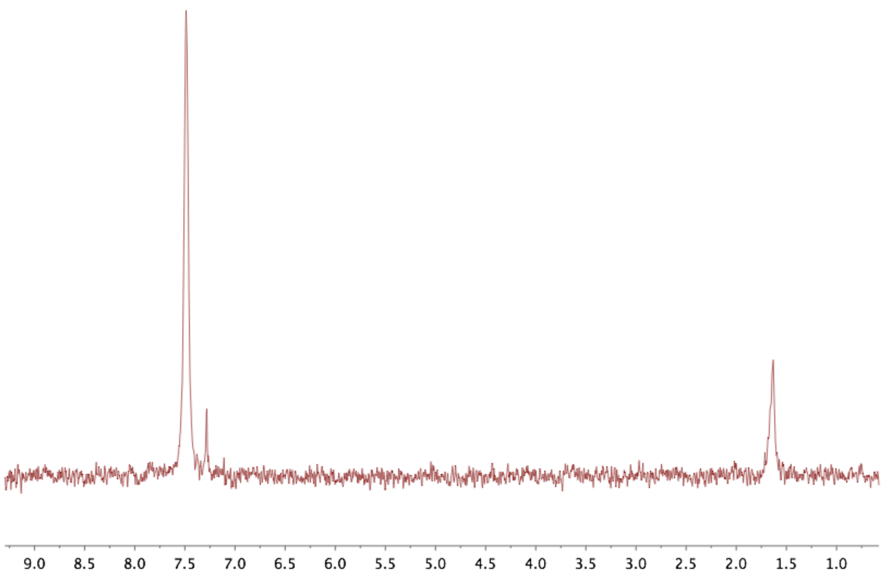

Figure S37. ^{2}D NMR ($CHCl_3$, 61.49MHz) spectrum of the deuteration of PPh_2Me (**3**) using **Rh@NHC** after 48h at 55°C.

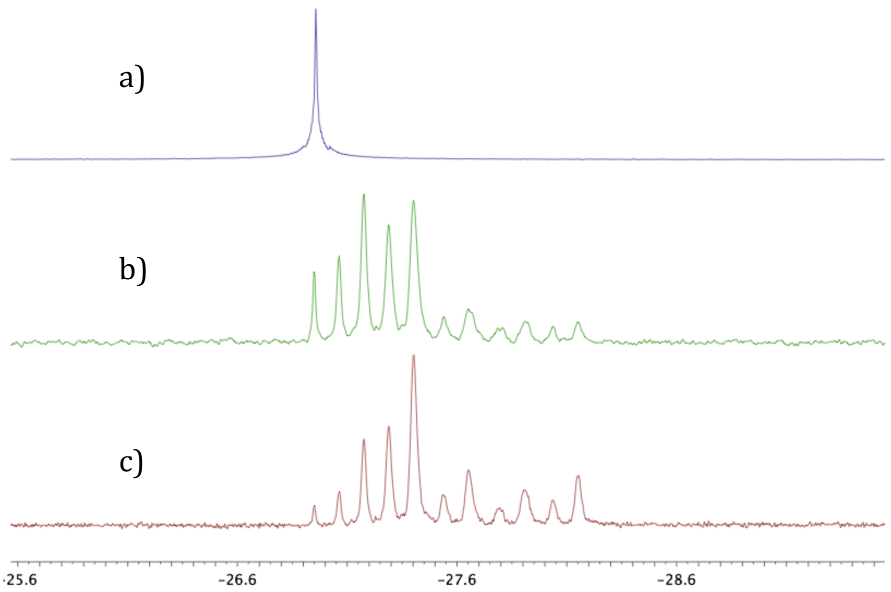

Figure S38. $^{31}P\{^{1}H\}$ NMR ($CDCl_3$, 162MHz) spectra of PPh_2Me (**3**) deuteration evolution using **Rh@NHC**: (a) t=0, (b) 48h and (c) 8 days at 55°C.

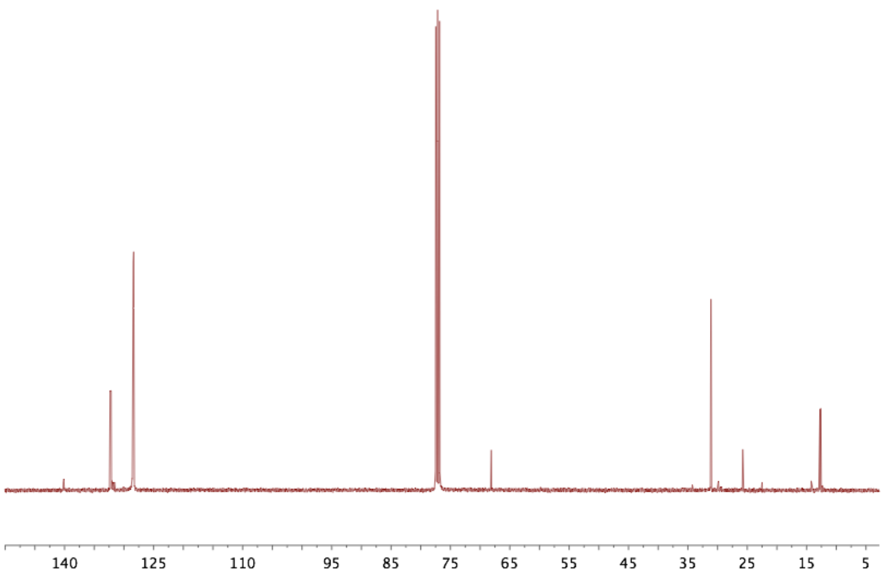

Figure S39. $^{13}C\{^{1}H\}$ NMR ($CDCl_3$, 100.6MHz) spectrum of the deuteration of PPh_2Me (**3**) using **Rh@NHC** after 48h at 55°C.

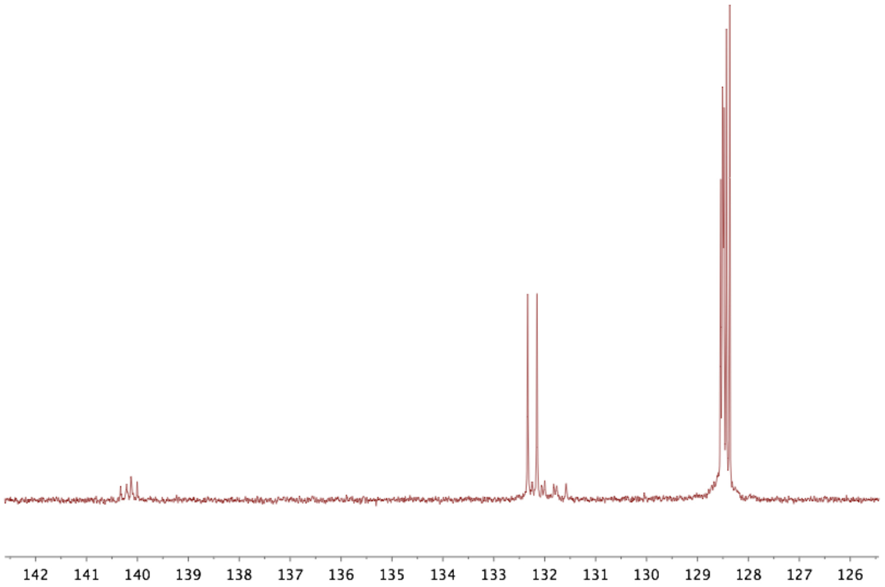

Figure S40. $^{13}C\{^{1}H\}$ NMR ($CDCl_3$, 100.6MHz) spectrum of the deuteration of PPh_2Me (**3**) using **Rh@NHC** after 48h at 55°C.

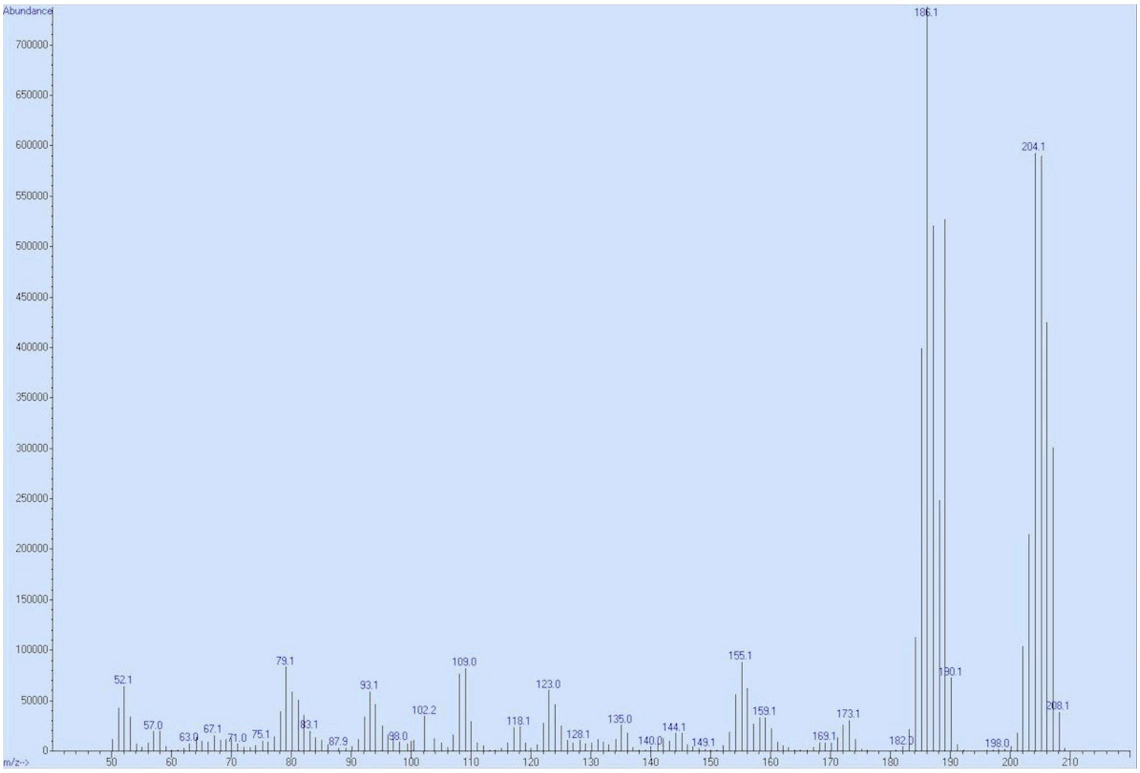

Figure S41. Mass spectrum of the deuteration of PPh_2Me (**3**) using **Rh@NHC** after 8 days at 55°C.

4.4. H/D exchange of P(*o*-tolyl)$_3$ (4):

4.4.1. Using **Ru@NHC**:

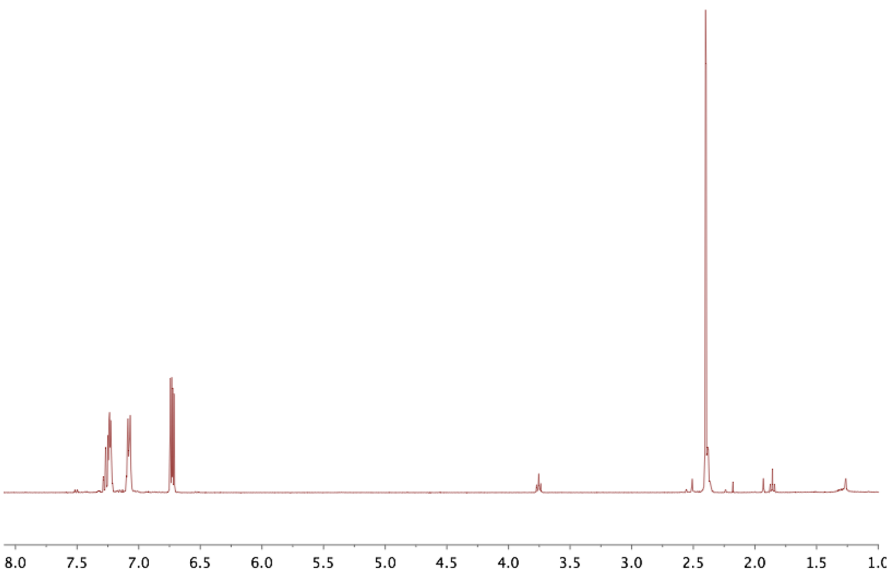

Figure S42. ^{1}H NMR ($CDCl_3$, 400MHz) spectra of the deuteration of P(*o*-tolyl)$_3$ (**4**) using **Ru@NHC** after 48h at 55°C.

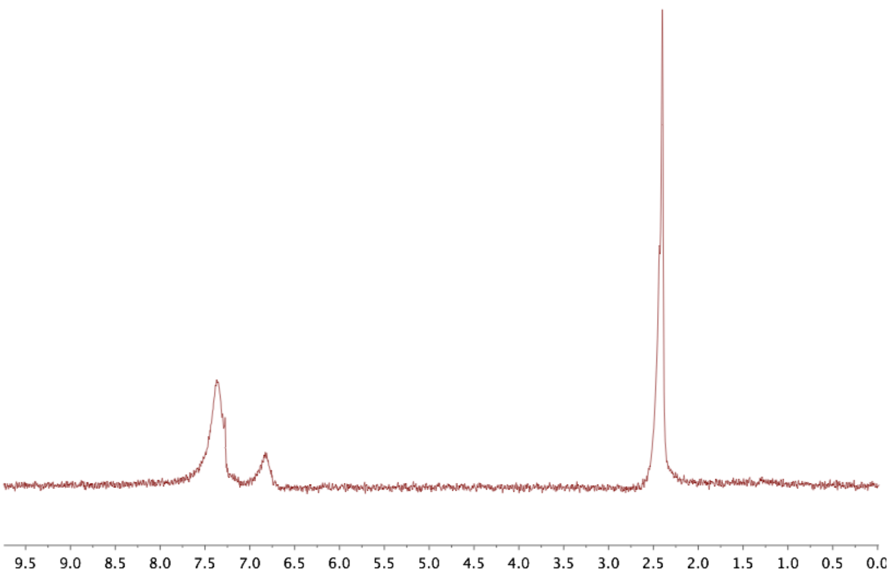

Figure S43. ^{2}D NMR ($CHCl_3$, 61.49MHz) spectrum of the deuteration of P(*o*-tolyl)$_3$ (**4**) using **Ru@NHC** after 48h at 55°C

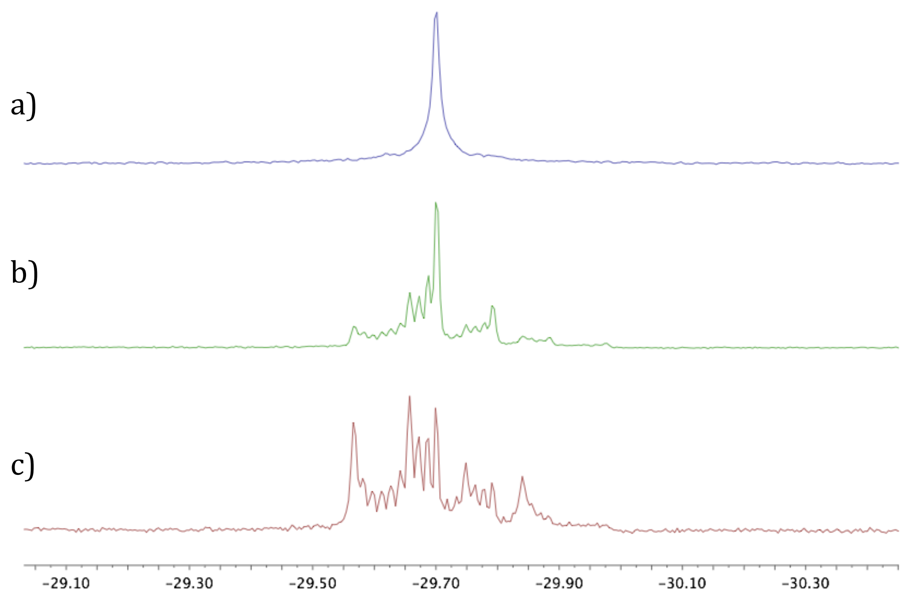

Figure S44. $^{31}P\{^{1}H\}$ NMR ($CDCl_3$, 162MHz) spectra of P(*o*-tolyl)$_3$ (**4**) deuteration evolution using **Ru@NHC**: (a) t=0, (b) 48h and (c) 8 days at 55°C.

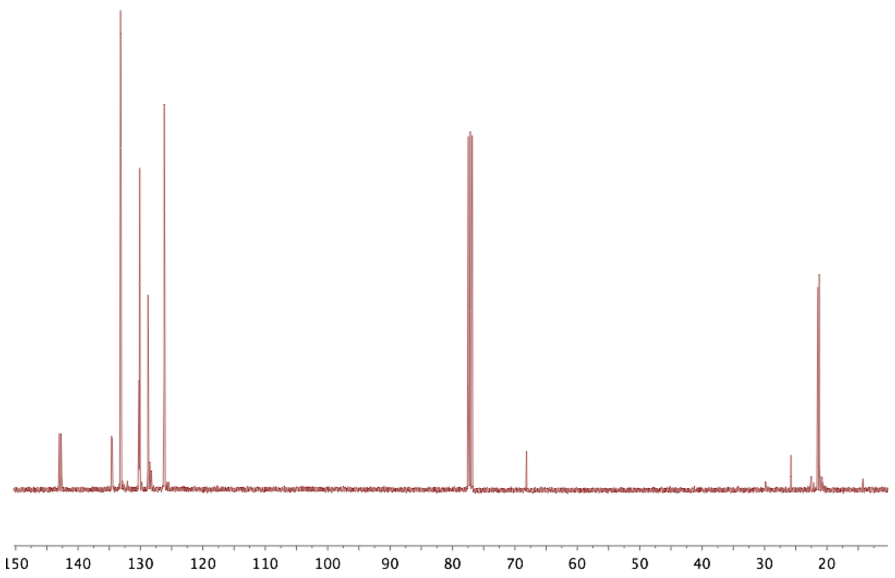

Figure S45. $^{13}C\{^{1}H\}$ NMR ($CDCl_3$, 100.6MHz) spectrum of the deuteration of P(*o*-tolyl)$_3$ (**4**) using **Ru@NHC** after 8 days at 55°C.

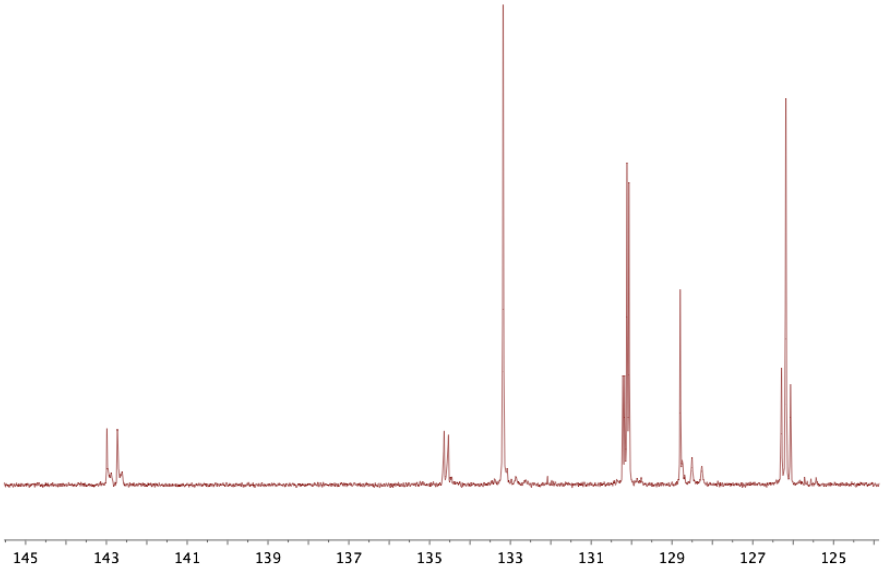

Figure S46. $^{13}C\{^{1}H\}$ NMR ($CDCl_3$, 100.6MHz) spectrum of the deuteration of P(*o*-tolyl)$_3$ (**4**) using **Ru@NHC** after 8 days at 55°C

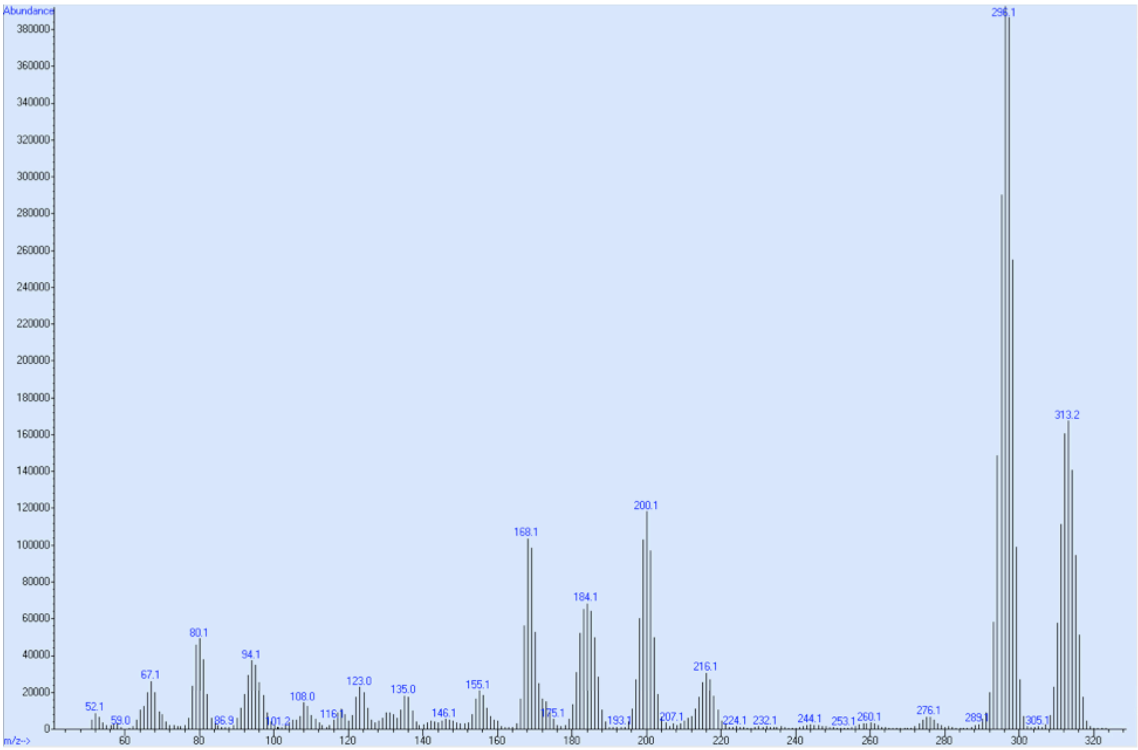

Figure S47. Mass spectrum of the deuteration of P(*o*-tolyl)$_3$ (**4**) using **Ru@NHC** after 8 days at 55°C.

4.4.2.Using **Rh@NHC**:

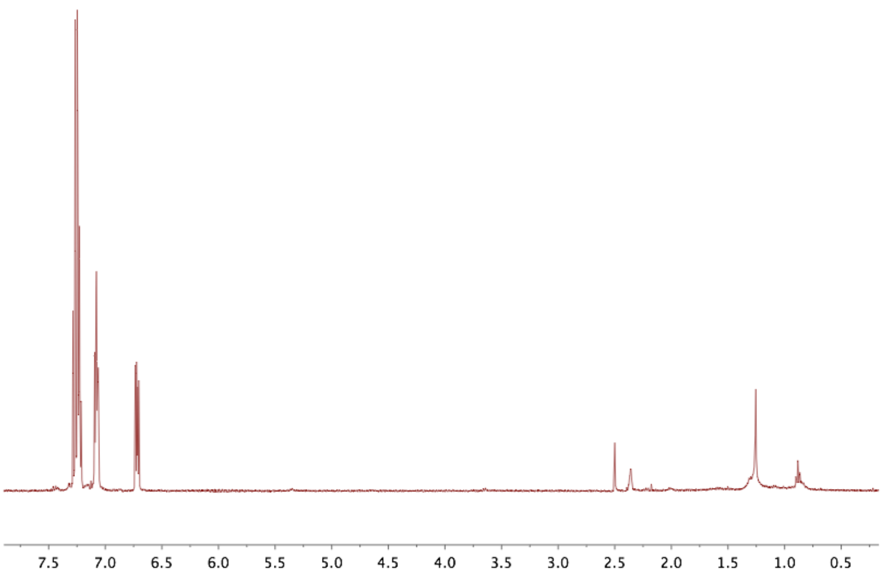

Figure S48. ^{1}H NMR ($CDCl_3$, 400MHz) spectra of the deuteration of P(*o*-tolyl)$_3$ (**4**) using **Rh@NHC** after 48h at 55°C.

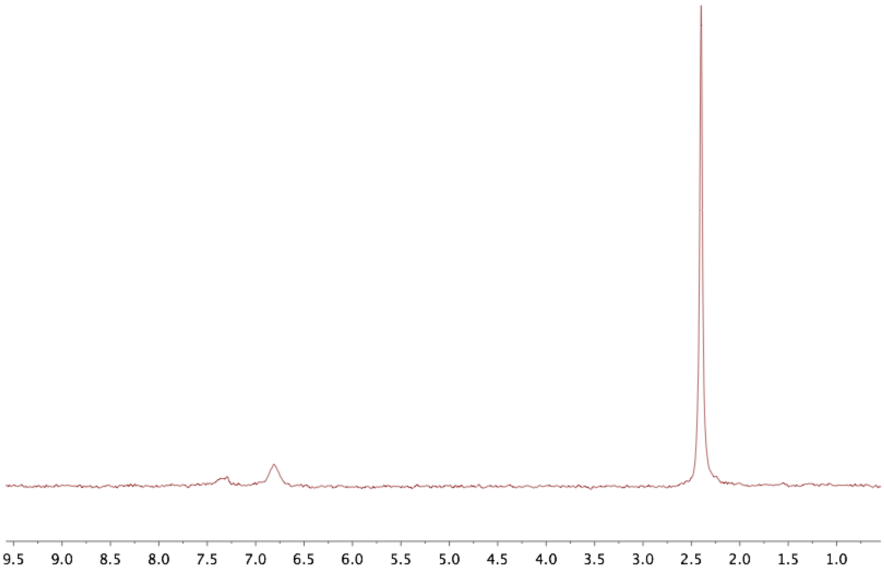

Figure S49. ^{2}D NMR ($CHCl_3$, 61.49MHz) spectrum of the deuteration of P(*o*-tolyl)$_3$ (**4**) using **Rh@NHC** after 48h at 55°C.

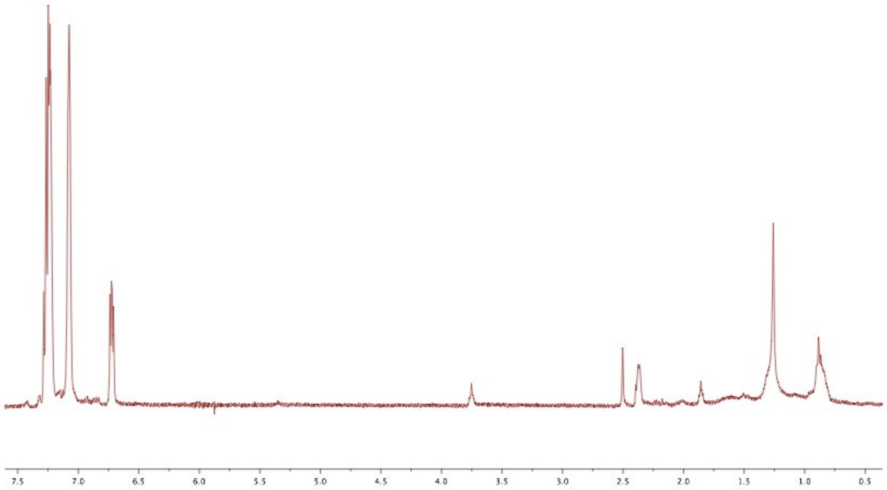

Figure S50. ^{1}H NMR ($CHCl_3$, 400MHz) spectrum of the deuteration of P(*o*-tolyl)$_3$ (**4**) using **Rh@NHC** after 12d at 55°C.

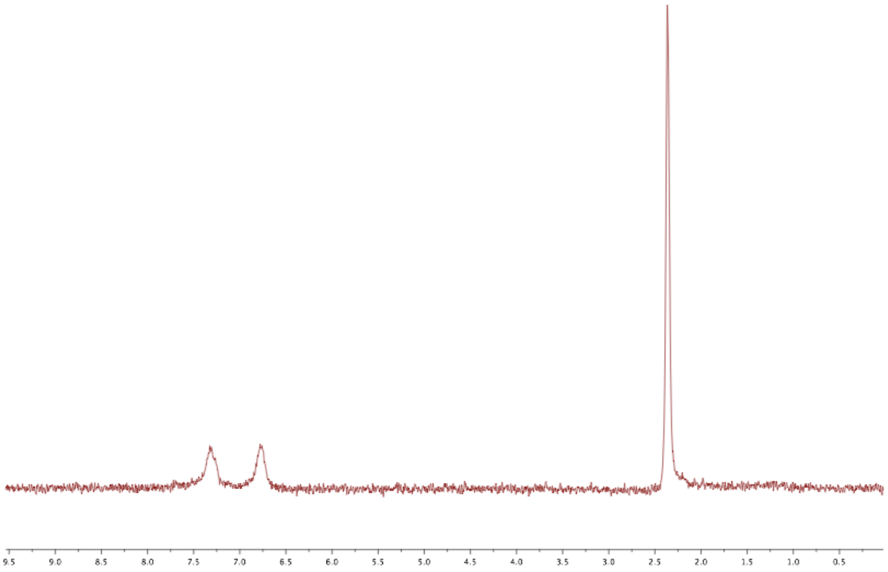

Figure S51. ^{2}D NMR ($CHCl_3$, 61.49MHz) spectrum of the deuteration of P(*o*-tolyl)$_3$ (**4**) using **Rh@NHC** after 12d at 55°C.

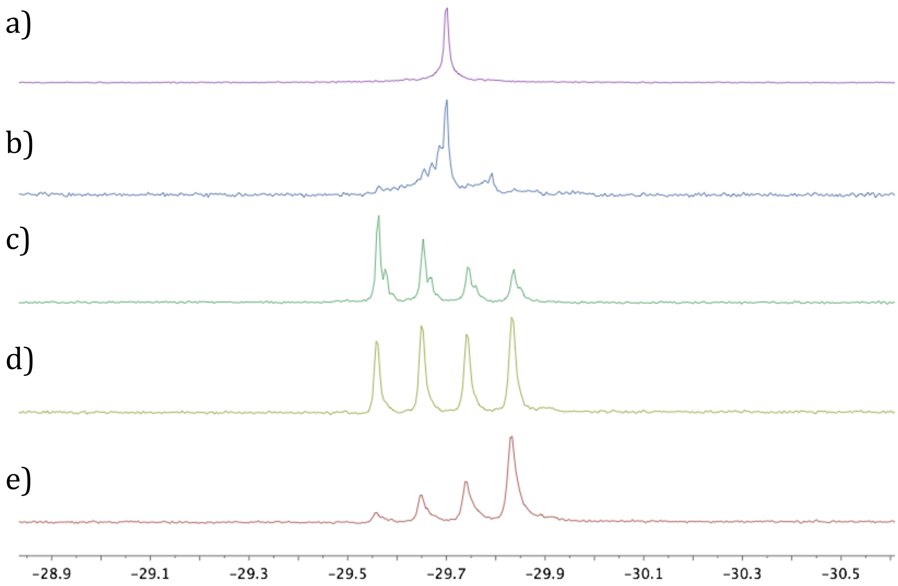

Figure S52. $^{31}P\{^1H\}$ NMR ($CDCl_3$, 162MHz) spectra of P(*o*-tolyl)$_3$ (**4**) deuteration evolution using **Rh@NHC**: (a) t=0 (b) 7h, (c) 2 days, (d) 8 days and (e) 12 days at 55°C.

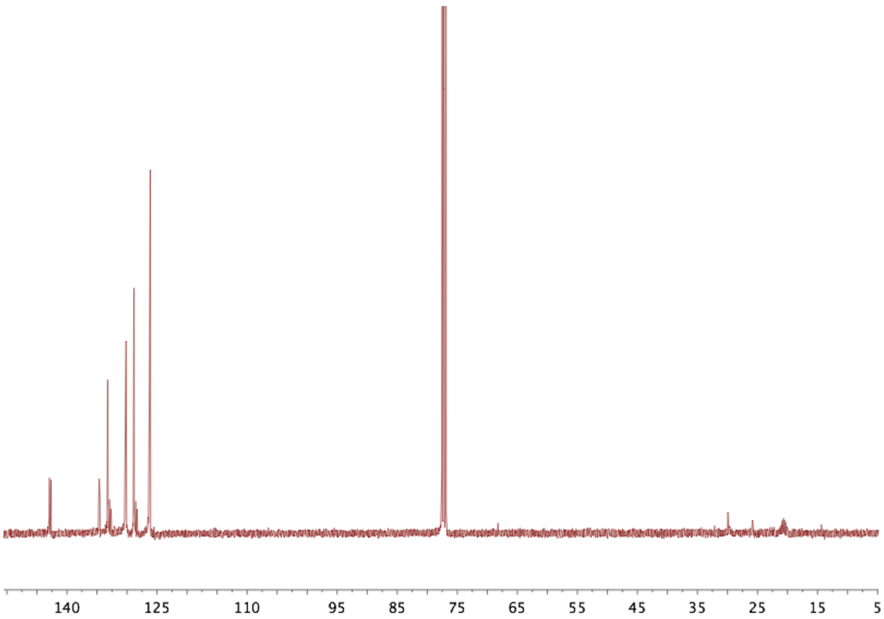

Figure S53. $^{13}C\{^1H\}$ NMR ($CDCl_3$, 100.6MHz) spectrum of the deuteration of P(*o*-tolyl)$_3$ (**4**) using **Rh@NHC** after 8 days at 55°C.

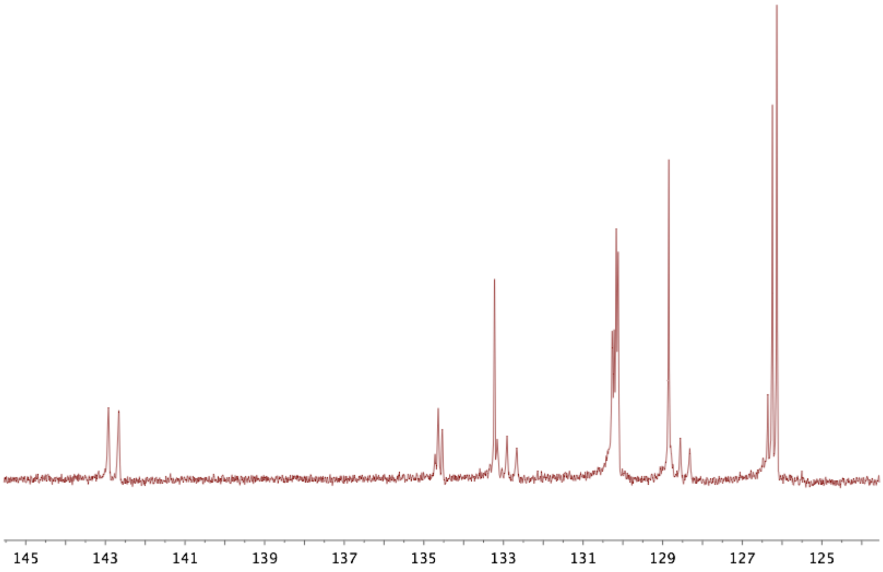

Figure S54. $^{13}C\{^1H\}$ NMR ($CDCl_3$, 100.6MHz) spectrum of the deuteration of P(*o*-tolyl)$_3$ (**4**) using **Rh@NHC** after 8 days at 55°C.

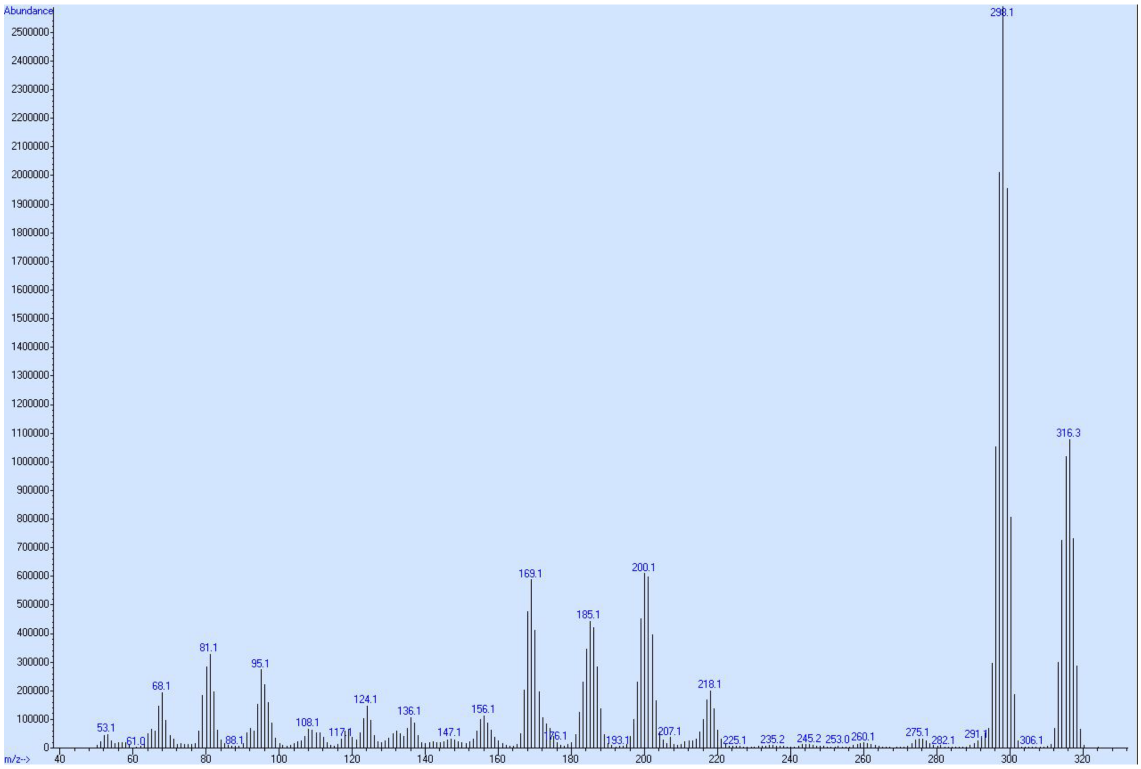

Figure S55. Mass spectrum of the deuteration of P(*o*-tolyl)$_3$ (**4**) using **Rh@NHC** after 12 days at 55°C.

4.5. H/D exchange of H_3B-P(*o*-tolyl)$_3$ (5)

4.5.1. Using **Rh@NHC**:

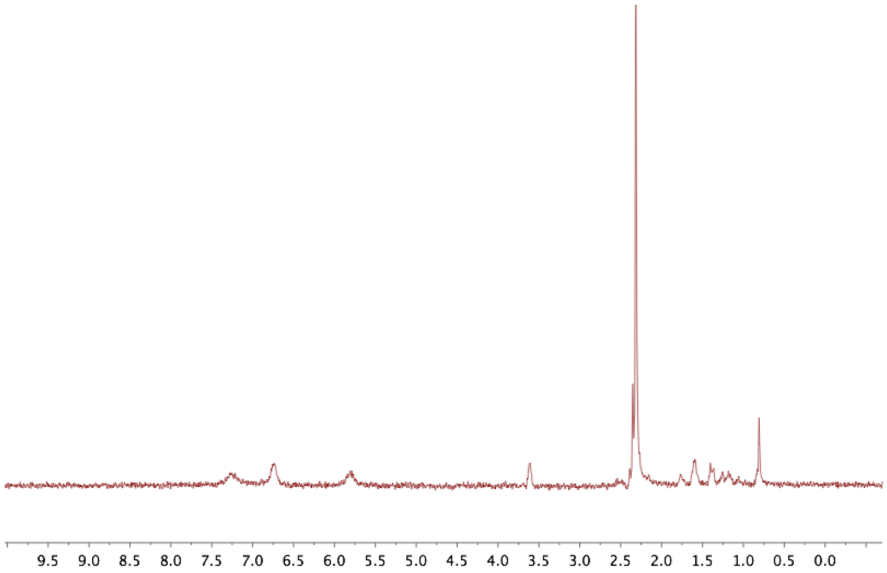

Figure S56. ^{2}D NMR ($CHCl_3$, 61.49MHz) spectrum of the deuteration of H_3B- P(*o*-tolyl)$_3$ (**5**) using **Rh@NHC** after 48h at 55°C.

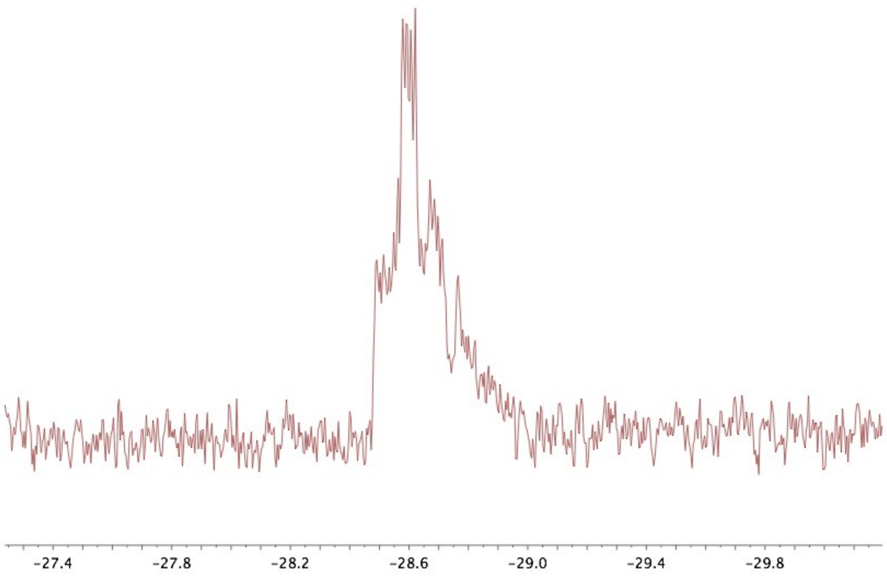

Figure S57. $^{31}P\{^1H\}$ NMR ($CDCl_3$, 162MHz) spectrum of deuteration of H_3B-P(*o*-tolyl)$_3$ (**5**) using **Rh@NHC** after 48h at 55°C.

4.6. H/D exchange of O=PPh$_3$ (6):

4.6.1. Using **Ru@PVP**:

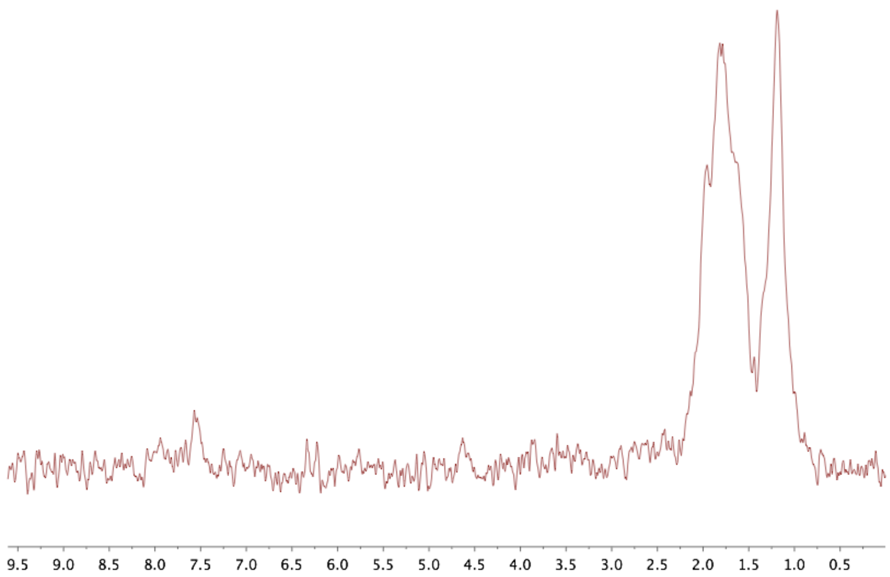

Figure S58. ^{2}D NMR ($CHCl_3$, 61.49MHz) spectrum of the deuteration of O=PPh$_3$ (**6**) using **Ru@PVP** after 36h at 55°C.

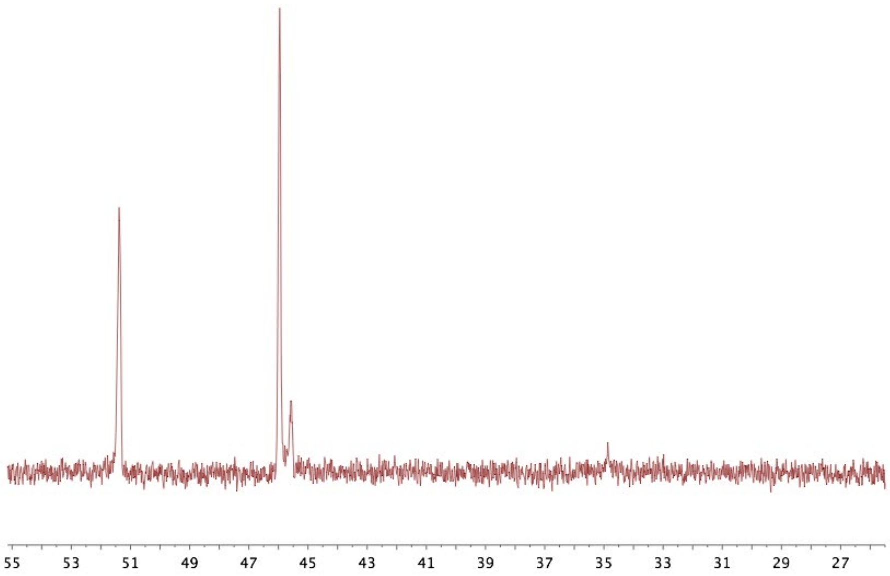

Figure S59. ^{31}P{^{1}H} NMR ($CDCl_3$, 162MHz) spectra of the deuteration of O=PPh$_3$ (**6**) using **Ru@PVP** after 36h at 55°C.

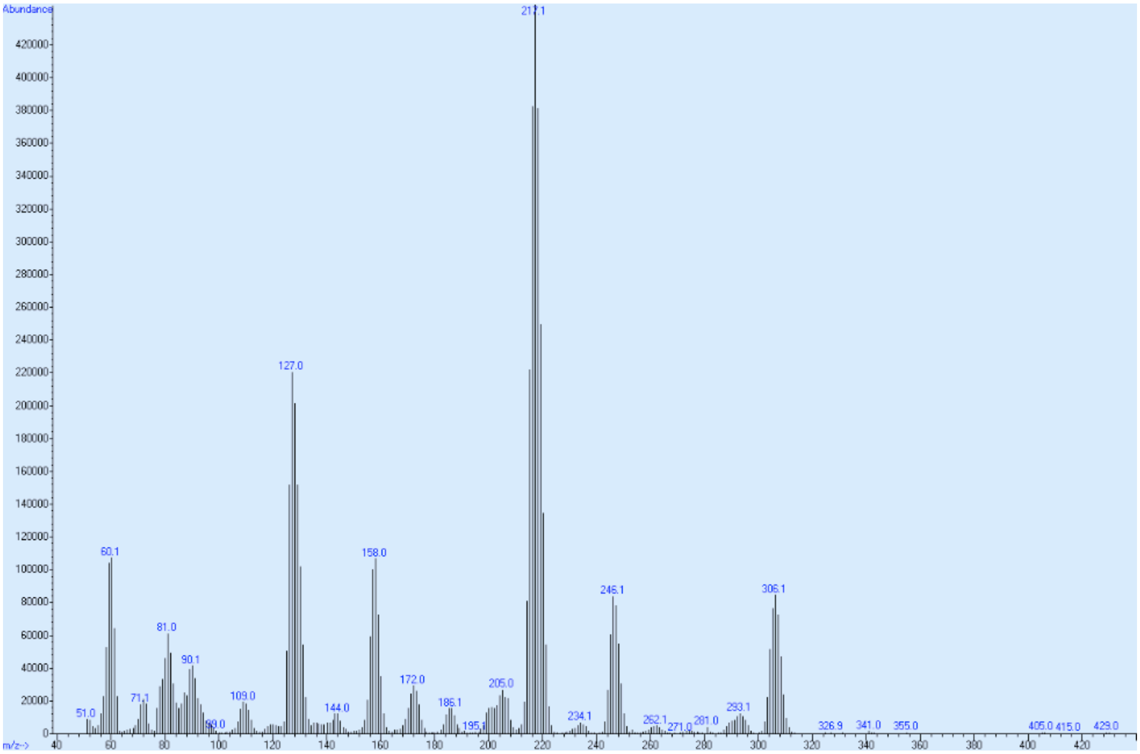

Figure S60. Mass spectrum of the deuteration of $O=PPh_3$ (**6**) using **Ru@PVP** after 36h at 55°C.

4.6.2. Using **Ru@NHC**:

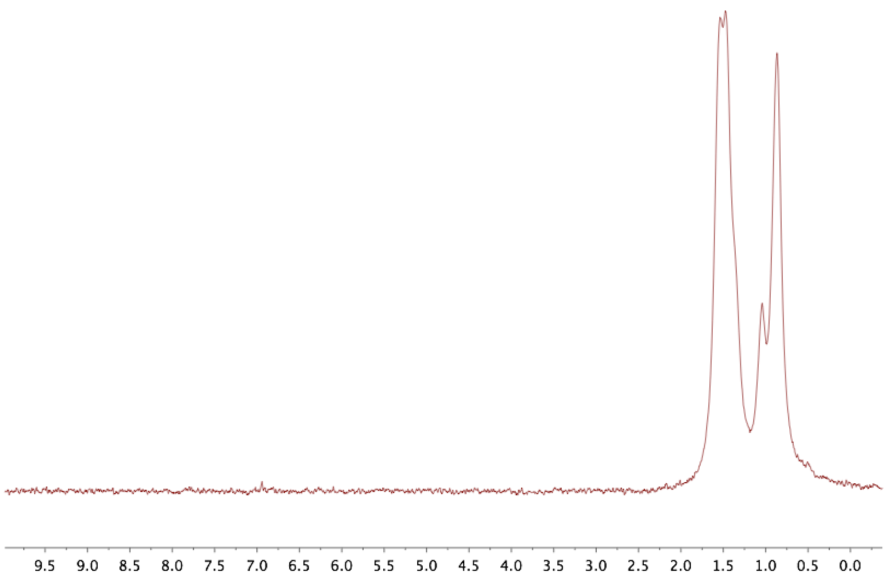

Figure S61. ^{2}D NMR ($CHCl_3$, 61.49MHz) spectrum of the deuteration of O=PPh_3 (**6**) using **Ru@NHC** after 48h at 55°C.

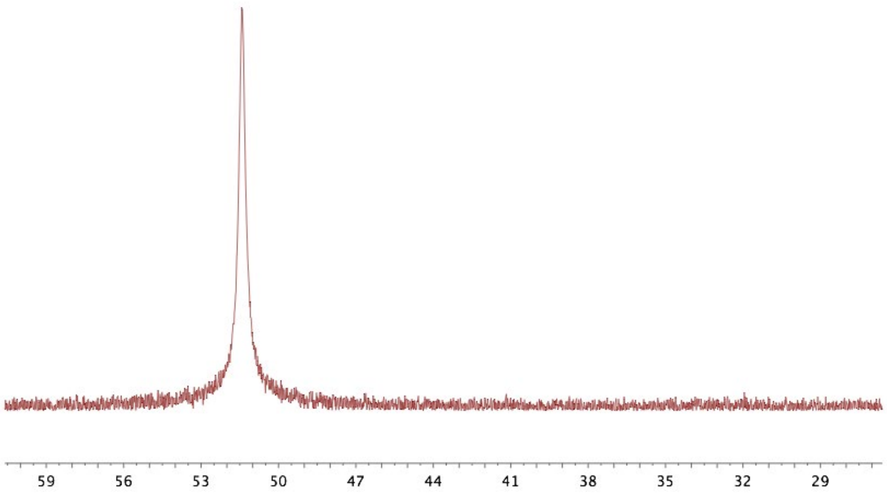

Figure S62. ^{31}P{^{1}H} NMR ($CDCl_3$, 162MHz) spectra of the deuteration of O=PPh_3 (**6**) using **Ru@NHC** after 48h at 55°C.

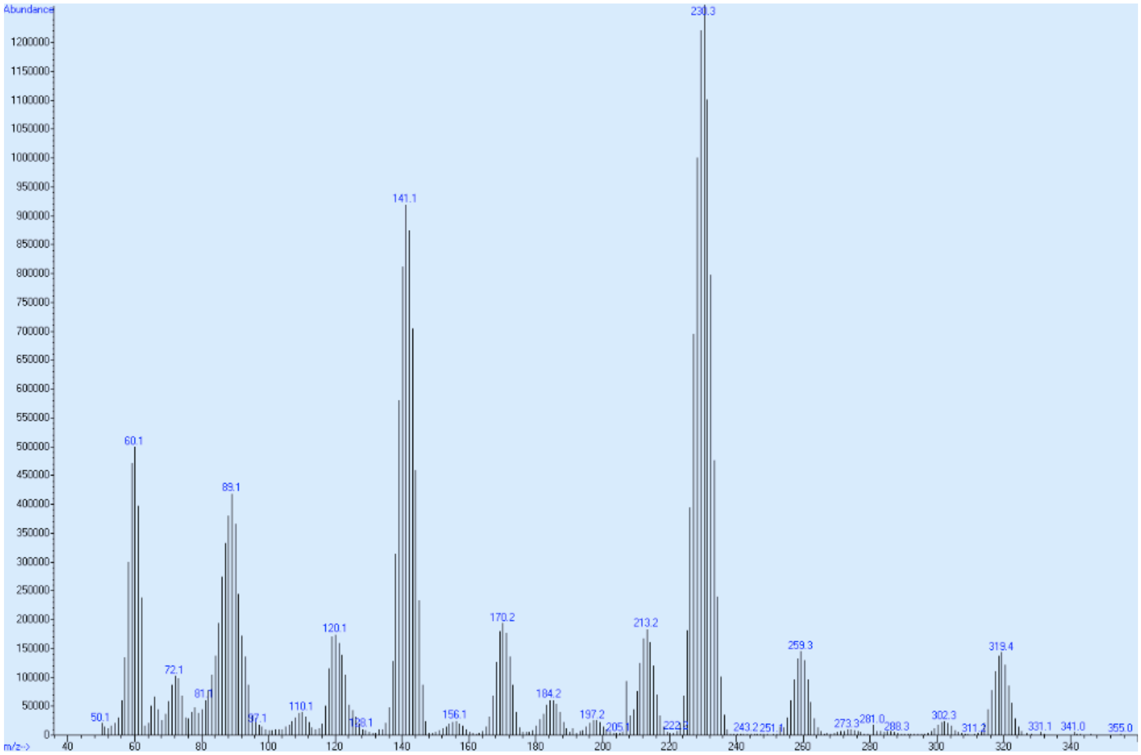

Figure S63. Mass spectrum of the deuteration of O=PPh$_3$ (**6**) using **Ru@NHC** after 48h at 55°C.

4.6.3. Using **Rh@NHC**:

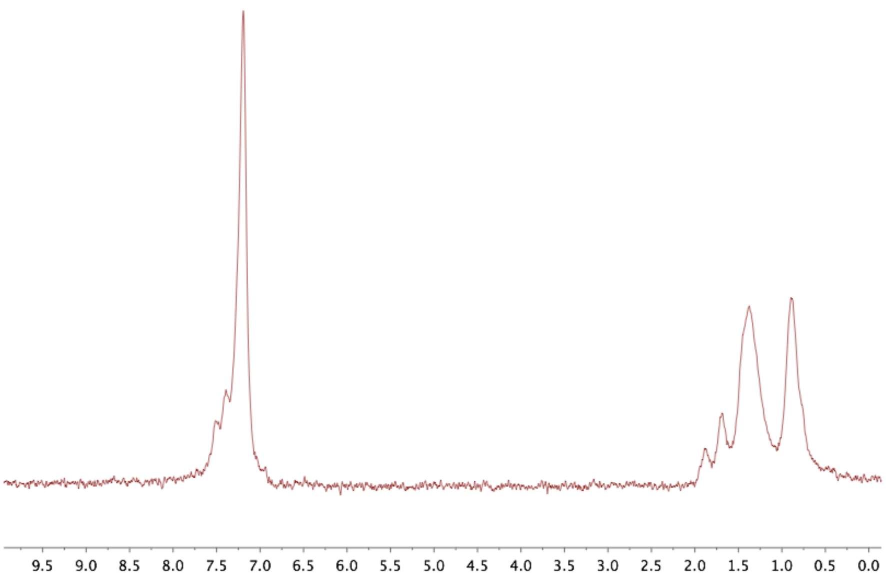

Figure S64. ^{2}D NMR ($CHCl_3$, 61.49MHz) spectrum of the deuteration of O=PPh_3 (**6**) using **Rh@NHC** after 48h at 55°C.

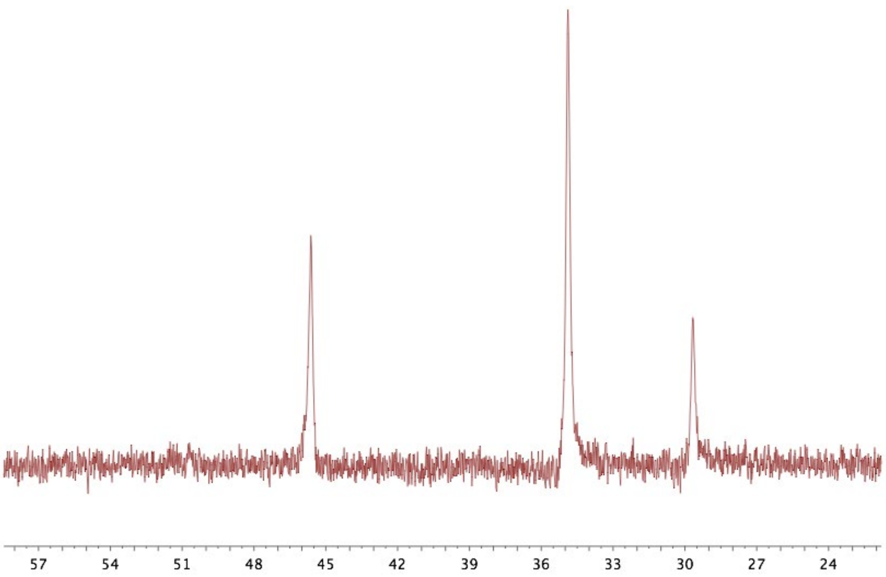

Figure S65. $^{31}P\{^{1}H\}$ NMR ($CDCl_3$, 162MHz) spectra of the deuteration of O=PPh_3 (**6**) using **Rh@NHC** after 48h at 55°C.

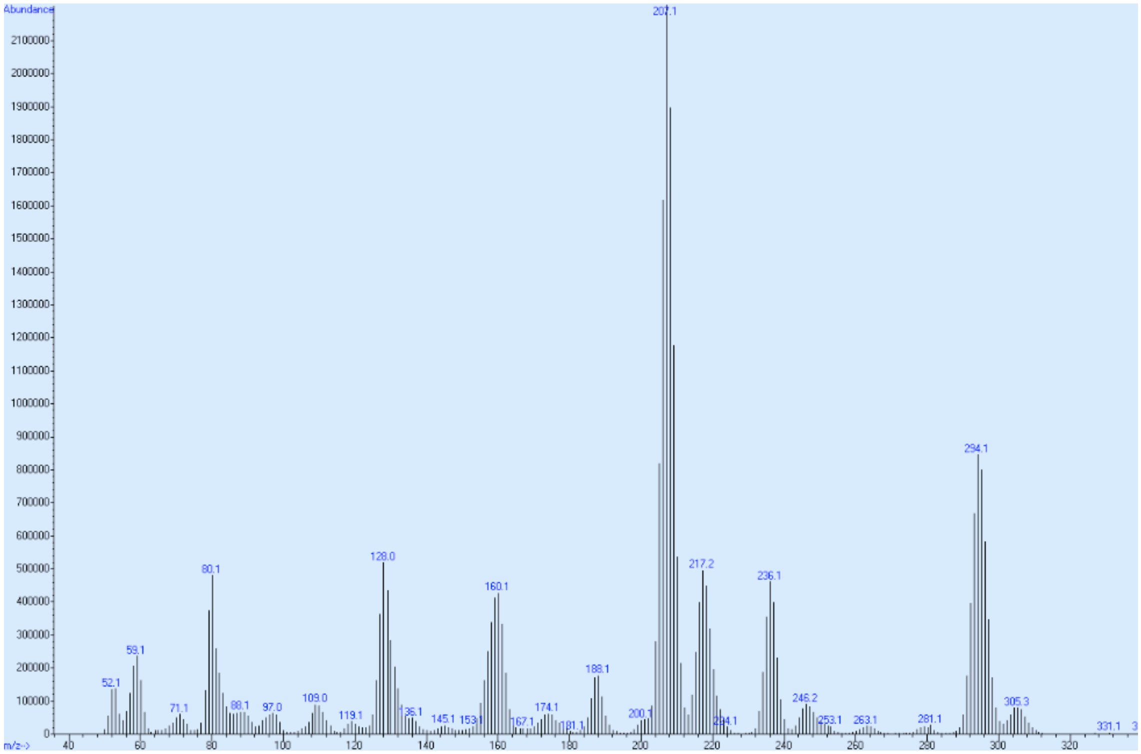

Figure S66. Mass spectrum of deuteration of O=PPh_3 (**6**) using **Rh@NHC** after 48h at 55°C.

4.7. H/D exchange of O=P(*o*-tolyl)$_3$ (7):

4.7.1. Using **Rh@NHC**:

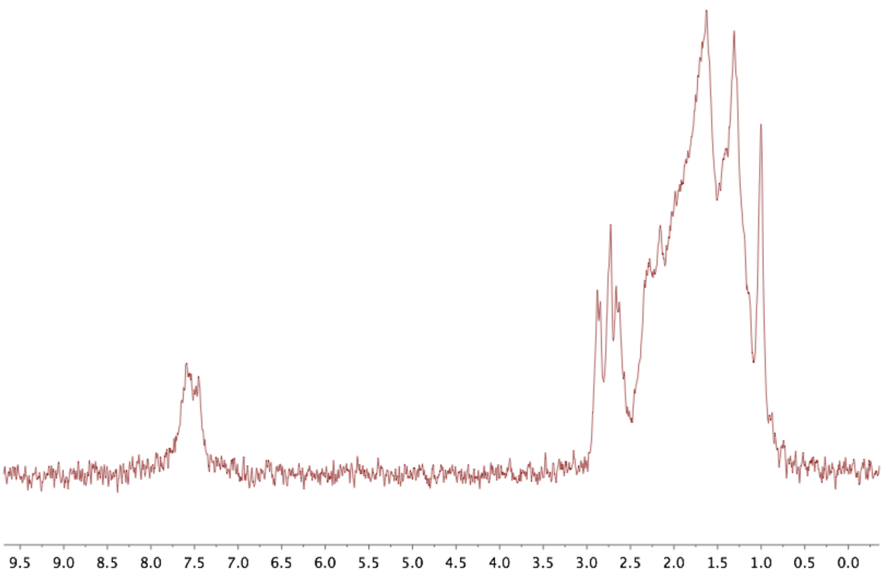

Figure S67. ^{2}D NMR ($CHCl_3$, 61.49MHz) spectrum of the deuteration of O=P(*o*-tolyl)$_3$ (**7**) using **Rh@NHC** after 48h at 55°C.

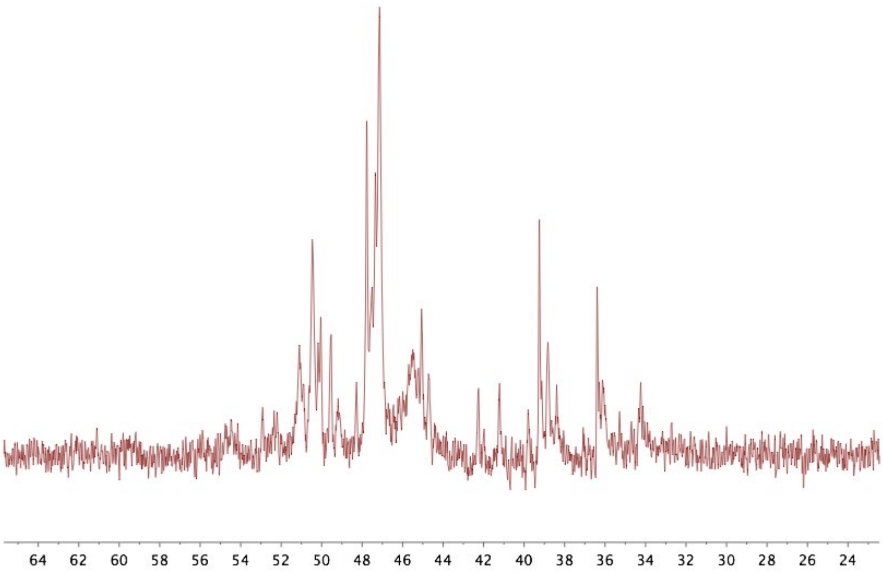

Figure S68. ^{31}P{^{1}H} NMR ($CDCl_3$, 162MHz) spectra of the deuteration of O=P(*o*-tolyl)$_3$ (**7**) using **Rh@NHC** after 48h at 55°C.

4.8.1. H/D exchange of dppm (11):

4.8.1. Using **Rh@NHC**:

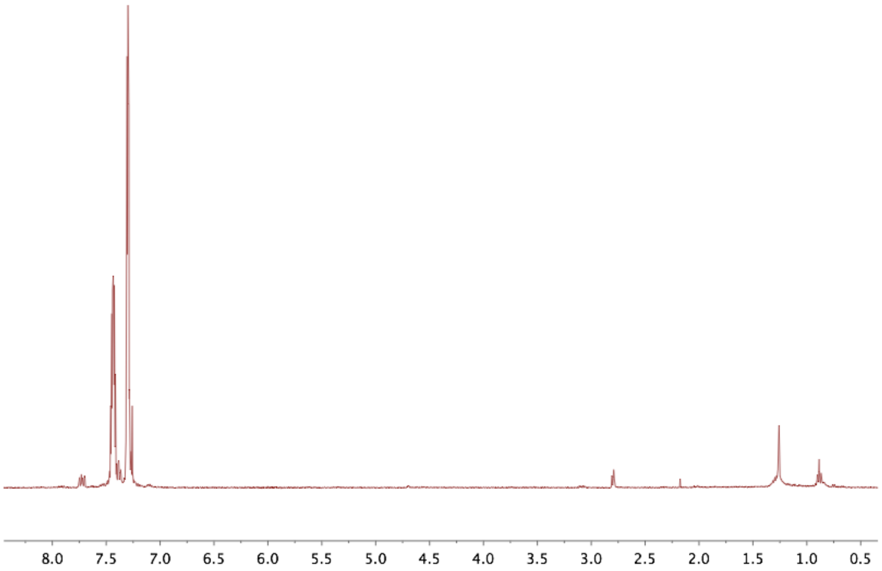

Figure S69. ^{1}H NMR ($CDCl_3$, 400MHz) spectrum of the deuteration of dppm (**11**) using **Rh@NHC** after 48h at 55°C.

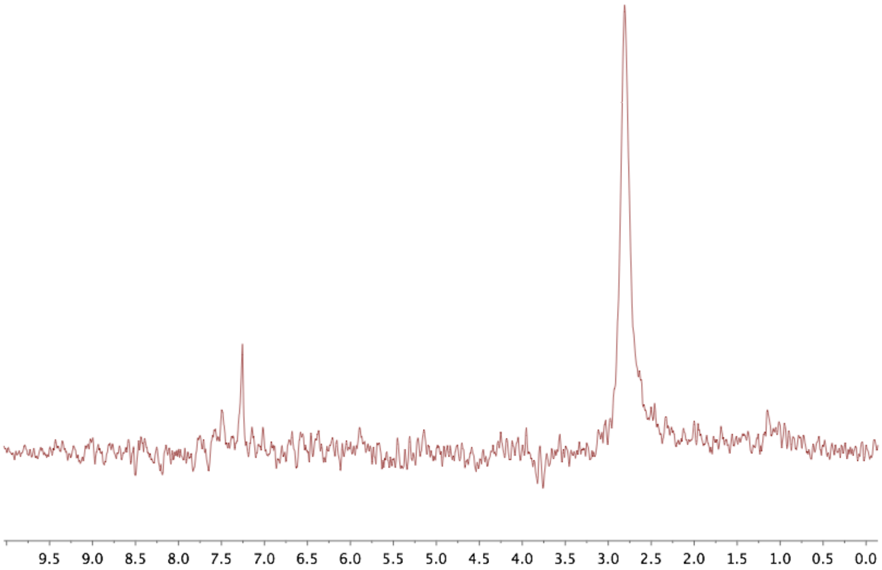

Figure S70. ^{2}D NMR ($CHCl_3$, 61.49MHz) spectrum of the deuteration of dppm (**11**) using **Rh@NHC** after 48h at 55°C.

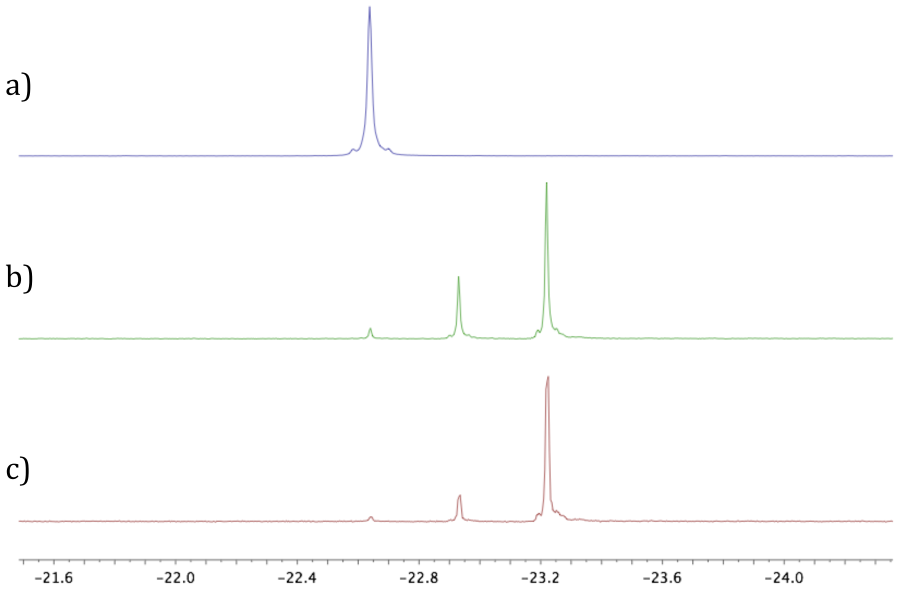

Figure S71. $^{31}P\{^{1}H\}$ NMR ($CDCl_3$, 162MHz) spectra of dppm (**11**) deuteration evolution using **Rh@NHC**: (a) t=0, (b) 48h and (c) 8 days at 55°C.

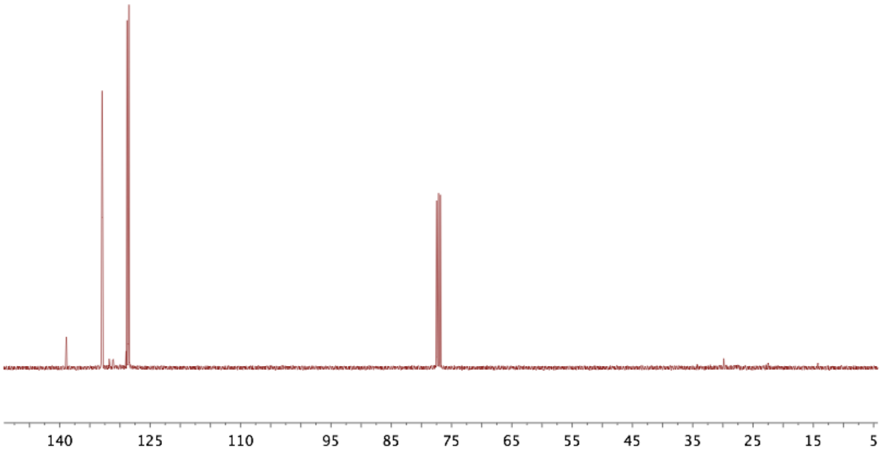

Figure S72. $^{13}C\{^{1}H\}$ NMR ($CDCl_3$, 100.6MHz) spectrum of the deuteration of dppm (**11**) using **Rh@NHC** after 48h at 55°C.

4.8.2. Using **Rh/C**:

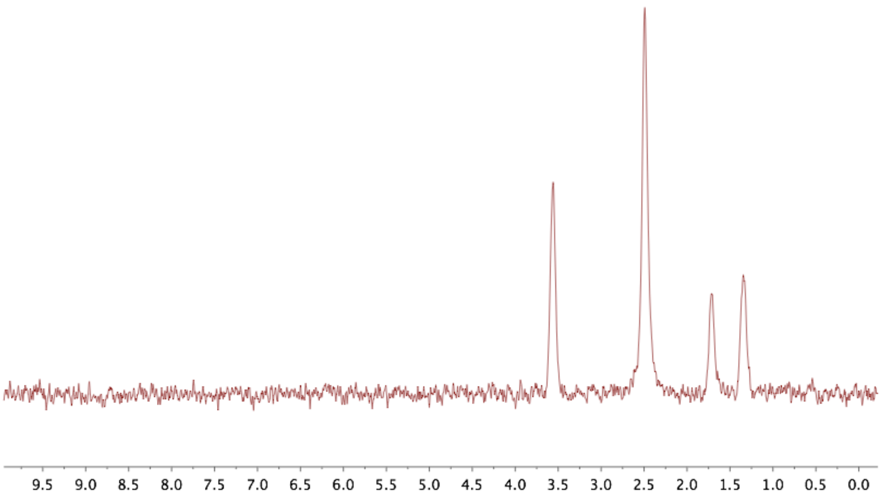

Figure S73. ^{2}D NMR ($CHCl_3$, 61.49MHz) spectrum of the deuteration of dppm (**11**) using **Rh/C** after 48h at 55°C.

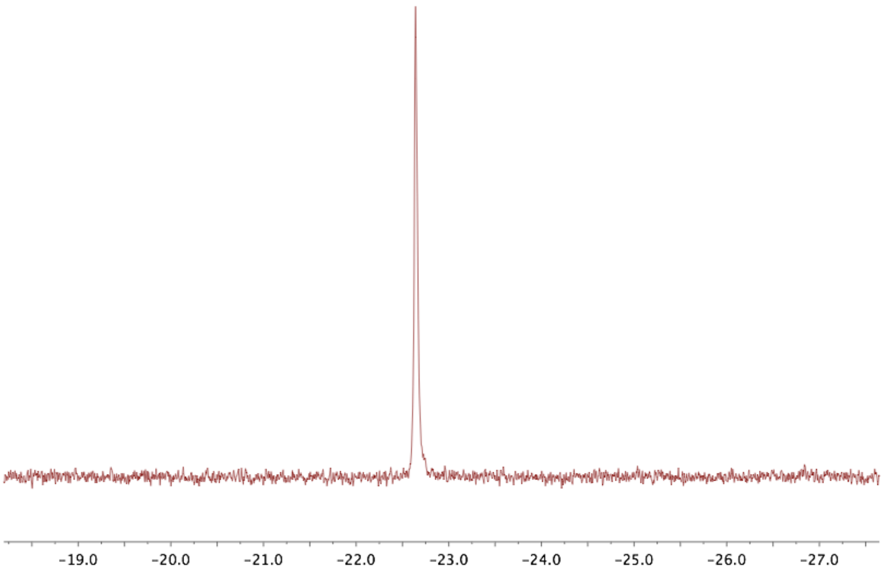

Figure S74. $^{31}P\{^{1}H\}$ NMR ($CDCl_3$, 162MHz) spectrum of the deuteration of dppm (**11**) using **Rh/C** after 48h at 55°C.

4.9. H/D exchange of dppb (12):

4.9.1. Using **Ru@PVP**:

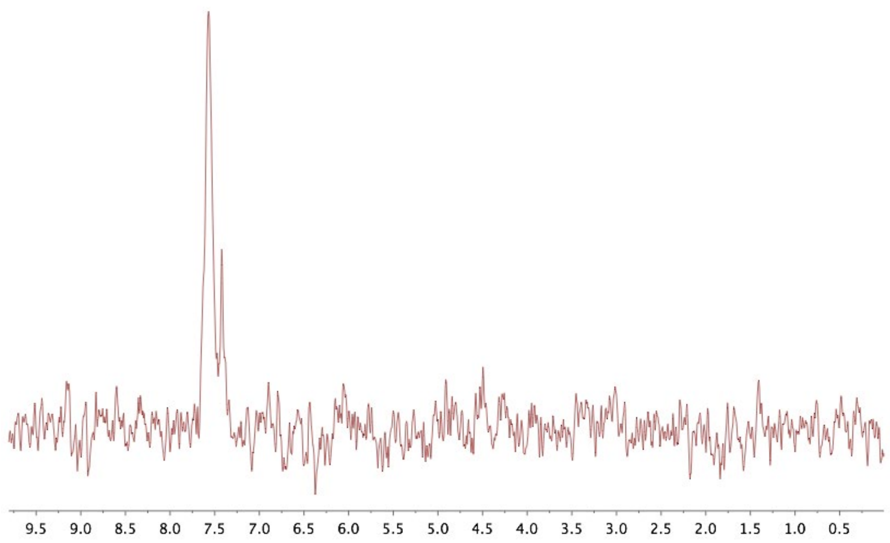

Figure S75. ^{2}D NMR ($CHCl_3$, 61.49MHz) spectrum of the deuteration of dppb (**12**) using **Ru@PVP** after 48h at 80°C.

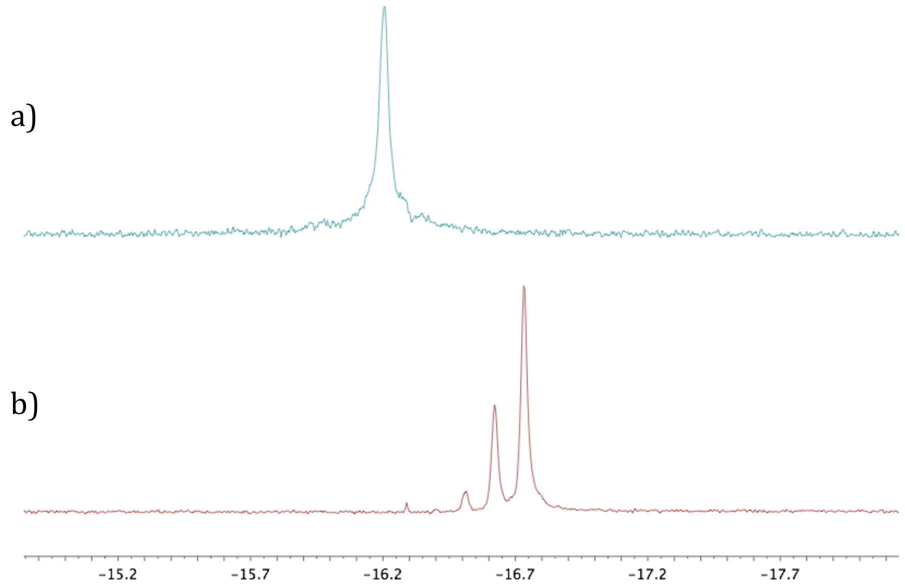

Figure S76. $^{31}P\{^{1}H\}$ NMR ($CDCl_3$, 162MHz) spectra of dppb (**12**) deuteration evolution using **Ru@PVP**: (a) t=0 and (b) 48h at 80°C.

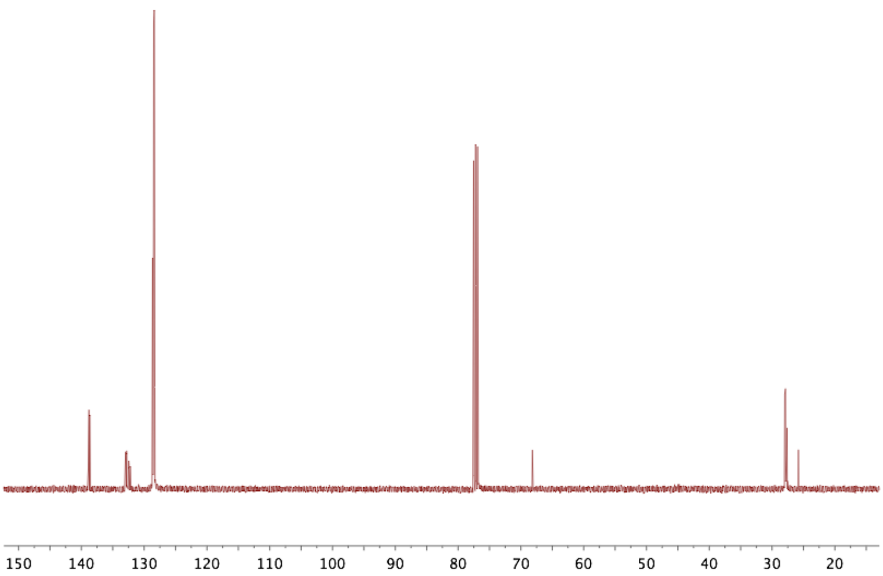

Figure S77. $^{13}C\{^{1}H\}$ NMR ($CDCl_3$, 100.6MHz) spectrum of the deuteration of dppb (**12**) using **Ru@PVP** after 48h at 80°C.

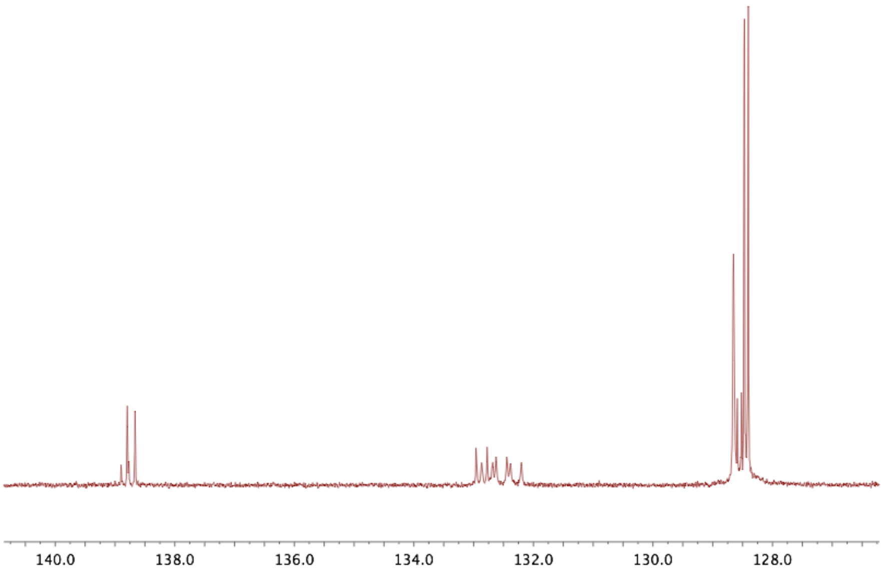

Figure S78. $^{13}C\{^{1}H\}$ NMR ($CDCl_3$, 100.6MHz) spectrum of the deuteration of dppb (**12**) using **Ru@PVP** after 48h at 80°C.

3.9.2. Using **Ru@NHC**:

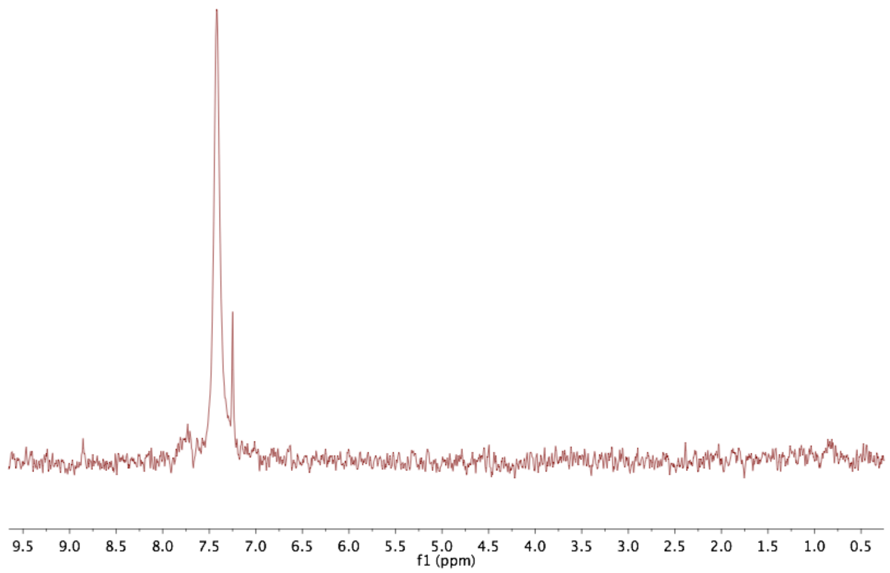

Figure S79. ^{2}D NMR ($CHCl_3$, 61.49MHz) spectrum of the deuteration of dppb (**12**) using **Ru@NHC** after 48h at 55°C.

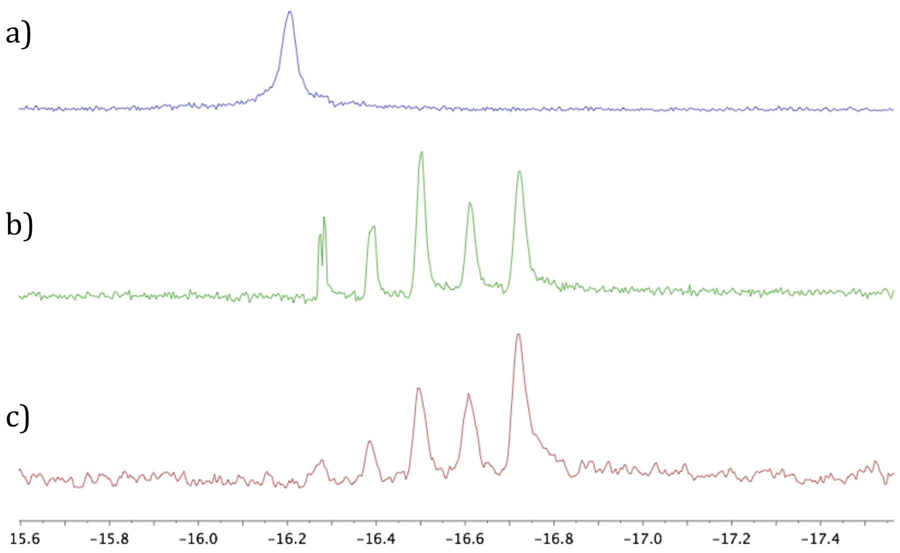

Figure S80. $^{31}P\{^{1}H\}$ NMR ($CDCl_3$, 162MHz) spectra of dppb (**12**) deuteration evolution using **Ru@NHC**: (a) t=0 and (b) 48h and (c) 8 days at 55°C.

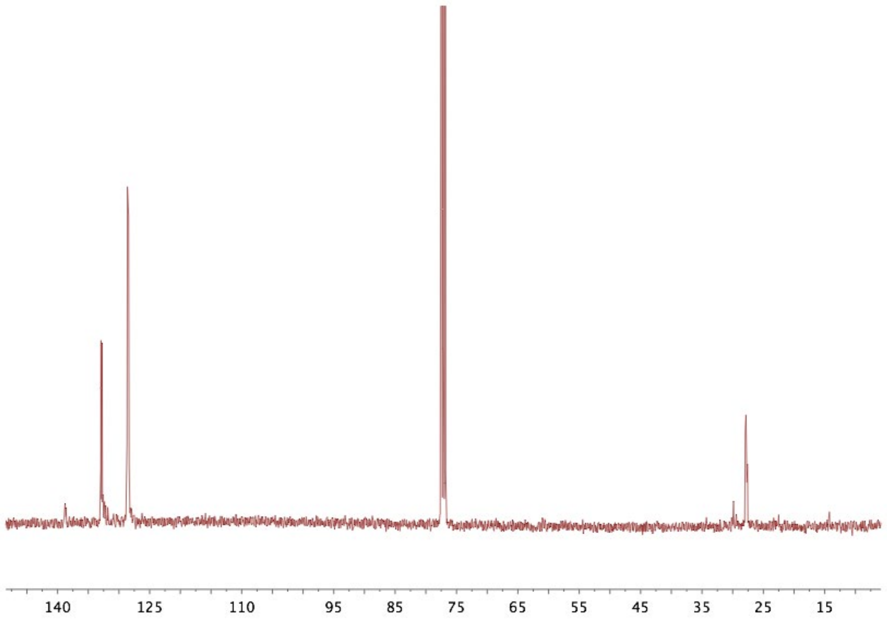

Figure S81. $^{13}C\{^{1}H\}$ NMR ($CDCl_3$, 100.6MHz) spectrum of the deuteration of dppb (**12**) using **Ru@NHC** after 48h at 55°C.

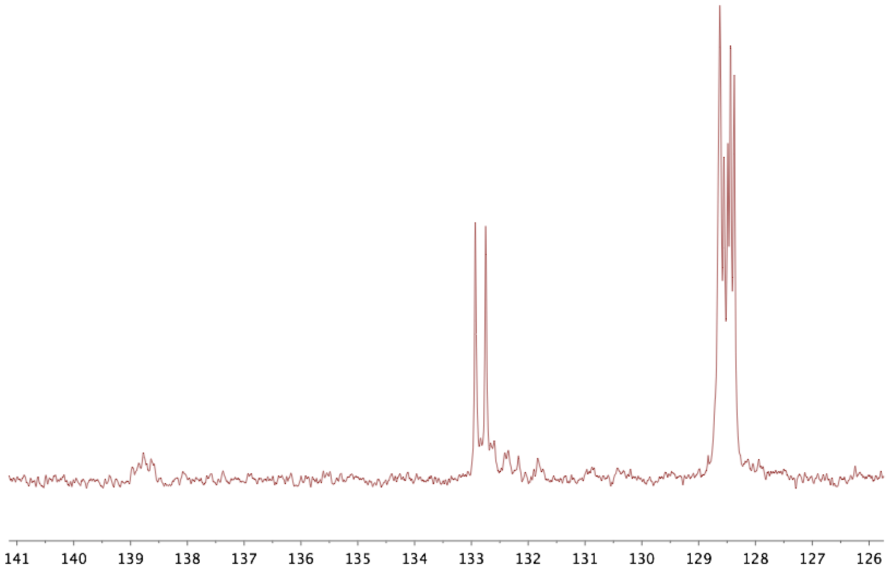

Figure S82. $^{13}C\{^{1}H\}$ NMR ($CDCl_3$, 100.6MHz) spectrum of the deuteration of dppb (**12**) using **Ru@NHC** after 48h at 55°C.

4.9.3. Using **Rh@NHC**:

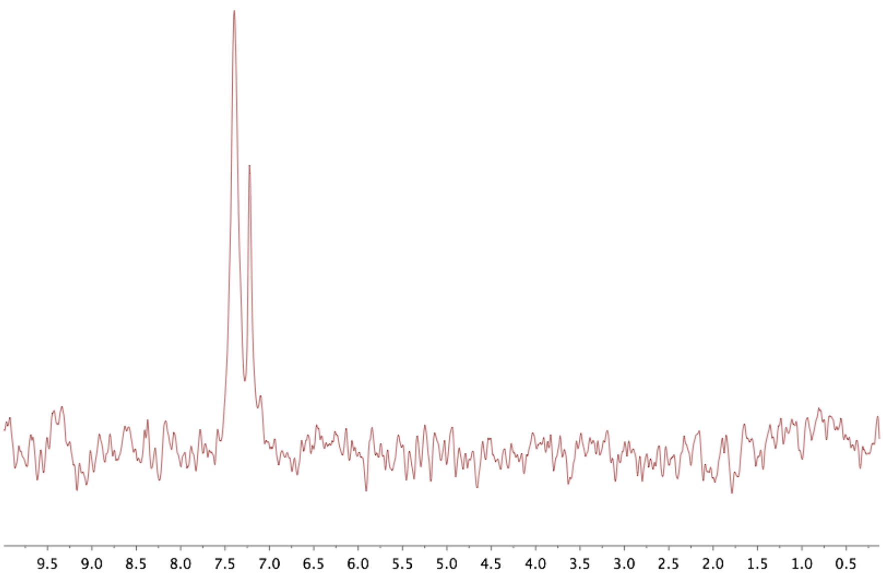

Figure S83. ^{2}D NMR ($CHCl_3$, 61.49MHz) spectrum of the deuteration of dppb (**12**) using **Rh@NHC** after 48h at 55°C.

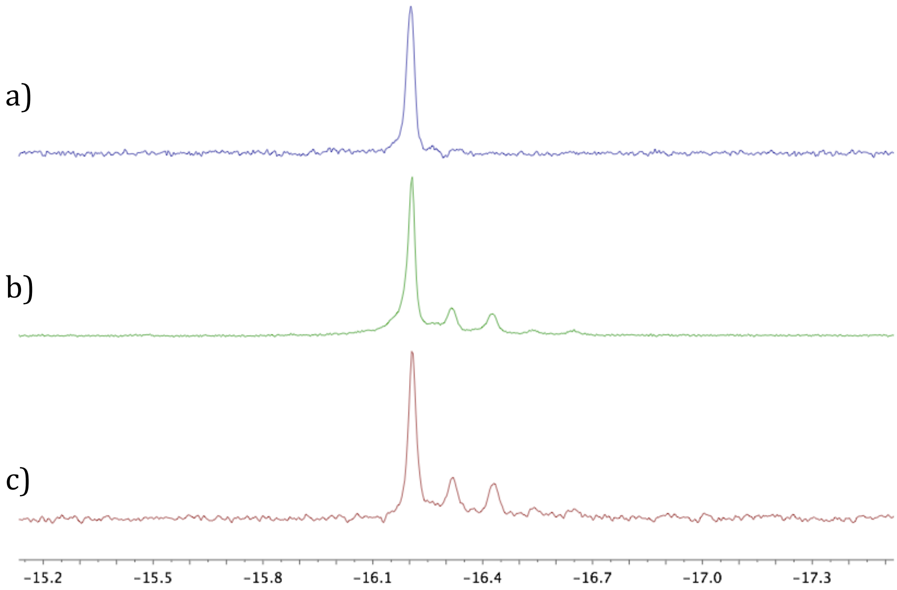

Figure S84. $^{31}P\{^{1}H\}$ NMR ($CDCl_3$, 162MHz) spectra of dppb (**12**) deuteration evolution using **Rh@NHC**: (a) t=0 and (b) 48h and (c) 8 days at 55°C.

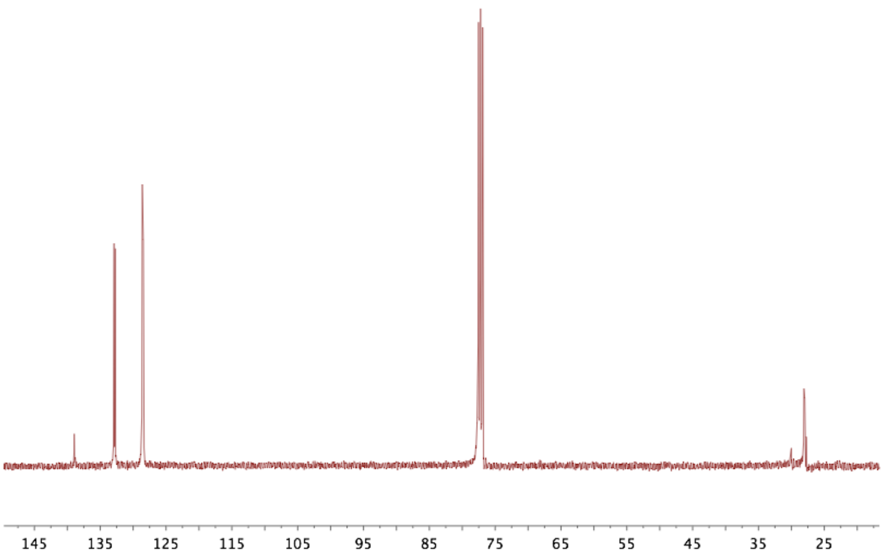

Figure S85. $^{13}C\{^{1}H\}$ NMR ($CDCl_3$, 100.6MHz) spectrum of the deuteration of dppb (**12**) using **Rh@NHC** after 48h at 55°C.

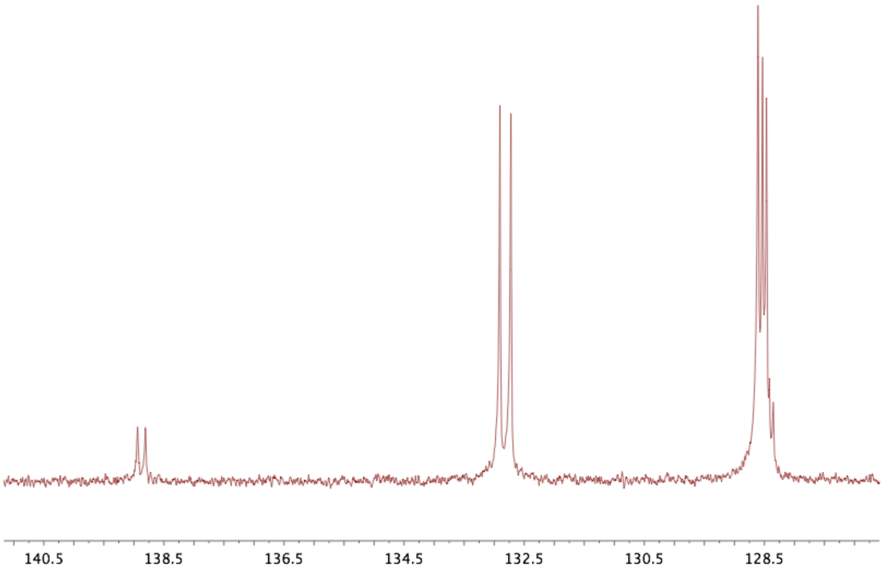

Figure S86. $^{13}C\{^{1}H\}$ NMR ($CDCl_3$, 100.6MHz) spectrum of the deuteration of dppb (**12**) using **Rh@NHC** after 48h at 55°C.

5. Quantification of the isomer ratio in deuteration experiments by ^{31}P NMR

5.1. Deuteration of PPh_3 (1)

<u>Data corresponding to Figure 2. Labels in Figures corresponds with that of Figure 2.</u>

e)

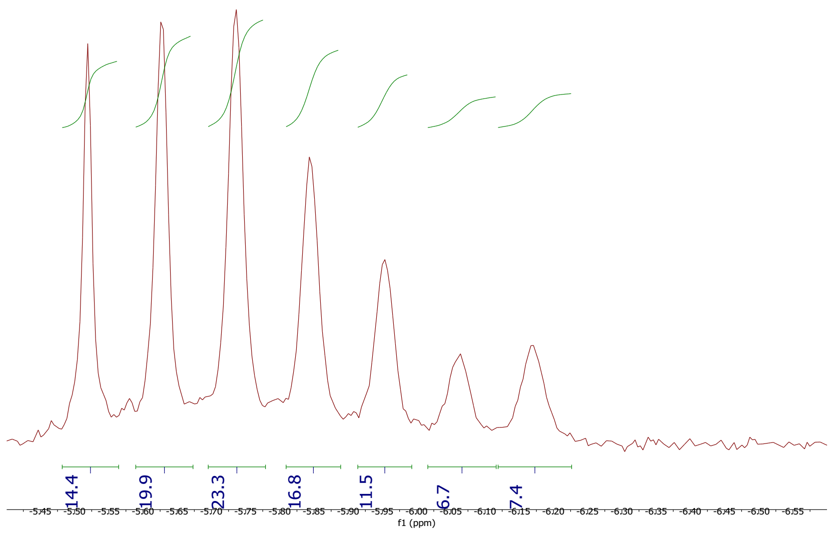

Figure S87. ^{31}P{1H} NMR spectra of deuteration of phosphine **1** after 48h of reaction under 2 bars of D_2 (D/H ratio= *ca*. 5) at 55°C using **Rh@NHC**.

d)

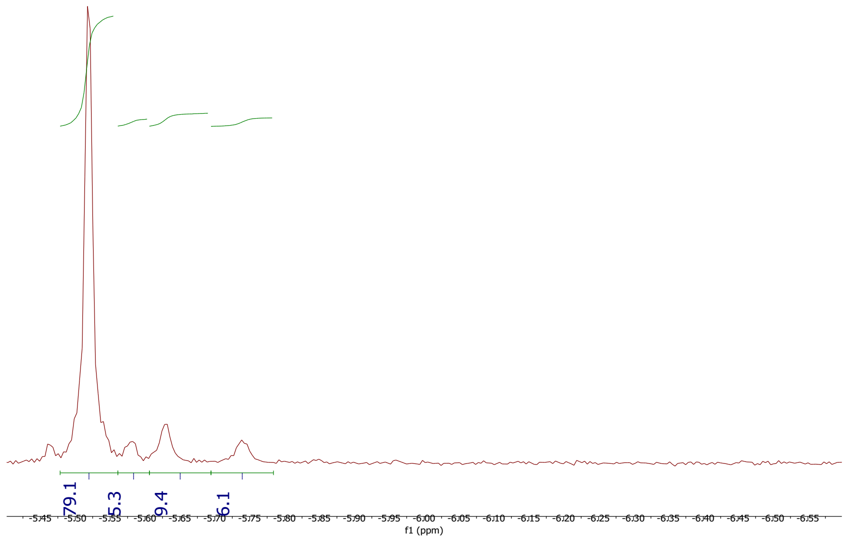

Figure S88. ^{31}P{1H} NMR spectra of deuteration of phosphine **1** after 48h of reaction under 2 bars of D_2 (D/H ratio= *ca*. 5) at 55°C using **Rh@PVP** .

c)

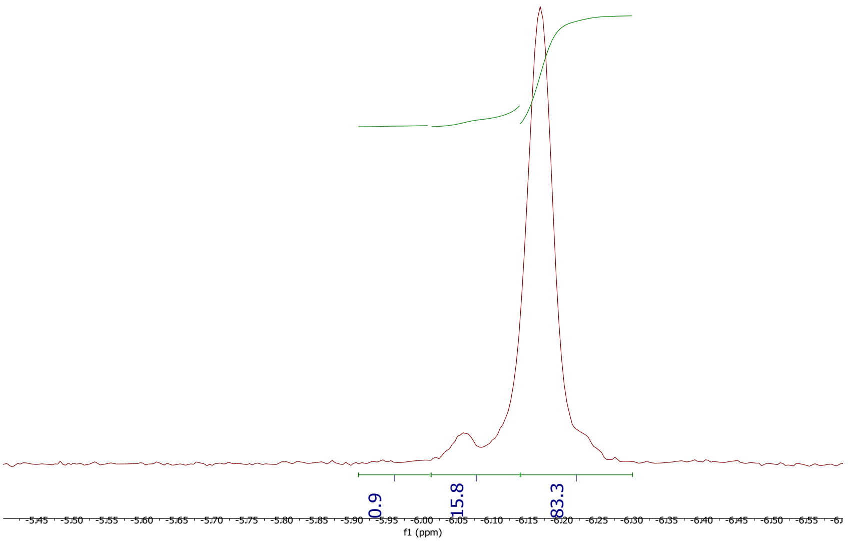

Figure S89. ^{31}P{1H} NMR spectra of deuteration of phosphine **1** after 48h of reaction under 2 bars of D_2 (D/H ratio= *ca*. 5) at 55°C using **Ru@NHC**.

b)

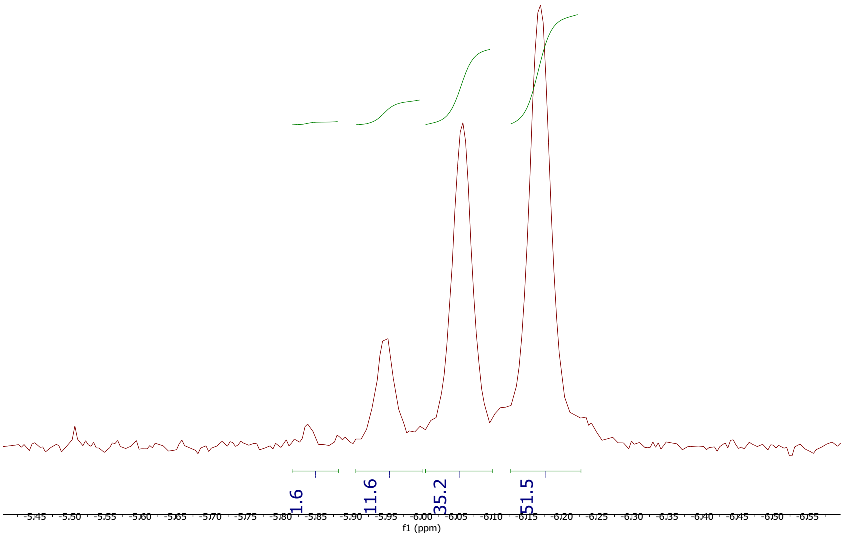

Figure S90. ^{31}P{1H} NMR spectra of deuteration of phosphine **1** after 48h of reaction under 2 bars of D_2 (D/H ratio= *ca*. 5) at 55°C using **Ru@PVP**.

a)

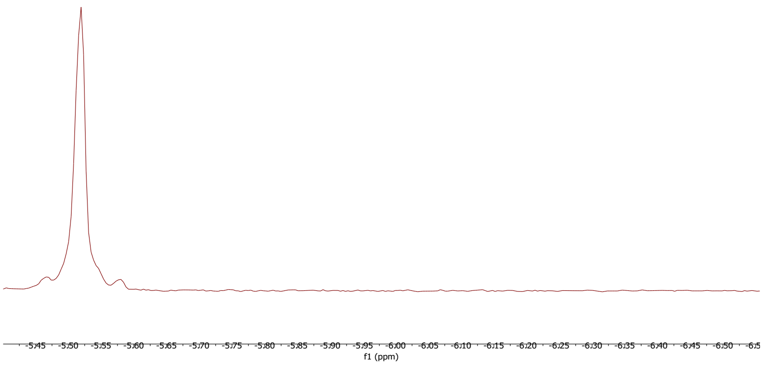

Figure S91. ^{31}P{1H} NMR spectra of phosphine **1.**

- Data corresponding to Table 1:

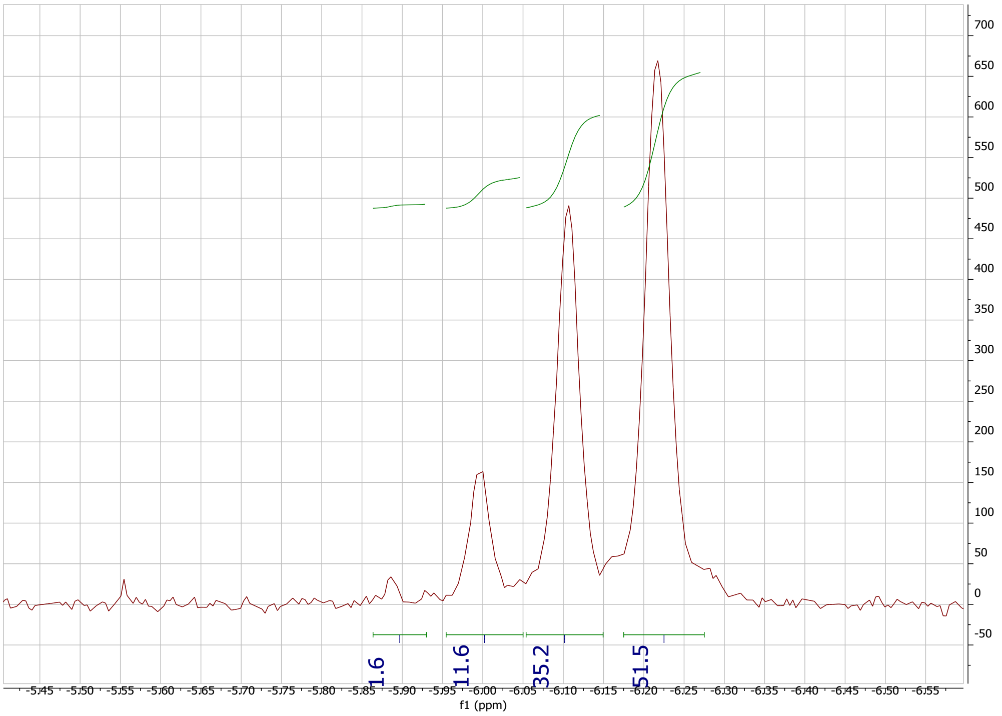

Figure S92. Spectrum corresponding to entry 1, deuteration of PPh_3 (**1**) using **Ru@PVP**.

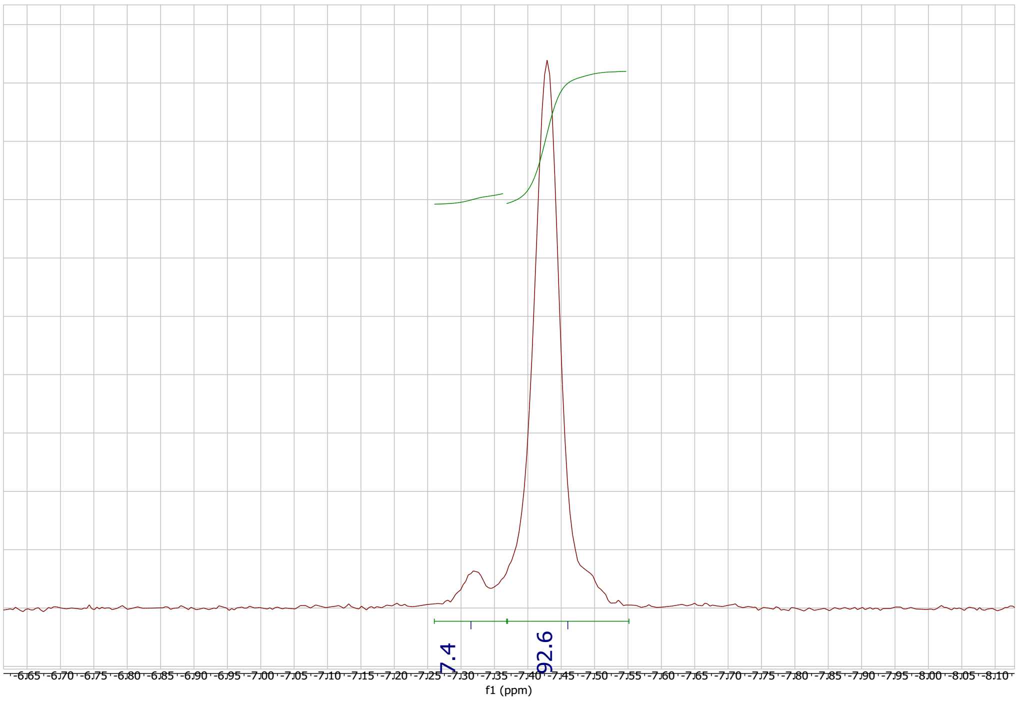

Figure S93. Spectrum corresponding to entry 2, deuteration of PPh_3 (**1**) using using **Ru@PVP**.

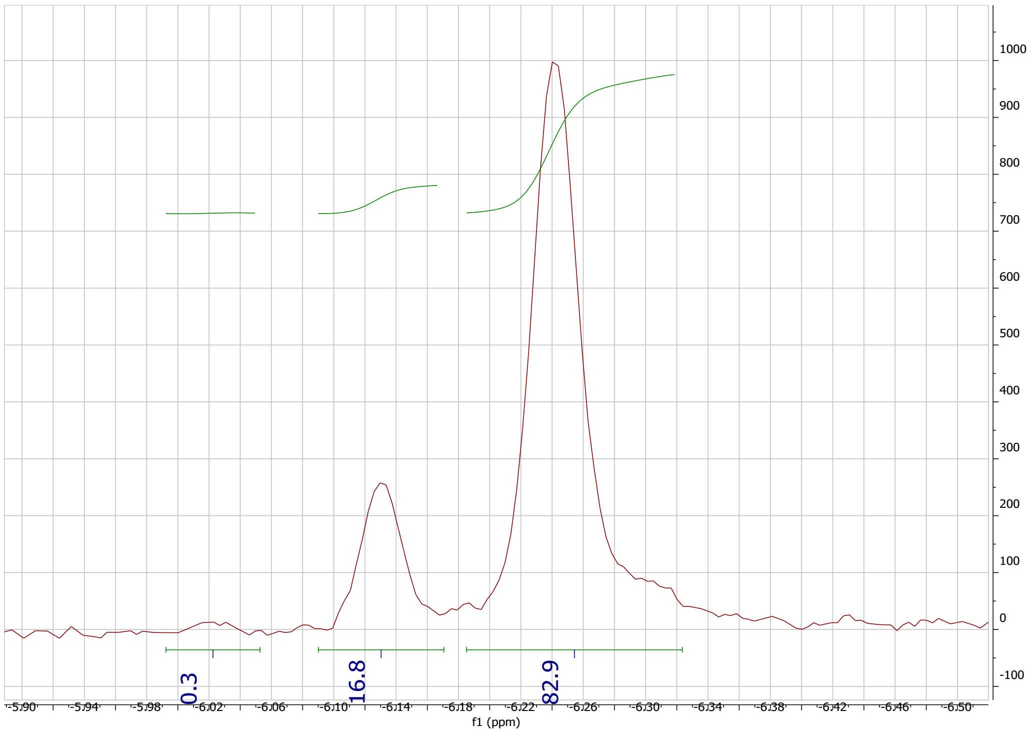

Figure S94. Spectrum corresponding to entry 4, deuteration of PPh_3 (**1**) using using **Ru@NHC**.

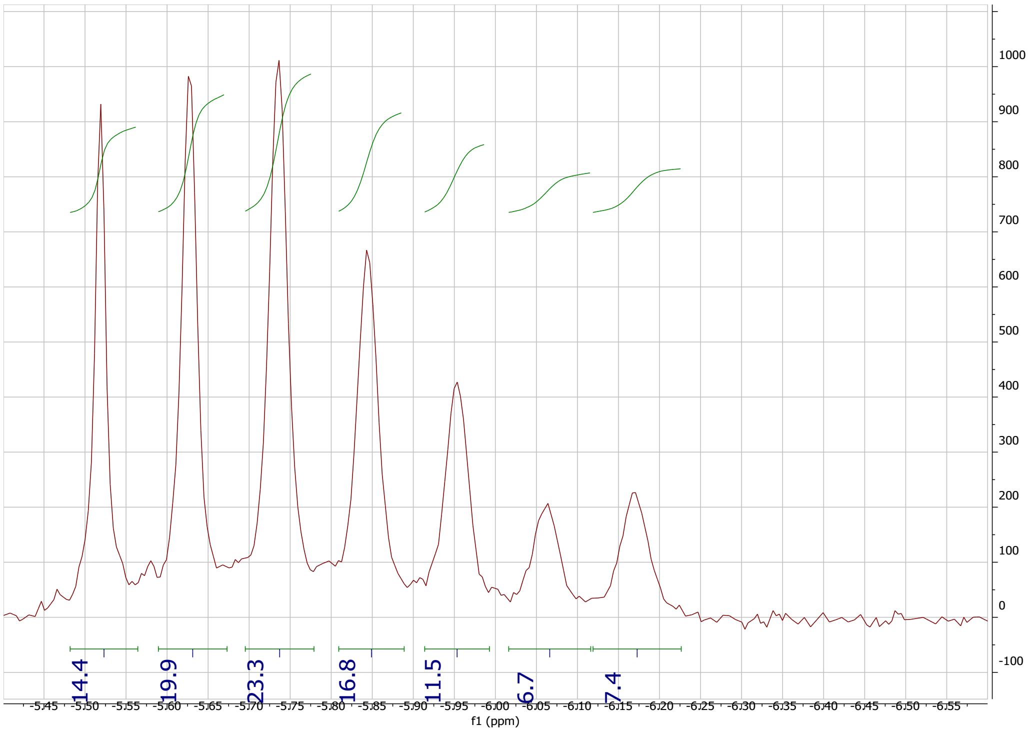

Figure S95. Spectrum corresponding to entry 5, deuteration of PPh_3 (**1**) using sing **Rh@PVP**.

5.2. Deuteration of P(*p*-tolyl)$_3$ (2)

<u>Data corresponding to Table 1</u>

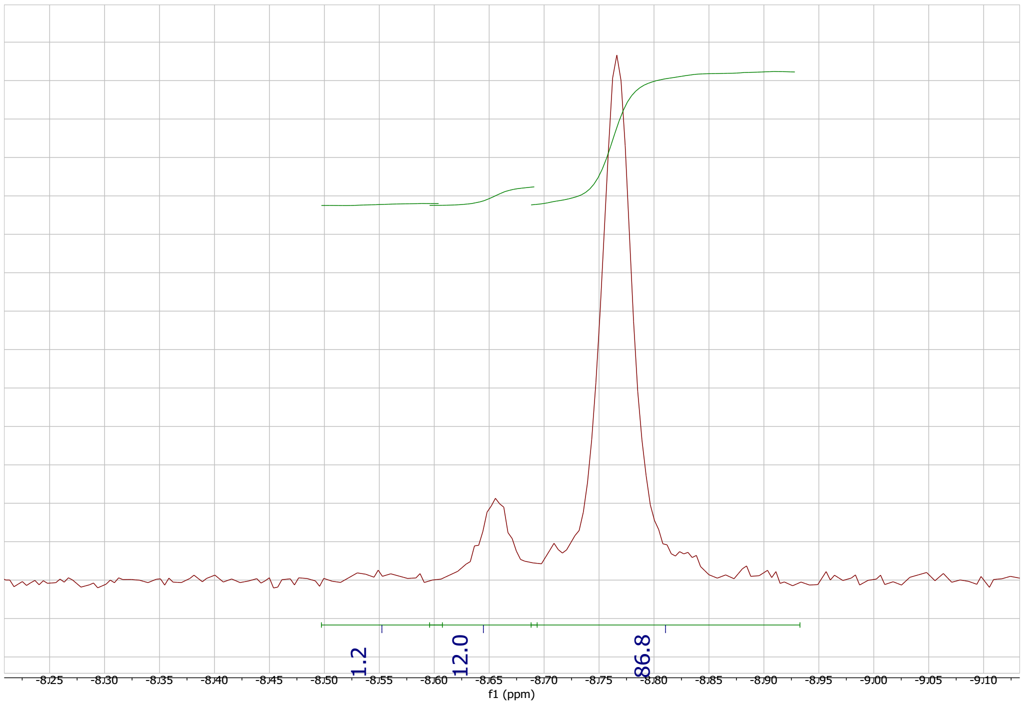

Figure S96. Spectrum corresponding to entry 6 of deuteration of P(*p*-tolyl)$_3$ (**2**) using **Ru@PVP**.

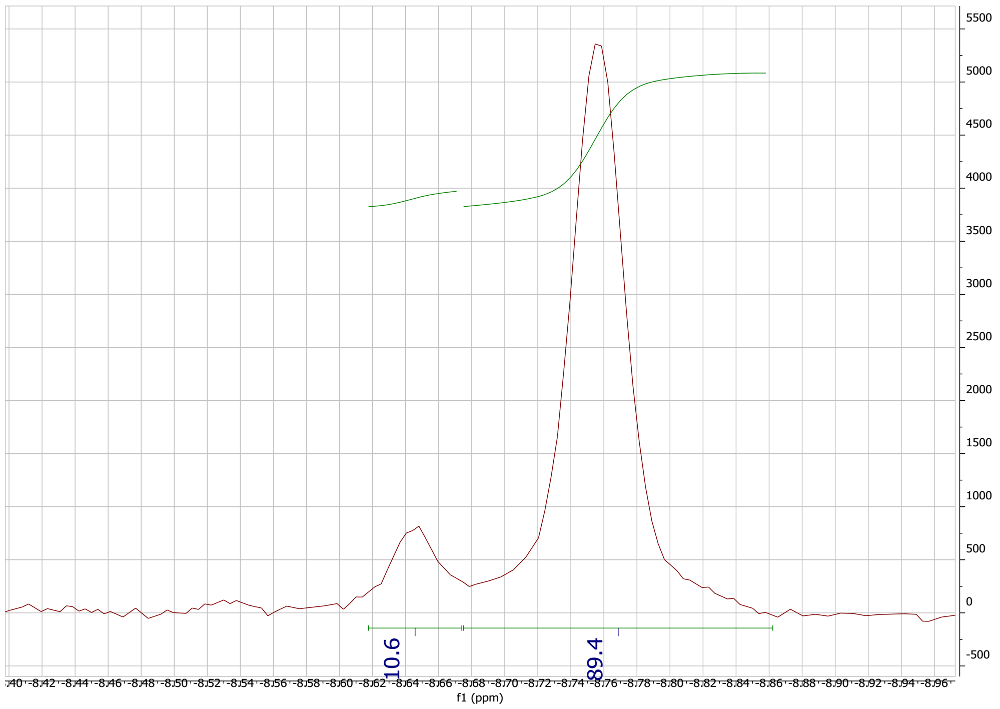

Figure S97. Spectrum corresponding to entry 7 of deuteration of P(*p*-tolyl)$_3$ (**2**)using **Ru@NHC**.

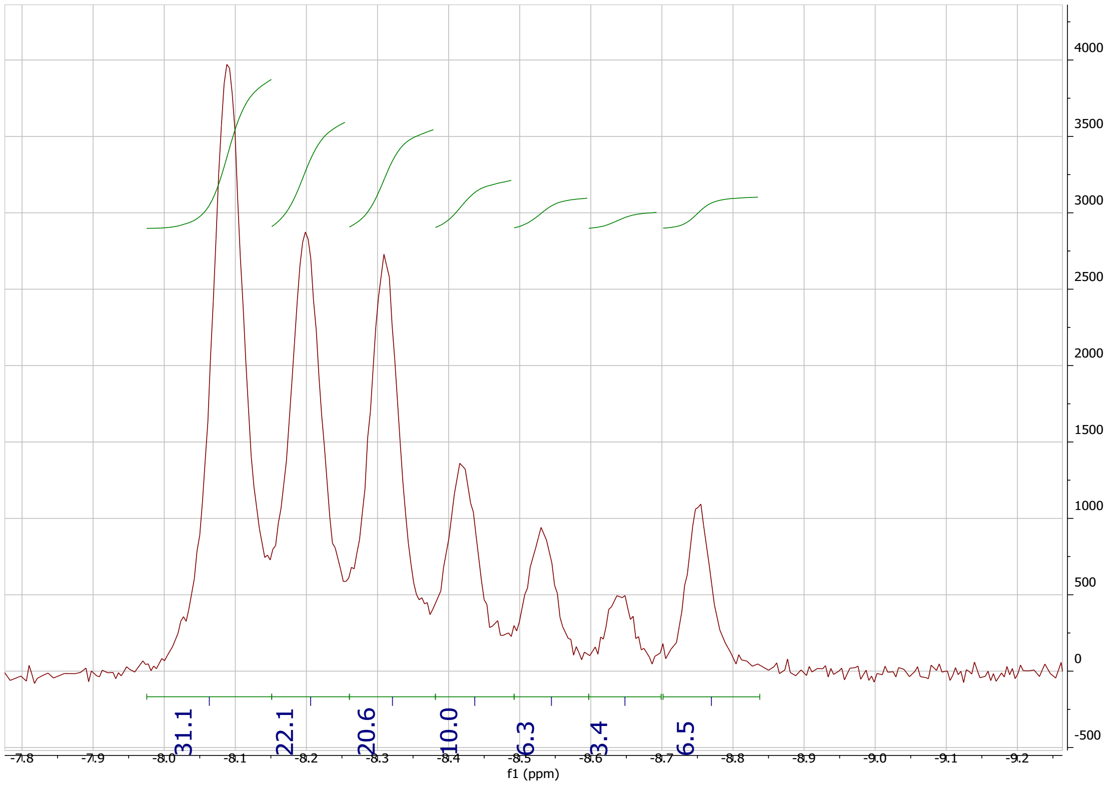

Figure S98. Spectrum corresponding to entry 8 of deuteration of P(*p*-tolyl)$_3$ (**2**) using **Rh@NHC**.

5.3. Deuteration of PMePh2 (3)

Data according to Scheme 2 b)

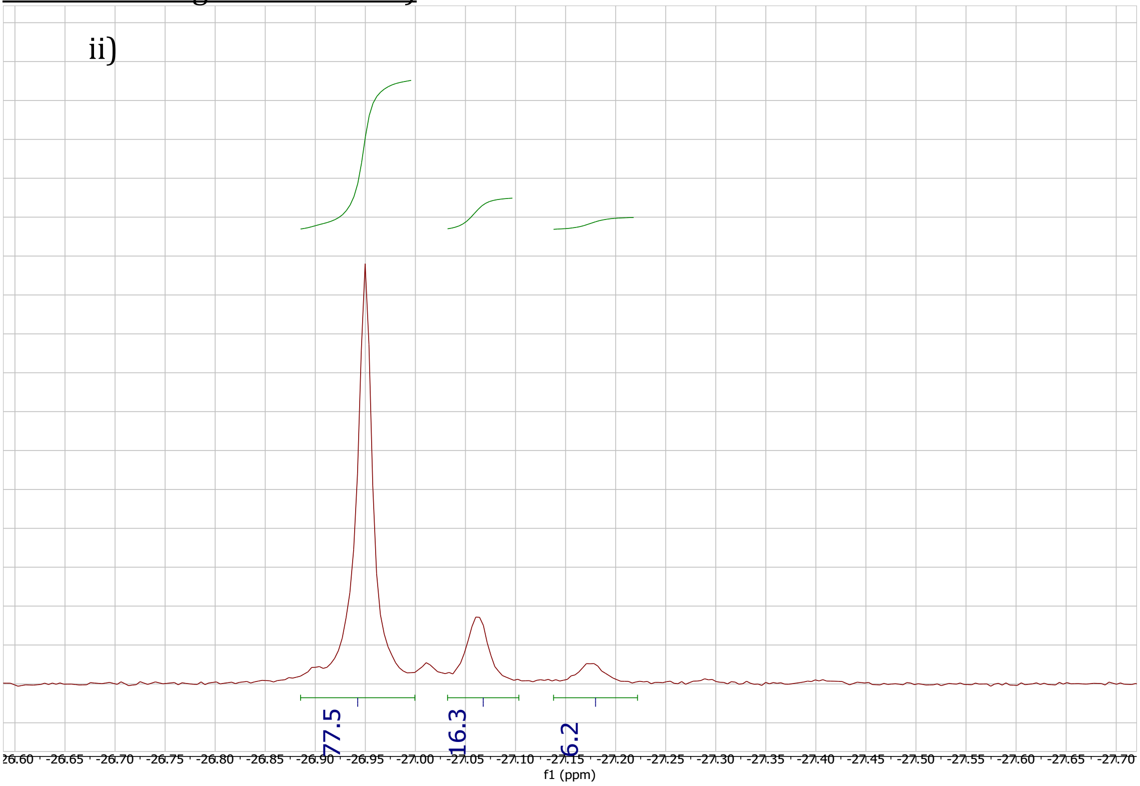

Figure S99. $^{31}P\{^{1}H\}$ NMR spectrum the reaction mixture resulting of the H/D exchange reaction of **3** at 55°C for 8 days using Ru@PVC.

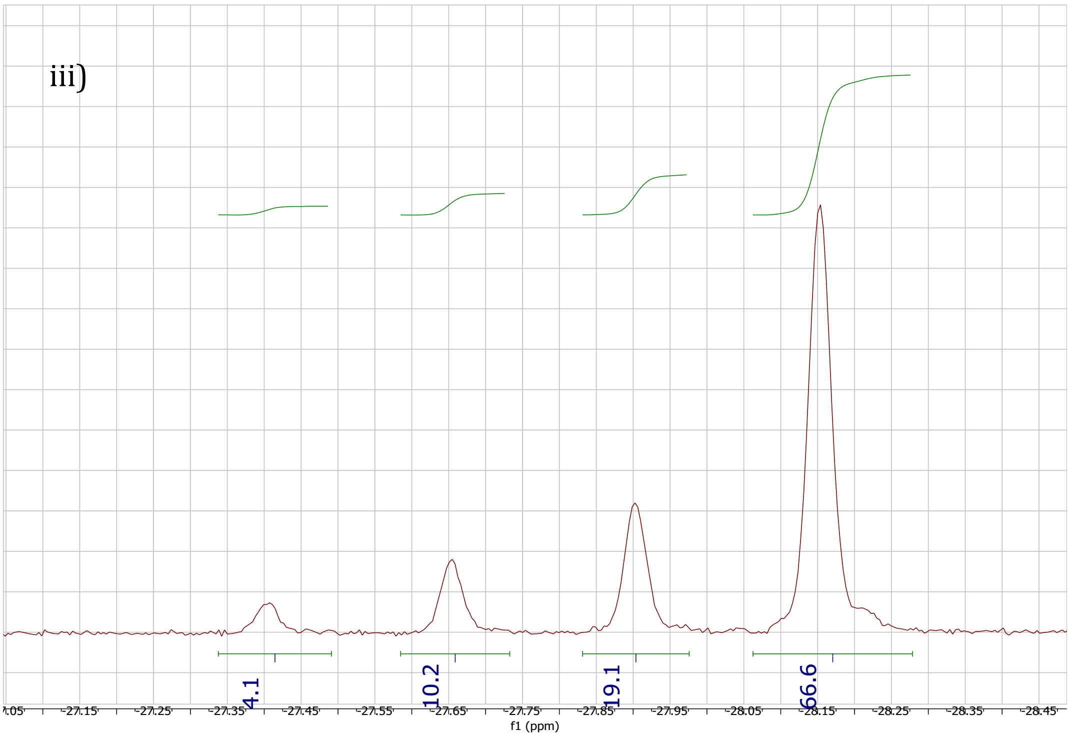

Figure S100. $^{31}P\{^{1}H\}$ NMR spectrum of the reaction mixture resulting from the H/D exchange of **3** at 55°C for 8 days using Ru@NHC.

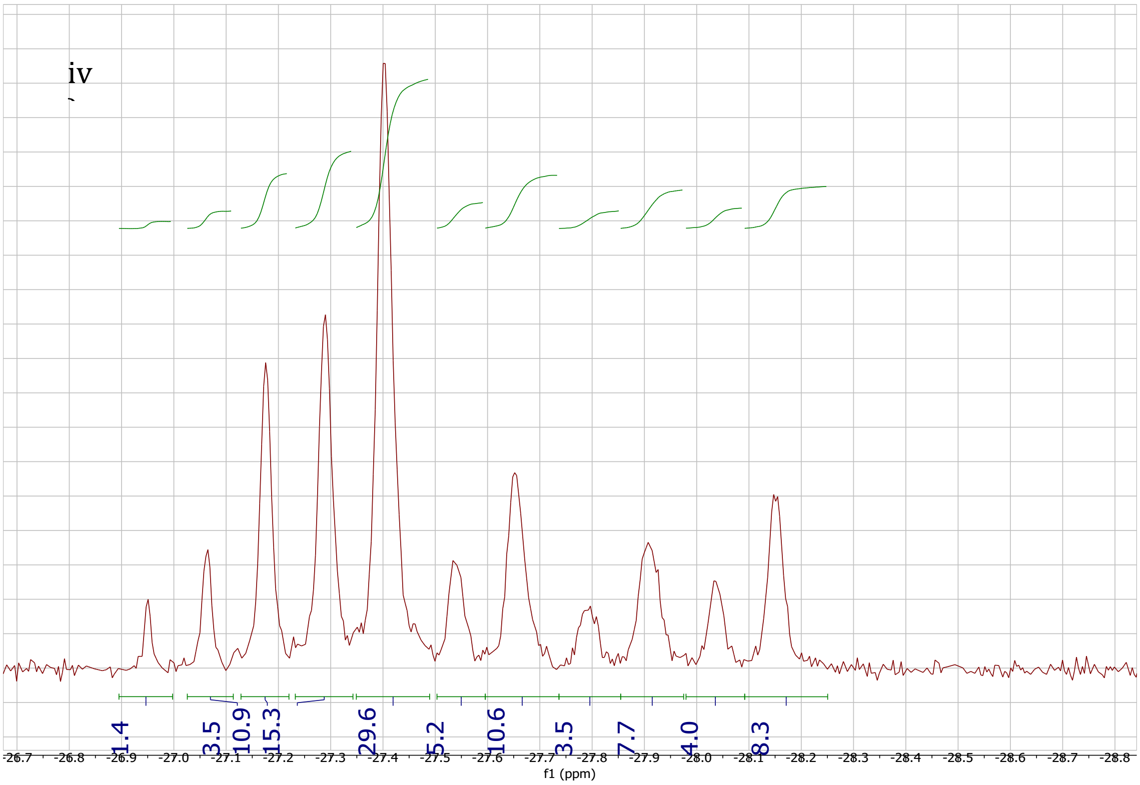

Figure S101. $^{31}P\{^{1}H\}$ NMR spectrum of the reaction mixture resulting from the H/D exchange reaction of **3** at 55°C for 8 days using Rh@NHC.

5.4. Deuteration of P(*o*-tolyl)3 (4)

Data corresponding to Scheme 3.

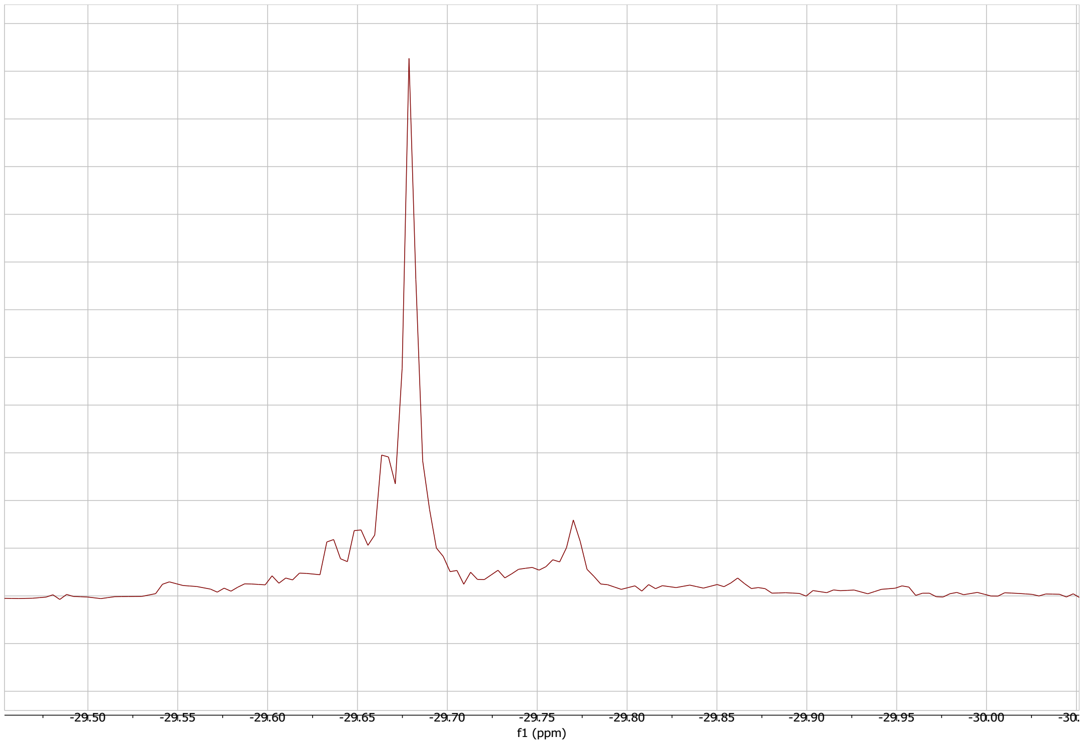

Figure S102. H/D exchange of substrate **4** in the presence of **Rh@NHC** catalyst at 55°C during 7h.

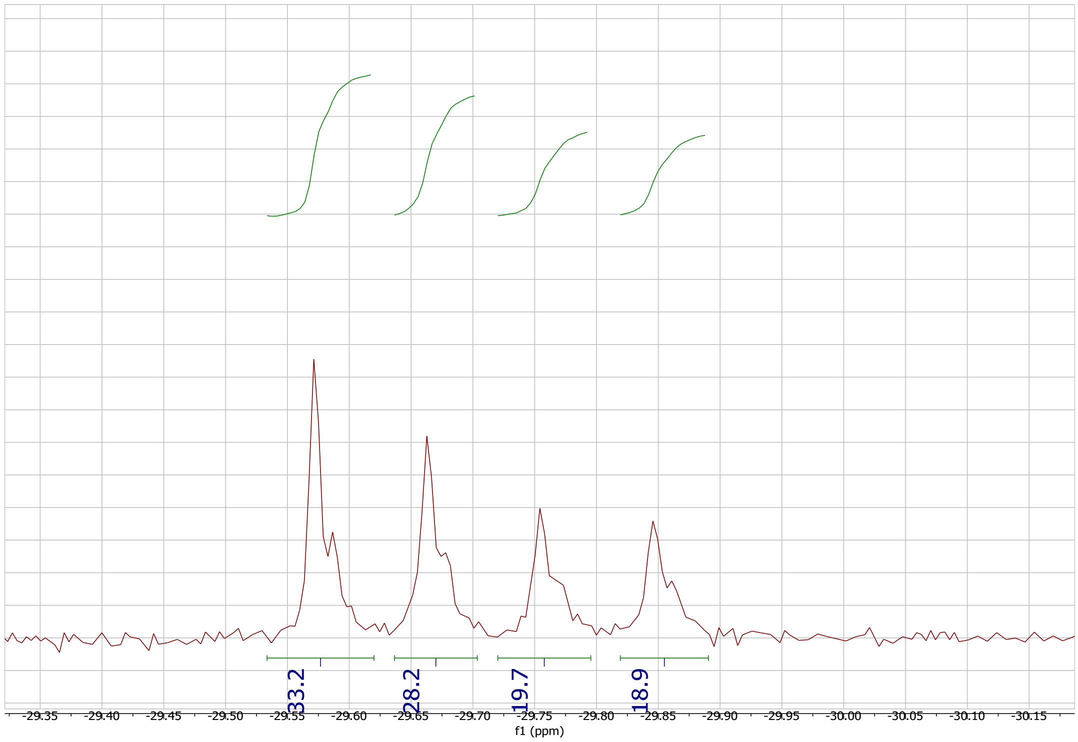

Figure S103. H/D exchange of substrate **4** in the presence of **Rh@NHC** catalyst at 55°C during 2 days.

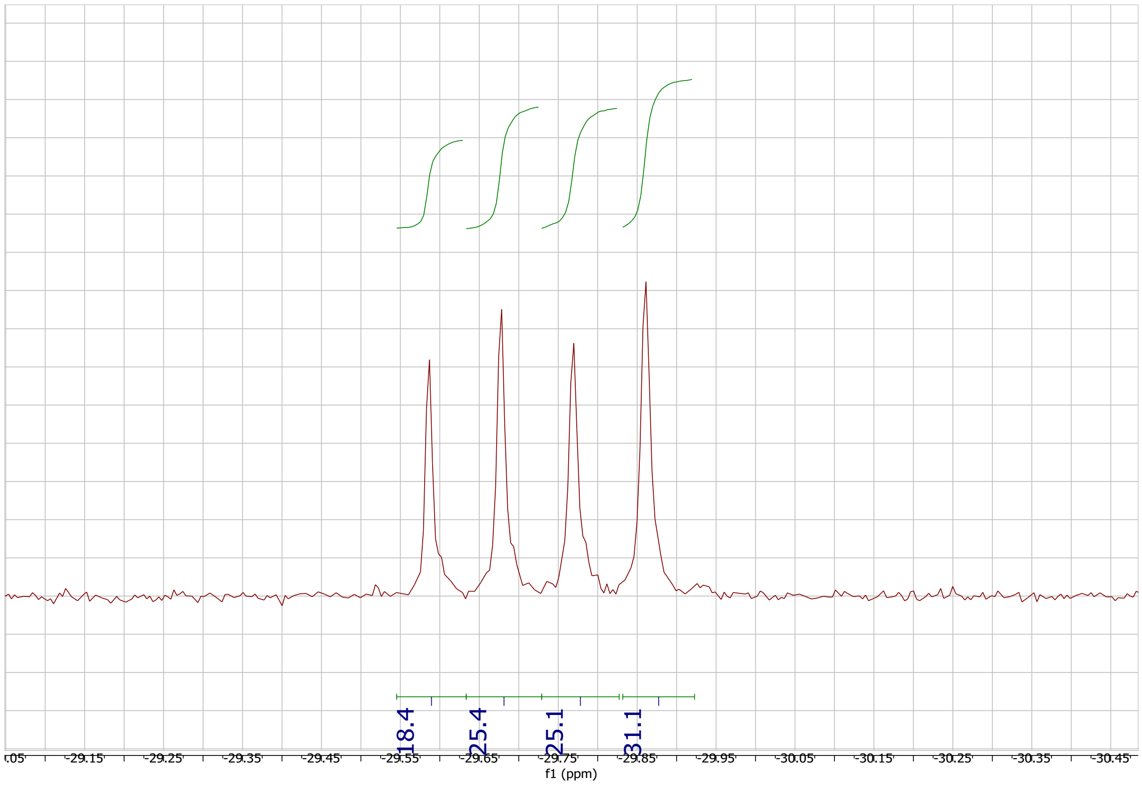

Figure S104. H/D exchange of substrate **4** in the presence of **Rh@NHC** catalyst at 55°C during 8 days.

12days

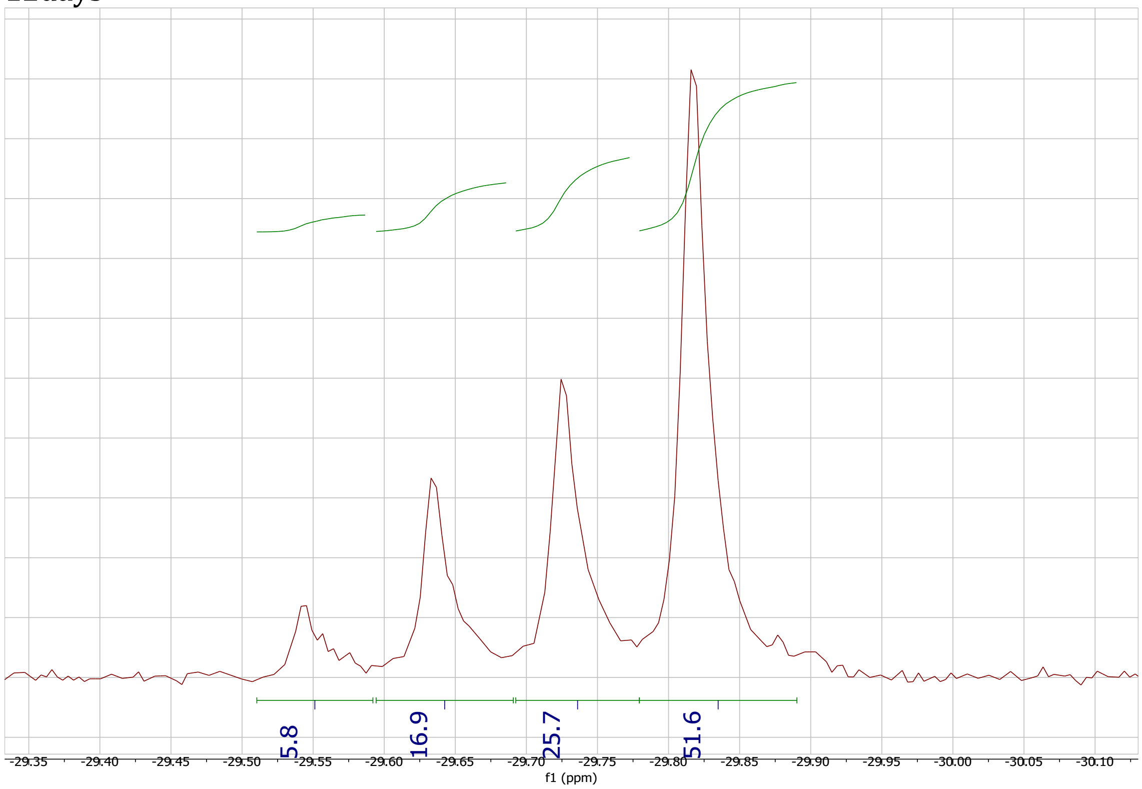

Figure S105. H/D exchange of substrate **4** in the presence of **Rh@NHC** catalyst at 55°C during 8 days.

5.5. Deuteration of diphenylphosphinomethane (11)

Data corresponding to Scheme 4b:

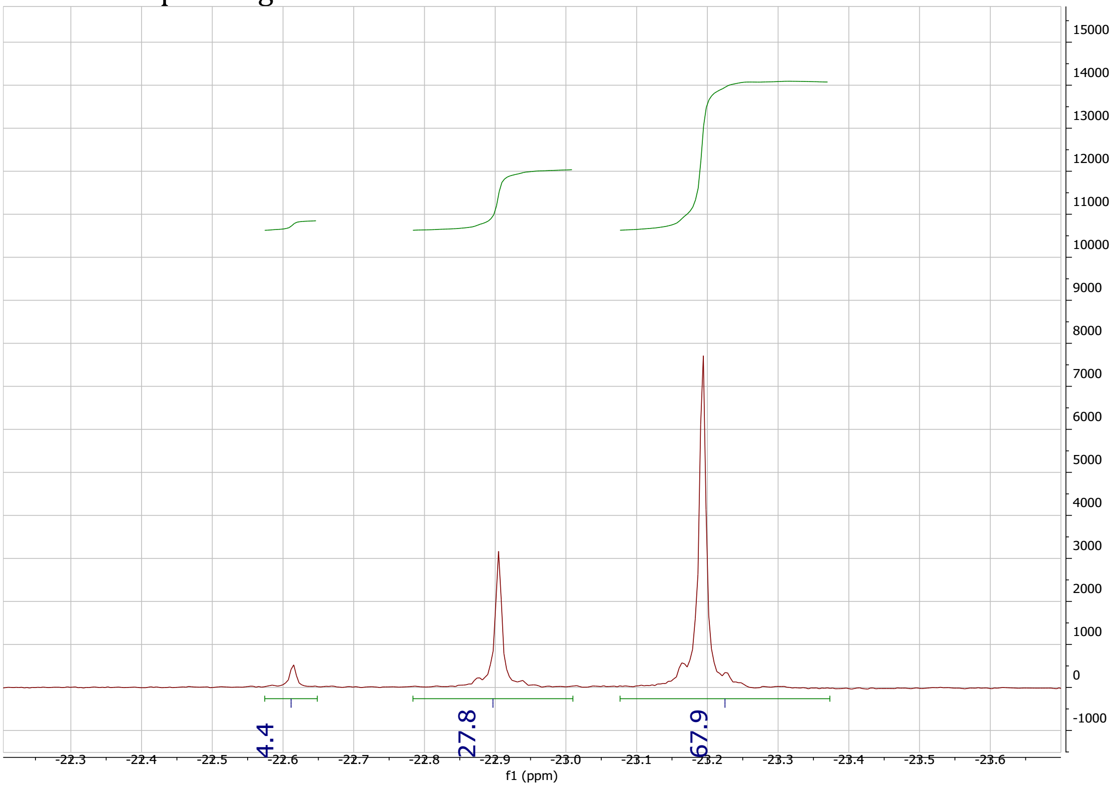

Figure S106. H/D exchange of substrate **11** in the presence of **Rh@NHC** catalyst at 55°C for 2 days.

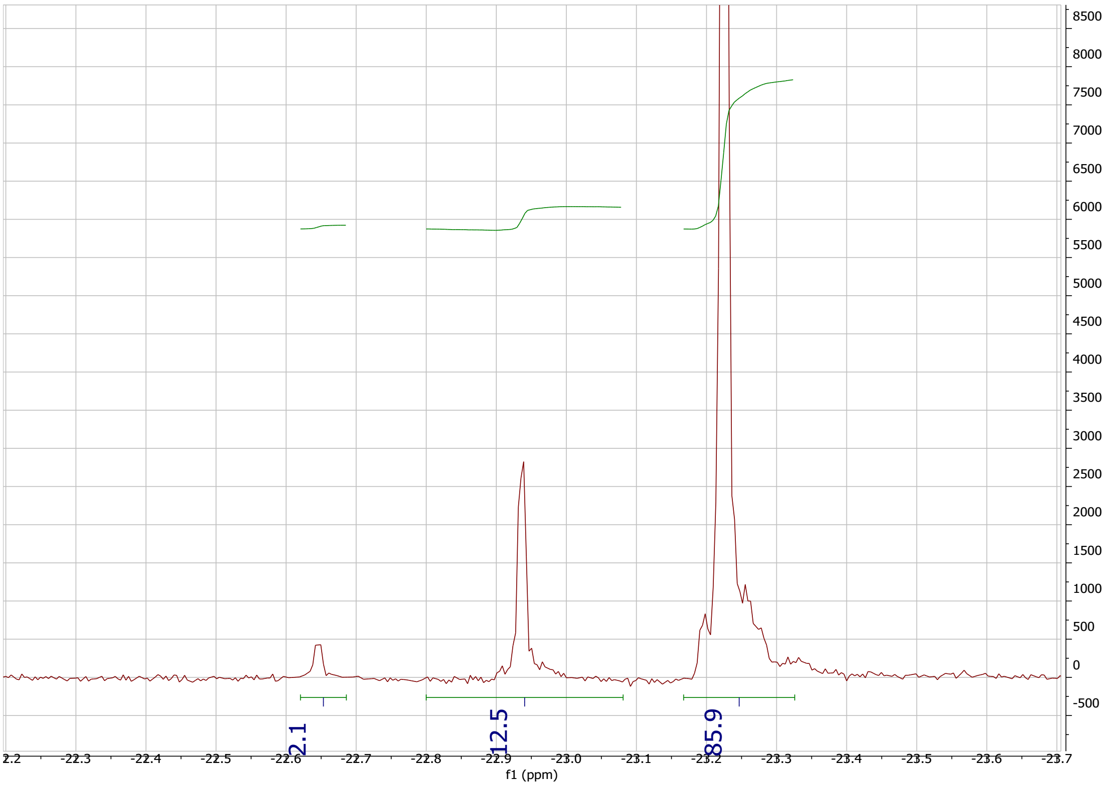

Figure S107. H/D exchange of substrate **11** in the presence of **Rh@NHC** catalyst at 55°C for 8 days.

5.6. Deuteration of diphenylphosphinobutane (12)

Data corresponding to Scheme 4 c)

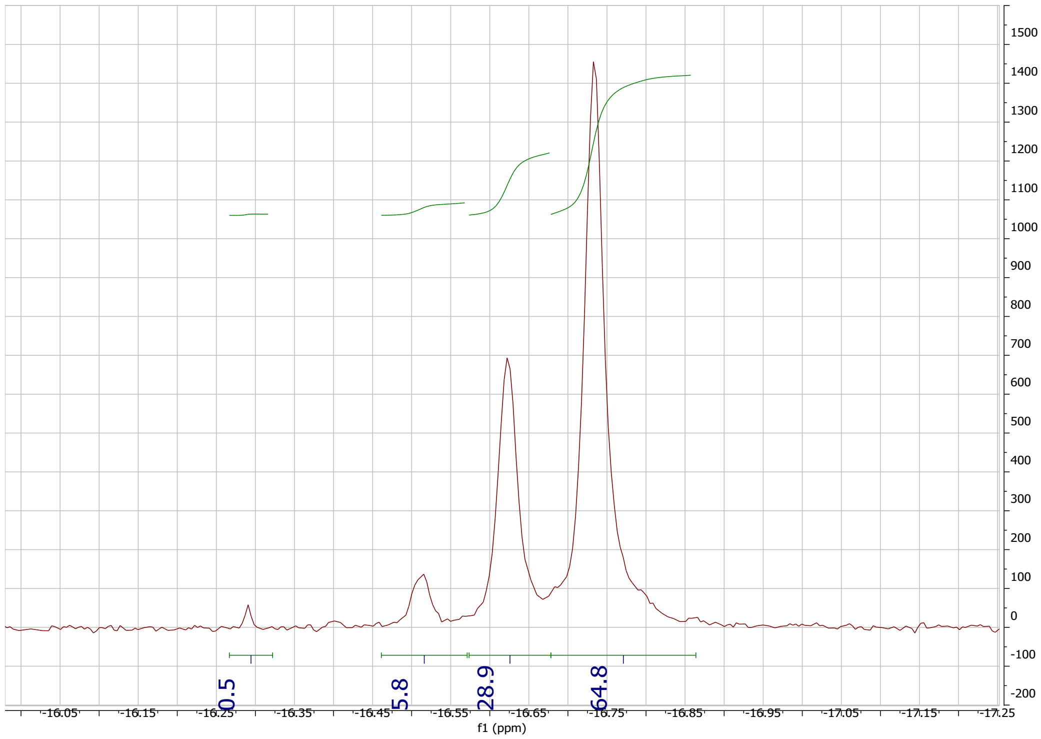

Figure S108. H/D exchange of substrate **12** in the presence of **Ru@PVP** catalyst at 55°C for 48h.

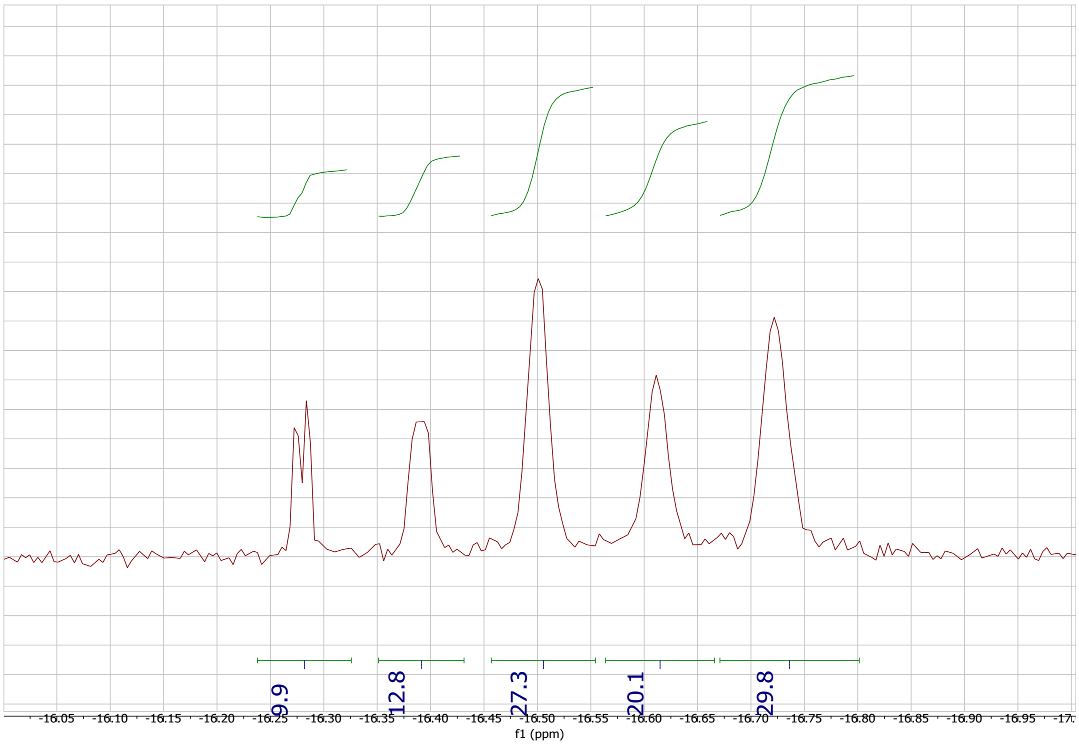

Figure S109. H/D exchange of substrate **12** in the presence of **Ru@NHC** catalyst at 55°C for 48h.

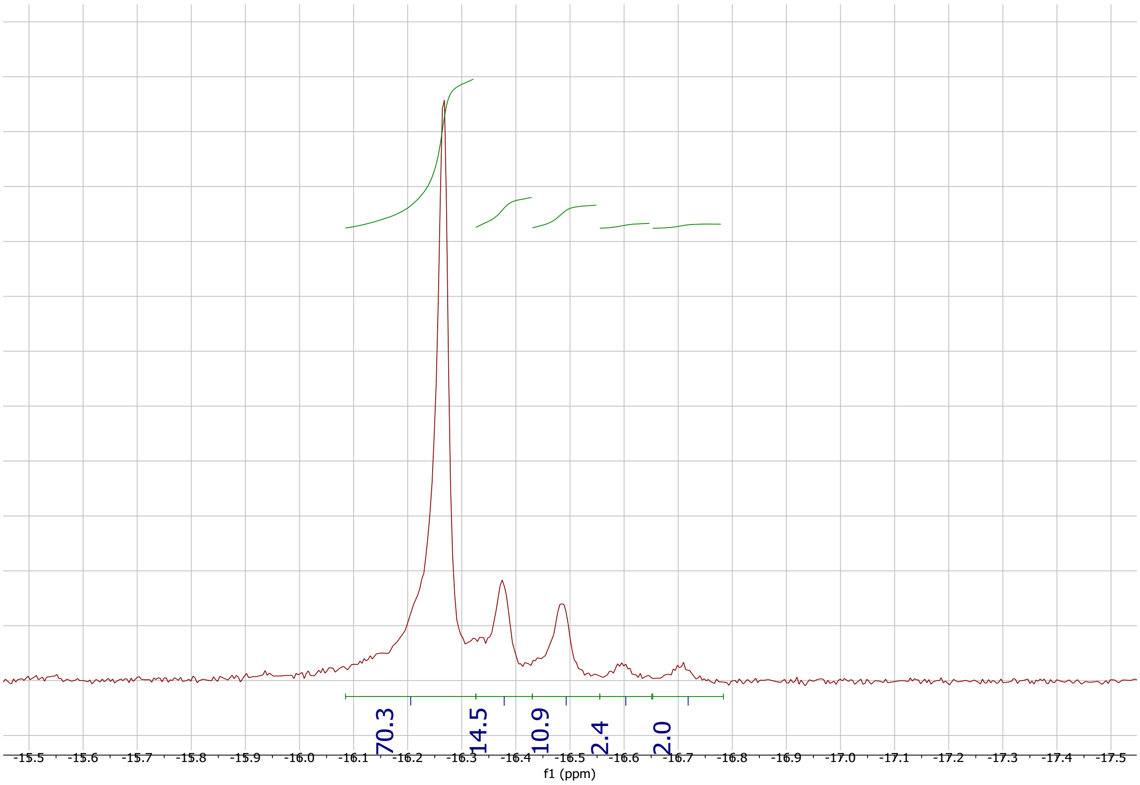

Figure S110. H/D exchange of substrate **12** in the presence of **Rh@NHC** catalyst at 55°C for 48h.

6. Computational Information

6.1. Computational Details

All calculations were performed within the periodic Density Functional Theory approach[4] using the plane-wave based Vienna ab Initio Simulation Package (VASP).[5] The exchange correlation functional used was revised Perdew-Burke-Ernzerhof (RPBE)[6] with an energy cut-off for the plane-waves basis set of 400 eV. The core electrons were simulated by the Projector Augmented Wave (PAW) method.[7] The wave function was considered to converge when the energy difference between one electronic step and the previous one was less than $1\cdot10^{-5}$ eV, whereas the minimum geometry was obtained when all forces were smaller than 0.01 eV A^{-1}. To set the partial occupancies the Methfessel-Paxton scheme[8] was used with smearing of 0.05 eV. The system under study was placed at the center of a 30 Å lattice cubic box with a minimum vacuum space between two neighboring systems of more than 10 Å to avoid possible interactions between periodic images. Since we were dealing with a molecular system only the Γ k-point was taken into account in the integration of the Brillouin zone. The Improved Dimer method[9] was employed to locate the transition states. Once the transition state was found, we performed a frequency calculation to ensure the TS character, and then displacements of the frequency was applied to the transition state, in both directions, forward and reverse, to guarantee the connection between reactants, transition and intermediate states.

6.2

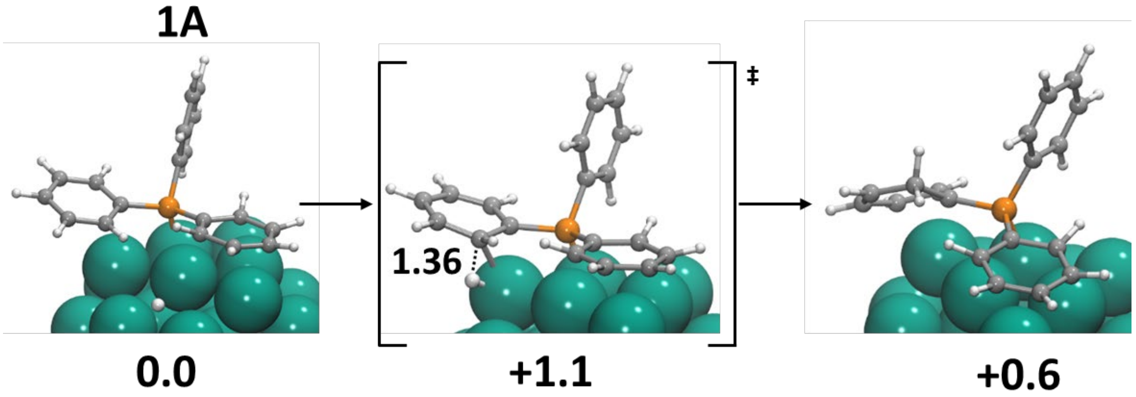

Figure S111. Alternative associative mechanism for the H/D exchange on the ortho position of **1**. Energy barrier and relative energies in eV and distances in Å.

6.3

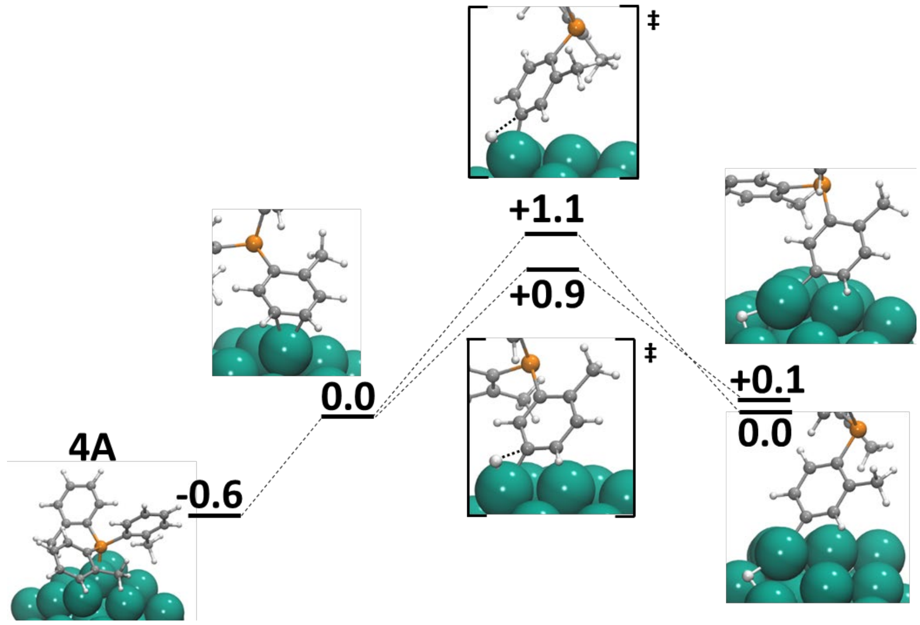

Figure S112. Proposed associative mechanism for the H/D exchange of 5-meta and *para* aromatic positions of phosphine **4**. Energies in eV and distance in Å.

7. DFT-optimized cartesian coordinates

1A

E = -552.52045 eV

Rh	13.494431	14.010385	17.219547
Rh	13.770614	14.135779	19.749125
Rh	14.033543	14.268660	22.273450
Rh	15.954273	13.076294	20.812344
Rh	15.661488	13.002108	18.190561
Rh	15.250906	15.661139	18.081115
Rh	12.589165	16.107816	18.346513
Rh	11.361086	13.711859	18.593908
Rh	13.252013	11.816421	18.492101
Rh	14.413548	11.884806	16.073319
Rh	15.659141	14.258804	15.780348
Rh	13.747586	16.190391	15.863417
Rh	11.335225	14.993537	16.219635
Rh	11.758187	12.330451	16.319233
Rh	13.210286	13.831154	14.645764
Rh	17.752969	11.987048	19.197934
Rh	16.953720	17.308778	19.006984
Rh	11.687495	18.198381	19.470919
Rh	9.245754	13.441714	19.972828
Rh	13.001592	9.601238	19.789597
Rh	15.302271	9.750004	14.993481
Rh	17.799447	14.527552	14.390877
Rh	13.965677	18.428380	14.674777
Rh	9.195959	15.986195	15.286987
Rh	10.030590	10.669256	15.468125
Rh	12.982097	13.699538	12.096710
Rh	15.549904	15.831162	20.723946
Rh	12.818953	16.277553	20.971640
Rh	11.589172	13.825259	21.232248
Rh	13.501118	11.888418	21.131926
Rh	17.466373	14.685806	19.134026
Rh	15.428191	10.731556	19.538093
Rh	16.606464	10.810620	17.028919
Rh	17.871685	13.278645	16.774791
Rh	14.364297	17.828035	19.286932
Rh	17.481737	15.956778	16.712345
Rh	15.510555	17.959594	16.782205
Rh	10.400686	15.864978	19.791711
Rh	12.827212	18.376065	17.043019
Rh	10.358123	17.154658	17.356499
Rh	11.064419	11.452564	19.948899
Rh	9.105564	14.725967	17.633970
Rh	9.553836	11.987786	17.724390
Rh	11.468342	10.049182	17.613558
Rh	14.163323	9.583325	17.397989

Rh	16.591641	12.127150	14.597191
Rh	12.676767	10.160768	15.123689
Rh	14.153097	11.704963	13.412921
Rh	15.410999	14.274246	13.066025
Rh	11.560726	17.262197	14.862880
Rh	13.429073	16.203014	13.171025
Rh	9.528704	13.318422	15.299877
Rh	11.006222	14.843158	13.576831
Rh	11.453785	12.174774	13.657038
Rh	16.014056	16.601556	14.461991
P	17.094872	16.560477	12.490790
C	17.488795	18.171525	11.662125
C	17.470465	19.361752	12.414702
C	17.779956	18.226274	10.282653
C	17.741893	20.592091	11.797052
C	18.040093	19.458834	9.667981
C	18.022247	20.642464	10.424173
C	15.743547	15.777088	11.509874
C	15.632259	14.461444	10.899953
C	14.577789	16.705577	11.446994
C	14.349823	14.056136	10.318875
C	13.327920	16.267033	10.888070
C	13.213618	14.936294	10.311443
C	18.700656	15.642472	12.513137
C	19.874205	16.405759	12.824578
C	18.858632	14.223207	12.319206
C	21.126342	15.802986	12.900831
C	20.151311	13.635643	12.417113
C	21.272437	14.410302	12.699669
H	17.233895	19.315704	13.481968
H	17.795727	17.310348	9.685675
H	17.725999	21.510456	12.389460
H	18.253819	19.496053	8.596807
H	18.223167	21.601856	9.941120
H	16.515128	13.908545	10.576007
H	14.792950	17.776729	11.472323
H	14.344899	13.170495	9.679133
H	12.571170	17.009791	10.628187
H	12.368374	14.724722	9.650647
H	19.785777	17.480694	12.992432
H	18.047803	13.615643	11.919206
H	22.002412	16.417254	13.122333
H	20.248149	12.560810	12.246142
H	22.259782	13.947655	12.763161

1B

E = -551.29687 eV

Rh	17.863257	15.654572	15.709877
Rh	17.905487	15.531268	18.273953

Rh	17.929329	15.398247	20.797293
Rh	19.690461	13.952240	19.397546
Rh	19.649281	14.134069	16.749279
Rh	19.817104	16.833659	16.885286
Rh	17.308261	17.823328	16.961596
Rh	15.562834	15.722027	16.875605
Rh	17.036570	13.459339	16.753174
Rh	18.421576	13.474020	14.439058
Rh	20.135694	15.557686	14.521031
Rh	18.686500	17.830906	14.637854
Rh	16.044990	17.132587	14.623720
Rh	15.886047	14.435245	14.488022
Rh	17.820944	15.757025	13.134025
Rh	21.417325	12.620216	17.804704
Rh	21.740102	17.979681	18.059174
Rh	16.775234	19.963201	18.215632
Rh	13.333692	15.816341	18.064560
Rh	16.229662	11.286615	17.820446
Rh	19.028653	11.319645	13.214092
Rh	22.388617	15.465064	13.342746
Rh	19.526810	19.988041	13.587728
Rh	14.347002	18.718570	13.602151
Rh	17.847703	15.901704	10.586931
Rh	19.903666	16.694531	19.534991
Rh	17.341123	17.743775	19.600401
Rh	15.573202	15.619592	19.506537
Rh	17.013763	13.277533	19.367635
Rh	21.663559	15.286020	17.984725
Rh	18.849448	11.865118	17.856146
Rh	20.240709	11.868681	15.489055
Rh	22.007601	13.987813	15.571185
Rh	19.298782	19.060549	18.210609
Rh	22.194543	16.717913	15.715032
Rh	20.720462	19.063353	15.817232
Rh	14.974872	17.943734	18.176168
Rh	18.176084	20.090923	15.927129
Rh	15.501256	19.402458	15.890167
Rh	14.658575	13.511113	17.948930
Rh	13.712982	17.317613	15.862750
Rh	13.414342	14.574967	15.623428
Rh	15.045271	12.196397	15.591412
Rh	17.574858	11.196787	15.496499
Rh	20.760561	13.330723	13.225042
Rh	16.456494	12.234372	13.172483
Rh	18.390703	13.555277	11.792852
Rh	21.048764	17.783316	13.443828
Rh	20.160353	15.664344	11.877961
Rh	16.924554	19.430173	13.556841
Rh	18.697283	17.965656	11.993438

Rh	14.002853	16.047769	13.327922
Rh	16.020566	17.349941	11.998985
Rh	15.837573	14.547710	11.865686
Rh	13.938820	13.226855	13.376724
P	11.468915	14.193416	14.580858
C	11.682366	12.880236	13.285142
C	10.926476	15.673074	13.620970
C	10.007721	13.681525	15.590566
C	12.294752	11.625707	13.668169
C	11.131406	12.975800	11.962178
C	9.711573	16.334584	13.869100
C	11.846052	16.246021	12.685302
C	9.788040	14.308974	16.835592
C	9.126871	12.661637	15.174391
C	12.344940	10.547426	12.735066
C	11.194746	11.903438	11.079356
C	9.389749	17.524931	13.193483
C	11.484372	17.417552	11.974261
C	8.702138	13.938319	17.638493
C	8.050366	12.280346	15.990018
C	11.811496	10.683512	11.459402
C	10.272499	18.065378	12.245662
C	7.834052	12.917584	17.220195
H	12.477601	11.409962	14.725515
H	10.638952	13.898862	11.652300
H	9.006213	15.925474	14.594296
H	12.624945	15.563999	12.163682
H	10.479808	15.085890	17.174111
H	9.278422	12.157705	14.218001
H	12.793207	9.602597	13.052068
H	10.756553	12.003589	10.083356
H	8.437430	18.019068	13.400431
H	12.158866	17.798044	11.203914
H	8.541691	14.437956	18.596941
H	7.382075	11.480179	15.661568
H	11.851927	9.849988	10.754863
H	10.010751	18.980667	11.709453
H	6.996275	12.616262	17.854020

1C
E = -550.72324 eV

Rh	17.464540	16.795125	16.521714
Rh	17.421568	16.762354	19.080552
Rh	17.406130	16.745380	21.612659
Rh	19.303473	15.366500	20.290876
Rh	19.294483	15.406411	17.650444
Rh	19.307882	18.130581	17.669185
Rh	16.708178	18.961440	17.660150
Rh	15.135868	16.755696	17.620995

Rh	16.710872	14.593012	17.625248
Rh	18.117220	14.596672	15.349216
Rh	19.739140	16.770191	15.344668
Rh	18.146281	18.968062	15.355057
Rh	15.579726	18.101904	15.349680
Rh	15.547394	15.404022	15.329083
Rh	17.419368	16.766002	13.945829
Rh	21.116002	14.056621	18.782611
Rh	21.119173	19.440827	18.794281
Rh	15.989528	21.108602	18.784556
Rh	12.841531	16.767348	18.778351
Rh	16.004112	12.411198	18.775248
Rh	18.843971	12.418419	14.197861
Rh	21.994913	16.756409	14.203064
Rh	18.841063	21.124069	14.216282
Rh	13.730567	19.468426	14.210013
Rh	17.440364	16.776254	11.377142
Rh	19.307638	18.133085	20.303826
Rh	16.671750	18.986226	20.300050
Rh	15.067900	16.748272	20.268325
Rh	16.676877	14.521524	20.272326
Rh	21.218459	16.744801	18.849739
Rh	18.570671	13.146242	18.822636
Rh	20.018064	13.148160	16.488986
Rh	21.661548	15.359364	16.495403
Rh	18.586466	20.366116	18.849443
Rh	21.665821	18.134108	16.503195
Rh	20.050599	20.380055	16.493353
Rh	14.343019	18.985942	18.823853
Rh	17.419751	21.227935	16.492076
Rh	14.798196	20.361116	16.490779
Rh	14.337580	14.525832	18.818814
Rh	13.174830	18.128919	16.481813
Rh	13.170397	15.395040	16.494817
Rh	14.793933	13.164151	16.485610
Rh	17.405463	12.299336	16.474175
Rh	20.478951	14.538561	14.147753
Rh	16.255673	13.152504	14.143735
Rh	18.133113	14.533059	12.695854
Rh	20.496092	18.994649	14.142734
Rh	19.762006	16.756052	12.703530
Rh	16.244994	20.359900	14.147745
Rh	18.141478	18.988734	12.714045
Rh	13.639240	16.770390	14.138492
Rh	15.520851	18.139929	12.691625
Rh	15.537479	15.398794	12.690268
Rh	13.695403	14.055521	14.181763
P	12.026455	12.824682	13.161357
C	10.433765	12.839589	14.096453

C	12.438958	11.039211	12.929826
C	11.596562	13.437147	11.472712
C	9.970467	14.076649	14.597577
C	9.682086	11.674214	14.348415
C	12.139954	10.335701	11.744768
C	13.096934	10.362774	13.980037
C	12.652810	13.779647	10.599985
C	10.267151	13.592902	11.030978
C	8.774877	14.145624	15.323210
C	8.489912	11.747272	15.086020
C	12.493226	8.983604	11.617113
C	13.438749	9.010611	13.851973
C	12.384199	14.262012	9.313372
C	10.002519	14.084143	9.743057
C	8.032800	12.980108	15.572432
C	13.140511	8.318118	12.668163
C	11.057384	14.419650	8.882701
H	10.554558	14.985525	14.426932
H	10.023985	10.707247	13.975521
H	11.637819	10.840118	10.917402
H	13.357641	10.904534	14.894078
H	13.689092	13.676233	10.936019
H	9.435255	13.336053	11.688921
H	8.429614	15.109893	15.704321
H	7.919605	10.835226	15.279553
H	12.262575	8.452101	10.690336
H	13.950755	8.503384	14.673317
H	13.212684	14.527220	8.652064
H	8.967086	14.205011	9.415000
H	7.105808	13.032913	16.148975
H	13.418835	7.266639	12.562860
H	10.847775	14.808400	7.883021

$1TS_{ortho\text{-}f}$

E = -551.06075 eV

Rh	13.521980	14.040473	17.246347
Rh	13.771626	13.959809	19.789442
Rh	14.002696	13.907193	22.308224
Rh	15.899815	12.747812	20.784124
Rh	15.609036	12.835749	18.152614
Rh	15.357672	15.514980	18.250028
Rh	12.720208	16.103793	18.512289
Rh	11.352692	13.782217	18.578519
Rh	13.134959	11.772165	18.348070
Rh	14.320824	11.954960	15.938633
Rh	15.712944	14.255273	15.852698
Rh	13.909682	16.313427	16.068019
Rh	11.441195	15.215210	16.292852
Rh	11.700808	12.529352	16.202781

Rh	13.273727	14.077579	14.659607
Rh	17.631929	11.620570	19.100946
Rh	17.144283	17.003515	19.257925
Rh	11.947297	18.169834	19.779212
Rh	9.210741	13.547702	19.913519
Rh	12.739277	9.512087	19.508663
Rh	15.101627	9.819377	14.764977
Rh	17.901502	14.493956	14.520691
Rh	14.313681	18.625161	15.075577
Rh	9.377869	16.395870	15.410442
Rh	9.865234	11.049913	15.253866
Rh	13.063377	14.124351	12.086520
Rh	15.628931	15.512742	20.874298
Rh	12.948720	16.093535	21.132788
Rh	11.552273	13.734341	21.221774
Rh	13.343758	11.668399	21.005425
Rh	17.496630	14.332266	19.251693
Rh	15.225310	10.512355	19.374636
Rh	16.422801	10.662587	16.889267
Rh	17.856944	13.072820	16.793078
Rh	14.590370	17.674599	19.559611
Rh	17.608573	15.779633	16.897618
Rh	15.782027	17.870897	17.076105
Rh	10.490730	15.914474	19.909519
Rh	13.125008	18.467764	17.398416
Rh	10.603464	17.375675	17.569124
Rh	10.914643	11.474638	19.776672
Rh	9.194224	15.026785	17.680564
Rh	9.454473	12.238598	17.571029
Rh	11.237038	10.191989	17.373168
Rh	13.934316	9.577308	17.140643
Rh	16.579330	12.100793	14.571007
Rh	12.492784	10.355476	14.933464
Rh	14.038936	11.889218	13.314024
Rh	15.546976	14.215777	13.156353
Rh	11.794710	17.568845	15.118007
Rh	13.741118	16.645077	13.405105
Rh	9.555516	13.734601	15.251435
Rh	11.197023	15.292685	13.642955
Rh	11.451570	12.546594	13.566936
Rh	16.187909	16.619598	14.600640
P	16.956187	16.583477	12.499863
C	17.235744	18.149728	11.554495
C	17.198284	19.390066	12.218449
C	17.530091	18.108624	10.175506
C	17.461904	20.575607	11.515424
C	17.788738	19.295033	9.476014
C	17.759111	20.529194	10.145804
C	15.730638	15.584126	11.536448

C	15.916412	14.286988	10.912046
C	14.329409	16.080283	11.558485
C	14.814068	13.694269	10.187177
C	13.384298	15.663270	10.550621
C	13.628246	14.410460	9.886506
C	18.649557	15.803906	12.545464
C	19.734182	16.696966	12.807450
C	18.955750	14.400263	12.456431
C	21.041313	16.231660	12.947633
C	20.295469	13.951087	12.620633
C	21.330303	14.852563	12.862624
H	16.966513	19.418073	13.287085
H	17.561837	17.152383	9.646481
H	17.435578	21.533967	12.039984
H	18.016882	19.255937	8.407838
H	17.966528	21.452312	9.598924
H	16.908179	13.881852	10.712691
H	13.907654	17.624807	12.123374
H	14.967210	12.710236	9.738647
H	12.506550	16.267918	10.321016
H	12.922048	14.022457	9.149446
H	19.539205	17.766712	12.896463
H	18.219311	13.679254	12.101447
H	21.845522	16.947458	13.133842
H	20.498793	12.881196	12.532541
H	22.355496	14.495609	12.981865

1I$_{ortho\text{-}f}$

E = -551.47416 eV

Rh	13.510288	14.028225	17.227594
Rh	13.755514	13.940108	19.773819
Rh	13.974823	13.873905	22.294361
Rh	15.874991	12.713872	20.766751
Rh	15.575166	12.798414	18.135857
Rh	15.360014	15.482101	18.249721
Rh	12.728200	16.102617	18.505233
Rh	11.335642	13.786998	18.555326
Rh	13.097034	11.762365	18.329186
Rh	14.288923	11.941003	15.917693
Rh	15.717427	14.222323	15.848711
Rh	13.923894	16.315458	16.083629
Rh	11.440406	15.223878	16.282154
Rh	11.678635	12.540495	16.173055
Rh	13.286507	14.121031	14.649643
Rh	17.600974	11.570392	19.083340
Rh	17.166997	16.945277	19.260631
Rh	11.955776	18.166239	19.768682
Rh	9.190092	13.567736	19.892697
Rh	12.684198	9.510014	19.486744

Rh	15.059057	9.802533	14.741624
Rh	17.906541	14.443957	14.547838
Rh	14.319818	18.592477	15.052371
Rh	9.389237	16.421181	15.381874
Rh	9.844462	11.078233	15.210267
Rh	13.066497	14.169908	12.066963
Rh	15.618261	15.477630	20.880963
Rh	12.939620	16.084801	21.117746
Rh	11.529386	13.739546	21.200432
Rh	13.310666	11.648621	20.986142
Rh	17.475202	14.289339	19.248109
Rh	15.171181	10.478171	19.362076
Rh	16.384875	10.628763	16.887729
Rh	17.849296	13.016730	16.800117
Rh	14.610634	17.642039	19.551710
Rh	17.596418	15.748152	16.892601
Rh	15.798069	17.858010	17.076953
Rh	10.497317	15.925486	19.893367
Rh	13.145167	18.478012	17.389958
Rh	10.622059	17.389248	17.541723
Rh	10.874513	11.489060	19.767683
Rh	9.186271	15.046035	17.650832
Rh	9.428483	12.261439	17.547339
Rh	11.191593	10.211707	17.360696
Rh	13.890771	9.563925	17.110529
Rh	16.565075	12.064503	14.577544
Rh	12.450830	10.349611	14.912932
Rh	14.009090	11.883426	13.308853
Rh	15.562060	14.160036	13.138016
Rh	11.811949	17.567602	15.099946
Rh	13.718078	16.612778	13.317306
Rh	9.540930	13.764988	15.233212
Rh	11.181323	15.283903	13.630108
Rh	11.454485	12.558692	13.545993
Rh	16.149586	16.546654	14.548569
P	17.001808	16.567968	12.481867
C	17.230916	18.127625	11.516151
C	17.149678	19.376300	12.159761
C	17.506617	18.075224	10.133394
C	17.349089	20.559494	11.432439
C	17.699619	19.259375	9.409673
C	17.623944	20.502533	10.058707
C	15.808463	15.530220	11.533248
C	15.983706	14.233832	10.904660
C	14.417856	16.055897	11.555105
C	14.880020	13.666356	10.165613
C	13.484067	15.666923	10.517934
C	13.709934	14.405651	9.860287
C	18.707253	15.833827	12.554546

C	19.770619	16.744554	12.821347
C	19.025586	14.432495	12.508460
C	21.077440	16.295505	13.019025
C	20.360817	13.999029	12.733671
C	21.378982	14.918135	12.988960
H	16.927455	19.414101	13.229618
H	17.570842	17.112362	9.619576
H	17.286401	21.525026	11.940710
H	17.910073	19.211638	8.338303
H	17.776671	21.424401	9.492108
H	16.970231	13.806940	10.724402
H	13.721761	18.312065	13.286076
H	15.015808	12.679641	9.717475
H	12.637259	16.302780	10.254739
H	13.004279	14.029103	9.116330
H	19.562192	17.814423	12.872715
H	18.306695	13.699263	12.140677
H	21.869607	17.023699	13.210146
H	20.578301	12.929720	12.679767
H	22.401716	14.574251	13.157563

1TS$_{meta\text{-}f}$

E = -550.25432 eV

Rh	13.493153	14.016195	17.190072
Rh	13.791359	14.135718	19.718930
Rh	14.071989	14.253090	22.245433
Rh	15.990717	13.073065	20.761743
Rh	15.674178	13.015215	18.142916
Rh	15.247689	15.676685	18.046389
Rh	12.590411	16.109966	18.325211
Rh	11.374088	13.704629	18.590492
Rh	13.279141	11.813225	18.458996
Rh	14.407082	11.904302	16.021583
Rh	15.640319	14.291747	15.746156
Rh	13.733417	16.205289	15.830905
Rh	11.316171	14.991749	16.220388
Rh	11.751966	12.326838	16.313055
Rh	13.171741	13.843060	14.628070
Rh	17.777864	11.991823	19.119080
Rh	16.945180	17.331890	18.963852
Rh	11.691617	18.192164	19.469585
Rh	9.278123	13.413888	19.991232
Rh	13.058667	9.594024	19.741195
Rh	15.299277	9.807254	14.894614
Rh	17.785034	14.562088	14.360737
Rh	13.929781	18.450728	14.666242
Rh	9.167748	15.960642	15.305986
Rh	10.025683	10.661933	15.474991
Rh	12.878101	13.690190	12.066697

Rh	15.567088	15.834742	20.684479
Rh	12.829336	16.257775	20.962514
Rh	11.627112	13.801693	21.222017
Rh	13.545374	11.879107	21.097855
Rh	17.470973	14.700181	19.100141
Rh	15.465787	10.732732	19.480881
Rh	16.616698	10.839811	16.950826
Rh	17.874297	13.314605	16.727549
Rh	14.352884	17.828924	19.280280
Rh	17.474674	15.982374	16.680453
Rh	15.475972	17.984487	16.758879
Rh	10.411715	15.843936	19.795458
Rh	12.809103	18.385027	17.035528
Rh	10.343998	17.154046	17.377887
Rh	11.106264	11.425933	19.924809
Rh	9.106928	14.705516	17.643177
Rh	9.569613	11.965839	17.739615
Rh	11.502931	10.030212	17.582103
Rh	14.196320	9.602544	17.329847
Rh	16.573432	12.169269	14.538198
Rh	12.676194	10.184369	15.075551
Rh	14.153955	11.739589	13.291410
Rh	15.364553	14.360164	13.083847
Rh	11.526679	17.252031	14.863373
Rh	13.389954	16.258493	13.127504
Rh	9.529361	13.299547	15.299588
Rh	10.986080	14.873376	13.563355
Rh	11.435347	12.158752	13.644641
Rh	16.010569	16.648308	14.438396
P	17.105050	16.580480	12.467823
C	17.528567	18.179996	11.633660
C	17.493750	19.382552	12.365587
C	17.866265	18.208614	10.264049
C	17.794519	20.600340	11.736249
C	18.157402	19.427961	9.638198
C	18.122678	20.624697	10.373188
C	15.739874	15.805776	11.483191
C	15.569973	14.455233	10.953479
C	14.600377	16.764908	11.422484
C	14.229636	14.062520	10.497587
C	13.350273	16.384802	10.831179
C	13.173867	15.029850	10.342649
C	18.698969	15.643415	12.503759
C	19.877098	16.389971	12.841416
C	18.845170	14.225438	12.291818
C	21.122459	15.773355	12.919949
C	20.131291	13.624111	12.389743
C	21.257317	14.383113	12.694170
H	17.224740	19.356904	13.425539

H	17.894888	17.282337	9.683910
H	17.766949	21.528998	12.311816
H	18.410062	19.444629	8.575128
H	18.349580	21.574044	9.881506
H	16.420738	13.834313	10.665951
H	14.846134	17.827861	11.478626
H	14.024373	12.570861	11.355948
H	12.645940	17.160543	10.519193
H	12.318113	14.822725	9.691502
H	19.796944	17.462423	13.027163
H	18.033490	13.632132	11.873457
H	22.001861	16.374669	13.162751
H	20.219637	12.551671	12.200634
H	22.239697	13.910046	12.757412

1I$_{meta-f}$

E = -551.02191 eV

Rh	13.505818	13.988194	17.151759
Rh	13.780040	14.149405	19.682157
Rh	14.042317	14.306545	22.218931
Rh	15.987693	13.141417	20.762152
Rh	15.696170	13.041488	18.143265
Rh	15.226232	15.693500	18.006238
Rh	12.548047	16.082100	18.263102
Rh	11.377679	13.664612	18.542871
Rh	13.316489	11.793507	18.464783
Rh	14.466740	11.874572	16.041216
Rh	15.652042	14.278745	15.708581
Rh	13.712542	16.171084	15.792224
Rh	11.323899	14.906786	16.144116
Rh	11.803562	12.259853	16.286815
Rh	13.232521	13.779236	14.568384
Rh	17.809921	12.075440	19.149356
Rh	16.897427	17.379597	18.929110
Rh	11.608172	18.157822	19.379166
Rh	9.280277	13.358967	19.935302
Rh	13.115046	9.603099	19.776035
Rh	15.397008	9.756169	14.974197
Rh	17.790382	14.564426	14.336649
Rh	13.879602	18.413237	14.630976
Rh	9.178070	15.850672	15.202039
Rh	10.104229	10.540469	15.493819
Rh	13.034438	13.544039	11.958118
Rh	15.528647	15.884430	20.650308
Rh	12.783242	16.267903	20.902385
Rh	11.610325	13.804512	21.177926
Rh	13.565195	11.915390	21.100422
Rh	17.457742	14.767046	19.087160
Rh	15.521226	10.775543	19.515471

Rh	16.675949	10.848317	17.007132
Rh	17.890300	13.344315	16.732051
Rh	14.288370	17.836927	19.219750
Rh	17.459783	16.004142	16.656620
Rh	15.431882	18.002869	16.724336
Rh	10.377011	15.805763	19.739585
Rh	12.763611	18.362598	16.973831
Rh	10.311589	17.067470	17.281371
Rh	11.136282	11.403498	19.917943
Rh	9.113731	14.616765	17.571168
Rh	9.604049	11.887884	17.713157
Rh	11.564523	9.981429	17.613436
Rh	14.275715	9.582309	17.381487
Rh	16.619260	12.144895	14.557262
Rh	12.755760	10.088579	15.134513
Rh	14.193055	11.640541	13.370436
Rh	15.393173	14.348966	13.010430
Rh	11.498021	17.185962	14.799079
Rh	13.402830	16.236173	13.101391
Rh	9.558106	13.170056	15.256920
Rh	11.022723	14.725754	13.517427
Rh	11.452842	12.047515	13.620328
Rh	16.011075	16.648592	14.437709
P	17.132143	16.629098	12.482507
C	17.593818	18.229715	11.669661
C	17.541046	19.435293	12.394697
C	17.966880	18.251110	10.308698
C	17.856678	20.649954	11.765506
C	18.274120	19.466636	9.683622
C	18.219163	20.667086	10.411010
C	15.742922	15.925968	11.480822
C	15.528462	14.622374	10.863618
C	14.628547	16.912253	11.473443
C	14.155710	14.289035	10.491860
C	13.347728	16.603082	10.904280
C	13.108000	15.259723	10.423580
C	18.704419	15.658119	12.492254
C	19.897818	16.368197	12.854574
C	18.818161	14.243783	12.241820
C	21.128008	15.720090	12.921697
C	20.089452	13.610031	12.327981
C	21.231598	14.334151	12.657826
H	17.245708	19.415086	13.447746
H	18.009818	17.321606	9.734418
H	17.812883	21.582142	12.334170
H	18.553147	19.477460	8.626983
H	18.455816	21.613896	9.919344
H	16.347722	14.014524	10.474070
H	14.898799	17.963563	11.595347

H	12.641939	11.712150	12.278725
H	12.660912	17.409986	10.635960
H	12.169955	15.052310	9.900638
H	19.841006	17.436675	13.070152
H	17.989963	13.682729	11.809256
H	22.019495	16.293458	13.186737
H	20.153743	12.541211	12.110700
H	22.202047	13.836384	12.713911

1TS$_{para\text{-}f}$
E = -550.38564 eV

Rh	13.480378	14.015303	17.188364
Rh	13.784300	14.143999	19.713898
Rh	14.078757	14.276128	22.238441
Rh	15.978377	13.085970	20.756899
Rh	15.655994	13.011574	18.131623
Rh	15.249570	15.666583	18.031226
Rh	12.592899	16.118573	18.322268
Rh	11.359278	13.728179	18.580759
Rh	13.251815	11.822020	18.474406
Rh	14.385463	11.893386	16.032463
Rh	15.633784	14.267455	15.722112
Rh	13.739783	16.179465	15.813146
Rh	11.319209	14.991557	16.188575
Rh	11.733171	12.329039	16.315354
Rh	13.165349	13.819993	14.600885
Rh	17.764708	11.994935	19.120554
Rh	16.960032	17.314361	18.935537
Rh	11.687981	18.202298	19.449915
Rh	9.261765	13.454622	19.992339
Rh	13.026182	9.611697	19.768905
Rh	15.259524	9.750798	14.948277
Rh	17.791356	14.539233	14.362013
Rh	13.932496	18.424772	14.639681
Rh	9.174304	15.989083	15.287183
Rh	10.003566	10.647199	15.515374
Rh	12.821191	13.688330	12.075932
Rh	15.577484	15.838100	20.667587
Rh	12.846152	16.285624	20.950611
Rh	11.625958	13.828458	21.219650
Rh	13.536637	11.893562	21.107657
Rh	17.476088	14.689264	19.066298
Rh	15.447758	10.742936	19.488842
Rh	16.595924	10.823828	16.968187
Rh	17.866996	13.296915	16.719183
Rh	14.372225	17.833047	19.263431
Rh	17.478532	15.957576	16.646408
Rh	15.485142	17.962357	16.748058
Rh	10.419836	15.876904	19.801142

Rh	12.816349	18.374801	17.017356
Rh	10.347126	17.142232	17.353957
Rh	11.084333	11.460750	19.952333
Rh	9.105687	14.722171	17.657105
Rh	9.542582	11.985913	17.751413
Rh	11.458010	10.053452	17.632886
Rh	14.169569	9.598172	17.359249
Rh	16.553351	12.139224	14.535261
Rh	12.633091	10.167101	15.116260
Rh	14.113919	11.707928	13.380439
Rh	15.420353	14.275053	13.011025
Rh	11.544370	17.251247	14.845693
Rh	13.469869	16.207250	13.134698
Rh	9.489222	13.312247	15.333645
Rh	10.918899	14.833424	13.560612
Rh	11.362265	12.164603	13.666863
Rh	16.002895	16.617194	14.419440
P	17.110297	16.595543	12.463724
C	17.521612	18.196827	11.627855
C	17.481832	19.400540	12.357004
C	17.862058	18.224154	10.258694
C	17.781759	20.617994	11.726150
C	18.151862	19.442999	9.631026
C	18.113723	20.640707	10.364107
C	15.778927	15.810112	11.462557
C	15.709691	14.494800	10.854500
C	14.603548	16.720027	11.368760
C	14.415963	14.034897	10.360021
C	13.337071	16.222188	10.897291
C	13.243286	14.846818	10.452933
C	18.719592	15.681767	12.508752
C	19.880203	16.453841	12.844259
C	18.895269	14.265342	12.311004
C	21.137160	15.863607	12.937484
C	20.191420	13.689422	12.430318
C	21.300581	14.473682	12.733286
H	17.210630	19.375836	13.416468
H	17.895513	17.297124	9.680012
H	17.751134	21.547431	12.300284
H	18.406876	19.458371	8.568462
H	18.340681	21.589499	9.871598
H	16.602654	13.971515	10.505289
H	14.797225	17.795976	11.359640
H	14.401779	13.099985	9.793704
H	12.522909	16.921154	10.688362
H	11.653784	14.376391	11.131084
H	19.778801	17.526672	13.016806
H	18.096799	13.651156	11.895568
H	22.002893	16.485733	13.176982

H	20.301509	12.616026	12.258353
H	22.291021	14.020219	12.812815

1I$_{para\text{-}f}$
E = -551.17816 eV

Rh	13.468921	14.023704	17.178941
Rh	13.806674	14.131139	19.696280
Rh	14.124126	14.250619	22.221607
Rh	16.005110	13.063583	20.708567
Rh	15.648694	13.002066	18.075826
Rh	15.251822	15.663426	18.003358
Rh	12.603452	16.120712	18.331040
Rh	11.364008	13.728380	18.600023
Rh	13.256504	11.813777	18.448598
Rh	14.340881	11.909674	15.980506
Rh	15.595541	14.274768	15.675971
Rh	13.716788	16.192433	15.789566
Rh	11.298534	15.013112	16.231695
Rh	11.712366	12.348116	16.311484
Rh	13.107417	13.859324	14.585304
Rh	17.772332	11.968484	19.034394
Rh	16.972522	17.306414	18.898208
Rh	11.725040	18.202509	19.477791
Rh	9.280782	13.447743	20.017188
Rh	13.033539	9.611540	19.735540
Rh	15.202052	9.783965	14.856360
Rh	17.746147	14.548434	14.321840
Rh	13.898206	18.443669	14.639496
Rh	9.147249	16.019982	15.334582
Rh	9.962101	10.676582	15.519793
Rh	12.768949	13.759167	12.021384
Rh	15.608467	15.826610	20.640978
Rh	12.883643	16.269788	20.952226
Rh	11.663379	13.820613	21.229701
Rh	13.565264	11.876634	21.080315
Rh	17.470417	14.670373	19.020922
Rh	15.459000	10.735741	19.443358
Rh	16.559964	10.823178	16.899411
Rh	17.848507	13.289323	16.651574
Rh	14.391491	17.830811	19.246358
Rh	17.466311	15.946075	16.606495
Rh	15.479749	17.961048	16.723489
Rh	10.438089	15.875206	19.818326
Rh	12.815985	18.376647	17.031369
Rh	10.345261	17.160419	17.386264
Rh	11.090009	11.461696	19.945718
Rh	9.099709	14.736735	17.681798
Rh	9.529116	12.009181	17.761744
Rh	11.440171	10.066569	17.620163

Rh	14.142037	9.608471	17.299556
Rh	16.511266	12.144034	14.482394
Rh	12.569515	10.176088	15.096416
Rh	14.041415	11.739039	13.356215
Rh	15.358762	14.264267	12.960628
Rh	11.509418	17.253171	14.848950
Rh	13.457270	16.240807	13.108927
Rh	9.461890	13.334655	15.357889
Rh	10.883435	14.868536	13.571670
Rh	11.312120	12.164142	13.629251
Rh	15.970016	16.631999	14.387680
P	17.127483	16.627883	12.466054
C	17.541035	18.223715	11.625757
C	17.482716	19.431965	12.346217
C	17.906261	18.242348	10.262920
C	17.789480	20.646012	11.712184
C	18.203193	19.457801	9.632336
C	18.147017	20.660225	10.356520
C	15.819432	15.820932	11.456674
C	15.807553	14.502598	10.850722
C	14.621232	16.696220	11.342441
C	14.537990	13.976370	10.377720
C	13.391243	16.152659	10.825171
C	13.340988	14.758490	10.433037
C	18.736697	15.711545	12.528469
C	19.891376	16.481713	12.890625
C	18.919711	14.295757	12.323923
C	21.147688	15.892249	12.997762
C	20.214358	13.720601	12.460657
C	21.317291	14.504133	12.785730
H	17.191946	19.413134	13.400590
H	17.954195	17.310983	9.692142
H	17.744189	21.579614	12.278314
H	18.477785	19.466792	8.574553
H	18.380417	21.606052	9.861664
H	16.723793	14.001949	10.532742
H	14.785821	17.777389	11.313388
H	14.549405	13.002730	9.881944
H	12.582119	16.826625	10.533215
H	10.924786	13.439446	12.344088
H	19.785850	17.553003	13.071141
H	18.131885	13.680027	11.891722
H	22.008956	16.513669	13.254516
H	20.327541	12.648432	12.283884
H	22.307136	14.051772	12.876057

1TS$_{ortho\text{-}e}$
E = -551.93331 eV

Rh	13.478457	13.976935	17.275934
Rh	13.594849	14.127432	19.819472
Rh	13.700768	14.277806	22.352769
Rh	15.692698	13.043175	21.029669
Rh	15.548937	12.941002	18.405326
Rh	15.203329	15.609595	18.242941
Rh	12.547927	16.110152	18.317630
Rh	11.260274	13.739889	18.507198
Rh	13.111290	11.795018	18.557300
Rh	14.425424	11.817571	16.217547
Rh	15.710793	14.179418	15.988867
Rh	13.867448	16.139377	15.910358
Rh	11.412171	14.991583	16.121049
Rh	11.767374	12.319772	16.279795
Rh	13.379652	13.775100	14.687678
Rh	17.570354	11.898909	19.530474
Rh	16.874950	17.241603	19.242163
Rh	11.615889	18.221868	19.377371
Rh	9.058558	13.513994	19.777586
Rh	12.743818	9.608550	19.844627
Rh	15.352265	9.678285	15.202150
Rh	17.985444	14.370602	14.764035
Rh	14.202749	18.343362	14.726177
Rh	9.349040	16.016546	15.067770
Rh	10.078418	10.676989	15.341853
Rh	13.299795	13.620873	12.143963
Rh	15.359909	15.789648	20.886539
Rh	12.615758	16.298758	20.957079
Rh	11.328761	13.859979	21.162794
Rh	13.215446	11.895463	21.206418
Rh	17.332251	14.600014	19.417487
Rh	15.209665	10.684536	19.741104
Rh	16.542276	10.737891	17.307854
Rh	17.866583	13.170199	17.129762
Rh	14.292032	17.802095	19.382850
Rh	17.540916	15.821253	16.963828
Rh	15.595244	17.866249	16.936042
Rh	10.271702	15.909690	19.651893
Rh	12.915609	18.357485	17.020700
Rh	10.395960	17.170790	17.211619
Rh	10.843214	11.495915	19.875169
Rh	9.087785	14.773439	17.420695
Rh	9.476949	12.023364	17.563737
Rh	11.356433	10.060103	17.574821
Rh	14.062426	9.540734	17.530536
Rh	16.689907	12.014861	14.863121
Rh	12.729033	10.124535	15.166546
Rh	14.332116	11.634323	13.540896
Rh	15.732869	14.206633	13.162948

Rh	11.749910	17.255223	14.804133
Rh	13.642425	16.119300	13.231145
Rh	9.619083	13.340242	15.139502
Rh	11.221421	14.790080	13.492239
Rh	11.628343	12.150079	13.608098
Rh	16.239765	16.504924	14.604899
P	17.244491	16.684886	12.582040
C	17.471775	18.354553	11.823572
C	17.690904	19.462308	12.667344
C	17.420465	18.551264	10.426151
C	17.863473	20.743367	12.122692
C	17.588257	19.834484	9.886551
C	17.810331	20.931667	10.733558
C	15.918378	15.847918	11.614946
C	15.932688	14.526071	11.013430
C	14.706236	16.692309	11.501211
C	14.702656	14.020823	10.402792
C	13.507991	16.158131	10.914661
C	13.507900	14.822667	10.342496
C	18.817870	15.785445	12.258150
C	19.846154	16.255922	11.419084
C	18.994219	14.557690	12.959546
C	21.003842	15.486635	11.222078
C	20.131467	13.760845	12.700104
C	21.138590	14.236602	11.848486
H	17.712399	19.316189	13.750706
H	17.242780	17.704649	9.757848
H	18.030110	21.595733	12.785949
H	17.541965	19.977170	8.803910
H	17.935523	21.931424	10.310484
H	16.876412	14.058512	10.722287
H	14.841902	17.776086	11.526517
H	14.778041	13.127699	9.778354
H	12.695406	16.837426	10.650796
H	12.703327	14.549170	9.654827
H	19.750703	17.220896	10.918265
H	17.633680	13.841960	13.099893
H	21.794622	15.859752	10.567006
H	20.238829	12.790002	13.189784
H	22.034204	13.632664	11.679384

$1I_{ortho\text{-}e}$
E = -552.70182 eV

Rh	13.473821	13.989785	17.272393
Rh	13.618031	14.133586	19.812023
Rh	13.739254	14.269387	22.344895
Rh	15.728814	13.042230	20.996297
Rh	15.556625	12.944711	18.371033
Rh	15.220305	15.609142	18.225542

Rh	12.566924	16.126883	18.323189
Rh	11.274936	13.756388	18.516596
Rh	13.117634	11.801084	18.539206
Rh	14.420349	11.832780	16.190496
Rh	15.727083	14.191023	15.978396
Rh	13.876422	16.153412	15.901089
Rh	11.413589	15.015664	16.132052
Rh	11.770922	12.341877	16.273344
Rh	13.357347	13.807328	14.684352
Rh	17.584549	11.889526	19.485632
Rh	16.907521	17.235793	19.198823
Rh	11.646370	18.229385	19.390976
Rh	9.076509	13.532889	19.786535
Rh	12.763268	9.621601	19.833823
Rh	15.325345	9.680335	15.178548
Rh	17.986952	14.413518	14.745279
Rh	14.216949	18.352798	14.724226
Rh	9.343302	16.047465	15.091412
Rh	10.068919	10.706336	15.337078
Rh	13.248958	13.661569	12.125852
Rh	15.394469	15.782796	20.871889
Rh	12.658240	16.304408	20.962822
Rh	11.354189	13.869934	21.169433
Rh	13.240779	11.897696	21.186011
Rh	17.354234	14.594003	19.384842
Rh	15.224233	10.689722	19.708880
Rh	16.546179	10.734833	17.265962
Rh	17.878594	13.164259	17.067415
Rh	14.329254	17.801327	19.368417
Rh	17.554868	15.837668	16.941020
Rh	15.612976	17.870400	16.917594
Rh	10.296726	15.919051	19.666630
Rh	12.925958	18.365418	17.025232
Rh	10.407557	17.190501	17.223357
Rh	10.857418	11.509960	19.879748
Rh	9.097943	14.797801	17.429802
Rh	9.489469	12.047177	17.573084
Rh	11.362144	10.075667	17.564507
Rh	14.078489	9.552546	17.507522
Rh	16.715551	12.038067	14.840614
Rh	12.716111	10.132271	15.158147
Rh	14.308624	11.646855	13.510423
Rh	15.694035	14.175282	13.173209
Rh	11.759932	17.274176	14.805897
Rh	13.653396	16.133609	13.221767
Rh	9.617841	13.368261	15.147892
Rh	11.211522	14.821066	13.486725
Rh	11.618708	12.174282	13.603040
Rh	16.249050	16.474938	14.578863

P	17.283027	16.665721	12.570458
C	17.464450	18.351338	11.830159
C	17.725229	19.441538	12.685727
C	17.375804	18.575245	10.438953
C	17.904465	20.728929	12.158682
C	17.552071	19.864613	9.916284
C	17.818184	20.942599	10.774682
C	15.940004	15.824610	11.626873
C	15.941950	14.501342	11.034899
C	14.736024	16.683149	11.498408
C	14.709011	14.012445	10.408519
C	13.540112	16.166426	10.892828
C	13.530916	14.833194	10.317106
C	18.883562	15.825785	12.270809
C	19.780551	16.177180	11.240550
C	19.204489	14.780682	13.180446
C	20.981319	15.470615	11.087923
C	20.413298	14.071008	12.999749
C	21.293182	14.416292	11.963121
H	17.776823	19.277670	13.765410
H	17.163410	17.745317	9.760004
H	18.104027	21.566373	12.831568
H	17.479713	20.026052	8.837627
H	17.952659	21.947122	10.365278
H	16.883789	14.011562	10.775583
H	14.884276	17.764834	11.524413
H	14.777726	13.108334	9.799184
H	12.739536	16.856661	10.621035
H	12.736445	14.569404	9.614075
H	19.553197	17.003331	10.564338
H	16.054395	12.299623	13.140759
H	21.673355	15.744226	10.287905
H	20.673963	13.251734	13.675605
H	22.229854	13.864581	11.841215

4A
E = -600.76199 eV

Rh	14.208521	12.846293	12.446572
Rh	14.192741	12.403659	14.990618
Rh	14.123303	11.760419	17.446100
Rh	15.928335	10.594319	15.856219
Rh	15.956305	11.172254	13.291455
Rh	16.136589	13.851605	13.844215
Rh	13.644823	14.879901	14.048970
Rh	11.904299	12.797148	13.591111
Rh	13.332384	10.542684	13.138051
Rh	14.779934	10.901881	10.871567
Rh	16.498937	12.928896	11.302902
Rh	15.033854	15.179337	11.754181

Rh	12.426557	14.534445	11.615449
Rh	12.266110	11.897356	11.061008
Rh	14.213315	13.373011	9.942846
Rh	17.684378	9.522337	14.113687
Rh	18.071441	14.772758	15.169218
Rh	13.046942	16.790978	15.594890
Rh	9.605217	12.673886	14.672867
Rh	12.481476	8.242049	13.800465
Rh	15.355307	8.999410	9.312557
Rh	18.776430	13.035661	10.174987
Rh	15.908764	17.470795	11.041939
Rh	10.691753	16.169604	10.718249
Rh	10.359107	10.952642	9.662658
Rh	14.257244	13.916842	7.434693
Rh	16.140398	13.269028	16.491755
Rh	13.506683	14.256610	16.660596
Rh	11.808817	12.187180	16.151591
Rh	13.283177	9.942092	15.664466
Rh	17.937071	12.140509	14.730536
Rh	15.135610	8.766797	13.949853
Rh	16.603148	9.167189	11.675248
Rh	18.339839	11.241891	12.134539
Rh	15.623511	15.851978	15.730444
Rh	18.515354	13.912095	12.713253
Rh	17.011961	16.220463	13.219163
Rh	11.247636	14.763669	15.155743
Rh	14.467040	17.227865	13.370345
Rh	11.774468	16.546158	13.171401
Rh	10.936858	10.415551	14.236048
Rh	10.027365	14.471737	12.710949
Rh	9.879526	11.802376	12.153747
Rh	11.379629	9.494227	11.668883
Rh	13.893089	8.516500	11.511418
Rh	17.149221	10.981181	9.660907
Rh	12.833930	9.936980	9.364215
Rh	14.838628	11.435892	8.265270
Rh	17.412740	15.331155	10.642790
Rh	16.585103	13.508292	8.734368
Rh	13.299819	16.952307	10.911884
Rh	15.103116	15.760278	9.177478
Rh	10.454457	13.599427	10.122522
Rh	12.447691	15.120822	9.029224
Rh	12.265316	12.428084	8.446292
P	16.055957	17.152617	17.535137
C	14.504939	18.179261	17.593124
C	17.518172	18.334422	17.491060
C	16.181374	16.487530	19.288203
C	13.201254	17.511619	17.675881
C	14.545643	19.556920	17.325606

C	17.856707	19.151792	16.365528
C	18.381272	18.352874	18.613012
C	16.862038	15.298964	19.672729
C	15.587828	17.281952	20.297149
C	12.065312	18.257254	17.197875
C	12.925494	16.303950	18.560826
C	13.385650	20.289582	16.984081
C	19.017848	19.950170	16.444645
C	17.081146	19.198447	15.073588
C	19.536433	19.142798	18.655755
C	16.867287	14.937634	21.038523
C	17.602976	14.393306	18.720690
C	15.616050	16.911820	21.646186
C	12.167592	19.629530	16.847789
C	19.857233	19.953952	17.563493
C	16.251397	15.720570	22.019458
H	15.505291	20.075273	17.354534
H	18.152118	17.740063	19.482318
H	15.099962	18.218570	20.020079
H	11.070516	17.819262	17.319604
H	11.904473	15.932157	18.401758
H	13.658762	15.465900	18.419279
H	13.025674	16.565078	19.629050
H	13.461902	21.361388	16.787684
H	19.267289	20.581705	15.587492
H	17.477948	19.976976	14.406606
H	17.153983	18.221906	14.541753
H	16.005612	19.375118	15.211604
H	20.172355	19.120823	19.543683
H	17.384488	14.019468	21.331363
H	16.912300	13.566055	18.370447
H	18.010655	14.923855	17.848218
H	18.429775	13.869581	19.222310
H	15.147297	17.553631	22.396059
H	11.273635	20.165853	16.520801
H	20.750933	20.582720	17.576181
H	16.280163	15.409153	23.066433

4TS$_{methyl}$

E = -600.39675 eV

Rh	14.193802	12.891738	12.438622
Rh	14.147573	12.513455	14.998213
Rh	14.046012	11.981279	17.463234
Rh	15.842671	10.716653	15.922370
Rh	15.913089	11.216265	13.330073
Rh	16.128427	13.910029	13.806360
Rh	13.646200	14.952431	13.978380
Rh	11.882725	12.908408	13.557848
Rh	13.279425	10.617343	13.176838

Rh	14.738446	10.893916	10.920350
Rh	16.489870	12.910355	11.302683
Rh	15.064548	15.194081	11.675090
Rh	12.446832	14.587232	11.533007
Rh	12.246953	11.940759	11.061692
Rh	14.223802	13.349008	9.918302
Rh	17.607138	9.556034	14.212424
Rh	18.076472	14.827608	15.118144
Rh	13.073033	16.909958	15.475418
Rh	9.566560	12.868950	14.628207
Rh	12.387773	8.360835	13.905740
Rh	15.285138	8.931851	9.419682
Rh	18.773656	12.941636	10.188056
Rh	15.991443	17.439135	10.908560
Rh	10.745938	16.212404	10.580060
Rh	10.325621	10.982876	9.688997
Rh	14.297286	13.803834	7.391680
Rh	16.105520	13.392535	16.460913
Rh	13.495944	14.541074	16.692534
Rh	11.767895	12.432178	16.149334
Rh	13.175261	10.134117	15.743865
Rh	17.880894	12.187192	14.770828
Rh	15.035968	8.846483	14.066496
Rh	16.522801	9.140380	11.794424
Rh	18.292435	11.197669	12.196780
Rh	15.660753	15.951637	15.652821
Rh	18.518912	13.881869	12.695500
Rh	17.059019	16.235916	13.132317
Rh	11.221822	14.959660	15.056092
Rh	14.529158	17.273464	13.225919
Rh	11.854745	16.659565	13.028439
Rh	10.876581	10.595468	14.292463
Rh	10.052065	14.591246	12.637775
Rh	9.844988	11.912398	12.139665
Rh	11.309105	9.571114	11.760637
Rh	13.806509	8.530853	11.627922
Rh	17.113745	10.886690	9.736433
Rh	12.785053	9.901543	9.450142
Rh	14.818049	11.340751	8.301284
Rh	17.442588	15.269421	10.569295
Rh	16.601305	13.380243	8.712372
Rh	13.363645	16.965253	10.755187
Rh	15.174096	15.679772	9.072308
Rh	10.468510	13.635607	10.060577
Rh	12.495975	15.090487	8.933288
Rh	12.274339	12.372363	8.435849
P	16.086310	17.158688	17.525706
C	14.487314	18.097518	17.600142
C	17.508989	18.385162	17.522535

C	16.211170	16.474347	19.275342
C	13.224019	17.349378	17.630836
C	14.456966	19.492367	17.434037
C	17.840720	19.216832	16.406209
C	18.365519	18.404387	18.649729
C	16.934696	15.313302	19.664415
C	15.563027	17.233546	20.279111
C	12.038950	18.079424	17.241431
C	13.032388	16.005454	18.304739
C	13.258175	20.188414	17.163697
C	18.998433	20.019095	16.492047
C	17.056281	19.271598	15.119886
C	19.515372	19.201649	18.700608
C	16.924730	14.939121	21.026754
C	17.730725	14.451638	18.717419
C	15.580385	16.853495	21.625840
C	12.067519	19.479050	17.010540
C	19.837022	20.017501	17.611754
C	16.256296	15.685934	22.001999
H	15.392431	20.051843	17.489409
H	18.137235	17.784868	19.514716
H	15.038974	18.150192	20.002236
H	11.070786	17.579907	17.332825
H	11.968206	15.775802	18.440102
H	13.889110	14.747201	18.226538
H	13.508720	16.008600	19.297841
H	13.277878	21.273585	17.041602
H	19.246934	20.656771	15.639125
H	17.437442	20.066677	14.463326
H	17.137272	18.306690	14.572964
H	15.980549	19.434284	15.271725
H	20.147983	19.179778	19.591201
H	17.472040	14.039548	21.322686
H	17.081387	13.627131	18.301930
H	18.161455	15.015314	17.878316
H	18.547150	13.930083	19.238673
H	15.070428	17.469608	22.370464
H	11.139751	19.994174	16.750772
H	20.730668	20.646267	17.628565
H	16.275501	15.365601	23.046499

$4I_{methyl}$

E = -601.25511 eV

Rh	14.108302	12.834921	12.463627
Rh	13.886214	12.360803	14.984258
Rh	13.637238	11.843868	17.453091
Rh	15.455473	10.518523	15.995080
Rh	15.670722	11.033901	13.407633
Rh	16.018944	13.690834	13.974097

Rh	13.633356	14.893725	14.028345
Rh	11.746975	12.991430	13.459128
Rh	13.005187	10.613724	13.102204
Rh	14.606489	10.838961	10.935334
Rh	16.465412	12.734001	11.465849
Rh	15.180907	15.087347	11.827172
Rh	12.541381	14.667244	11.516327
Rh	12.179867	12.045560	10.967986
Rh	14.310310	13.339631	9.961890
Rh	17.186567	9.246623	14.346944
Rh	17.952911	14.450392	15.429232
Rh	13.077967	16.871665	15.536998
Rh	9.394331	13.094541	14.430981
Rh	11.920907	8.401581	13.727836
Rh	15.092270	8.860142	9.436179
Rh	18.803338	12.623195	10.474750
Rh	16.285618	17.281654	11.129314
Rh	11.002914	16.424914	10.522219
Rh	10.273533	11.236939	9.488555
Rh	14.546311	13.840112	7.472257
Rh	15.834066	13.167095	16.586540
Rh	13.343405	14.503329	16.699667
Rh	11.493877	12.513863	16.056072
Rh	12.751091	10.084716	15.685638
Rh	17.623509	11.861568	14.971819
Rh	14.575783	8.731701	14.074822
Rh	16.204484	8.942175	11.872480
Rh	18.097128	10.873079	12.411299
Rh	15.649993	15.839739	15.740581
Rh	18.463789	13.561233	12.971247
Rh	17.170262	15.962360	13.370264
Rh	11.179860	15.038393	15.003663
Rh	14.683352	17.183132	13.374183
Rh	12.021848	16.748883	13.025489
Rh	10.577938	10.739602	14.133120
Rh	10.087789	14.803684	12.511133
Rh	9.728327	12.137393	11.937812
Rh	11.034932	9.733951	11.590712
Rh	13.480595	8.520726	11.554958
Rh	17.026394	10.678001	9.895753
Rh	12.656485	9.981834	9.373972
Rh	14.826444	11.318053	8.336489
Rh	17.613770	15.029858	10.814282
Rh	16.740428	13.205926	8.892301
Rh	13.646724	16.982015	10.835096
Rh	15.447608	15.617650	9.236312
Rh	10.556800	13.872582	9.955584
Rh	12.735000	15.188976	8.930842
Rh	12.369140	12.517722	8.368079

P	16.115359	17.189813	17.524849
C	14.490517	18.079280	17.656574
C	17.441345	18.526313	17.463677
C	16.400706	16.490581	19.239434
C	13.243439	17.308806	17.713916
C	14.431498	19.474079	17.503025
C	17.734116	19.315432	16.308082
C	18.226754	18.726348	18.624244
C	17.422258	15.547068	19.550840
C	15.577769	16.968428	20.284101
C	12.040521	18.018883	17.349431
C	13.124727	15.920382	18.251737
C	13.214179	20.149922	17.263962
C	18.806848	20.230369	16.374092
C	16.965238	19.243974	15.014112
C	19.285404	19.641996	18.662183
C	17.518181	15.076735	20.876922
C	18.442968	15.063479	18.549025
C	15.704571	16.500634	21.598138
C	12.034408	19.419736	17.132239
C	19.585748	20.395847	17.523841
C	16.670909	15.533113	21.893823
H	15.356715	20.051431	17.537826
H	18.011449	18.158795	19.527544
H	14.826835	17.730604	20.070790
H	11.086202	17.493969	17.445705
H	12.110899	15.719711	18.629892
H	14.530268	13.549458	17.808586
H	13.881999	15.660118	18.994534
H	13.211916	21.235979	17.148635
H	19.029859	20.828142	15.486062
H	17.384104	19.937272	14.271275
H	16.996416	18.218956	14.586654
H	15.899433	19.486351	15.145928
H	19.865330	19.757746	19.581102
H	18.292679	14.342148	21.113974
H	17.940315	14.485759	17.733050
H	18.988969	15.897728	18.082310
H	19.174019	14.390832	19.017843
H	15.048762	16.893786	22.378542
H	11.091903	19.919100	16.895888
H	20.412957	21.109820	17.528158
H	16.777245	15.145613	22.909967

4TS$_{meta}$
E = -598.82226 eV

Rh	13.974673	12.924982	12.431568
Rh	13.974286	12.436833	14.964635
Rh	13.973585	11.753727	17.427768

Rh	15.811794	10.693364	15.804942
Rh	15.768185	11.291333	13.243371
Rh	15.876248	13.957478	13.838110
Rh	13.385864	14.941917	14.086497
Rh	11.687537	12.803787	13.599559
Rh	13.162922	10.594772	13.103858
Rh	14.577814	11.018480	10.832595
Rh	16.255040	13.081516	11.277371
Rh	14.729537	15.287711	11.772017
Rh	12.141795	14.577497	11.655244
Rh	12.044768	11.950483	11.059332
Rh	13.937928	13.484766	9.938648
Rh	17.562047	9.681071	14.032867
Rh	17.828117	14.883702	15.143071
Rh	12.589222	16.881201	15.561368
Rh	9.414328	12.590234	14.727971
Rh	12.379783	8.265702	13.752213
Rh	15.174738	9.148905	9.248328
Rh	18.508901	13.286733	10.117558
Rh	15.519469	17.599818	11.075192
Rh	10.344066	16.165066	10.782893
Rh	10.154152	10.941431	9.659400
Rh	13.936608	14.046425	7.433406
Rh	15.946006	13.332774	16.433409
Rh	13.320155	14.284526	16.670992
Rh	11.637549	12.182534	16.174139
Rh	13.188999	9.970081	15.646940
Rh	17.755661	12.286705	14.659951
Rh	15.008458	8.852754	13.887232
Rh	16.472468	9.336124	11.612726
Rh	18.147964	11.433197	12.062007
Rh	15.453884	15.928099	15.866345
Rh	18.248077	14.111177	12.682349
Rh	16.629897	16.376473	13.261629
Rh	10.972712	14.721772	15.202637
Rh	14.076006	17.327519	13.366964
Rh	11.384458	16.524873	13.237283
Rh	10.806905	10.376019	14.243576
Rh	9.741002	14.404172	12.743322
Rh	9.681454	11.743520	12.188003
Rh	11.223482	9.517461	11.651014
Rh	13.785898	8.596617	11.463655
Rh	16.936111	11.185996	9.597921
Rh	12.651806	10.000449	9.345413
Rh	14.601272	11.586451	8.232596
Rh	17.084871	15.521924	10.636814
Rh	16.289587	13.701071	8.712954
Rh	12.897632	16.997125	10.929056
Rh	14.739626	15.905710	9.198313

Rh	10.170699	13.582032	10.164976
Rh	12.103280	15.158805	9.055927
Rh	12.000113	12.480572	8.450184
P	16.264430	17.058911	17.685752
C	14.844275	18.240015	17.777999
C	17.822234	18.096719	17.568225
C	16.395050	16.343861	19.411668
C	13.513659	17.673165	17.639893
C	14.979763	19.634182	17.638961
C	18.157213	18.904177	16.435149
C	18.723433	18.063284	18.659102
C	17.125575	15.169435	19.752511
C	15.743710	17.060374	20.441321
C	12.607978	18.454258	16.853898
C	13.026596	16.493304	18.461420
C	13.909744	20.443530	17.201800
C	19.370660	19.624488	16.464431
C	17.315075	19.027970	15.191029
C	19.925866	18.780165	18.654378
C	17.123056	14.745595	21.098642
C	17.921012	14.356292	18.763114
C	15.765671	16.627121	21.772421
C	12.758545	19.833911	16.670310
C	20.254587	19.568624	17.546798
C	16.450377	15.451200	22.102306
H	15.949718	20.097763	17.831172
H	18.482765	17.470699	19.540224
H	15.219018	17.986106	20.196920
H	11.384625	17.948422	15.695440
H	11.962012	16.298683	18.277394
H	13.633750	15.551988	18.307264
H	13.151420	16.696562	19.540034
H	14.040555	21.526661	17.136704
H	19.621524	20.246365	15.600500
H	17.662317	19.860402	14.562694
H	17.392995	18.095762	14.578916
H	16.246062	19.164404	15.394969
H	20.592374	18.722195	19.518198
H	17.679977	13.841234	21.360019
H	17.257348	13.650177	18.201353
H	18.437452	14.974117	18.014636
H	18.670327	13.732072	19.271523
H	15.255455	17.211146	22.542217
H	12.053323	20.405300	16.062494
H	21.186490	20.138834	17.523526
H	16.475846	15.092845	23.134175

$4I_{meta}$

E = -599.45385 eV

Rh	13.997525	12.928597	12.431616
Rh	14.020021	12.426463	14.952029
Rh	14.019735	11.743358	17.426321
Rh	15.835772	10.682546	15.802696
Rh	15.788918	11.284115	13.225953
Rh	15.919942	13.952497	13.817547
Rh	13.434062	14.911383	14.115731
Rh	11.713319	12.831963	13.622940
Rh	13.168702	10.606241	13.110068
Rh	14.564857	11.029365	10.823431
Rh	16.266664	13.087040	11.258957
Rh	14.765268	15.294000	11.794848
Rh	12.180705	14.604522	11.678341
Rh	12.042814	11.987996	11.077764
Rh	13.949075	13.516044	9.943193
Rh	17.567335	9.651173	13.992845
Rh	17.855090	14.890071	15.150161
Rh	12.759929	16.844496	15.710274
Rh	9.445554	12.633220	14.776645
Rh	12.386690	8.281563	13.749245
Rh	15.143837	9.174375	9.225045
Rh	18.516333	13.262396	10.098809
Rh	15.558954	17.620795	11.134012
Rh	10.376624	16.224612	10.839843
Rh	10.130032	11.008823	9.701533
Rh	13.928440	14.100726	7.453363
Rh	15.968510	13.331558	16.426837
Rh	13.334551	14.300591	16.670042
Rh	11.696715	12.185603	16.194198
Rh	13.208881	9.970075	15.655927
Rh	17.776433	12.264188	14.644137
Rh	15.006123	8.855125	13.884439
Rh	16.444710	9.327464	11.588732
Rh	18.157451	11.423464	12.041636
Rh	15.460460	15.911642	15.818944
Rh	18.271103	14.094509	12.664557
Rh	16.717089	16.377279	13.241129
Rh	11.033042	14.756500	15.259154
Rh	14.161654	17.330260	13.431941
Rh	11.469649	16.557571	13.334129
Rh	10.824277	10.410967	14.276462
Rh	9.792264	14.485615	12.794353
Rh	9.685148	11.801391	12.242812
Rh	11.199903	9.567709	11.673355
Rh	13.758569	8.618328	11.449835
Rh	16.919030	11.184905	9.585696
Rh	12.621119	10.032152	9.354485
Rh	14.571163	11.616497	8.230355
Rh	17.108929	15.524815	10.637537

Rh	16.288637	13.723599	8.699906
Rh	12.939743	17.027216	10.977523
Rh	14.762926	15.927210	9.227310
Rh	10.176613	13.643046	10.208606
Rh	12.108382	15.224941	9.096970
Rh	11.987297	12.540531	8.480700
P	16.224092	17.052962	17.656951
C	14.782041	18.196804	17.785075
C	17.779573	18.096281	17.556990
C	16.373417	16.310384	19.373523
C	13.438362	17.599382	17.724331
C	14.877157	19.586258	17.631771
C	18.119221	18.915581	16.434659
C	18.682481	18.043454	18.646271
C	17.086153	15.122018	19.701585
C	15.758738	17.038885	20.417448
C	12.491289	18.364987	16.971555
C	12.972562	16.534312	18.708146
C	13.759241	20.376995	17.255504
C	19.337917	19.626856	16.470677
C	17.279067	19.056394	15.191669
C	19.889279	18.752673	18.649456
C	17.089987	14.690135	21.045414
C	17.864723	14.301531	18.705444
C	15.787198	16.598911	21.745914
C	12.586250	19.756272	16.799643
C	20.222836	19.551574	17.550935
C	16.444744	15.403870	22.061067
H	15.846115	20.075286	17.753846
H	18.440124	17.441504	19.520252
H	15.253546	17.977972	20.184594
H	12.471640	17.903135	13.961759
H	11.924565	16.271993	18.512374
H	13.594314	15.614103	18.685622
H	13.038066	16.916594	19.743441
H	13.875416	21.461647	17.174576
H	19.592222	20.257113	15.613723
H	17.591962	19.930237	14.602386
H	17.404198	18.157277	14.539968
H	16.203512	19.132988	15.392455
H	20.555324	18.680688	19.512611
H	17.630998	13.772967	21.295301
H	17.197432	13.562672	18.184473
H	18.351275	14.911434	17.931059
H	18.637621	13.697432	19.203203
H	15.300412	17.191384	22.524385
H	11.827912	20.321171	16.251354
H	21.158768	20.115399	17.533559
H	16.471683	15.037358	23.090116

4Ad
E = -2285.4574 eV

Rh	10.020457	14.777422	15.931906
Rh	10.235010	16.963754	14.416736
Rh	10.544816	12.620478	17.448486
Rh	11.046592	17.220380	16.876431
Rh	11.213706	15.012550	18.450377
Rh	11.033483	14.664299	13.344660
Rh	11.180725	12.444983	14.900681
Rh	12.064469	17.440199	19.322338
Rh	12.037787	12.321796	12.405991
Rh	12.300303	18.675686	14.864292
Rh	12.220900	17.109583	12.603198
Rh	12.527947	16.000658	15.053094
Rh	12.657419	12.676064	19.158791
Rh	12.625496	13.794761	16.578365
Rh	12.773032	11.156790	16.820082
Rh	13.229262	18.865984	17.449369
Rh	13.450873	15.253241	20.127112
Rh	13.163485	14.608205	11.571890
Rh	13.427459	16.178676	17.591409
Rh	13.431403	13.667794	14.036551
Rh	13.466361	10.999907	14.198712
Rh	14.550254	20.211941	15.514000
Rh	14.526428	18.631185	13.166031
Rh	14.746762	17.616117	19.372871
Rh	14.622018	17.608088	15.615690
Rh	14.503316	16.990643	10.883059
Rh	14.705718	16.000667	13.281683
Rh	14.772205	14.976015	15.770848
Rh	14.869909	13.942807	18.114590
Rh	14.997155	13.056150	20.473505
Rh	14.920919	12.364692	15.893288
Rh	14.787429	12.355750	12.125479
Rh	14.983492	9.775018	16.080522
Rh	15.066848	11.280310	18.413977
Rh	16.028480	18.971214	17.369749
Rh	16.098910	16.319459	17.502387
Rh	16.118103	13.837326	13.876779
Rh	16.311385	15.375112	20.006943
Rh	16.141191	14.737932	11.147077
Rh	16.311483	11.106169	14.081966
Rh	16.800443	18.875594	14.693688
Rh	16.847031	17.193491	12.461792
Rh	16.882467	16.225209	14.941258
Rh	17.061461	14.007315	16.430765
Rh	17.215349	12.927339	18.938014
Rh	17.252155	11.301678	16.701369

Rh	17.430193	17.700121	19.175204
Rh	17.475821	12.554191	12.187863
Rh	18.317071	17.615819	16.662313
Rh	18.474530	15.347968	18.214335
Rh	18.411180	14.980418	13.094091
Rh	18.527035	12.765789	14.656203
Rh	18.997795	17.339502	14.128179
Rh	19.288210	13.054493	17.171792
Rh	19.591143	15.260810	15.731074
N	14.623540	23.163347	14.475525
N	14.601096	23.132727	16.652716
N	14.934641	6.820160	15.052317
N	14.917775	6.864496	17.231281
N	22.579643	14.672118	15.230994
N	22.343118	15.919257	17.010336
N	7.121890	15.419800	16.780399
N	7.137273	14.088146	15.044507
P	16.581869	14.871326	8.829491
C	10.977532	21.907090	12.645878
C	10.923940	21.869910	18.472121
C	11.196718	8.056091	18.902549
C	11.240288	7.965549	13.361499
C	11.502313	24.381653	12.856436
C	11.517345	24.328635	18.310210
C	11.805681	5.489480	13.396309
C	11.793172	5.589242	18.886427
C	12.041059	22.942758	13.073267
C	12.014874	22.880751	18.056259
C	12.326771	6.920013	13.692760
C	12.298216	7.023211	18.576431
C	13.392632	22.760518	12.369560
C	13.436423	22.567287	10.976018
C	13.366738	22.647618	18.744045
C	13.412672	22.368106	20.122918
C	13.655955	7.172198	12.969470
C	13.626398	7.294419	19.296036
C	14.807322	11.772687	6.507662
C	13.662556	7.393162	11.579136
C	13.627863	7.561022	20.678279
C	13.728437	13.018346	8.354939
C	15.815806	11.464888	5.592973
C	14.937564	12.789197	7.481552
C	14.602130	24.491411	14.902745
C	14.634398	22.858457	13.052330
C	14.588739	24.471957	16.261732
C	14.652280	22.496287	10.290195
C	14.626066	22.274858	15.551309
C	14.607533	22.782196	18.065702
C	14.628257	22.252390	20.802267

C	14.859028	7.539492	10.873530
C	14.923626	7.715988	16.123407
C	14.912253	7.122563	13.628772
C	14.886236	7.215928	18.645194
C	14.821803	7.721505	21.385327
C	14.931474	5.495367	15.488573
C	14.919511	5.523728	16.847677
C	16.996654	12.213509	5.622153
C	15.858524	22.626556	10.983686
C	15.883358	22.819093	12.377774
C	16.160443	13.523546	7.546997
C	15.855431	22.719029	18.740159
C	15.832345	22.439580	20.119706
C	16.082692	7.447777	11.542150
C	16.048314	7.598905	20.727042
C	16.144706	7.228794	12.931058
C	16.115951	7.334685	19.346426
C	17.152327	13.221114	6.579847
C	13.510815	17.136002	7.213346
C	14.562719	16.192021	7.362948
C	13.418592	18.197409	8.096278
C	15.572938	16.345331	8.316316
C	17.221637	23.057281	13.089428
C	17.188733	23.025978	18.046404
C	14.453765	18.419616	9.055406
C	15.635910	17.590301	9.107622
C	17.504154	7.041496	13.617653
C	17.479449	7.116739	18.676390
C	17.732550	24.498006	12.824914
C	17.646394	24.475675	18.357660
C	16.944211	18.286857	9.468049
C	18.006771	5.582768	13.451273
C	17.981362	5.667522	18.912273
C	19.291269	13.097031	9.341697
C	18.308501	22.026004	12.713444
C	18.311209	22.028007	18.399878
C	18.585908	8.029192	13.127779
C	18.557287	8.128640	19.125813
C	18.333198	15.250297	8.292072
C	19.439677	14.410540	8.614130
C	18.550226	16.366368	7.456166
C	20.716289	14.777939	8.141454
C	19.826997	16.708869	6.993837
C	20.923227	15.916037	7.352410
C	21.850289	16.741969	11.613610
C	21.205330	13.932654	20.679403
C	21.365653	20.119513	16.389470
C	21.582767	15.287453	16.007210
C	21.656997	10.448686	15.433454

C	22.697577	12.405010	11.652947
C	21.327102	18.313954	20.366408
C	22.719800	13.800304	11.599376
C	21.491359	16.935528	20.517891
C	22.597837	11.753574	12.884761
C	21.522617	18.908997	19.116871
C	22.628383	14.579421	12.767501
C	21.814980	16.109825	19.423404
C	21.846505	18.142541	17.981228
C	22.512095	12.473996	14.090801
C	22.493917	13.892796	14.003136
C	21.927981	16.731639	18.147480
C	22.777820	16.103643	12.670421
C	22.145562	14.629126	19.670604
C	22.196536	18.846926	16.661302
C	22.539645	11.716663	15.426150
C	24.253420	16.496080	12.396032
C	23.856719	14.929297	15.729976
C	23.708928	15.699796	16.833874
C	23.612891	14.471155	20.154782
C	23.705060	19.212756	16.610746
C	23.991109	11.339162	15.825605
C	5.670833	15.441362	11.989980
C	5.585170	18.741204	16.223411
C	5.828055	13.951229	19.842919
C	5.793493	15.144662	16.456022
C	5.803103	14.318069	15.380785
C	5.789227	10.699753	15.659104
C	7.176289	15.228613	12.304810
C	7.081745	18.337439	16.306637
C	7.309519	14.256568	19.492849
C	7.259756	11.183212	15.543281
C	7.402809	16.308632	17.901719
C	7.435040	13.205937	13.922128
C	7.397969	17.712527	17.673399
C	7.465781	13.750932	12.608288
C	7.502459	15.752637	19.206806
C	7.512383	11.806727	14.163292
C	7.595552	18.550371	18.788091
C	7.682820	12.856160	11.542503
C	7.698030	16.645915	20.277631
C	7.727063	10.967222	13.053382
C	7.759781	18.026252	20.072405
C	7.829001	11.484277	11.759948
C	8.021509	15.806619	11.147937
C	8.010145	14.765720	15.911729
C	7.964388	19.556424	15.957095
C	8.222534	13.711295	20.613225
C	8.216038	10.021139	15.890366

H	10.074145	22.028678	13.264991
H	10.024516	22.022994	17.854692
H	10.308411	7.859369	18.280861
H	10.343864	7.777430	13.973748
H	10.556771	24.521004	13.405475
H	10.671112	22.035272	11.595556
H	10.569267	24.505227	17.776568
H	10.622734	21.992954	19.524524
H	10.588181	17.706923	12.806315
H	10.881704	5.293706	13.964420
H	10.932591	7.927129	12.304564
H	10.876444	8.003391	19.955055
H	10.867127	5.381144	18.325894
H	11.046937	11.967730	19.028884
H	11.340069	20.878140	12.774808
H	11.304029	24.570695	11.789022
H	11.256050	20.829956	18.327242
H	11.338114	24.498610	19.384147
H	11.532062	9.083247	18.701590
H	11.584331	8.990177	13.579985
H	11.577156	5.365834	12.325463
H	11.571722	5.473975	19.959632
H	11.593329	15.571387	11.779266
H	11.923317	14.164261	19.983419
H	12.203415	22.805063	14.152398
H	12.164382	22.762146	16.972741
H	12.211235	25.146902	13.206296
H	12.446072	16.473736	20.830280
H	12.241512	25.082533	17.967981
H	12.050336	13.356033	10.958742
H	12.502493	22.480977	10.417274
H	12.512161	6.988914	14.774479
H	12.482568	7.085537	17.493757
H	12.480394	22.242838	20.676385
H	12.541343	4.718856	13.672153
H	13.863866	11.219442	6.471722
H	12.532857	4.821686	18.613239
H	12.752473	20.020418	16.058141
H	13.696422	12.291438	9.181744
H	12.679347	18.844955	13.016960
H	12.714615	7.448539	11.040088
H	12.678013	7.643674	21.209819
H	13.202243	10.993189	18.640386
H	13.365518	12.379444	20.736252
H	15.672103	10.668742	4.857884
H	12.847568	17.510327	11.016860
H	12.806546	12.884141	7.767368
H	13.697840	14.015133	8.806873
H	14.599387	25.318160	14.202743

H	14.574856	25.279277	16.983631
H	14.659118	22.350316	9.206592
H	14.861365	16.226577	20.784114
H	14.636088	22.024815	21.871175
H	14.653722	13.698331	10.850428
H	14.838849	7.720365	9.795692
H	14.796757	7.938011	22.456385
H	14.908847	4.720820	17.574912
H	14.934161	4.663557	14.794527
H	14.913076	10.692770	12.926471
H	16.181578	11.721862	19.886327
H	16.127011	10.036421	17.501276
H	17.797801	12.027674	4.902418
H	12.753176	16.979533	6.441909
H	14.550001	15.298277	6.738564
H	16.325711	18.962649	12.897327
H	16.799036	22.587785	10.430473
H	16.999703	25.258956	13.133270
H	16.772840	22.370976	20.669465
H	12.574825	18.890906	8.053928
H	16.890983	25.220077	18.065254
H	17.050267	22.957153	14.171229
H	17.029056	22.952142	16.960564
H	17.250960	16.681408	20.681282
H	17.008183	7.549489	10.972978
H	16.972054	7.711604	21.297222
H	16.756838	19.085484	10.199492
H	17.291949	4.849267	13.852143
H	17.268606	4.915499	18.542596
H	18.794588	12.346483	8.705987
H	17.364287	7.228384	14.693002
H	17.345342	7.253202	17.592753
H	19.053406	13.223884	12.872327
H	18.669913	13.212918	10.254504
H	18.077987	13.790698	6.557226
H	17.660486	15.748187	11.554235
H	14.497590	19.397444	9.542689
H	17.894129	14.482516	19.799745
H	17.943900	24.651422	11.754356
H	17.843084	24.603547	19.434241
H	17.939849	10.998116	14.996433
H	17.969651	20.996807	12.901623
H	17.372368	18.765020	8.566686
H	18.011053	20.989082	18.189955
H	18.172308	5.344884	12.387995
H	18.141848	5.478924	19.986153
H	18.241739	9.072578	13.189388
H	18.213578	9.165206	19.008887
H	18.456190	11.718059	18.118720

H	18.665324	24.680750	13.382850
H	18.581600	18.820850	15.268725
H	20.266928	12.698602	9.648128
H	18.606563	22.103536	11.655700
H	18.576813	24.706753	17.813800
H	18.606318	22.091952	19.459311
H	17.707637	17.610339	9.875420
H	18.891331	7.828391	12.088500
H	18.853751	7.978340	20.176109
H	18.963161	5.445986	13.981853
H	18.940517	5.506794	18.393369
H	19.211144	22.199923	13.320890
H	19.205226	22.248644	17.796136
H	17.697119	16.966639	7.139480
H	19.485530	7.934378	13.756442
H	19.463209	7.999882	18.511790
H	20.795690	16.483065	11.799984
H	20.149288	14.051212	20.401883
H	20.288108	19.932392	16.488317
H	20.626609	10.672307	15.121349
H	21.564763	14.131909	8.383256
H	22.776071	11.819131	10.733040
H	21.067901	18.930803	21.230906
H	21.943081	17.839478	11.650283
H	22.101752	16.426547	10.589763
H	22.828914	14.298676	10.634865
H	21.427229	12.854037	20.705274
H	21.340184	14.315597	21.703284
H	19.956636	17.582930	6.350633
H	21.373062	16.491152	21.507257
H	21.553093	20.468434	15.361388
H	21.430712	19.992038	19.023976
H	22.058957	9.661650	14.775515
H	22.603492	10.662669	12.911295
H	21.643358	20.943468	17.066282
H	21.616006	10.031989	16.452319
H	22.494563	16.531122	13.643213
H	22.044926	14.097410	18.711110
H	21.987027	18.141656	15.842186
H	22.140645	12.392950	16.200146
H	21.928864	16.166789	7.004888
H	24.360032	17.593060	12.386209
H	24.588760	16.116431	11.417374
H	23.852351	13.403945	20.290427
H	23.758027	14.975339	21.123771
H	23.943408	19.697605	15.649856
H	23.960014	19.919033	17.417512
H	24.434725	10.655847	15.083367
H	23.993655	10.825633	16.800810

H	24.935921	16.097966	13.161738
H	24.341194	14.889958	19.446654
H	24.357377	18.334961	16.711505
H	24.744690	14.545499	15.243780
H	24.443787	16.116470	17.509904
H	24.646034	12.217997	15.908342
H	4.911346	17.892068	16.406320
H	4.970260	15.563259	17.020680
H	5.016844	15.117510	12.811532
H	4.989875	13.868879	14.825302
H	5.136140	14.252078	19.043661
H	5.068534	11.510303	15.479321
H	5.380758	14.880893	11.086515
H	5.471539	16.509047	11.802938
H	5.355152	19.144417	15.223735
H	5.350284	19.521580	16.965296
H	5.529764	14.477999	20.763874
H	5.694439	12.870655	20.013862
H	5.582920	9.899390	14.930219
H	5.598968	10.297133	16.667307
H	7.267764	17.572739	15.536032
H	7.420758	15.809833	13.208357
H	7.561241	13.705952	18.572720
H	7.417641	11.966671	16.300261
H	7.725860	15.388622	10.172462
H	7.734291	13.241197	10.523132
H	7.612983	19.632566	18.648498
H	7.874006	16.896423	11.092075
H	7.798763	16.253929	21.290737
H	7.761283	19.877131	14.922825
H	7.758233	20.418525	16.611259
H	7.940014	14.101538	21.603808
H	7.805823	9.889214	13.203996
H	8.135296	12.614540	20.660044
H	7.919706	18.697479	20.920270
H	8.004415	10.813308	10.914886
H	8.049858	9.702727	16.931807
H	8.048589	9.140157	15.250591
H	9.095148	15.624394	11.293810
H	9.036584	19.314600	16.031394
H	9.280004	13.952877	20.434177
H	9.271225	10.323295	15.793184

4Ad'
E = -2285.5100 eV

Rh	10.042383	14.663308	16.171636
Rh	10.287989	16.849695	14.651884
Rh	10.567668	12.465769	17.637240
Rh	11.038039	17.082914	17.140543

Rh	11.199471	14.853610	18.681922
Rh	11.112228	14.586235	13.556901
Rh	11.248243	12.347714	15.086993
Rh	12.014623	17.282047	19.611023
Rh	12.141621	12.276803	12.597252
Rh	12.323343	18.582233	15.160989
Rh	12.290525	17.059394	12.857917
Rh	12.561051	15.905197	15.330423
Rh	12.661997	12.523956	19.381219
Rh	12.650226	13.673752	16.820937
Rh	12.808613	11.025332	17.023706
Rh	13.190683	18.745393	17.774362
Rh	13.409099	15.089321	20.403973
Rh	13.281149	14.568428	11.798261
Rh	13.416828	16.057526	17.886042
Rh	13.504338	13.601926	14.269628
Rh	13.522527	10.931049	14.408768
Rh	14.552031	20.123913	15.871274
Rh	14.577873	18.619198	13.513732
Rh	14.691995	17.472461	19.708570
Rh	14.637527	17.527890	15.946907
Rh	14.657357	16.960223	11.226762
Rh	14.727417	15.975587	13.646577
Rh	14.797925	14.885921	16.077392
Rh	14.882021	13.822082	18.397638
Rh	14.979886	12.898081	20.741861
Rh	14.941661	12.271839	16.156802
Rh	14.877521	12.319885	12.373249
Rh	15.037750	9.679066	16.289719
Rh	15.081177	11.158651	18.650911
Rh	15.981355	18.873547	17.749705
Rh	16.091446	16.213726	17.847084
Rh	16.192061	13.773905	14.230745
Rh	16.271472	15.234996	20.340036
Rh	16.155572	14.739600	11.774307
Rh	16.375749	11.045146	14.334360
Rh	16.816925	18.840950	15.082529
Rh	16.887620	17.210589	12.832913
Rh	16.905327	16.175404	15.294994
Rh	17.085885	13.924767	16.735952
Rh	17.223864	12.811486	19.234533
Rh	17.283689	11.226811	16.965177
Rh	17.393804	17.571206	19.539140
Rh	17.557844	12.560510	12.481172
Rh	18.308136	17.548032	17.041014
Rh	18.466598	15.253581	18.556320
Rh	18.456545	14.960081	13.421416
Rh	18.594762	12.711684	14.964494
Rh	19.013298	17.323243	14.492822

Rh	19.315623	12.982286	17.492351
Rh	19.570823	15.181175	16.023684
N	14.718407	23.056828	14.782765
N	14.669181	23.043553	16.960777
N	15.041284	6.732911	15.215034
N	15.006692	6.751354	17.393774
N	22.513569	14.620155	15.250613
N	22.411133	15.819767	17.077280
N	7.152609	15.292293	17.061025
N	7.158866	13.921757	15.355438
P	16.778979	15.366419	6.474239
C	11.051775	21.858900	12.926081
C	10.958605	21.816447	18.746567
C	11.248657	7.869995	19.053496
C	11.364766	7.888391	13.484441
C	11.649324	24.314943	13.148581
C	11.593223	24.265285	18.577450
C	11.943777	5.414905	13.498648
C	11.903195	5.418825	18.989911
C	12.142892	22.860062	13.366082
C	12.069811	22.807922	18.337442
C	12.452167	6.845143	13.819880
C	12.376433	6.870503	18.714277
C	13.495207	22.641261	12.674056
C	13.544266	22.416574	11.285420
C	13.411576	22.557039	19.038314
C	13.443433	22.259088	20.413537
C	13.790685	7.111019	13.119058
C	13.693127	7.156593	19.448805
C	13.897778	12.756109	5.045121
C	13.816739	7.350059	11.732073
C	13.679511	7.424243	20.830556
C	14.892155	12.989187	7.351635
C	13.735658	13.131001	3.704640
C	14.773763	13.440611	5.911271
C	14.728078	24.387846	15.201459
C	14.734109	22.734170	13.362930
C	14.699444	24.379359	16.560736
C	14.762601	22.310051	10.609056
C	14.680799	22.178773	15.865835
C	14.659394	22.689346	18.372589
C	14.652533	22.117972	21.100004
C	15.023165	7.501939	11.044565
C	15.008916	7.615924	16.296164
C	15.037719	7.050328	13.794764
C	14.959429	7.091380	18.809888
C	14.865433	7.599390	21.547811
C	15.055024	5.403335	15.635943
C	15.032054	5.415643	16.995223

C	14.457031	14.221260	3.202091
C	15.966456	22.438722	11.307137
C	15.986832	22.661846	12.696886
C	15.505853	14.553417	5.397666
C	15.901406	22.603784	19.055453
C	15.864198	22.302062	20.430100
C	16.237456	7.394357	11.727699
C	16.099114	7.486774	20.901590
C	16.279581	7.156808	13.114576
C	16.181696	7.219598	19.522238
C	15.329778	14.921860	4.045474
C	13.707287	17.529150	8.294079
C	14.414298	16.687142	7.447932
C	14.384392	18.260059	9.315893
C	15.829115	16.521098	7.561729
C	17.324663	22.893479	13.412751
C	17.243377	22.913426	18.379702
C	15.793172	18.072957	9.463706
C	16.521976	17.207581	8.582423
C	17.629187	6.947242	13.813837
C	17.552079	7.004736	18.866078
C	17.825368	24.343770	13.182247
C	17.718458	24.343721	18.749127
C	18.024953	17.138515	8.735240
C	18.144606	5.500996	13.589548
C	18.055923	5.558358	19.115086
C	19.362386	14.627057	4.830761
C	18.424969	21.885816	13.011011
C	18.349491	21.885985	18.699730
C	18.709417	7.964449	13.385818
C	18.623439	8.021936	19.318614
C	17.664452	16.505349	5.302137
C	18.844645	16.040894	4.652637
C	17.236614	17.831984	5.079866
C	19.540499	16.927695	3.806533
C	17.942317	18.696536	4.232119
C	19.102231	18.241054	3.591363
C	21.521649	16.653001	11.563906
C	21.378721	13.788293	20.756614
C	21.499290	20.034771	16.549282
C	21.581146	15.212841	16.118081
C	21.714960	10.427453	15.651115
C	22.144513	12.318075	11.702149
C	21.542440	18.170355	20.507600
C	22.162304	13.713037	11.630326
C	21.691229	16.787168	20.630580
C	22.256425	11.680784	12.939578
C	21.715682	18.784817	19.264575
C	22.261711	14.505796	12.789160

C	21.973223	15.976999	19.513037
C	21.998970	18.033886	18.107775
C	22.357243	12.414771	14.137145
C	22.310619	13.832355	14.040244
C	22.062531	16.619180	18.246377
C	22.415335	16.027952	12.657755
C	22.287508	14.487425	19.721270
C	22.332810	18.758210	16.794875
C	22.612380	11.670011	15.455787
C	23.897767	16.408856	12.399316
C	23.823247	14.855926	15.667549
C	23.759564	15.596916	16.800624
C	23.771851	14.298050	20.137614
C	23.841098	19.124981	16.739099
C	24.103675	11.255363	15.572290
C	5.661805	15.213020	12.284482
C	5.604421	18.598441	16.497946
C	5.854095	13.889885	20.169563
C	5.823239	14.996789	16.760001
C	5.827040	14.146247	15.703598
C	5.839388	10.527245	16.022102
C	7.168565	15.024220	12.608373
C	7.105967	18.208406	16.559512
C	7.334685	14.162238	19.789347
C	7.305425	11.021195	15.893486
C	7.443079	16.193807	18.169715
C	7.455646	13.024393	14.245163
C	7.446752	17.595547	17.925991
C	7.475121	13.553094	12.925042
C	7.549056	15.651498	19.480555
C	7.547796	11.629269	14.504743
C	7.670771	18.443526	19.028041
C	7.699020	12.647940	11.869769
C	7.769293	16.554935	20.538065
C	7.768240	10.778216	13.404619
C	7.848193	17.931845	20.315595
C	7.862095	11.280702	12.104662
C	8.015727	15.604432	11.454424
C	8.035538	14.629536	16.193414
C	7.971311	19.433022	16.187292
C	8.255217	13.614031	20.901971
C	8.272079	9.870638	16.251053
H	10.151247	21.999239	13.545335
H	10.070196	21.977124	18.115239
H	10.374115	7.672626	18.412841
H	10.459508	7.687526	14.079582
H	10.701889	24.480768	13.686842
H	10.749771	22.007268	11.877155
H	10.651304	24.452626	18.036544

H	10.644575	21.952967	19.793701
H	10.638655	17.629342	13.074274
H	11.012300	5.207003	14.050102
H	11.074652	7.862449	12.422265
H	10.913522	7.779334	20.098832
H	10.986320	5.200340	18.418226
H	11.045181	11.825237	19.222612
H	11.384853	20.818820	13.044598
H	11.470060	24.512444	12.079394
H	11.277771	20.770733	18.615237
H	11.410388	24.446260	19.649004
H	11.564932	8.909371	18.887524
H	11.698715	8.912251	13.721568
H	11.732220	5.302605	12.423062
H	11.677436	5.276094	20.059029
H	11.687212	15.508792	12.014383
H	11.900892	13.993644	20.216321
H	12.291702	22.713997	14.446172
H	12.228266	22.680652	17.256191
H	12.376245	25.057634	13.509508
H	12.367112	16.293077	21.097792
H	12.331531	25.004579	18.233568
H	12.298200	13.291114	11.103590
H	12.612660	22.331185	10.722957
H	12.621105	6.901883	14.904999
H	12.566866	6.960332	17.634515
H	12.505700	22.135233	20.958008
H	12.679817	4.645348	13.776546
H	13.335147	11.902980	5.436150
H	12.662880	4.675040	18.706403
H	12.739180	19.909115	16.402685
H	15.942447	12.959006	7.683619
H	12.724824	18.788485	13.321685
H	12.876769	7.414055	11.180526
H	12.724272	7.498135	21.353757
H	13.215573	10.849262	18.854168
H	13.341669	12.220009	20.969195
H	13.054607	12.571244	3.058208
H	12.981308	17.428016	11.305254
H	14.454118	11.989788	7.488649
H	14.367238	13.674795	8.036842
H	14.753504	25.209682	14.496254
H	14.700934	25.192328	17.276609
H	14.774265	22.134906	9.530041
H	14.798737	16.066708	21.098309
H	14.649664	21.873328	22.165318
H	14.773557	13.707151	11.064677
H	15.017909	7.696742	9.969114
H	14.828077	7.818957	22.617918

H	15.027445	4.604203	17.712752
H	15.076241	4.579804	14.932419
H	14.996559	10.646485	13.152729
H	16.184465	11.582455	20.139838
H	16.162242	9.932164	17.719297
H	14.343929	14.530101	2.159584
H	12.630674	17.660497	8.162218
H	13.879226	16.157609	6.657188
H	16.376499	18.968267	13.264637
H	16.909194	22.370737	10.760805
H	17.092434	25.093878	13.514362
H	16.799619	22.213998	20.986138
H	13.891856	19.111592	9.788972
H	16.971484	25.107960	18.487044
H	17.154095	22.764611	14.492000
H	17.089237	22.884357	17.291029
H	17.175295	16.559738	21.036359
H	17.171553	7.495701	11.172191
H	17.016349	7.608798	21.480314
H	18.328593	17.411415	9.755714
H	17.426839	4.745632	13.942266
H	17.346071	4.801916	18.748727
H	18.700557	13.891036	4.344330
H	17.472509	7.083634	14.894258
H	17.427433	7.133948	17.780401
H	19.122587	13.239853	13.162194
H	19.413528	14.348059	5.895048
H	15.885369	15.770428	3.643206
H	17.692355	15.806044	11.908306
H	16.363862	18.768928	10.084562
H	17.875393	14.367066	20.123798
H	18.028754	24.523641	12.114282
H	17.915046	24.425881	19.830161
H	17.990011	10.938354	15.260355
H	18.094330	20.847067	13.153813
H	18.525376	17.827475	8.033006
H	18.037784	20.861344	18.442214
H	18.331622	5.312633	12.519935
H	18.212587	5.378327	20.190968
H	18.358325	9.000719	13.501667
H	18.280243	9.057176	19.189107
H	18.462058	11.619847	18.403106
H	18.761153	24.516635	13.738304
H	18.598119	18.768851	15.672581
H	20.364340	14.519168	4.390025
H	18.737717	22.008794	11.961855
H	18.652208	24.586058	18.215886
H	18.635066	21.899737	19.763509
H	18.405074	16.127145	8.519050

H	19.021931	7.822315	12.339052
H	18.908413	7.881238	20.373418
H	19.091687	5.345344	14.131472
H	19.017439	5.395631	18.601190
H	19.317365	22.044475	13.637511
H	19.252689	22.119677	18.114141
H	16.333532	18.192550	5.577433
H	19.605380	7.839850	14.014592
H	19.536176	7.888537	18.715780
H	20.465925	16.373303	11.697846
H	20.313283	13.928055	20.525121
H	20.422676	19.845260	16.650773
H	20.648727	10.680947	15.555044
H	20.449106	16.576346	3.308224
H	22.059395	11.725258	10.787626
H	21.313153	18.774926	21.389047
H	21.589711	17.751524	11.613743
H	21.833594	16.356033	10.550043
H	22.104440	14.195792	10.653643
H	21.583727	12.706035	20.755987
H	21.561081	14.151181	21.780511
H	17.583805	19.716870	4.071852
H	21.592640	16.326534	21.614630
H	21.682402	20.401794	15.526668
H	21.637903	19.870702	19.193053
H	21.948456	9.630287	14.927579
H	22.273753	10.590009	12.977302
H	21.779684	20.847222	17.238633
H	21.872832	10.010255	16.658684
H	22.111778	16.477412	13.616290
H	22.137621	13.974998	18.757485
H	22.118571	18.065360	15.966404
H	22.388783	12.362112	16.282451
H	19.665890	18.903309	2.928851
H	24.004626	17.504453	12.343833
H	24.247545	15.986220	11.443641
H	23.999460	13.225317	20.247537
H	23.968399	14.785967	21.105930
H	24.075549	19.616918	15.781026
H	24.099427	19.824429	17.550653
H	24.374201	10.543986	14.775306
H	24.286727	10.762617	16.541042
H	24.566477	16.046325	13.192959
H	24.473856	14.716321	19.402776
H	24.493723	18.246427	16.831463
H	24.673277	14.475364	15.115622
H	24.543746	15.988453	17.435141
H	24.785334	12.114847	15.498367
H	4.941243	17.744724	16.697588

H	5.002942	15.419156	17.325962
H	5.009861	14.885881	13.106517
H	5.011018	13.678172	15.168068
H	5.153062	14.197675	19.381033
H	5.111096	11.329001	15.833523
H	5.383370	14.640702	11.384838
H	5.447493	16.276125	12.088240
H	5.354817	18.992704	15.499494
H	5.374982	19.381858	17.238256
H	5.583791	14.430538	21.091032
H	5.702182	12.813662	20.352886
H	5.636866	9.714933	15.305444
H	5.656220	10.137431	17.036595
H	7.286399	17.439264	15.791981
H	7.398983	15.615285	13.508859
H	7.560217	13.594856	18.872893
H	7.459559	11.815520	16.640095
H	7.742752	15.168101	10.480571
H	7.741976	13.020106	10.845262
H	7.697222	19.523689	18.876556
H	7.847649	16.690338	11.382698
H	7.876403	16.173867	21.554807
H	7.750963	19.741804	15.153000
H	7.764692	20.299279	16.835839
H	7.998553	14.024398	21.891478
H	7.859366	9.702965	13.568061
H	8.145786	12.520127	20.966808
H	8.028415	18.610416	21.153480
H	8.043132	10.601117	11.267783
H	8.113862	9.564571	17.297365
H	8.107791	8.979940	15.623771
H	9.091405	15.445209	11.616735
H	9.046805	19.203254	16.249846
H	9.314378	13.830095	20.701835
H	9.324672	10.178492	16.141902

4TSd'$_{\text{methyl}}$

E = -2284.5045 eV

Rh	9.867055	14.641266	15.886025
Rh	10.168013	16.777745	14.303602
Rh	10.397563	12.479800	17.400273
Rh	10.888621	17.066955	16.805699
Rh	11.008817	14.882025	18.399754
Rh	10.991318	14.478794	13.298147
Rh	11.109746	12.286954	14.882685
Rh	11.832494	17.333039	19.287446
Rh	12.036809	12.153210	12.401893
Rh	12.213645	18.515683	14.794196
Rh	12.212794	16.930665	12.531813

Rh	12.413408	15.846521	15.051688
Rh	12.461521	12.569881	19.173249
Rh	12.508653	13.642915	16.613877
Rh	12.639032	11.007977	16.859254
Rh	12.979052	18.750396	17.417481
Rh	13.206709	15.161276	20.139349
Rh	13.177991	14.438669	11.580008
Rh	13.249653	16.058264	17.612201
Rh	13.383052	13.519390	14.065376
Rh	13.391914	10.849699	14.253786
Rh	14.407121	20.073164	15.515153
Rh	14.487026	18.500194	13.198784
Rh	14.494552	17.552545	19.438619
Rh	14.494619	17.488940	15.676768
Rh	14.605363	16.759040	10.949488
Rh	14.617006	15.862376	13.409201
Rh	14.655827	14.859675	15.845468
Rh	14.707823	13.818431	18.199488
Rh	14.765554	12.949505	20.572398
Rh	14.788929	12.227457	15.979630
Rh	14.775800	12.218367	12.226946
Rh	14.872282	9.648087	16.181477
Rh	14.889545	11.173387	18.508567
Rh	15.869791	18.903130	17.416652
Rh	15.922424	16.197017	17.631586
Rh	16.088951	13.689058	14.073613
Rh	16.076621	15.259781	20.103621
Rh	16.090592	14.589020	11.601983
Rh	16.253053	10.948451	14.227096
Rh	16.704861	18.735758	14.791154
Rh	16.846047	17.055099	12.454297
Rh	16.801694	16.091861	15.069759
Rh	16.902594	13.872988	16.608162
Rh	17.049113	12.810405	19.108089
Rh	17.130293	11.183186	16.871909
Rh	17.213788	17.579442	19.300436
Rh	17.467286	12.443979	12.365609
Rh	18.160267	17.490173	16.786049
Rh	18.309261	15.247884	18.385049
Rh	18.364118	14.846763	13.258011
Rh	18.456702	12.652652	14.854080
Rh	18.916920	17.211181	14.260579
Rh	19.133906	12.950239	17.359945
Rh	19.461842	15.144982	15.896350
N	14.514120	23.019487	14.424925
N	14.471867	23.012765	16.601636
N	14.827729	6.686558	15.126203
N	14.810354	6.716971	17.304231
N	22.437485	14.573940	15.314967

N	22.232354	15.795846	17.117864
N	6.988938	15.288203	16.785932
N	6.982235	13.926305	15.072725
P	17.737595	15.533369	8.047796
C	10.908665	21.730373	12.528274
C	10.792986	21.740376	18.413625
C	11.082149	7.898363	18.987003
C	11.143063	7.852896	13.396181
C	11.412923	24.208785	12.748686
C	11.369288	24.200729	18.228991
C	11.716545	5.380420	13.450518
C	11.707147	5.438740	18.952299
C	11.956586	22.773393	12.976181
C	11.879136	22.753820	17.992845
C	12.230732	6.814179	13.745032
C	12.194741	6.880813	18.651332
C	13.322366	22.600661	12.297994
C	13.392481	22.410164	10.904880
C	13.228435	22.539615	18.691366
C	13.268168	22.277084	20.073599
C	13.567522	7.064767	13.035116
C	13.522659	7.159685	19.368397
C	16.488977	12.583955	5.399808
C	13.588365	7.297054	11.646880
C	13.526977	7.445244	20.746532
C	16.068733	12.829693	7.871859
C	16.991870	13.009672	4.163687
C	16.657306	13.342128	6.576626
C	14.491070	24.352238	14.837769
C	14.550545	22.707276	13.003668
C	14.466512	24.347255	16.196576
C	14.620346	22.351208	10.240105
C	14.503874	22.141382	15.510071
C	14.472148	22.680314	18.019462
C	14.480352	22.185629	20.762674
C	14.792230	7.433836	10.951671
C	14.819587	7.575266	16.202726
C	14.818230	6.996395	13.703816
C	14.781861	7.069072	18.718010
C	14.722609	7.612094	21.449440
C	14.822806	5.359354	15.553834
C	14.809938	5.378970	16.913510
C	17.677531	14.228227	4.081667
C	15.812791	22.490062	10.955772
C	15.811331	22.679445	12.350669
C	17.363860	14.578382	6.493432
C	15.715649	22.643312	18.703550
C	15.686198	22.382151	20.086478
C	16.009198	7.318992	11.628541

C	15.948332	7.472859	20.792828
C	16.057082	7.088890	13.016228
C	16.013214	7.188201	19.416146
C	17.857476	14.998989	5.237990
C	14.217249	17.684008	7.392362
C	15.320528	16.802120	7.263617
C	14.117707	18.503836	8.505577
C	16.320581	16.710641	8.236037
C	17.135419	22.927067	13.085327
C	17.051283	22.961876	18.019013
C	15.100138	18.442242	9.539131
C	16.226051	17.534617	9.440556
C	17.408835	6.871761	13.708704
C	17.373206	6.948374	18.746611
C	17.650765	24.364828	12.814307
C	17.499838	24.409599	18.351094
C	17.396381	17.734889	10.368755
C	17.910143	5.419792	13.488338
C	17.858641	5.497052	19.002998
C	20.734701	14.614070	7.786570
C	18.229992	21.892391	12.742115
C	18.176432	21.963652	18.365290
C	18.495080	7.878397	13.270567
C	18.463532	7.954192	19.179099
C	19.145607	16.617320	7.494713
C	20.463443	16.071693	7.474885
C	18.963835	17.976680	7.159646
C	21.536238	16.915784	7.127918
C	20.047323	18.799572	6.821879
C	21.342412	18.266858	6.808307
C	21.680525	16.738470	11.715598
C	21.100586	13.782009	20.760970
C	21.276117	19.998870	16.502201
C	21.456556	15.176587	16.120474
C	21.554482	10.348426	15.503024
C	22.298060	12.375770	11.687647
C	21.164718	18.174300	20.472011
C	22.350551	13.771119	11.659510
C	21.342908	16.797549	20.622639
C	22.294972	11.700917	12.910660
C	21.379992	18.775597	19.228831
C	22.372290	14.527101	12.846306
C	21.694678	15.979271	19.530975
C	21.731235	18.015964	18.096455
C	22.314188	12.397659	14.134124
C	22.306702	13.818065	14.075773
C	21.819847	16.604976	18.258695
C	22.571652	16.047379	12.770221
C	22.047147	14.504105	19.777227

C	22.105043	18.727886	16.787100
C	22.437176	11.614874	15.449654
C	24.062264	16.389237	12.506594
C	23.722092	14.818490	15.799682
C	23.594462	15.573373	16.917867
C	23.507718	14.377317	20.290183
C	23.613095	19.099640	16.773723
C	23.914061	11.226195	15.726870
C	5.522293	15.239901	12.001234
C	5.472446	18.612315	16.202454
C	5.691428	13.895603	19.894194
C	5.656618	15.006496	16.483213
C	5.652339	14.160972	15.422744
C	5.634961	10.529500	15.748254
C	7.026488	15.021601	12.319456
C	6.967589	18.200558	16.272184
C	7.173707	14.165677	19.519276
C	7.098791	11.030566	15.623363
C	7.285904	16.190901	17.891945
C	7.272638	13.023381	13.965352
C	7.296514	17.591612	17.643209
C	7.305503	13.546467	12.643087
C	7.390423	15.653255	19.204859
C	7.346233	11.628704	14.231216
C	7.521721	18.443078	18.742279
C	7.518145	12.633373	11.592364
C	7.613072	16.559901	20.259387
C	7.556032	10.769649	13.135445
C	7.696106	17.935795	20.032020
C	7.658529	11.264546	11.833358
C	7.880000	15.579247	11.158889
C	7.865539	14.620174	15.915107
C	7.852654	19.409466	15.895144
C	8.088639	13.622257	20.639070
C	8.071917	9.890021	15.995426
H	9.992757	21.845719	13.129854
H	9.895793	21.880342	17.789906
H	10.197581	7.699227	18.360792
H	10.243134	7.666774	14.003912
H	10.458202	24.343516	13.282667
H	10.621403	21.856653	11.472389
H	10.422625	24.365463	17.688908
H	10.486170	21.871148	19.463509
H	10.564839	17.533007	12.733061
H	10.785972	5.186181	14.008253
H	10.842434	7.803206	12.337697
H	10.759329	7.829765	20.037780
H	10.782437	5.222917	18.392510
H	10.839959	11.869012	19.004837

H	11.275715	20.703953	12.664834
H	11.230444	24.395495	11.678088
H	11.132335	20.700252	18.281422
H	11.184106	24.380735	19.300271
H	11.407050	8.931099	18.798411
H	11.480974	8.881137	13.606983
H	11.501201	5.251104	12.377651
H	11.489841	5.313925	20.025287
H	11.585466	15.370401	11.734820
H	11.703245	14.060520	19.964663
H	12.099808	22.636955	14.058305
H	12.035431	22.624555	16.911535
H	12.111770	24.978517	13.108885
H	12.139053	16.359872	20.801796
H	12.090427	24.955686	17.882433
H	12.182638	13.166644	10.901490
H	12.469224	22.318067	10.329628
H	12.404474	6.889920	14.828265
H	12.375250	6.952776	17.568488
H	12.333732	22.146515	20.622173
H	12.449849	4.612353	13.739099
H	15.952332	11.632502	5.463271
H	12.455395	4.682029	18.672851
H	12.601543	19.889397	16.017965
H	16.786630	12.898819	8.703837
H	12.633969	18.658799	12.948056
H	12.645950	7.367656	11.100301
H	12.578463	7.539207	21.278390
H	13.025043	10.881982	18.701377
H	13.124542	12.309924	20.790608
H	16.849805	12.391752	3.273199
H	12.899113	17.209787	10.942949
H	15.751084	11.781435	7.774214
H	15.186642	13.416859	8.174907
H	14.495511	25.171498	14.128899
H	14.448809	25.162116	16.910013
H	14.648070	22.206059	9.157004
H	14.587524	16.167046	20.804316
H	14.483877	21.969671	21.834025
H	14.688522	13.550206	10.900960
H	14.782384	7.622936	9.875239
H	14.699480	7.845900	22.516807
H	14.798938	4.571596	17.635574
H	14.826768	4.531878	14.854685
H	14.888658	10.556749	13.022456
H	15.968824	11.617836	20.009998
H	15.984711	9.916220	17.622964
H	18.074151	14.580077	3.125717
H	13.462985	17.723061	6.603011

H	15.398254	16.191914	6.362090
H	16.232339	18.907364	13.037629
H	16.763432	22.461764	10.419220
H	16.910663	25.128321	13.097861
H	16.623709	22.332147	20.643297
H	13.292956	19.213157	8.608061
H	16.738581	25.152982	18.070743
H	16.943522	22.838999	14.164602
H	16.897519	22.901456	16.931552
H	17.051063	16.537088	20.785014
H	16.940891	7.409292	11.067230
H	16.873042	7.589028	21.360758
H	17.485575	18.769841	10.723346
H	17.187674	4.673185	13.850024
H	17.136152	4.748232	18.646030
H	20.384297	13.959691	6.970943
H	17.259329	7.014412	14.789367
H	17.238769	7.070790	17.661371
H	19.035149	13.148089	13.030097
H	20.209426	14.291258	8.699723
H	18.396884	15.944536	5.162411
H	17.706383	16.492068	11.207714
H	15.149948	19.263494	10.260167
H	17.670826	14.375888	19.967193
H	17.885332	24.506005	11.746930
H	17.696216	24.522956	19.429341
H	17.860797	10.883385	15.178814
H	17.892013	20.866591	12.948633
H	18.352713	17.459150	9.903279
H	17.884332	20.928169	18.132059
H	18.088194	5.224139	12.418554
H	18.018697	5.322444	20.079227
H	18.152382	8.918301	13.383227
H	18.132281	8.993084	19.045887
H	18.298708	11.585700	18.286041
H	18.571017	24.555020	13.390175
H	18.476827	18.688595	15.388040
H	21.812210	14.434822	7.916852
H	18.540227	21.950626	11.686640
H	18.428321	24.655574	17.810457
H	18.457725	22.007400	19.429440
H	18.801376	7.729586	12.222930
H	18.759164	7.817434	20.231440
H	18.859340	5.258287	14.024809
H	18.814904	5.317097	18.485170
H	19.124986	22.081050	13.356536
H	19.076420	22.203022	17.777380
H	17.955819	18.396607	7.159102
H	19.393187	7.750936	13.895659

H	19.367236	7.804680	18.566549
H	20.616827	16.499552	11.875147
H	20.049192	13.869932	20.453464
H	20.197199	19.807373	16.571302
H	20.502334	10.579331	15.281784
H	22.548566	16.500199	7.111522
H	22.276057	11.810696	10.752069
H	20.880198	18.785338	21.332495
H	21.798890	17.831622	11.786874
H	21.946836	16.451424	10.686910
H	22.387229	14.284312	10.697485
H	21.352855	12.710262	20.795867
H	21.194585	14.170426	21.787390
H	19.875563	19.848785	6.567251
H	21.212578	16.348355	21.608351
H	21.489561	20.355909	15.481967
H	21.279386	19.858022	19.137734
H	21.898151	9.574754	14.797996
H	22.284482	10.609732	12.915535
H	21.532108	20.819250	17.191584
H	21.595852	9.910413	16.513003
H	22.298291	16.471202	13.747931
H	21.979523	13.976187	18.812908
H	21.917655	18.025518	15.960175
H	22.102314	12.276860	16.264402
H	22.198360	18.895645	6.549696
H	24.208133	17.481803	12.504946
H	24.385778	16.004047	11.526053
H	23.774177	13.315395	20.416049
H	23.619327	14.872047	21.268478
H	23.874084	19.586149	15.819575
H	23.844387	19.805957	17.587540
H	24.294217	10.554194	14.940335
H	23.992242	10.696862	16.690492
H	24.727618	15.961619	13.270972
H	24.237947	14.827484	19.603101
H	24.265919	18.224254	16.891161
H	24.601922	14.438227	15.296538
H	24.341725	15.975922	17.588999
H	24.577999	12.101138	15.770597
H	4.796309	17.769882	16.406674
H	4.840596	15.434929	17.050862
H	4.867496	14.926987	12.826547
H	4.832013	13.701719	14.886038
H	4.994062	14.197188	19.100070
H	4.903832	11.325635	15.546760
H	5.228758	14.672031	11.103563
H	5.328023	16.306695	11.804148
H	5.231925	19.001757	15.199841

H	5.251733	19.405110	16.935368
H	5.416652	14.442961	20.810361
H	5.539100	12.820761	20.084911
H	5.440977	9.708763	15.038982
H	5.447996	10.149239	16.765675
H	7.139738	17.424424	15.510233
H	7.272555	15.612190	13.216394
H	7.403152	13.594265	18.606167
H	7.243767	11.831585	16.364390
H	7.592406	15.144816	10.188325
H	7.570573	13.000406	10.566431
H	7.552784	19.522688	18.586247
H	7.733724	16.668080	11.082957
H	7.719312	16.181999	21.277239
H	7.640534	19.714253	14.857845
H	7.656679	20.283188	16.536842
H	7.823337	14.033309	21.625996
H	7.630736	9.693915	13.303862
H	7.982511	12.528095	20.704494
H	7.878343	18.616685	20.867628
H	7.830085	10.578842	10.999558
H	7.906036	9.585771	17.041178
H	7.923118	8.996036	15.369187
H	8.952786	15.398421	11.315946
H	8.924408	19.164370	15.961721
H	9.148235	13.841573	20.446032
H	9.122788	10.207733	15.896330
H	15.815954	18.882474	19.253884

4Id’$_{methyl}$

E = -2285.2963 eV

Rh	9.891059	14.643645	15.875470
Rh	10.171839	16.778996	14.286984
Rh	10.406384	12.491870	17.403809
Rh	10.908644	17.076802	16.779746
Rh	11.034684	14.901851	18.392506
Rh	10.994933	14.464497	13.284002
Rh	11.111303	12.278063	14.882512
Rh	11.854370	17.354351	19.255985
Rh	12.036692	12.132030	12.405563
Rh	12.228681	18.508235	14.754385
Rh	12.203604	16.901544	12.511258
Rh	12.429495	15.840986	15.021462
Rh	12.476026	12.601528	19.170083
Rh	12.505089	13.652477	16.590127
Rh	12.644729	11.023436	16.865604
Rh	13.019270	18.749962	17.376644
Rh	13.236630	15.194669	20.128584
Rh	13.185231	14.406874	11.561975

Rh	13.264904	16.071469	17.580856
Rh	13.388941	13.510412	14.051503
Rh	13.393536	10.842059	14.264734
Rh	14.426470	20.072458	15.476505
Rh	14.505309	18.482167	13.146016
Rh	14.527455	17.566486	19.405851
Rh	14.511731	17.480333	15.620243
Rh	14.556867	16.744145	10.915919
Rh	14.640997	15.854879	13.380520
Rh	14.665623	14.853028	15.826097
Rh	14.717987	13.838002	18.181177
Rh	14.781640	12.985407	20.557305
Rh	14.806047	12.229200	15.981305
Rh	14.777915	12.175673	12.220632
Rh	14.868858	9.646999	16.190437
Rh	14.896628	11.186927	18.510285
Rh	15.876129	18.905902	17.375927
Rh	15.943799	16.218554	17.581535
Rh	16.082222	13.671162	14.043446
Rh	16.099572	15.291070	20.079809
Rh	16.083763	14.550167	11.550097
Rh	16.247037	10.931367	14.223026
Rh	16.711196	18.715561	14.738180
Rh	16.808487	17.093370	12.374627
Rh	16.829074	16.069291	15.054616
Rh	16.919339	13.872784	16.582361
Rh	17.055697	12.830410	19.086108
Rh	17.129779	11.177398	16.868071
Rh	17.244628	17.606222	19.251614
Rh	17.466461	12.386747	12.357535
Rh	18.181126	17.505821	16.743611
Rh	18.313879	15.259869	18.355682
Rh	18.366711	14.826239	13.230057
Rh	18.457526	12.626065	14.838850
Rh	18.913707	17.207929	14.212234
Rh	19.148314	12.947340	17.344754
Rh	19.455959	15.144683	15.866403
N	14.524854	23.025629	14.429702
N	14.490221	23.000174	16.606614
N	14.824691	6.687663	15.126928
N	14.809586	6.720287	17.305560
N	22.438943	14.575649	15.307969
N	22.223849	15.807198	17.102478
N	7.011403	15.302647	16.784704
N	6.997063	13.947371	15.066947
P	17.719700	15.546304	8.128051
C	10.902723	21.749779	12.583031
C	10.805411	21.713959	18.389514
C	11.083061	7.916327	18.963164

C	11.133278	7.846761	13.401856
C	11.407654	24.232423	12.761092
C	11.379449	24.176794	18.233205
C	11.708582	5.374740	13.461840
C	11.690259	5.451580	18.958144
C	11.954256	22.800430	13.003762
C	11.892205	22.733183	17.984723
C	12.222434	6.809713	13.751585
C	12.189210	6.886618	18.644090
C	13.312811	22.618103	12.313414
C	13.369537	22.423095	10.920367
C	13.238243	22.514142	18.687926
C	13.270625	22.245670	20.069239
C	13.557706	7.058690	13.038019
C	13.515305	7.164406	19.364885
C	16.623810	12.513583	5.498829
C	13.574323	7.285021	11.648537
C	13.514533	7.450017	20.743159
C	16.126717	12.788900	7.950869
C	17.148735	12.930508	4.269371
C	16.739790	13.296619	6.665648
C	14.501566	24.354742	14.853107
C	14.548073	22.718240	13.007493
C	14.481746	24.338124	16.211981
C	14.591620	22.353373	10.245763
C	14.518897	22.138896	15.506800
C	14.485351	22.659424	18.022923
C	14.479052	22.153912	20.765006
C	14.775816	7.418651	10.948774
C	14.819285	7.577185	16.203033
C	14.810513	6.993994	13.703456
C	14.776617	7.074383	18.718781
C	14.707425	7.617384	21.450413
C	14.817276	5.360806	15.556446
C	14.805866	5.381815	16.916190
C	17.805349	14.164816	4.184586
C	15.791366	22.487495	10.950254
C	15.803363	22.681493	12.344404
C	17.416401	14.549256	6.581023
C	15.724918	22.623387	18.714509
C	15.688150	22.356518	20.096294
C	15.994773	7.308137	11.622443
C	15.935599	7.480136	20.797795
C	16.047086	7.085309	13.011261
C	16.005335	7.196733	19.421147
C	17.932954	14.960090	5.331311
C	14.146503	17.537241	7.298580
C	15.289782	16.698416	7.240325
C	13.978372	18.393232	8.374202

C	16.262869	16.684573	8.242482
C	17.134164	22.925305	13.068709
C	17.063422	22.948784	18.039147
C	14.930659	18.408721	9.436952
C	16.105411	17.556048	9.410956
C	17.401766	6.874869	13.700156
C	17.368035	6.965116	18.754422
C	17.641999	24.367427	12.807113
C	17.501443	24.399661	18.371878
C	17.199915	17.828366	10.396453
C	17.900775	5.420055	13.495340
C	17.858027	5.513172	18.999137
C	20.730807	14.669492	7.885643
C	18.229435	21.898312	12.705355
C	18.192044	21.958421	18.396507
C	18.487690	7.873630	13.243549
C	18.453838	7.970630	19.199089
C	19.115644	16.650943	7.589114
C	20.440210	16.122377	7.570344
C	18.916234	18.007413	7.254503
C	21.501853	16.978569	7.218894
C	19.988698	18.842498	6.911956
C	21.290223	18.325653	6.894690
C	21.684992	16.740830	11.723672
C	21.087264	13.808926	20.756495
C	21.264456	20.011795	16.472098
C	21.454285	15.181260	16.105005
C	21.549835	10.342913	15.488104
C	22.311105	12.374324	11.682723
C	21.143837	18.198184	20.443802
C	22.368659	13.769290	11.653919
C	21.324610	16.822617	20.600819
C	22.298654	11.700250	12.906408
C	21.359040	18.794502	19.198238
C	22.386572	14.525921	12.840366
C	21.679156	15.999712	19.513355
C	21.714623	18.030712	18.069969
C	22.314008	12.397421	14.129503
C	22.312514	13.817856	14.069498
C	21.805949	16.620597	18.238713
C	22.589965	16.045499	12.763757
C	22.032219	14.525685	19.767054
C	22.089898	18.738824	16.758971
C	22.424150	11.615696	15.446949
C	24.078107	16.382489	12.480279
C	23.721203	14.826602	15.795023
C	23.587286	15.587167	16.908851
C	23.493155	14.400494	20.279291
C	23.598818	19.106638	16.744133

C	23.899138	11.237177	15.747485
C	5.521626	15.277896	12.017213
C	5.506664	18.631902	16.213764
C	5.699617	13.901867	19.890490
C	5.677615	15.023894	16.485389
C	5.668813	14.182230	15.421949
C	5.641187	10.563154	15.724855
C	7.028780	15.059090	12.320456
C	7.001276	18.217970	16.285191
C	7.183017	14.167097	19.515133
C	7.110477	11.047280	15.597913
C	7.309988	16.199032	17.895652
C	7.283261	13.050983	13.953340
C	7.325121	17.600827	17.653590
C	7.312841	13.582894	12.634604
C	7.406821	15.654970	19.206691
C	7.358469	11.654684	14.209916
C	7.548791	18.446561	18.757376
C	7.527141	12.677097	11.577861
C	7.627802	16.556269	20.266108
C	7.569740	10.803229	13.108382
C	7.716314	17.932722	20.045448
C	7.671107	11.307194	11.809719
C	7.869225	15.623211	11.153430
C	7.884278	14.637188	15.908415
C	7.888979	19.427938	15.918534
C	8.096180	13.616124	20.632693
C	8.069896	9.890702	15.955424
H	9.991759	21.875235	13.190064
H	9.910697	21.859951	17.763750
H	10.198706	7.715237	18.337233
H	10.235166	7.662584	14.012827
H	10.458574	24.375000	13.302927
H	10.607474	21.856950	11.527242
H	10.435433	24.345925	17.689997
H	10.494158	21.832712	19.439570
H	10.556967	17.491375	12.683914
H	10.777573	5.182389	14.019526
H	10.829638	7.792739	12.344397
H	10.757443	7.865077	20.014025
H	10.766872	5.236067	18.395974
H	10.855174	11.896916	19.013281
H	11.272037	20.725959	12.734182
H	11.214040	24.403599	11.689969
H	11.146835	20.675846	18.247381
H	11.188421	24.345254	19.305286
H	11.416142	8.944013	18.761001
H	11.471729	8.875814	13.607487
H	11.494205	5.241117	12.389307

H	11.466455	5.339452	20.031233
H	11.585285	15.335716	11.718590
H	11.721217	14.098004	19.958496
H	12.108612	22.680695	14.086280
H	12.053953	22.615364	16.902994
H	12.110722	25.006834	13.102674
H	12.183050	16.411823	20.779401
H	12.101359	24.936135	17.898319
H	12.205848	13.116256	10.891295
H	12.440845	22.334472	10.353157
H	12.398285	6.888595	14.834246
H	12.375263	6.945281	17.561492
H	12.333088	22.111491	20.611876
H	12.441757	4.607959	13.754065
H	16.110615	11.549559	5.565390
H	12.434782	4.686769	18.690632
H	12.622312	19.881998	15.968167
H	16.815245	12.896876	8.803406
H	12.650563	18.644005	12.920238
H	12.630064	7.352439	11.104603
H	12.563904	7.543316	21.271381
H	13.034245	10.906755	18.710088
H	13.138020	12.352777	20.789298
H	17.046271	12.294074	3.386320
H	12.863287	17.191472	10.906468
H	15.847193	11.728609	7.864142
H	15.216542	13.351672	8.214441
H	14.501715	25.179826	14.151048
H	14.464450	25.146952	16.932129
H	14.609250	22.202986	9.163250
H	14.620018	16.190327	20.795062
H	14.477084	21.933569	21.835505
H	14.681816	13.513867	10.879451
H	14.762315	7.601966	9.871316
H	14.680334	7.850758	22.517795
H	14.792637	4.575148	17.639034
H	14.818220	4.532419	14.858377
H	14.881307	10.519038	13.033823
H	15.980701	11.652640	20.012942
H	15.981377	9.921877	17.635670
H	18.218306	14.511068	3.233342
H	13.418716	17.513954	6.483786
H	15.419597	16.059405	6.365216
H	16.294901	18.836685	12.928363
H	16.736876	22.449799	10.405543
H	16.903320	25.126010	13.107111
H	16.622447	22.307058	20.658443
H	13.121693	19.070431	8.424283
H	16.739524	25.138856	18.082654

H	16.952474	22.825901	14.148822
H	16.917790	22.885298	16.950681
H	17.077440	16.573797	20.741120
H	16.924601	7.396318	11.057885
H	16.858224	7.598057	21.368839
H	17.326670	18.905291	10.577658
H	17.180874	4.678255	13.871649
H	17.140062	4.765061	18.631871
H	20.389985	14.007521	7.072211
H	17.257319	7.031553	14.779603
H	17.236344	7.096595	17.669876
H	19.026218	13.109144	13.022855
H	20.210784	14.342149	8.800171
H	18.450159	15.917721	5.253217
H	17.656827	15.571610	11.702072
H	14.919410	19.254848	10.130509
H	17.687248	14.398737	19.943239
H	17.863924	24.520232	11.738593
H	17.687174	24.516405	19.451699
H	17.850991	10.856851	15.180874
H	17.895674	20.868628	12.898009
H	18.154623	17.361558	10.130552
H	17.906081	20.919949	18.169732
H	18.071162	5.210858	12.426815
H	18.014728	5.328962	20.074203
H	18.145394	8.915471	13.338697
H	18.118259	9.009511	19.077368
H	18.305380	11.602272	18.283544
H	18.568283	24.554369	13.374387
H	18.491133	18.689159	15.320513
H	21.810901	14.505759	8.015516
H	18.534733	21.971858	11.649473
H	18.433747	24.648868	17.839389
H	18.468866	22.011528	19.461527
H	18.792617	7.706711	12.198198
H	18.749380	7.823080	20.249993
H	18.853794	5.265166	14.026945
H	18.816976	5.342217	18.483219
H	19.126804	22.081136	13.318049
H	19.093010	22.198773	17.810617
H	17.903298	18.414624	7.254641
H	19.386490	7.756527	13.869517
H	19.358430	7.831879	18.585399
H	20.622467	16.505755	11.898002
H	20.034947	13.898140	20.453062
H	20.184953	19.825059	16.545595
H	20.500831	10.564325	15.243024
H	22.519255	16.575662	7.201118
H	22.292093	11.808630	10.747433

H	20.857631	18.812409	21.301640
H	21.808730	17.833468	11.792801
H	21.932779	16.452947	10.690892
H	22.412166	14.282241	10.692310
H	21.337143	12.736740	20.793848
H	21.186400	14.200218	21.781326
H	19.803116	19.888781	6.655301
H	21.194085	16.377955	21.588589
H	21.475146	20.363791	15.449579
H	21.257004	19.876272	19.102521
H	21.914520	9.568252	14.794742
H	22.283373	10.609142	12.911732
H	21.526147	20.833948	17.157156
H	21.572667	9.910270	16.500952
H	22.332876	16.469003	13.745866
H	21.963136	13.992495	18.805624
H	21.900072	18.035474	15.933381
H	22.072135	12.275905	16.256247
H	22.137157	18.964237	6.630212
H	24.228176	17.474391	12.481528
H	24.386240	16.000629	11.493633
H	23.758198	13.338933	20.410667
H	23.605752	14.899911	21.255039
H	23.859708	19.592347	15.789615
H	23.833045	19.812402	17.557490
H	24.297476	10.570802	14.965328
H	23.964948	10.705077	16.710505
H	24.752499	15.948303	13.233171
H	24.223504	14.846037	19.589561
H	24.249471	18.229626	16.860380
H	24.603822	14.447597	15.295724
H	24.330952	15.994923	17.580757
H	24.555226	12.117007	15.805856
H	4.828918	17.788443	16.408288
H	4.864100	15.451462	17.057309
H	4.874196	14.964912	12.848213
H	4.846190	13.726352	14.886007
H	5.002721	14.208142	19.097766
H	4.918604	11.370115	15.536078
H	5.219517	14.711455	11.121500
H	5.326264	16.345116	11.823205
H	5.270220	19.030264	15.213712
H	5.284478	19.418494	16.952864
H	5.427947	14.448088	20.808208
H	5.543407	12.827172	20.078640
H	5.433655	9.752746	15.007493
H	5.454755	10.174003	16.739081
H	7.174956	17.446322	15.518911
H	7.282800	15.646224	13.217699

H	7.409089	13.597938	18.599694
H	7.269275	11.840093	16.345102
H	7.568690	15.195779	10.183800
H	7.578031	13.050932	10.554276
H	7.583685	19.526633	18.606538
H	7.722592	16.712537	11.086476
H	7.728424	16.173402	21.282734
H	7.680666	19.739783	14.882610
H	7.692171	20.297601	16.565390
H	7.832800	14.025297	21.620830
H	7.647128	9.726628	13.269644
H	7.985157	12.522266	20.695177
H	7.897163	18.609189	20.884917
H	7.844790	10.627466	10.971352
H	7.906896	9.582058	17.000345
H	7.903471	9.003146	15.324469
H	8.943507	15.440809	11.295722
H	8.960236	19.180935	15.986925
H	9.156402	13.831726	20.439832
H	9.124237	10.194245	15.851957
H	15.846650	18.896123	19.228571

4TSd'$_{\text{methyl-meta}}$

E = -2284.3944 eV

Rh	9.731686	14.513175	16.067806
Rh	9.967923	16.682041	14.520112
Rh	10.289254	12.382971	17.608036
Rh	10.801685	16.960148	16.983038
Rh	10.968266	14.781262	18.572173
Rh	10.742588	14.363062	13.467005
Rh	10.908083	12.157088	15.059624
Rh	11.849068	17.221052	19.414259
Rh	11.733884	12.007007	12.551046
Rh	12.061415	18.389480	14.919890
Rh	11.922758	16.801223	12.682295
Rh	12.239631	15.715922	15.164274
Rh	12.410589	12.460908	19.297290
Rh	12.371992	13.521057	16.723776
Rh	12.491437	10.884339	16.975482
Rh	12.967915	18.622994	17.510781
Rh	13.238815	15.048992	20.232322
Rh	12.860876	14.303282	11.690647
Rh	13.176647	15.928609	17.689057
Rh	13.146389	13.389146	14.151385
Rh	13.150644	10.707619	14.353465
Rh	14.295858	19.918180	15.538156
Rh	14.292995	18.372803	13.207117
Rh	14.528164	17.416342	19.469194
Rh	14.360241	17.331994	15.682231

Rh	14.182539	16.733702	11.020335
Rh	14.390053	15.736898	13.449746
Rh	14.505525	14.708533	15.885227
Rh	14.642919	13.678643	18.215390
Rh	14.772706	12.833223	20.593552
Rh	14.631393	12.084824	16.014778
Rh	14.456301	12.049628	12.266073
Rh	14.691786	9.502867	16.226573
Rh	14.795970	11.038003	18.549964
Rh	15.814328	18.755538	17.383737
Rh	15.853845	16.058130	17.593251
Rh	15.837771	13.526732	14.029824
Rh	16.103085	15.123782	20.073607
Rh	15.729596	14.431904	11.556176
Rh	15.997117	10.773581	14.209675
Rh	16.549887	18.555099	14.729045
Rh	16.570982	16.933448	12.347966
Rh	16.639662	15.922329	15.015998
Rh	16.780319	13.721602	16.545341
Rh	16.992591	12.659090	19.054736
Rh	16.971404	11.019675	16.822241
Rh	17.228395	17.437511	19.203362
Rh	17.152106	12.208149	12.311303
Rh	18.079865	17.314495	16.674229
Rh	18.257679	15.081861	18.277861
Rh	18.089082	14.652357	13.141452
Rh	18.233536	12.454244	14.750681
Rh	18.724971	17.019874	14.124867
Rh	19.016785	12.759781	17.234828
Rh	19.319444	14.968669	15.773885
N	14.418414	22.836744	14.398702
N	14.367057	22.879208	16.575830
N	14.736734	6.539128	15.164833
N	14.718598	6.578364	17.342878
N	22.282326	14.415036	15.158734
N	22.108151	15.618796	16.975814
N	6.849873	15.164097	16.947290
N	6.844782	13.788617	15.245294
P	19.079742	16.943430	7.949057
C	10.789289	21.559084	12.503433
C	10.673826	21.654270	18.399308
C	10.965782	7.734441	18.983038
C	11.041373	7.674431	13.429686
C	11.379912	24.019310	12.666721
C	11.264388	24.106923	18.162933
C	11.630202	5.205625	13.509829
C	11.607959	5.279406	18.955987
C	11.872028	22.572226	12.936803
C	11.766923	22.652524	17.960442

C	12.135029	6.645891	13.788737
C	12.088843	6.724424	18.660465
C	13.239996	22.335491	12.281290
C	13.321334	22.046221	10.906126
C	13.112651	22.446889	18.668487
C	13.145195	22.207246	20.055187
C	13.469273	6.896473	13.073901
C	13.406387	7.013912	19.391964
C	19.633982	13.832835	5.266037
C	13.485074	7.116212	11.683612
C	13.392980	7.295595	20.770716
C	18.937600	13.807787	7.690257
C	19.983295	14.469650	4.067470
C	19.343411	14.558345	6.438357
C	14.396149	24.177441	14.780592
C	14.464067	22.472642	12.989337
C	14.365896	24.204064	16.139861
C	14.555151	21.923398	10.260981
C	14.401663	21.986332	15.503247
C	14.359878	22.575400	18.000445
C	14.353815	22.125032	20.751855
C	14.686065	7.253231	10.983787
C	14.705365	7.431070	16.237817
C	14.721599	6.840003	13.740161
C	14.672996	6.934809	18.755115
C	14.579342	7.468904	21.487818
C	14.760779	5.214080	15.598768
C	14.748032	5.238973	16.958181
C	20.046906	15.868258	4.023177
C	15.742671	22.109310	10.973737
C	15.730689	22.397144	12.351561
C	19.407648	15.982648	6.391512
C	15.599667	22.550218	18.692105
C	15.563163	22.310418	20.078797
C	15.905430	7.151658	11.658493
C	15.812983	7.343258	20.843813
C	15.958393	6.933569	13.048247
C	15.895473	7.065332	19.466539
C	19.758782	16.610720	5.176802
C	15.085112	16.632810	6.836480
C	16.483118	16.705332	6.818333
C	14.396432	16.803008	8.044433
C	17.223281	16.990554	7.988074
C	17.047858	22.694149	13.081535
C	16.939109	22.859930	18.011195
C	15.115490	17.051267	9.227342
C	16.529887	17.236721	9.218785
C	17.313815	6.730600	13.738698
C	17.266362	6.844799	18.812586

C	17.521251	24.141266	12.782714
C	17.391175	24.308252	18.336278
C	17.200965	17.741797	10.458686
C	17.819367	5.276876	13.541276
C	17.777506	5.405695	19.087288
C	22.066729	17.949130	7.644193
C	18.176211	21.689457	12.759282
C	18.059277	21.859875	18.368964
C	18.397538	7.730248	13.278538
C	18.331956	7.875837	19.247199
C	19.596610	18.672654	7.524401
C	20.985647	19.000253	7.496369
C	18.649994	19.707384	7.360734
C	21.359636	20.344046	7.301402
C	19.045605	21.038784	7.172327
C	20.408488	21.360827	7.141807
C	21.380872	16.710278	11.772869
C	21.069086	13.544581	20.617247
C	21.114512	19.822747	16.446542
C	21.316775	15.009265	15.985054
C	21.396820	10.191250	15.286445
C	21.975569	12.318142	11.486061
C	21.112150	17.941231	20.390741
C	22.053643	13.712482	11.491918
C	21.295927	16.562584	20.515012
C	22.019333	11.608370	12.689570
C	21.297089	18.562765	19.152630
C	22.143411	14.433130	12.697865
C	21.623612	15.762078	19.402908
C	21.622598	17.822117	18.000364
C	22.102967	12.270078	13.929005
C	22.112317	13.690997	13.907237
C	21.717381	16.408661	18.137815
C	22.385283	15.947914	12.660021
C	21.988047	14.285182	19.620608
C	21.963146	18.557000	16.695166
C	22.281437	11.455375	15.218321
C	23.840351	16.250703	12.213905
C	23.575643	14.660130	15.617428
C	23.467134	15.403658	16.745819
C	23.460434	14.158586	20.098401
C	23.466820	18.944400	16.660037
C	23.768659	11.056765	15.415130
C	5.318938	15.059381	12.176757
C	5.311552	18.481400	16.336177
C	5.600892	13.765958	20.061976
C	5.517751	14.876595	16.649097
C	5.514706	14.022936	15.595177
C	5.537171	10.406965	15.947044

C	6.829421	14.874095	12.485280
C	6.807104	18.070837	16.406630
C	7.077260	14.063100	19.683685
C	7.004200	10.892515	15.798518
C	7.147087	16.078316	18.043556
C	7.133946	12.886044	14.136839
C	7.141736	17.476672	17.782792
C	7.140831	13.406163	12.812822
C	7.266756	15.551952	19.359237
C	7.232426	11.492699	14.404048
C	7.359572	18.339368	18.874453
C	7.356491	12.494059	11.761478
C	7.481492	16.469339	20.405648
C	7.444883	10.635028	13.307590
C	7.543271	17.844461	20.167633
C	7.524082	11.128537	12.003308
C	7.660838	15.442597	11.313842
C	7.726427	14.491562	16.082049
C	7.691593	19.275322	16.013641
C	8.005417	13.544671	20.804299
C	7.969704	9.738830	16.149150
H	9.876472	21.717892	13.099858
H	9.779961	21.785315	17.768879
H	10.088990	7.528106	18.348467
H	10.143911	7.492852	14.042564
H	10.422476	24.198443	13.182829
H	10.509311	21.678379	11.444837
H	10.320622	24.264779	17.615893
H	10.362470	21.811293	19.444243
H	10.272013	17.384753	12.902145
H	10.701318	5.010563	14.070245
H	10.738132	7.610299	12.372847
H	10.633272	7.666844	20.030837
H	10.690568	5.057926	18.386476
H	10.782267	11.769858	19.197623
H	11.117816	20.522459	12.657145
H	11.219081	24.183374	11.588982
H	11.008045	20.610355	18.292528
H	11.076427	24.311269	19.229318
H	11.286314	8.769213	18.795028
H	11.375169	8.707285	13.625936
H	11.415589	5.063074	12.438508
H	11.380053	5.152658	20.026550
H	11.288181	15.247550	11.884372
H	11.703577	13.965884	20.111278
H	11.997821	22.459161	14.024125
H	11.926311	22.499204	16.882716
H	12.097805	24.775497	13.017182
H	12.234576	16.287595	20.923895

H	11.990702	24.850248	17.802477
H	11.840530	13.038585	11.050149
H	12.403876	21.916061	10.329633
H	12.309605	6.734132	14.870940
H	12.280563	6.797660	17.579813
H	12.207903	22.088252	20.601527
H	12.369337	4.446251	13.806701
H	19.580765	12.740251	5.298171
H	12.364095	4.527778	18.683921
H	12.514476	19.753704	16.135997
H	19.435897	14.208137	8.587303
H	12.413174	18.534705	13.094690
H	12.540952	7.176963	11.138704
H	12.437804	7.379558	21.292299
H	12.936410	10.763461	18.803725
H	13.136519	12.203543	20.885039
H	20.202134	13.875723	3.176199
H	12.511327	17.188854	11.041700
H	19.178491	12.738062	7.601886
H	17.852963	13.891770	7.872354
H	14.405510	24.980843	14.054260
H	14.347290	25.035172	16.834173
H	14.590669	21.688049	9.194178
H	14.655605	16.027522	20.846650
H	14.351707	21.927111	21.826760
H	14.321429	13.382838	10.929478
H	14.672532	7.432434	9.905688
H	14.542423	7.697082	22.556046
H	14.753984	4.434788	17.684025
H	14.782513	4.384034	14.903001
H	14.587559	10.381407	13.069463
H	15.947381	11.483770	20.002013
H	15.847411	9.772958	17.634014
H	20.310928	16.383169	3.095634
H	14.535198	16.430622	5.912634
H	17.008026	16.546158	5.875703
H	16.080448	18.690228	12.941168
H	16.697042	22.030838	10.449361
H	16.762578	24.890067	13.055574
H	16.497956	22.270538	20.641288
H	13.307677	16.714223	8.075861
H	16.632389	25.051877	18.049574
H	16.857431	22.621583	14.162645
H	16.790345	22.794187	16.923452
H	17.115265	16.398771	20.694403
H	16.835066	7.241841	11.093872
H	16.730545	7.465178	21.422107
H	16.950861	18.802184	10.616190
H	17.100821	4.532456	13.914580

H	17.073041	4.639406	18.731177
H	22.217626	17.393558	6.703398
H	17.168308	6.891828	14.817425
H	17.140455	6.954121	17.724872
H	18.742802	12.943410	12.936205
H	21.800503	17.203859	8.410051
H	19.798560	17.701076	5.129808
H	17.338681	15.412397	11.624791
H	14.231677	18.150160	10.301042
H	17.677602	14.218212	19.881668
H	17.746622	24.267385	11.711585
H	17.585346	24.427865	19.414274
H	17.631189	10.690581	15.115529
H	17.865049	20.654672	12.962969
H	18.294481	17.615444	10.431539
H	17.765139	20.824089	18.139508
H	17.997522	5.065843	12.474484
H	17.931674	5.244189	20.166393
H	18.054275	8.771367	13.370271
H	17.982282	8.906510	19.100387
H	18.197915	11.422058	18.197113
H	18.438725	24.367368	13.350182
H	18.346382	18.506934	15.244540
H	23.027655	18.407714	7.920528
H	18.502959	21.751398	11.709332
H	18.321499	24.548192	17.796231
H	18.333864	21.907337	19.434640
H	18.702447	7.559766	12.233760
H	18.617796	7.754884	20.304092
H	18.769727	5.127341	14.079113
H	18.741565	5.239815	18.579294
H	19.054942	21.906759	13.387774
H	18.963637	22.092855	17.785170
H	17.585936	19.464267	7.380464
H	19.296787	7.615325	13.904138
H	19.245341	7.738221	18.646377
H	20.342950	16.537952	12.114053
H	20.009795	13.634163	20.339595
H	20.039113	19.619713	16.538482
H	20.335842	10.431182	15.123495
H	22.424661	20.595265	7.280155
H	21.900830	11.779972	10.537510
H	20.847634	18.537647	21.267784
H	21.583023	17.792499	11.813000
H	21.428460	16.403802	10.718091
H	22.058579	14.251525	10.542341
H	21.325231	12.473261	20.628297
H	21.189316	13.916639	21.646883
H	18.289149	21.818024	7.044962

H	21.189973	16.097885	21.496491
H	21.299071	20.195077	15.426191
H	21.194139	19.646516	19.081604
H	21.700456	9.433404	14.546838
H	21.994058	10.517676	12.664746
H	21.377736	20.636837	17.140613
H	21.487156	9.729198	16.282561
H	22.267440	16.335941	13.681815
H	21.900664	13.769205	18.651333
H	21.767378	17.865924	15.861080
H	21.989891	12.099620	16.063315
H	20.732055	22.394942	6.997085
H	24.032507	17.335209	12.252757
H	24.017503	15.913067	11.180402
H	23.732101	13.096228	20.208066
H	23.592911	14.643980	21.078689
H	23.704873	19.449684	15.709686
H	23.706885	19.638985	17.481333
H	24.107169	10.399631	14.597745
H	23.892767	10.508583	16.363285
H	24.579394	15.752396	12.859818
H	24.173701	14.617091	19.399178
H	24.130033	18.073496	16.750323
H	24.446682	14.293093	15.089464
H	24.226003	15.803126	17.405691
H	24.436893	11.929109	15.442293
H	4.636502	17.640493	16.550510
H	4.701356	15.307208	17.214758
H	4.675558	14.737939	13.007630
H	4.695076	13.557252	15.063122
H	4.897592	14.047971	19.265917
H	4.810896	11.212214	15.765938
H	5.031428	14.480929	11.284022
H	5.101969	16.120933	11.974989
H	5.068584	18.860971	15.330430
H	5.092242	19.281166	17.061917
H	5.315403	14.313707	20.974585
H	5.470361	12.689562	20.259476
H	5.321266	9.594101	15.234940
H	5.364968	10.020456	16.964718
H	6.976013	17.286565	15.652027
H	7.070564	15.474785	13.376789
H	7.315900	13.490467	18.773551
H	7.171772	11.689698	16.539248
H	7.371188	14.999162	10.348056
H	7.389727	12.859584	10.734299
H	7.377356	19.418021	18.709655
H	7.494346	16.528161	11.235099
H	7.597545	16.100608	21.425761

H	7.473583	19.572947	14.975649
H	7.502106	20.153982	16.650449
H	7.735985	13.957949	21.789090
H	7.540128	9.561231	13.477138
H	7.919109	12.449348	20.878211
H	7.718185	18.534288	20.997310
H	7.698242	10.444075	11.168965
H	7.821430	9.436622	17.198136
H	7.795044	8.846903	15.526724
H	8.738095	15.282164	11.459958
H	8.763524	19.028787	16.076706
H	9.060474	13.781850	20.606110
H	9.022369	10.042191	16.029337
H	15.833044	18.740888	19.240398

4Id'$_{methyl-meta}$

E = -2284.9853 eV

Rh	9.762824	14.559521	16.046494
Rh	10.005164	16.741532	14.513813
Rh	10.277973	12.421489	17.582369
Rh	10.835699	17.007432	16.973427
Rh	10.985092	14.816851	18.563841
Rh	10.752991	14.400272	13.450445
Rh	10.884510	12.201675	15.037490
Rh	11.874616	17.257533	19.399769
Rh	11.714700	12.048929	12.542333
Rh	12.118246	18.413792	14.890783
Rh	11.947553	16.812913	12.654803
Rh	12.252503	15.743213	15.166097
Rh	12.392161	12.479605	19.274547
Rh	12.367494	13.555799	16.693112
Rh	12.461838	10.917463	16.951657
Rh	12.998406	18.643942	17.488695
Rh	13.249485	15.068947	20.220050
Rh	12.857602	14.299806	11.638501
Rh	13.193251	15.939143	17.697816
Rh	13.152147	13.413542	14.128625
Rh	13.123476	10.733723	14.327160
Rh	14.340117	19.929986	15.521676
Rh	14.336070	18.394919	13.142221
Rh	14.546872	17.446835	19.471636
Rh	14.404007	17.351711	15.692681
Rh	14.218207	16.633956	11.005691
Rh	14.431217	15.758595	13.444389
Rh	14.518907	14.740807	15.850162
Rh	14.638296	13.663355	18.198121
Rh	14.749770	12.820877	20.587111
Rh	14.613868	12.101917	15.998148
Rh	14.445358	12.073313	12.226377

Rh	14.649627	9.522008	16.174103
Rh	14.760983	11.026907	18.527306
Rh	15.856826	18.746363	17.379200
Rh	15.859757	16.037928	17.624229
Rh	15.838891	13.535749	14.016257
Rh	16.102909	15.102625	20.077833
Rh	15.771037	14.466858	11.418820
Rh	15.962255	10.803686	14.164350
Rh	16.591993	18.559372	14.721272
Rh	16.642301	16.879238	12.434457
Rh	16.684323	15.925076	15.055442
Rh	16.795497	13.721425	16.541191
Rh	16.965995	12.628010	19.032272
Rh	16.929079	10.999791	16.793671
Rh	17.245832	17.416170	19.227622
Rh	17.130197	12.267688	12.263336
Rh	18.103103	17.304594	16.712892
Rh	18.254147	15.039143	18.291310
Rh	18.133399	14.645310	13.188896
Rh	18.213024	12.425544	14.728599
Rh	18.783852	17.008929	14.172184
Rh	19.005642	12.699805	17.210112
Rh	19.321682	14.926810	15.784676
N	14.454087	22.836199	14.317092
N	14.410343	22.915360	16.492451
N	14.607604	6.562651	15.101550
N	14.615756	6.591384	17.279839
N	22.288677	14.364250	15.137541
N	22.122248	15.491957	17.002951
N	6.891125	15.195306	16.991793
N	6.856817	13.789775	15.315556
P	19.214599	17.127523	8.009075
C	10.808190	21.524597	12.452238
C	10.715504	21.716475	18.327496
C	10.901561	7.772318	18.962108
C	10.910711	7.748241	13.394987
C	11.394193	23.981788	12.645236
C	11.301079	24.165569	18.042119
C	11.471479	5.273833	13.479653
C	11.502626	5.305499	18.934845
C	11.893573	22.532871	12.889739
C	11.808071	22.708547	17.872466
C	11.995863	6.708697	13.748720
C	12.003535	6.742168	18.630676
C	13.255438	22.308772	12.217108
C	13.322840	22.019735	10.841618
C	13.152029	22.521102	18.589015
C	13.181477	22.310439	19.980137
C	13.326847	6.941035	13.021955

C	13.332090	7.014213	19.348706
C	20.839064	14.413067	5.328912
C	13.334123	7.160465	11.631566
C	13.336717	7.282200	20.730422
C	20.127128	14.116707	7.728237
C	20.974327	15.144552	4.141532
C	20.275045	14.976449	6.490444
C	14.441482	24.183173	14.676882
C	14.486700	22.455401	12.911049
C	14.415239	24.232934	16.035386
C	14.550071	21.902568	10.183158
C	14.438540	22.004479	15.435693
C	14.400851	22.637227	17.921880
C	14.388793	22.246403	20.681279
C	14.531069	7.280424	10.921684
C	14.610283	7.450584	16.178814
C	14.584051	6.867186	13.677651
C	14.590402	6.937179	18.695409
C	14.532416	7.444847	21.434065
C	14.606920	5.235195	15.528572
C	14.610453	5.253588	16.888185
C	20.533811	16.473459	4.097951
C	15.744556	22.091093	10.882569
C	15.746294	22.381255	12.260112
C	19.831948	16.332425	6.447361
C	15.639550	22.623633	18.615049
C	15.599919	22.416520	20.006999
C	15.754476	7.161142	11.586272
C	15.757737	7.321372	20.773469
C	15.816008	6.943097	12.975498
C	15.822320	7.056006	19.392839
C	19.968575	17.054597	5.241344
C	15.657751	15.085096	7.293965
C	16.878735	15.746498	7.116683
C	15.042353	15.081822	8.552012
C	17.492447	16.461092	8.172568
C	17.071431	22.679987	12.974609
C	16.981485	22.905250	17.926466
C	15.613941	15.799464	9.645124
C	16.827128	16.555132	9.430908
C	17.173886	6.723437	13.655651
C	17.182997	6.836855	18.717690
C	17.562601	24.112293	12.636293
C	17.453367	24.353109	18.222293
C	17.320266	17.464040	10.521955
C	17.659363	5.263082	13.457537
C	17.675694	5.381959	18.938277
C	21.557938	19.226524	7.905419
C	18.183506	21.651503	12.670858

C	18.089003	21.896237	18.299161
C	18.265244	7.709170	13.184533
C	18.269825	7.835668	19.174626
C	19.026484	18.911779	7.546186
C	20.173288	19.759529	7.596118
C	17.770259	19.477386	7.241409
C	20.007958	21.133117	7.331195
C	17.628934	20.848239	6.984163
C	18.754076	21.681855	7.028681
C	21.474027	16.847842	11.883493
C	21.060575	13.300222	20.563854
C	21.143959	19.721733	16.622374
C	21.327236	14.936839	15.984582
C	21.313256	10.152313	15.022689
C	21.998948	12.462205	11.357720
C	21.138245	17.702964	20.493795
C	22.111139	13.851813	11.436300
C	21.314784	16.319944	20.571814
C	22.002936	11.692159	12.524085
C	21.322830	18.364608	19.276328
C	22.193947	14.508219	12.678528
C	21.637144	15.555454	19.432893
C	21.642325	17.661194	18.099203
C	22.080140	12.287902	13.796991
C	22.122092	13.707482	13.848672
C	21.733596	16.243916	18.190100
C	22.470725	16.017022	12.716890
C	21.990920	14.069179	19.599659
C	21.975069	18.435055	16.814263
C	22.215327	11.404938	15.045630
C	23.926726	16.310753	12.268778
C	23.582829	14.568579	15.614075
C	23.479176	15.265213	16.772389
C	23.460125	13.914200	20.080639
C	23.484147	18.798263	16.761465
C	23.691828	10.975542	15.259272
C	5.309866	15.017722	12.247153
C	5.331271	18.493614	16.362203
C	5.681231	13.852766	20.156406
C	5.554867	14.893468	16.729967
C	5.533604	14.021278	15.691505
C	5.535826	10.415678	16.108889
C	6.822305	14.817044	12.536297
C	6.830222	18.091944	16.406466
C	7.150768	14.140602	19.746553
C	6.998331	10.904418	15.930948
C	7.203328	16.127525	18.068924
C	7.123521	12.860778	14.223154
C	7.193260	17.521345	17.785328

C	7.117520	13.351710	12.888501
C	7.337651	15.623341	19.392091
C	7.209197	11.472431	14.520129
C	7.426352	18.402607	18.858853
C	7.304900	12.414219	11.854386
C	7.566207	16.558641	20.419817
C	7.393358	10.588352	13.439734
C	7.627090	17.929371	20.157587
C	7.458100	11.052244	12.123825
C	7.645603	15.350459	11.343319
C	7.752305	14.515660	16.116345
C	7.699202	19.294913	15.976235
C	8.096745	13.641406	20.861038
C	7.972992	9.763529	16.297571
H	9.904197	21.667776	13.066040
H	9.824736	21.830657	17.689459
H	10.012762	7.577088	18.340846
H	10.015688	7.576835	14.014123
H	10.438760	24.149032	13.168836
H	10.510835	21.665465	11.400931
H	10.358982	24.309053	17.488211
H	10.398172	21.895872	19.367089
H	10.314652	17.399503	12.861513
H	10.543314	5.093305	14.046087
H	10.599439	7.684921	12.340332
H	10.582751	7.715990	20.014838
H	10.576164	5.097246	18.375246
H	10.752129	11.821478	19.191960
H	11.142483	20.486248	12.578220
H	11.226974	24.161307	11.570871
H	11.054252	20.672087	18.245503
H	11.108126	24.392081	19.103238
H	11.236619	8.800629	18.764256
H	11.256450	8.777798	13.586889
H	11.248674	5.129209	12.410244
H	11.283798	5.184554	20.007947
H	11.284923	15.267516	11.872700
H	11.708549	13.992275	20.097024
H	12.030739	22.403622	13.973886
H	11.972021	22.532963	16.798850
H	12.110687	24.735336	13.003674
H	12.263267	16.308287	20.914438
H	12.026732	24.903069	17.668712
H	11.852549	13.046065	11.007643
H	12.399652	21.883463	10.275879
H	12.180767	6.799242	14.828993
H	12.185388	6.808892	17.547852
H	12.242982	22.200881	20.526320
H	12.202052	4.506022	13.775641

H	21.181190	13.373815	5.362454
H	12.244155	4.541575	18.656786
H	12.562078	19.784271	16.122506
H	20.330230	14.688333	8.646929
H	12.476537	18.550724	13.054011
H	12.386601	7.234237	11.094585
H	12.388376	7.364310	21.264744
H	12.897127	10.776794	18.778995
H	13.109249	12.200484	20.859902
H	21.420474	14.678190	3.259435
H	12.502364	16.997834	10.991735
H	20.807930	13.253039	7.690931
H	19.099466	13.727690	7.824773
H	14.454333	24.973577	13.936292
H	14.403382	25.075741	16.715893
H	14.575560	21.667726	9.116036
H	14.668506	16.036296	20.839606
H	14.384344	22.073349	21.760453
H	14.313897	13.383056	10.894154
H	14.510977	7.459885	9.843809
H	14.509755	7.663046	22.504750
H	14.606120	4.445723	17.609717
H	14.601394	4.408520	14.828518
H	14.555351	10.412336	13.026067
H	15.902588	11.462487	19.986794
H	15.801505	9.766623	17.606082
H	20.624962	17.058761	3.179211
H	15.199688	14.549780	6.457561
H	17.369898	15.704672	6.142127
H	16.127474	18.686660	12.934046
H	16.692868	22.012700	10.347456
H	16.813832	24.877637	12.890559
H	16.533538	22.387585	20.572183
H	14.117046	14.523386	8.701468
H	16.706124	25.100940	17.916371
H	16.887431	22.637861	14.058410
H	16.829191	22.820767	16.840498
H	17.141418	16.359229	20.700317
H	16.680619	7.237994	11.013635
H	16.682731	7.435200	21.341438
H	16.901249	18.475800	10.406121
H	16.935138	4.529249	13.840707
H	16.956838	4.638014	18.564381
H	21.958201	18.631541	7.067275
H	17.038891	6.888732	14.735007
H	17.047250	6.985123	17.635741
H	18.741365	12.921038	12.916569
H	21.545090	18.563411	8.785234
H	19.624052	18.089540	5.194679

H	17.412309	15.313772	11.691170
H	14.342091	18.366637	11.395352
H	17.670116	14.176205	19.875583
H	17.786841	24.207224	11.561702
H	17.645476	24.493644	19.298224
H	17.589465	10.673489	15.082358
H	17.859648	20.626782	12.903809
H	18.421213	17.533351	10.541654
H	17.778198	20.860922	18.090791
H	17.823941	5.046295	12.389651
H	17.838636	5.182529	20.009726
H	17.933873	8.754267	13.276083
H	17.933341	8.876131	19.072305
H	18.157839	11.392129	18.186528
H	18.484418	24.341780	13.195364
H	18.378727	18.491698	15.288858
H	22.261645	20.049604	8.097425
H	18.500058	21.680429	11.616211
H	18.389002	24.568429	17.680791
H	18.368824	21.960288	19.362647
H	18.560136	7.532604	12.137915
H	18.570797	7.669601	20.221288
H	18.613066	5.103230	13.986444
H	18.631852	5.219569	18.414499
H	19.071057	21.873583	13.285409
H	18.994038	22.104207	17.707039
H	16.889274	18.832981	7.205595
H	19.168079	7.586432	13.803487
H	19.171511	7.708057	18.554294
H	20.434939	16.669797	12.220423
H	20.003849	13.405245	20.281580
H	20.066708	19.531701	16.724092
H	20.261171	10.415112	14.837354
H	20.886395	21.785039	7.368148
H	21.928694	11.974037	10.382256
H	20.878236	18.271291	21.390556
H	21.692879	17.922260	11.991077
H	21.511774	16.606776	10.810659
H	22.152197	14.438259	10.516707
H	21.311271	12.227777	20.540389
H	21.175509	13.636571	21.606344
H	16.644162	21.259007	6.745950
H	21.207260	15.823038	21.537015
H	21.317835	20.125093	15.612134
H	21.222675	19.450443	19.241204
H	21.631214	9.426765	14.257047
H	21.951401	10.605224	12.442988
H	21.430244	20.507774	17.339261
H	21.363426	9.641090	15.997319

H	22.373835	16.353864	13.758672
H	21.905701	13.589193	18.611813
H	21.751313	17.775511	15.961652
H	21.908588	12.008411	15.915143
H	18.660016	22.752746	6.830992
H	24.145160	17.386885	12.363628
H	24.084106	16.026410	11.216161
H	23.725270	12.846758	20.148192
H	23.589293	14.358494	21.080596
H	23.711996	19.327230	15.821463
H	23.752166	19.464673	17.597395
H	24.044330	10.360688	14.415377
H	23.782820	10.373532	16.177937
H	24.661337	15.760874	12.876820
H	24.180266	14.397050	19.405292
H	24.134158	17.914333	16.812497
H	24.451352	14.212262	15.074507
H	24.240304	15.625468	17.451995
H	24.369494	11.835746	15.353892
H	4.665812	17.652499	16.603976
H	4.748541	15.328765	17.306337
H	4.676419	14.725633	13.096539
H	4.705418	13.541580	15.185634
H	4.963563	14.119072	19.367773
H	4.803105	11.212124	15.915144
H	4.998992	14.422036	11.373711
H	5.104833	16.077238	12.023402
H	5.064190	18.856363	15.356444
H	5.123134	19.302923	17.080487
H	5.412707	14.419473	21.062658
H	5.553677	12.780888	20.378847
H	5.316052	9.583438	15.420886
H	5.377593	10.055002	17.138211
H	6.987743	17.296616	15.661089
H	7.085205	15.430960	13.412195
H	7.373261	13.550538	18.843368
H	7.170541	11.719308	16.651170
H	7.337769	14.892068	10.390161
H	7.326623	12.756966	10.818932
H	7.440516	19.478330	18.675789
H	7.493601	16.436591	11.245211
H	7.694168	16.207427	21.444714
H	7.458055	19.574491	14.938345
H	7.517494	20.182865	16.602328
H	7.842629	14.071873	21.842587
H	7.477511	9.517356	13.631292
H	8.013055	12.547517	20.955006
H	7.813289	18.633017	20.973176
H	7.610117	10.347279	11.302274

H	7.838907	9.486298	17.355135
H	7.794435	8.856347	15.698723
H	8.722721	15.177530	11.478971
H	8.773763	19.055226	16.022091
H	9.148373	13.875707	20.642553
H	9.022592	10.068160	16.156282
H	15.886520	18.746472	19.227492

4A’

E = -599.87479 eV

Rh	14.391975	13.141060	11.382880
Rh	14.242522	12.571490	13.902899
Rh	14.096792	11.942563	16.342907
Rh	15.734885	10.568962	14.747626
Rh	15.847949	11.192527	12.178882
Rh	16.415833	13.759903	12.833530
Rh	14.108877	15.149247	13.099014
Rh	12.102141	13.426365	12.506077
Rh	13.152736	10.980694	11.977949
Rh	14.665783	11.188903	9.746435
Rh	16.695296	12.914573	10.273510
Rh	15.612234	15.345233	10.820923
Rh	12.946974	15.133433	10.619223
Rh	12.339627	12.573927	9.948804
Rh	14.529229	13.761877	8.904598
Rh	17.293653	9.256825	12.980658
Rh	18.465656	14.340377	14.250972
Rh	13.812758	17.077069	14.714648
Rh	9.786436	13.642360	13.584071
Rh	11.932953	8.827293	12.564247
Rh	14.949486	9.258731	8.147511
Rh	18.975733	12.669987	9.193096
Rh	16.851260	17.482137	10.186653
Rh	11.494201	17.058212	9.787748
Rh	10.325590	11.979961	8.526032
Rh	14.668598	14.353806	6.427280
Rh	16.317217	13.183333	15.417266
Rh	13.907211	14.562924	15.661744
Rh	11.875352	12.789225	15.064551
Rh	12.986923	10.329238	14.536024
Rh	17.940320	11.795136	13.696444
Rh	14.617651	8.941858	12.816838
Rh	16.176144	9.153253	10.551515
Rh	18.231161	10.905011	11.100413
Rh	16.204892	15.787400	14.514712
Rh	18.813798	13.522782	11.732623
Rh	17.730200	15.996592	12.270577
Rh	11.687133	15.423620	14.146248
Rh	15.358632	17.400833	12.415674

Rh	12.629659	17.163676	12.238910
Rh	10.791988	11.206196	13.119606
Rh	10.537629	15.404793	11.694840
Rh	9.959511	12.803913	11.049687
Rh	11.054122	10.322222	10.521583
Rh	13.427874	8.927701	10.335796
Rh	17.036439	10.902320	8.608464
Rh	12.596483	10.543082	8.258345
Rh	14.846762	11.772299	7.181863
Rh	17.986114	15.132436	9.661716
Rh	16.893171	13.508647	7.712564
Rh	14.153132	17.391616	9.949653
Rh	15.790740	15.975085	8.246941
Rh	10.840831	14.557007	9.099131
Rh	13.040498	15.757121	8.035293
Rh	12.474534	13.130543	7.366904
P	16.057739	16.738310	19.289314
C	14.960495	17.681405	18.134657
C	17.405947	17.925443	19.753589
C	15.065455	16.578566	20.848645
C	13.773731	17.073601	17.644406
C	15.365733	18.927567	17.579323
C	18.601494	17.959625	18.976409
C	17.319631	18.720450	20.917893
C	15.389231	15.516152	21.743308
C	13.967692	17.417246	21.142367
C	12.966647	17.776904	16.685192
C	13.320762	15.695038	18.052455
C	14.647448	19.550935	16.558427
C	19.652614	18.797587	19.401177
C	18.774021	17.145241	17.710820
C	18.379520	19.542332	21.321595
C	14.588556	15.339860	22.889580
C	16.562938	14.585678	21.509186
C	13.185215	17.220887	22.287945
C	13.423815	18.997779	16.103030
C	19.553301	19.581065	20.558017
C	13.496375	16.173079	23.164673
H	16.272485	19.405241	17.954087
H	16.412191	18.691425	21.523101
H	13.717869	18.232585	20.459570
H	11.927566	17.459006	16.547120
H	12.286128	15.491326	17.748212
H	14.019461	14.890840	17.622215
H	13.413280	15.494417	19.129823
H	15.003233	20.495738	16.140710
H	20.573471	18.825062	18.811231
H	19.832588	17.099598	17.416306
H	18.398378	16.117602	17.833656

H	18.216890	17.586288	16.866344
H	18.286080	20.144345	22.229042
H	14.826104	14.521526	23.575385
H	16.571680	14.193794	20.479886
H	17.524202	15.105908	21.657477
H	16.533039	13.735688	22.205962
H	12.338857	17.881923	22.490858
H	12.760168	19.582619	15.461433
H	20.391748	20.212220	20.863466
H	12.890930	16.000733	24.058017

4TS'$_{methyl}$

E = -599.69171 eV

Rh	14.404476	13.193042	11.413811
Rh	14.267539	12.652792	13.937372
Rh	14.132731	12.071258	16.390924
Rh	15.734628	10.658413	14.814881
Rh	15.855950	11.242388	12.225478
Rh	16.440619	13.820157	12.838178
Rh	14.140769	15.199940	13.105683
Rh	12.122401	13.502482	12.539623
Rh	13.159634	11.040529	12.039267
Rh	14.659549	11.217819	9.797800
Rh	16.698160	12.941497	10.293415
Rh	15.638995	15.387545	10.808153
Rh	12.970162	15.184622	10.619762
Rh	12.340799	12.619185	9.998575
Rh	14.533391	13.775499	8.923292
Rh	17.287393	9.317796	13.047746
Rh	18.494200	14.405109	14.245057
Rh	13.838232	17.195667	14.683331
Rh	9.821445	13.751687	13.628669
Rh	11.936216	8.904497	12.660010
Rh	14.926270	9.263271	8.222958
Rh	18.977957	12.663215	9.205325
Rh	16.888241	17.498488	10.146016
Rh	11.530014	17.099816	9.777875
Rh	10.320777	12.013662	8.585695
Rh	14.667659	14.332624	6.442642
Rh	16.338615	13.294604	15.437474
Rh	13.945655	14.730626	15.797964
Rh	11.917460	12.929051	15.115630
Rh	12.993394	10.450236	14.619055
Rh	17.939440	11.868595	13.743021
Rh	14.608773	9.009954	12.898379
Rh	16.166843	9.181186	10.626269
Rh	18.226505	10.934879	11.143049
Rh	16.236336	15.880847	14.497129
Rh	18.829450	13.551718	11.734026

Rh	17.751322	16.043251	12.256292
Rh	11.736349	15.540323	14.155411
Rh	15.379587	17.456654	12.390988
Rh	12.689421	17.235220	12.216362
Rh	10.810986	11.314053	13.198589
Rh	10.572182	15.463583	11.721750
Rh	9.969378	12.861508	11.087248
Rh	11.057759	10.370884	10.599668
Rh	13.417348	8.962323	10.425506
Rh	17.019084	10.897000	8.659233
Rh	12.588487	10.561994	8.329542
Rh	14.836772	11.762432	7.218700
Rh	18.013790	15.136136	9.652750
Rh	16.896737	13.487856	7.724700
Rh	14.188867	17.432538	9.917187
Rh	15.824477	15.967405	8.233291
Rh	10.854501	14.597371	9.124442
Rh	13.065404	15.776547	8.032737
Rh	12.479577	13.139886	7.404180
P	16.087562	16.607234	19.223100
C	14.992412	17.508190	18.031556
C	17.399443	17.840132	19.682843
C	15.058805	16.499975	20.765740
C	13.823170	16.856702	17.505260
C	15.324069	18.798271	17.554756
C	18.596246	17.906559	18.909884
C	17.287586	18.639940	20.841938
C	15.391259	15.502325	21.728745
C	13.944514	17.336329	20.996290
C	12.935211	17.628135	16.652088
C	13.409876	15.476821	17.862706
C	14.540006	19.478072	16.610886
C	19.621697	18.776875	19.331985
C	18.793908	17.092064	17.648720
C	18.322496	19.494173	21.244055
C	14.586722	15.389618	22.880591
C	16.583868	14.580660	21.565381
C	13.158673	17.204531	22.148577
C	13.309414	18.926481	16.184632
C	19.497338	19.563158	20.484448
C	13.481894	16.223852	23.095781
H	16.216598	19.292186	17.942765
H	16.379424	18.591150	21.444559
H	13.690373	18.105897	20.264154
H	11.897401	17.292031	16.557522
H	12.324691	15.315060	17.841486
H	14.016740	14.242823	17.371270
H	13.811050	15.143378	18.829611
H	14.845232	20.466119	16.258861

H	20.542311	18.828162	18.743238
H	19.855093	17.065868	17.361356
H	18.436857	16.057894	17.773051
H	18.233687	17.520134	16.799260
H	18.208308	20.098652	22.147470
H	14.834096	14.623848	23.621631
H	16.624969	14.143463	20.555435
H	17.533200	15.122508	21.714083
H	16.549000	13.762697	22.299354
H	12.303458	17.867049	22.304650
H	12.587897	19.538351	15.638165
H	20.316171	20.220137	20.788834
H	12.877526	16.106146	23.998744

4I′$_{methyl}$

E = -600.35808 eV

Rh	14.402353	13.192525	11.449856
Rh	14.260698	12.639674	13.967211
Rh	14.100248	11.998276	16.426095
Rh	15.722567	10.617162	14.813560
Rh	15.849313	11.236425	12.246791
Rh	16.430139	13.807665	12.895591
Rh	14.129564	15.211268	13.118660
Rh	12.117958	13.489060	12.571871
Rh	13.155103	11.038972	12.053021
Rh	14.661679	11.243283	9.811751
Rh	16.706884	12.961976	10.349036
Rh	15.639940	15.396050	10.881785
Rh	12.966455	15.191162	10.669025
Rh	12.351265	12.637889	10.019552
Rh	14.549717	13.816531	8.974615
Rh	17.263339	9.295243	13.041019
Rh	18.490031	14.374256	14.307532
Rh	13.799282	17.154437	14.761947
Rh	9.821240	13.731847	13.667415
Rh	11.919582	8.896789	12.631758
Rh	14.923297	9.315221	8.209518
Rh	18.977437	12.708354	9.250569
Rh	16.887803	17.522993	10.249564
Rh	11.524084	17.124390	9.862660
Rh	10.322741	12.061729	8.599313
Rh	14.682766	14.401170	6.490907
Rh	16.335839	13.208711	15.513510
Rh	13.971126	14.688812	15.875419
Rh	11.929078	12.912709	15.156347
Rh	12.993928	10.419715	14.619783
Rh	17.944025	11.854272	13.762777
Rh	14.592210	8.988730	12.871788
Rh	16.155665	9.197715	10.619232

Rh	18.220450	10.939055	11.145698
Rh	16.237544	15.829078	14.554819
Rh	18.829086	13.557384	11.785007
Rh	17.757532	16.038164	12.326630
Rh	11.764268	15.493205	14.252140
Rh	15.395739	17.458080	12.513599
Rh	12.688859	17.248237	12.321098
Rh	10.801022	11.303077	13.223405
Rh	10.582805	15.471239	11.786339
Rh	9.970304	12.874360	11.121649
Rh	11.039968	10.410326	10.611436
Rh	13.387965	8.995818	10.387992
Rh	17.013355	10.952857	8.669980
Rh	12.574699	10.608630	8.325734
Rh	14.815380	11.815784	7.239456
Rh	18.000055	15.170001	9.707057
Rh	16.883440	13.545519	7.767295
Rh	14.187174	17.444396	10.034021
Rh	15.809048	16.025477	8.302009
Rh	10.843758	14.642487	9.186094
Rh	13.066397	15.831253	8.098645
Rh	12.471360	13.215876	7.453737
P	16.030842	16.623470	19.196252
C	14.957614	17.544512	18.008365
C	17.357078	17.838920	19.661238
C	15.003775	16.513973	20.737250
C	13.765244	16.911714	17.486941
C	15.316421	18.821620	17.526707
C	18.559135	17.886147	18.895447
C	17.245876	18.650055	20.812456
C	15.356626	15.532165	21.708872
C	13.876931	17.333864	20.963212
C	12.876906	17.721653	16.666738
C	13.375681	15.524907	17.747447
C	14.542646	19.513974	16.579355
C	19.589917	18.751701	19.314252
C	18.756562	17.055924	17.644551
C	18.286588	19.498097	21.212560
C	14.560314	15.419744	22.866345
C	16.562346	14.627483	21.548733
C	13.099000	17.201871	22.120903
C	13.290279	18.998187	16.177371
C	19.465947	19.549906	20.458471
C	13.443197	16.238404	23.078160
H	16.222144	19.297733	17.906604
H	16.333219	18.614264	21.409544
H	13.606478	18.091219	20.224433
H	11.829903	17.412518	16.583308
H	12.291201	15.347417	17.772301

H	15.171286	13.699764	16.827369
H	13.899083	15.019530	18.562869
H	14.875854	20.486358	16.209725
H	20.513939	18.789583	18.729705
H	19.817814	17.025446	17.357677
H	18.398124	16.023849	17.781569
H	18.197171	17.473780	16.789347
H	18.173664	20.111528	22.110044
H	14.824514	14.666843	23.614713
H	16.604598	14.181596	20.542553
H	17.504202	15.184500	21.688832
H	16.542689	13.815488	22.289836
H	12.233326	17.851357	22.273232
H	12.590035	19.619356	15.614730
H	20.288730	20.202785	20.760963
H	12.845543	16.121487	23.985705

4TS’$_{methyl-meta}$
E = -599.43278 eV

Rh	14.365741	13.112851	11.296353
Rh	14.407162	12.678194	13.841497
Rh	14.448152	12.242434	16.337168
Rh	15.932720	10.712516	14.726681
Rh	15.862350	11.194830	12.124836
Rh	16.508983	13.789591	12.535975
Rh	14.225343	15.219664	12.806038
Rh	12.171292	13.506914	12.573795
Rh	13.151191	11.014611	12.123758
Rh	14.500275	11.051614	9.786790
Rh	16.571366	12.767712	10.043329
Rh	15.578777	15.245734	10.474538
Rh	12.889102	15.058808	10.477928
Rh	12.201556	12.479393	10.062542
Rh	14.323909	13.549976	8.769220
Rh	17.332823	9.297573	12.913118
Rh	18.715784	14.390997	13.705649
Rh	14.045617	17.312516	14.351038
Rh	9.993278	13.869816	13.823894
Rh	11.946636	8.936596	12.944674
Rh	14.612519	9.003012	8.297057
Rh	18.757795	12.405158	8.787723
Rh	16.798138	17.297594	9.602099
Rh	11.412235	16.961084	9.655911
Rh	10.073288	11.835006	8.856675
Rh	14.277005	13.974790	6.260581
Rh	16.627575	13.334893	15.143836
Rh	14.331763	14.851929	15.505470
Rh	12.192092	13.107173	15.185423
Rh	13.188198	10.548069	14.740039

Rh	18.093675	11.843957	13.386401
Rh	14.646516	9.011858	12.966581
Rh	16.025739	9.053378	10.579138
Rh	18.138446	10.798512	10.834340
Rh	16.501174	15.929263	14.064061
Rh	18.819188	13.436410	11.259914
Rh	17.809041	15.951596	11.705044
Rh	11.970707	15.680716	14.199954
Rh	15.439409	17.400620	12.072615
Rh	12.707356	17.227856	11.993553
Rh	10.903143	11.388534	13.456035
Rh	10.618472	15.469109	11.789470
Rh	9.925856	12.831586	11.331257
Rh	10.927034	10.306185	10.903314
Rh	13.260033	8.853241	10.611969
Rh	16.744874	10.644827	8.456071
Rh	12.298748	10.358751	8.506273
Rh	14.433743	11.444051	7.164356
Rh	17.848388	14.896658	9.136779
Rh	16.557029	13.163248	7.424283
Rh	14.091877	17.254173	9.631841
Rh	15.572970	15.711659	7.877596
Rh	10.654527	14.462125	9.222199
Rh	12.812834	15.536313	7.881430
Rh	12.120384	12.914331	7.459700
P	15.980009	16.763062	19.482853
C	14.989394	17.840222	18.337092
C	17.306357	17.906776	20.087328
C	14.897266	16.485430	20.955836
C	14.216748	17.198436	17.314284
C	15.074965	19.248736	18.348011
C	18.467555	18.088206	19.277030
C	17.250318	18.523862	21.355624
C	15.256152	15.445861	21.865371
C	13.687031	17.184119	21.151731
C	13.747748	18.017490	16.241748
C	13.885248	15.753874	17.348506
C	14.436440	20.036976	17.376719
C	19.515122	18.886610	19.775388
C	18.601127	17.465958	17.901850
C	18.307889	19.311343	21.831495
C	14.387191	15.162842	22.936549
C	16.538278	14.650869	21.718372
C	12.836320	16.881764	22.223974
C	13.770815	19.424613	16.303912
C	19.446517	19.494268	21.036315
C	13.189076	15.866114	23.121789
H	15.641801	19.741609	19.140469
H	16.366116	18.384600	21.981055

H	13.404555	17.975138	20.454032
H	12.578152	17.183365	15.318927
H	12.795291	15.597778	17.408076
H	16.225361	15.050398	15.693277
H	14.400507	15.212593	18.154093
H	14.483693	21.127398	17.439075
H	20.408171	19.026151	19.158784
H	19.638936	17.527726	17.543186
H	18.292538	16.408595	17.903037
H	17.962722	17.979150	17.162924
H	18.238470	19.778170	22.817327
H	14.656797	14.365566	23.635731
H	16.674469	14.294242	20.684408
H	17.424155	15.262021	21.959615
H	16.539089	13.780533	22.390367
H	11.904729	17.438402	22.354003
H	13.322227	20.027983	15.510350
H	20.281046	20.102330	21.394835
H	12.534491	15.616668	23.960907

4I′$_{\text{methyl-meta}}$

E = -600.09683 eV

Rh	14.330101	13.073701	11.243380
Rh	14.455321	12.661623	13.793461
Rh	14.562006	12.212642	16.294397
Rh	16.058024	10.740931	14.626941
Rh	15.923775	11.223258	12.036376
Rh	16.462757	13.844630	12.442492
Rh	14.149323	15.181279	12.768297
Rh	12.163271	13.383984	12.584510
Rh	13.212005	10.942784	12.115453
Rh	14.504780	11.032710	9.731285
Rh	16.528390	12.838343	9.943660
Rh	15.426437	15.258207	10.411072
Rh	12.755754	14.971045	10.487097
Rh	12.150207	12.370915	10.071650
Rh	14.207727	13.536692	8.720066
Rh	17.475818	9.380057	12.773866
Rh	18.653672	14.556115	13.571660
Rh	13.979906	17.235662	14.374677
Rh	10.000032	13.626482	13.924002
Rh	12.116820	8.819798	12.960693
Rh	14.662211	9.004581	8.231978
Rh	18.676355	12.569699	8.637695
Rh	16.529670	17.388759	9.549530
Rh	11.192214	16.829957	9.681971
Rh	10.029356	11.665129	8.916931
Rh	14.071145	13.969135	6.232074
Rh	16.670287	13.379971	15.055699

Rh	14.318417	14.846175	15.494384
Rh	12.261390	12.979676	15.207554
Rh	13.330359	10.470678	14.717231
Rh	18.144862	11.970871	13.245598
Rh	14.796159	8.992380	12.896902
Rh	16.117322	9.087312	10.483099
Rh	18.172301	10.918219	10.687455
Rh	16.379878	15.978361	14.016436
Rh	18.758152	13.595058	11.109578
Rh	17.652062	16.056355	11.600427
Rh	11.959730	15.509144	14.219473
Rh	15.253253	17.412443	12.013097
Rh	12.547529	17.157347	12.048067
Rh	10.978886	11.217020	13.509842
Rh	10.481243	15.296633	11.850839
Rh	9.901939	12.651836	11.421376
Rh	10.984778	10.163511	10.956229
Rh	13.354538	8.805880	10.569282
Rh	16.711173	10.736856	8.341540
Rh	12.287649	10.275190	8.503610
Rh	14.344796	11.446328	7.116072
Rh	17.668813	15.038630	9.025747
Rh	16.414376	13.262647	7.326718
Rh	13.839285	17.231885	9.632354
Rh	15.324319	15.759305	7.822321
Rh	10.506453	14.313796	9.299161
Rh	12.592663	15.477196	7.902235
Rh	11.984717	12.832824	7.479034
P	16.058900	16.760767	19.609609
C	15.215871	17.866038	18.376449
C	17.383217	17.863973	20.295713
C	14.868461	16.554422	21.010217
C	14.480677	17.270661	17.303000
C	15.405094	19.266447	18.378445
C	18.586317	18.037232	19.547699
C	17.271278	18.472179	21.564777
C	15.152094	15.567009	22.001024
C	13.648360	17.260772	21.074010
C	14.141520	18.091274	16.180284
C	14.030739	15.852233	17.326979
C	14.942286	20.075711	17.330429
C	19.616993	18.818689	20.105546
C	18.779912	17.430361	18.172916
C	18.313606	19.240343	22.101844
C	14.203960	15.344528	23.018090
C	16.440196	14.768114	21.999255
C	12.717001	17.016888	22.092830
C	14.342730	19.486239	16.206877
C	19.493027	19.416435	21.367169

C	12.997170	16.055301	23.072062
H	15.927959	19.733026	19.215702
H	16.352976	18.344199	22.141513
H	13.423681	18.012740	20.314645
H	11.020757	16.638640	13.032416
H	12.933477	15.790755	17.415248
H	16.230526	15.061695	15.637172
H	14.515403	15.255812	18.115193
H	15.078036	21.159941	17.375729
H	20.540626	18.954632	19.534822
H	19.838146	17.464578	17.875377
H	18.439962	16.382847	18.141006
H	18.199976	17.973669	17.407987
H	18.199437	19.699578	23.087340
H	14.418321	14.589776	23.780925
H	16.670749	14.378685	20.994208
H	17.301164	15.387632	22.301820
H	16.377163	13.919943	22.696373
H	11.779960	17.578822	22.119430
H	14.052269	20.109793	15.356303
H	20.314957	20.012089	21.772372
H	12.280648	15.853892	23.872622

S87-1A$_H$
E = -556.53601 eV

Rh	13.484227	14.029225	17.245421
Rh	13.753516	14.150471	19.778053
Rh	14.003024	14.281588	22.306049
Rh	15.939095	13.105121	20.848548
Rh	15.646328	13.021260	18.213221
Rh	15.235001	15.681118	18.119349
Rh	12.568321	16.126749	18.378052
Rh	11.343315	13.730985	18.613802
Rh	13.244718	11.829780	18.519865
Rh	14.405833	11.894321	16.098277
Rh	15.642906	14.284216	15.815320
Rh	13.734178	16.214624	15.891544
Rh	11.322050	15.007963	16.235657
Rh	11.755747	12.345063	16.335301
Rh	13.200514	13.864162	14.668609
Rh	17.747701	12.011675	19.230061
Rh	16.923392	17.335552	19.018128
Rh	11.658068	18.208010	19.494966
Rh	9.232805	13.453725	19.992918
Rh	12.991412	9.631355	19.828443
Rh	15.286910	9.759999	15.050096
Rh	17.828102	14.592237	14.467716
Rh	13.952762	18.439707	14.707656
Rh	9.198449	15.996245	15.290824

Rh	10.047477	10.686651	15.464030
Rh	12.979483	13.740870	12.112854
Rh	15.523786	15.857358	20.755304
Rh	12.790508	16.293366	21.001969
Rh	11.569441	13.845972	21.253416
Rh	13.480698	11.904944	21.167366
Rh	17.437304	14.701412	19.190278
Rh	15.420275	10.765126	19.591237
Rh	16.600148	10.823020	17.088350
Rh	17.861656	13.309411	16.805322
Rh	14.334436	17.843842	19.325971
Rh	17.477916	15.973218	16.748292
Rh	15.480074	17.973308	16.810329
Rh	10.368560	15.879966	19.811519
Rh	12.784913	18.390358	17.056806
Rh	10.337505	17.157401	17.377351
Rh	11.057127	11.475218	19.972346
Rh	9.089661	14.742613	17.644341
Rh	9.541125	12.015160	17.724082
Rh	11.467264	10.071090	17.632832
Rh	14.174985	9.604734	17.435704
Rh	16.627674	12.132074	14.665809
Rh	12.674735	10.167167	15.151602
Rh	14.149408	11.725240	13.427075
Rh	15.443564	14.232853	13.057420
Rh	11.541811	17.265006	14.872217
Rh	13.441342	16.209299	13.198273
Rh	9.535882	13.339055	15.306080
Rh	11.004561	14.866461	13.560095
Rh	11.460759	12.195321	13.663265
Rh	15.988995	16.600252	14.474137
P	17.071543	16.590754	12.513959
C	17.466114	18.197653	11.677577
C	17.437398	19.396478	12.415796
C	17.783843	18.235042	10.303208
C	17.723985	20.618899	11.788063
C	18.060904	19.458602	9.679055
C	18.032159	20.651465	10.420585
C	15.721592	15.804362	11.527867
C	15.607465	14.503217	10.893715
C	14.556698	16.736891	11.472483
C	14.314123	14.101050	10.328244
C	13.304619	16.309654	10.908343
C	13.178019	14.986226	10.325489
C	18.692281	15.697655	12.555699
C	19.845677	16.482386	12.886203
C	18.884296	14.289079	12.341475
C	21.112912	15.908238	12.949442
C	20.188323	13.729996	12.419815

C	21.293064	14.526467	12.712166
H	17.183332	19.364152	13.479259
H	17.809320	17.311759	9.717923
H	17.700644	21.544559	12.368510
H	18.297624	19.481808	8.612268
H	18.247156	21.603972	9.929905
H	16.489637	13.962162	10.546620
H	14.776438	17.806831	11.505489
H	14.304176	13.218684	9.684203
H	12.548444	17.059581	10.667039
H	12.331416	14.782415	9.664978
H	19.731716	17.550756	13.078098
H	18.076220	13.666901	11.959025
H	21.973191	16.537978	13.188454
H	20.310206	12.661693	12.226676
H	22.291989	14.087664	12.759706
H	15.862032	12.352062	12.999119

S87-TS
E = -555.42377 eV

Rh	13.438972	14.030162	17.254992
Rh	13.732022	14.135347	19.786396
Rh	14.015882	14.259110	22.300916
Rh	15.932360	13.088449	20.815471
Rh	15.620905	13.029995	18.198986
Rh	15.191350	15.690036	18.113385
Rh	12.528533	16.113844	18.403598
Rh	11.316913	13.706002	18.641312
Rh	13.226310	11.822166	18.510466
Rh	14.369039	11.921536	16.077957
Rh	15.581468	14.297570	15.803359
Rh	13.662546	16.220066	15.905738
Rh	11.261672	15.007935	16.281951
Rh	11.713206	12.341426	16.358622
Rh	13.130261	13.868212	14.681787
Rh	17.724772	12.034005	19.190619
Rh	16.886639	17.341574	19.035753
Rh	11.628587	18.193366	19.546544
Rh	9.214579	13.417517	20.036364
Rh	12.985423	9.597660	19.801758
Rh	15.232607	9.795911	14.967602
Rh	17.729207	14.598250	14.369715
Rh	13.838829	18.461730	14.722158
Rh	9.113042	16.000145	15.368596
Rh	9.978368	10.668269	15.514842
Rh	12.881858	13.750538	12.139622
Rh	15.508408	15.855425	20.739359
Rh	12.780099	16.271871	21.019753
Rh	11.563296	13.818749	21.282417

Rh	13.480900	11.886167	21.154872
Rh	17.424734	14.723001	19.120353
Rh	15.410507	10.745114	19.539143
Rh	16.556604	10.836009	17.025658
Rh	17.820329	13.288534	16.759859
Rh	14.301856	17.855760	19.311095
Rh	17.407340	15.992662	16.708915
Rh	15.438672	17.983592	16.803626
Rh	10.351157	15.850051	19.853936
Rh	12.731990	18.391165	17.105984
Rh	10.290387	17.168136	17.421957
Rh	11.045772	11.450884	19.987077
Rh	9.047254	14.730910	17.712410
Rh	9.500950	11.989669	17.769890
Rh	11.417753	10.060676	17.657542
Rh	14.125206	9.609689	17.405786
Rh	16.563432	12.163101	14.624233
Rh	12.616270	10.172929	15.163770
Rh	14.053199	11.730944	13.443831
Rh	15.313533	14.289283	13.089155
Rh	11.465412	17.290981	14.927584
Rh	13.362371	16.241955	13.222824
Rh	9.462936	13.324742	15.373878
Rh	10.913105	14.862932	13.646315
Rh	11.363499	12.205308	13.702874
Rh	15.946798	16.659170	14.495001
P	17.038063	16.570661	12.526064
C	17.489773	18.146129	11.658334
C	17.417753	19.371614	12.348016
C	17.902321	18.130556	10.309153
C	17.755472	20.568345	11.697142
C	18.230261	19.328657	9.661086
C	18.158784	20.548525	10.354499
C	15.674165	15.793808	11.553239
C	15.537704	14.482274	10.936513
C	14.520795	16.740410	11.497956
C	14.244959	14.100507	10.359727
C	13.262754	16.325287	10.939289
C	13.124055	14.999454	10.358287
C	18.618618	15.628395	12.639993
C	19.760920	16.391908	13.091985
C	18.863759	14.197422	12.290429
C	21.047040	15.890894	13.030909
C	20.265884	13.782633	12.103575
C	21.302660	14.589746	12.486963
H	17.093704	19.378968	13.392896
H	17.964891	17.185545	9.762493
H	17.699603	21.515042	12.240485
H	18.542411	19.310940	8.613847

H	18.417041	21.480879	9.846332
H	16.406197	13.909700	10.606937
H	14.752417	17.807850	11.528078
H	14.223494	13.217284	9.716887
H	12.516897	17.081825	10.687298
H	12.273326	14.804161	9.699889
H	19.598343	17.408039	13.456875
H	18.167619	13.745565	11.579066
H	21.880955	16.509491	13.369682
H	20.444351	12.785162	11.695361
H	22.334190	14.248436	12.370490
H	18.475797	13.413704	13.327279

S87-I
E = -555.95492 eV

Rh	13.504557	14.045543	17.255765
Rh	13.815104	14.168673	19.781878
Rh	14.078465	14.293037	22.310021
Rh	16.006814	13.138520	20.831628
Rh	15.711708	13.070819	18.200361
Rh	15.241916	15.719190	18.100386
Rh	12.572420	16.130563	18.396494
Rh	11.393520	13.709628	18.654188
Rh	13.317335	11.846357	18.526157
Rh	14.457250	11.939639	16.101170
Rh	15.660157	14.331256	15.777035
Rh	13.702711	16.236922	15.896134
Rh	11.321241	14.984768	16.276574
Rh	11.792756	12.330315	16.366976
Rh	13.214322	13.868272	14.683267
Rh	17.812776	12.078247	19.216592
Rh	16.894939	17.419322	19.004758
Rh	11.638530	18.192671	19.528041
Rh	9.297859	13.396272	20.039266
Rh	13.127728	9.623681	19.829209
Rh	15.347976	9.809969	14.992495
Rh	17.814446	14.706477	14.417225
Rh	13.879201	18.475266	14.719432
Rh	9.134340	15.935711	15.365524
Rh	10.099477	10.642308	15.522261
Rh	12.942057	13.735406	12.145591
Rh	15.547168	15.888323	20.752410
Rh	12.805870	16.285791	21.013259
Rh	11.641563	13.815587	21.277482
Rh	13.574491	11.909149	21.161001
Rh	17.472558	14.784425	19.163824
Rh	15.513225	10.789141	19.562789

Rh	16.684342	10.896391	17.035908
Rh	17.897537	13.380930	16.780862
Rh	14.310155	17.870351	19.334820
Rh	17.454461	16.039369	16.766759
Rh	15.433964	18.020826	16.811393
Rh	10.384667	15.836395	19.845956
Rh	12.734533	18.398600	17.077586
Rh	10.310015	17.130892	17.415667
Rh	11.138225	11.432580	19.991947
Rh	9.105667	14.678343	17.692272
Rh	9.613231	11.953934	17.782463
Rh	11.565787	10.046154	17.663999
Rh	14.273533	9.637279	17.416137
Rh	16.633891	12.183360	14.637441
Rh	12.749099	10.183880	15.159476
Rh	14.203368	11.767208	13.449626
Rh	15.415656	14.362755	13.087780
Rh	11.490304	17.258871	14.917369
Rh	13.382249	16.235681	13.213619
Rh	9.539864	13.284339	15.350821
Rh	10.976840	14.846661	13.637528
Rh	11.465162	12.184450	13.713602
Rh	15.949030	16.691428	14.443934
P	16.998140	16.699346	12.438216
C	17.424407	18.264053	11.547897
C	17.596364	19.452967	12.284798
C	17.647999	18.273152	10.154174
C	17.998401	20.631637	11.638801
C	18.043984	19.454798	9.512513
C	18.223800	20.633726	10.254252
C	15.651400	15.861679	11.499000
C	15.548201	14.532987	10.916141
C	14.463761	16.761686	11.450183
C	14.266408	14.099519	10.355749
C	13.213267	16.294375	10.918106
C	13.115814	14.961694	10.348411
C	18.500353	15.674795	12.555670
C	19.354489	15.968726	13.699076
C	19.096198	14.799925	11.453297
C	20.118323	14.858737	14.175395
C	20.015662	13.730186	11.993315
C	20.439337	13.746826	13.282815
H	17.405655	19.449174	13.361821
H	17.505290	17.361895	9.566955
H	18.129957	21.549378	12.217683
H	18.210682	19.455711	8.432244
H	18.534209	21.552938	9.751249
H	16.428442	13.965456	10.614863
H	14.652241	17.838304	11.472419

H	14.270558	13.206669	9.725742
H	12.430436	17.017786	10.682075
H	12.265119	14.727956	9.702804
H	19.355854	16.927131	14.222269
H	19.662215	15.454078	10.752994
H	20.691953	14.972149	15.097658
H	20.316054	12.926894	11.315515
H	21.063179	12.938800	13.672928
H	18.315795	14.332946	10.836821

S88-4A’$_{\text{meta-para}}$

E = -600.12558 eV

Rh	15.138572	12.945362	11.197171
Rh	14.992497	12.326518	13.687084
Rh	14.853303	11.640971	16.153782
Rh	16.465526	10.292456	14.508555
Rh	16.582890	10.975347	11.960162
Rh	17.164766	13.525788	12.679059
Rh	14.870887	14.918604	12.924155
Rh	12.852227	13.219486	12.353152
Rh	13.892148	10.779468	11.756026
Rh	15.408881	11.041755	9.510692
Rh	17.444943	12.746062	10.102982
Rh	16.363524	15.150380	10.693301
Rh	13.691320	14.957538	10.497873
Rh	13.077994	12.424315	9.771600
Rh	15.273359	13.639506	8.738445
Rh	18.024822	9.018882	12.709522
Rh	19.217267	14.055176	14.113718
Rh	14.621527	16.860700	14.604474
Rh	10.550917	13.420616	13.458568
Rh	12.664727	8.628790	12.310091
Rh	15.675217	9.167339	7.862450
Rh	19.725325	12.528625	8.999892
Rh	17.602146	17.328185	10.147344
Rh	12.247579	16.929179	9.738001
Rh	11.051343	11.888478	8.337236
Rh	15.413494	14.299058	6.287931
Rh	17.088409	12.876523	15.240150
Rh	14.721072	14.240969	15.460944
Rh	12.642084	12.554483	14.914133
Rh	13.733134	10.086065	14.306925
Rh	18.691733	11.521530	13.473075
Rh	15.354590	8.712361	12.543861
Rh	16.904679	8.992061	10.277450
Rh	18.974469	10.726693	10.849605
Rh	16.971254	15.501541	14.421562
Rh	19.565689	13.313129	11.560818
Rh	18.474185	15.773440	12.150649

Rh	12.491461	15.161215	14.078467
Rh	16.119489	17.174763	12.395289
Rh	13.383003	16.965764	12.183160
Rh	11.540362	10.993156	12.926454
Rh	11.306949	15.249773	11.606551
Rh	10.712079	12.650546	10.888947
Rh	11.772614	10.180198	10.291248
Rh	14.145412	8.786344	10.074032
Rh	17.763955	10.780616	8.363648
Rh	13.314239	10.461071	8.025478
Rh	15.562624	11.687005	6.966237
Rh	18.745192	14.981974	9.537310
Rh	17.621725	13.428252	7.560560
Rh	14.928198	17.228589	9.918497
Rh	16.542078	15.886346	8.153640
Rh	11.569429	14.451674	8.991659
Rh	13.800304	15.677742	7.945585
Rh	13.196512	13.095638	7.214761
P	14.480176	16.893026	19.831761
C	14.277900	17.683787	18.170979
C	16.316616	16.799273	20.093419
C	13.953590	18.214893	21.020851
C	12.948834	17.835558	17.631431
C	15.378343	18.025490	17.366616
C	16.996740	15.581306	19.798397
C	17.043897	17.877396	20.645221
C	13.618391	17.828504	22.351780
C	13.832077	19.570445	20.646351
C	12.794893	18.384177	16.350766
C	11.719814	17.428466	18.412607
C	15.220318	18.583058	16.068016
C	18.382327	15.509521	20.052130
C	16.296105	14.374790	19.212209
C	18.418867	17.780220	20.893720
C	13.179676	18.823307	23.247711
C	13.736020	16.396359	22.834094
C	13.390625	20.542249	21.554484
C	13.903785	18.798005	15.557171
C	19.092550	16.589065	20.591249
C	13.062792	20.165729	22.863297
H	16.390760	17.902341	17.755713
H	16.525193	18.806051	20.890413
H	14.088998	19.871804	19.628439
H	11.786022	18.530723	15.955479
H	10.818427	17.489180	17.786636
H	11.814466	16.395748	18.787649
H	11.566941	18.072707	19.293672
H	16.089339	19.011669	15.562988
H	18.909527	14.578156	19.826014

H	16.911428	13.470911	19.329007
H	15.317572	14.200265	19.684505
H	16.111251	14.502188	18.129113
H	18.957042	18.629581	21.322159
H	12.919549	18.532221	24.269673
H	13.260755	15.692827	22.132098
H	14.791151	16.088233	22.926632
H	13.265619	16.275586	23.820500
H	13.308458	21.585615	21.239476
H	13.738834	19.424490	14.676856
H	20.164991	16.496135	20.781440
H	12.715226	20.910904	23.583314

S88-TS$_{para}$
E = -599.06876 eV

Rh	15.063218	12.907981	10.809534
Rh	14.855570	12.450715	13.339208
Rh	14.655263	11.876403	15.831411
Rh	16.314291	10.460514	14.300826
Rh	16.512807	11.008426	11.727550
Rh	17.029431	13.606344	12.305642
Rh	14.708985	15.024220	12.402204
Rh	12.753166	13.219053	11.882050
Rh	13.822619	10.765040	11.463392
Rh	15.391769	10.917790	9.251609
Rh	17.397826	12.669728	9.783423
Rh	16.276628	15.102371	10.208089
Rh	13.617325	14.864969	9.938950
Rh	13.047116	12.277787	9.362518
Rh	15.259809	13.452125	8.325328
Rh	17.934128	9.114740	12.624358
Rh	19.055135	14.241176	13.756017
Rh	14.381354	16.987326	13.957236
Rh	10.405365	13.475303	12.898743
Rh	12.602728	8.636735	12.098255
Rh	15.718961	8.950329	7.727977
Rh	19.702763	12.424458	8.758194
Rh	17.532807	17.239950	9.545484
Rh	12.161704	16.752797	8.993745
Rh	11.062450	11.648126	7.906169
Rh	15.463322	13.968440	5.820092
Rh	16.906402	13.085803	14.908819
Rh	14.496665	14.424181	14.989577
Rh	12.445169	12.697181	14.457262
Rh	13.589954	10.228253	14.035973
Rh	18.563427	11.671290	13.249609
Rh	15.267057	8.763805	12.403517
Rh	16.873360	8.932003	10.173178
Rh	18.924146	10.711482	10.684585

Rh	16.782981	15.643549	13.927884
Rh	19.463032	13.347589	11.265840
Rh	18.336906	15.836363	11.687454
Rh	12.313547	15.266552	13.472476
Rh	15.983479	17.227817	11.776850
Rh	13.229884	16.990268	11.490484
Rh	11.421660	11.035814	12.536693
Rh	11.219576	15.191330	10.974359
Rh	10.644215	12.562323	10.391033
Rh	11.741669	10.074078	9.968450
Rh	14.119779	8.687856	9.891039
Rh	17.774458	10.623356	8.167642
Rh	13.363553	10.233259	7.720368
Rh	15.605075	11.429970	6.664509
Rh	18.690032	14.893398	9.124304
Rh	17.639556	13.215680	7.204229
Rh	14.842124	17.108135	9.255359
Rh	16.522190	15.670885	7.627364
Rh	11.540464	14.244938	8.404376
Rh	13.795534	15.437073	7.352343
Rh	13.226795	12.827838	6.772071
P	14.557393	17.038071	20.515993
C	14.429833	17.512278	18.736288
C	16.381737	16.941598	20.820944
C	14.001001	18.507808	21.489451
C	13.177861	17.358281	18.065970
C	15.573012	17.830459	17.968319
C	17.072540	15.743145	20.470156
C	17.091666	17.983215	21.456895
C	13.698961	18.331226	22.872775
C	13.779974	19.766934	20.891157
C	13.144688	17.438369	16.660976
C	11.890360	17.100170	18.819949
C	15.526618	17.916689	16.571231
C	18.446825	15.650685	20.765811
C	16.387061	14.584524	19.773744
C	18.458177	17.865595	21.744864
C	13.196186	19.430940	23.594701
C	13.916276	17.010735	23.583824
C	13.272502	20.845165	21.629362
C	14.319311	17.628688	15.895358
C	19.140282	16.693117	21.395424
C	12.979830	20.676351	22.988865
H	16.529246	17.994414	18.469707
H	16.567234	18.901403	21.729017
H	14.009878	19.904395	19.832149
H	12.184895	17.306724	16.154175
H	11.074224	16.838116	18.131584
H	12.012144	16.279481	19.546440

H	11.578555	17.988363	19.394158
H	16.432624	18.171487	16.015006
H	18.981196	14.734096	20.498060
H	17.005938	13.676783	19.823879
H	15.405481	14.366068	20.223737
H	16.206561	14.805050	18.708191
H	18.984035	18.688030	22.236412
H	12.962872	19.300912	24.655776
H	13.499966	16.169862	23.005869
H	14.989835	16.798409	23.721615
H	13.446253	17.017846	24.577891
H	13.110083	21.810458	21.143090
H	14.077569	18.528494	14.345554
H	20.205853	16.585781	21.613765
H	12.581904	21.506854	23.577546

S88-TS$_{meta-5}$
E = -599.24205 eV

Rh	15.419689	13.091914	11.192195
Rh	15.544880	12.516059	13.686635
Rh	15.682477	11.843109	16.168245
Rh	16.873337	10.291410	14.345038
Rh	16.742374	10.986685	11.809271
Rh	17.685291	13.467548	12.374799
Rh	15.598637	15.107599	12.865022
Rh	13.342111	13.641535	12.645182
Rh	14.032866	11.069955	11.956508
Rh	15.279187	11.148003	9.545768
Rh	17.527407	12.617258	9.821390
Rh	16.824605	15.139659	10.451643
Rh	14.130630	15.220226	10.603884
Rh	13.154908	12.755157	10.029562
Rh	15.326557	13.722481	8.708658
Rh	18.043679	8.885315	12.418465
Rh	19.965827	13.746694	13.489137
Rh	15.655588	17.069028	14.515683
Rh	11.269649	14.103955	14.014615
Rh	12.672252	9.072304	12.696928
Rh	15.094978	9.229457	7.911886
Rh	19.609907	12.122273	8.423039
Rh	18.219298	17.125975	9.634203
Rh	12.841307	17.350149	10.007798
Rh	10.930846	12.428579	8.889254
Rh	15.203678	14.330641	6.238622
Rh	17.865393	12.806384	14.940725
Rh	15.813272	14.424880	15.440369
Rh	13.399910	12.977720	15.193885
Rh	14.154292	10.442708	14.536769
Rh	19.085189	11.303083	12.983216

Rh	15.353890	8.869537	12.577871
Rh	16.609583	8.944649	10.124639
Rh	18.885211	10.420074	10.357704
Rh	17.902644	15.484900	14.067986
Rh	19.879441	12.934491	10.953952
Rh	19.171668	15.519414	11.607252
Rh	13.437649	15.625067	14.478695
Rh	17.081652	17.173601	12.130611
Rh	14.293691	17.299356	12.249757
Rh	11.904689	11.541992	13.412927
Rh	11.982796	15.808264	12.044197
Rh	11.017163	13.273539	11.460224
Rh	11.672739	10.694363	10.805338
Rh	13.846187	9.052634	10.291313
Rh	17.403261	10.626388	8.064866
Rh	12.941819	10.783277	8.326024
Rh	15.153087	11.737916	6.978627
Rh	18.978617	14.657573	8.976773
Rh	17.443488	13.246391	7.229384
Rh	15.516218	17.337193	9.842327
Rh	16.743762	15.829003	7.889328
Rh	11.792508	14.987564	9.461363
Rh	13.994888	15.895892	8.040496
Rh	12.992826	13.429510	7.492818
P	14.049061	16.741483	20.101237
C	13.603022	17.075765	18.332763
C	15.900213	16.605444	20.090234
C	13.754627	18.367271	20.942981
C	12.207559	17.104015	17.959166
C	14.585459	17.194878	17.342128
C	16.496786	15.314924	19.989152
C	16.730044	17.737526	20.246035
C	13.747592	18.395847	22.369130
C	13.489148	19.556477	20.230242
C	11.881445	17.352953	16.627115
C	11.105415	16.914080	18.979752
C	14.255385	17.392529	15.965563
C	17.902347	15.220826	20.035173
C	15.678307	14.051643	19.816051
C	18.125166	17.619013	20.291838
C	13.478608	19.618789	23.014146
C	14.044018	17.166530	23.204166
C	13.216729	20.760331	20.894771
C	12.867247	17.543478	15.610477
C	18.714224	16.352593	20.183539
C	13.212376	20.791889	22.295028
H	15.638476	17.165658	17.625573
H	16.277595	18.727205	20.334280
H	13.500156	19.543794	19.138535

H	10.828495	17.454374	16.346912
H	10.123872	16.852805	18.488378
H	11.259570	15.991706	19.563745
H	11.069700	17.744742	19.703518
H	15.297507	18.485906	15.277763
H	18.366365	14.233499	19.955615
H	16.300653	13.159717	19.978723
H	14.827038	14.017757	20.513366
H	15.256808	13.981794	18.797497
H	18.744307	18.511945	20.409986
H	13.474357	19.646034	24.107842
H	13.473027	16.291764	22.853447
H	15.111011	16.892142	23.150091
H	13.797921	17.341668	24.261440
H	13.015265	21.667085	20.318462
H	12.578478	18.062162	14.691788
H	19.801182	16.243100	20.217949
H	13.002543	21.723024	22.827686

S88-I$_{para}$

E = -600.095450 eV

Rh	14.990433	13.173629	10.924564
Rh	14.745500	12.877545	13.483822
Rh	14.516470	12.488845	15.960494
Rh	16.177746	10.916273	14.552903
Rh	16.406096	11.285592	11.960039
Rh	16.976703	13.900069	12.359178
Rh	14.661359	15.316883	12.307310
Rh	12.653882	13.533847	11.896859
Rh	13.734005	11.058920	11.685815
Rh	15.323636	11.061929	9.499477
Rh	17.340408	12.805253	9.920402
Rh	16.256450	15.267377	10.118704
Rh	13.570911	15.032464	9.831812
Rh	13.001451	12.422693	9.463444
Rh	15.229194	13.503757	8.380140
Rh	17.805502	9.443009	12.992004
Rh	18.964077	14.636693	13.779031
Rh	14.337237	17.382241	13.800125
Rh	10.319791	13.871578	12.862326
Rh	12.494849	8.974409	12.431593
Rh	15.664301	8.960415	8.100314
Rh	19.648703	12.455963	8.929698
Rh	17.518414	17.328536	9.372669
Rh	12.135587	16.840541	8.766888
Rh	11.036886	11.693745	8.032711
Rh	15.470602	13.823949	5.847771
Rh	16.794221	13.556245	14.979596
Rh	14.360173	15.019612	15.053522

Rh	12.341640	13.157133	14.502982
Rh	13.493651	10.652747	14.264671
Rh	18.462575	12.034844	13.470020
Rh	15.160211	9.083523	12.755728
Rh	16.805540	9.116927	10.539482
Rh	18.844484	10.914598	10.978806
Rh	16.739028	16.049482	13.836418
Rh	19.414212	13.541066	11.358397
Rh	18.271034	16.068508	11.601972
Rh	12.208659	15.668535	13.339476
Rh	15.914010	17.520861	11.593014
Rh	13.187290	17.264948	11.333354
Rh	11.318402	11.409172	12.724436
Rh	11.176533	15.439524	10.834570
Rh	10.578814	12.749412	10.436962
Rh	11.671151	10.270581	10.216395
Rh	14.042344	8.871149	10.255079
Rh	17.715802	10.649344	8.449424
Rh	13.323761	10.253793	7.981532
Rh	15.580396	11.348011	6.869399
Rh	18.668592	14.956660	9.088999
Rh	17.611355	13.161548	7.307798
Rh	14.821085	17.191113	9.044981
Rh	16.509055	15.664091	7.522524
Rh	11.516759	14.310836	8.322504
Rh	13.778191	15.416970	7.230545
Rh	13.221248	12.754853	6.843218
P	14.648405	16.697399	20.569967
C	14.571804	16.884191	18.728030
C	16.460866	16.771282	20.945896
C	13.983447	18.301407	21.217776
C	13.399149	16.484903	18.046490
C	15.676669	17.372240	17.971768
C	17.229709	15.576055	20.818118
C	17.081627	17.937596	21.444021
C	13.650477	18.390760	22.602492
C	13.723556	19.405329	20.376710
C	13.363569	16.573262	16.619897
C	12.141688	16.051487	18.769264
C	15.641438	17.464474	16.584089
C	18.587555	15.608744	21.189801
C	16.638122	14.286612	20.285288
C	18.433610	17.943714	21.813032
C	13.085030	19.589106	23.080767
C	13.901755	17.252357	23.570544
C	13.154504	20.585402	20.875247
C	14.480747	17.054049	15.851071
C	19.191163	16.772405	21.685056
C	12.835868	20.679053	22.235797

H	16.582375	17.684666	18.496354
H	16.497766	18.854652	21.546063
H	13.973669	19.344546	19.315868
H	12.386008	16.444530	16.142407
H	11.466543	15.503685	18.096449
H	12.377308	15.412134	19.634620
H	11.591426	16.924361	19.161533
H	16.509961	17.871308	16.055951
H	19.180514	14.694270	21.095232
H	17.308231	13.437281	20.481897
H	15.658927	14.073109	20.743737
H	16.475158	14.337284	19.195278
H	18.888838	18.860331	22.196937
H	12.830472	19.662877	24.142159
H	13.545114	16.291967	23.164826
H	14.977827	17.128456	23.778490
H	13.395391	17.434842	24.529515
H	12.967768	21.426003	20.202071
H	14.357190	18.426069	12.131347
H	20.245591	16.760448	21.972559
H	12.392760	21.593117	22.639694

S88-I$_{meta\text{-}5}$

E = -600.055390 eV

Rh	15.421191	13.154935	11.253744
Rh	15.598152	12.556679	13.732526
Rh	15.790798	11.932281	16.200065
Rh	16.971284	10.370762	14.369933
Rh	16.772958	11.036378	11.835459
Rh	17.730296	13.508318	12.344085
Rh	15.625884	15.136936	12.877486
Rh	13.349584	13.658255	12.723431
Rh	14.073296	11.104087	12.034131
Rh	15.258020	11.185709	9.610182
Rh	17.494352	12.667118	9.804960
Rh	16.794109	15.178058	10.427422
Rh	14.086986	15.248994	10.652406
Rh	13.136277	12.767380	10.127874
Rh	15.251979	13.741980	8.747801
Rh	18.108920	8.958039	12.407881
Rh	19.976093	13.846190	13.454880
Rh	15.696084	17.099988	14.549069
Rh	11.299538	14.086981	14.112760
Rh	12.746459	9.082240	12.810280
Rh	15.085838	9.233729	7.982097
Rh	19.552913	12.172745	8.358545
Rh	18.105956	17.189403	9.595483
Rh	12.758911	17.336500	10.057899
Rh	10.886774	12.399501	9.023547

Rh	15.084397	14.335812	6.284788
Rh	17.934292	12.925659	14.902926
Rh	15.798870	14.479028	15.486944
Rh	13.478572	12.981785	15.264069
Rh	14.234448	10.465930	14.602767
Rh	19.154057	11.366327	12.962191
Rh	15.435891	8.915067	12.655597
Rh	16.633550	8.999159	10.155064
Rh	18.917785	10.520963	10.363413
Rh	17.927205	15.573561	14.086006
Rh	19.884067	13.035254	10.897671
Rh	19.125881	15.587217	11.547126
Rh	13.458265	15.662033	14.499941
Rh	16.969921	17.274987	12.151345
Rh	14.209034	17.320491	12.274368
Rh	11.934506	11.547769	13.500006
Rh	11.936931	15.780971	12.102853
Rh	10.994853	13.219731	11.575953
Rh	11.716463	10.661092	10.898375
Rh	13.888861	9.053046	10.392376
Rh	17.365994	10.650901	8.080302
Rh	12.919117	10.776407	8.424971
Rh	15.083864	11.741494	7.033696
Rh	18.931909	14.736189	8.949292
Rh	17.373375	13.265050	7.235209
Rh	15.452595	17.356050	9.848170
Rh	16.649507	15.830182	7.865239
Rh	11.727389	14.942359	9.537944
Rh	13.878294	15.888346	8.086124
Rh	12.916840	13.401472	7.565357
P	14.011479	16.711766	20.099293
C	13.545456	17.026487	18.334097
C	15.861776	16.552664	20.073333
C	13.751795	18.355238	20.919843
C	12.147440	17.181235	17.998000
C	14.496017	16.995926	17.304794
C	16.448642	15.254764	20.024391
C	16.701447	17.683841	20.173758
C	13.720009	18.400522	22.344997
C	13.529647	19.542930	20.189592
C	11.809356	17.461946	16.675820
C	11.057288	17.074279	19.043016
C	14.153769	17.214913	15.929495
C	17.854140	15.152294	20.068729
C	15.620776	13.991009	19.907561
C	18.095622	17.557389	20.218930
C	13.463509	19.635991	22.971274
C	13.975939	17.175558	23.200224
C	13.271045	20.759702	20.834910

C	12.780303	17.540534	15.632459
C	18.675038	16.282908	20.165005
C	13.236561	20.806133	22.234480
H	15.545699	16.839414	17.564863
H	16.257373	18.679885	20.221186
H	13.560740	19.516994	19.098336
H	10.763955	17.673825	16.429008
H	10.064117	17.085198	18.571961
H	11.156597	16.140982	19.621652
H	11.097546	17.902458	19.768709
H	17.537873	17.398046	13.879977
H	18.310949	14.159045	20.030103
H	16.235854	13.102371	20.111690
H	14.768421	13.995553	20.604245
H	15.200042	13.879486	18.892363
H	18.721565	18.450027	20.295867
H	13.436993	19.675717	24.064314
H	13.394867	16.307685	22.849516
H	15.038417	16.880318	23.169273
H	13.714436	17.369386	24.250506
H	13.100970	21.663844	20.244657
H	12.502950	18.060382	14.710014
H	19.761302	16.167060	20.201275
H	13.032895	21.746782	22.752463

8. References

[1] F. Martinez-Espinar, P. Blondeau, P. Nolis, B. Chaudret, C. Claver, S. Castillón, C. Godard, NHC-stabilised Rh nanoparticles: Surface study and application in the catalytic hydrogenation of aromatic substrates *J. Catal.* **2017**, *354*, 113-127.

[2] C. R. Hilliard, N. Bhuvanesh, J. A. Gladysz, J. Blümel, Synthesis, purification, and characterization of phosphine oxides and their hydrogen peroxide adducts *Dalton Trans.* **2012**, *41*, 1742-1754.

[3] F. Dornhaus, M. Bolte, H.-W. Lerner, M. Wagner, Phosphanylborohydrides: First Assessment of the Relative Lewis Basicities of $[BH_3PPh_2]^-$, CH_3PPh_2, and $HPPh_2$ *Eur. J. Inorg. Chem.* **2006**, 1777-1785.

[4] a) Hohenberg, P.; Kohn, W. Inhomogeneous electron gas *Phys. Rev.* **1964**, *136*, 864-871. b) Kohn, W.; Sham, L. J. Self-Consistent Equations Including Exchange and Correlation Effects *Phys. Rev.* A **1965**, *140*, 1133-1138.

[5] a) Kresse, G.; Hafner, J. *Ab initio* molecular dynamics for liquid metals *Phys. Rev. B* **1993**, *47*, 558-561. b) Kresse, G.; Furthmüller, J. Efficiency of ab-initio total energy calculations for metals and semiconductors using a plane-wave basis set *Comput. Mat. Sci.* **1996**, *6*, 15-50. c) Kresse, G.; Furthmüller, J. Efficient iterative schemes for *ab initio* total-energy calculations using a plane-wave basis set *Phys. Rev. B* **1996**, *54*, 11169-11186.

[6] Hammer, B.; Hansen, L. B.; Norskov, J. K. Improved adsorption energetics within density-functional theory using revised Perdew-Burke-Ernzerhof functionals *Phys. Rev. B* **1999**, *59*, 7413-7421.

[7] Kresse, G.; Joubert, D. From ultrasoft pseudopotentials to the projector augmented-wave method *Phys. Rev. B* **1999**, *59*, 1758-1755.

[8] Methfessel, M.; Paxton, A. T. High-precision sampling for Brillouin-zone integration in metals *Phys. Rev. B* **1989**, *40*, 3616-3621.

[9] Dimer method: Henkelman, G.; Jonsson, H. A dimer method for finding saddle points on high dimensional potential surfaces using only first derivatives *J. Chem. Phys.* **1999**, *111*, 7010-7022. Improved Dimer method: Heyden, A.; Bell, A. T.; Keil, F. J. Efficient methods for finding transition states in chemical reactions: Comparison of improved dimer method and partitioned rational function optimization method *J. Chem. Phys.* **2005**, *123*, 224101.
